# Novel insight on marker genes and pathogenic peripheral neutrophil subtypes in acute pancreatitis

**DOI:** 10.3389/fimmu.2022.964622

**Published:** 2022-08-22

**Authors:** Deyu Zhang, Meiqi Wang, Yang Zhang, Chuanchao Xia, Lisi Peng, Keliang Li, Hua Yin, Shiyu Li, Xiaoli Yang, Xiaoju Su, Haojie Huang

**Affiliations:** ^1^ Department of gastroenterology, First Affiliated Hospital, Naval Medical University, Shanghai, China; ^2^ Academy of Medical Sciences, Zhengzhou University, Zhengzhou, China

**Keywords:** acute pancreatitis, neutrophil, COVID-19, biomarkers, WGCNA, single-cell sequencing

## Abstract

Acute pancreatitis is a common critical and acute gastrointestinal disease worldwide, with an increasing percentage of morbidity. However, the gene expression pattern in peripheral blood has not been fully analyzed. In addition, the mechanism of coronavirus disease 2019 (COVID-19)-induced acute pancreatitis has not been investigated. Here, after bioinformatic analysis with machine-learning methods of the expression data of peripheral blood cells and validation in local patients, two functional gene modules in peripheral blood cells of acute pancreatitis were identified, and S100A6, S100A9, and S100A12 were validated as predictors of severe pancreatitis. Additionally, through a combination analysis of bulk sequencing and single-cell sequencing data of COVID-19 patients, a pivotal subtype of neutrophils with strong activation of the interferon-related pathway was identified as a pivotal peripheral blood cell subtype for COVID-19-induced acute pancreatitis. These results could facilitate the prognostic prediction of acute pancreatitis and research on COVID-19-induced acute pancreatitis.

## Introduction

Acute pancreatitis (AP) is a clinically common critical and acute disease that occurs in approximately 13 million people worldwide every year (1). With the exacerbation of alcohol abuse and obesity worldwide, the morbidity of AP has surged in recent years. According to the Atlanta criteria for the classification of AP, AP can be divided into three subtypes based on the degree of illness, including mild acute pancreatitis (MAP), moderate severe acute pancreatitis (MSAP), and severe acute pancreatitis (SAP). The key differentiator among these three types is AP with or without transient organ failure or persistent organ failure (2). In general, 20% of patients have SAP, and the mortality rate is as high as 20%–40%. However, reliable diagnostic and prognostic predictors of AP in the transcriptome of peripheral blood cells have yet to be clarified.

Additionally, a great number of reports indicate that coronavirus disease 2019 (COVID-19) infection can potentially result in AP (3, 4). Some single-center studies have confirmed this relationship between COVID-19 and AP (5, 6). Some literature reviews have suggested that there is an increased prevalence of AP in patients infected with COVID-19 and that severe acute respiratory syndrome coronavirus 2 (SARS-CoV-2) might itself cause AP in some patients (7, 8). However, the pathogenesis of AP concomitance with COVID-19 infection remains unclear.

Neutrophils are a type of immune cell enriched in peripheral blood that originate and differentiate in bone marrow and are then released from bone marrow into blood (9). In blood, neutrophils can monitor antigens, pathogens, and tissue inflammation. Once inflammation or antigen signals are detected, inactivated neutrophils can transition to various activated phenotypes. Most of the activated neutrophils are proinflammatory. However, the characteristics of activated neutrophils vary depending on the immunogenicity of pathogens (10). Neutrophils play an important role in AP. A previous study proved that neutrophils mediate further activation of trypsinogen by-products of Dihydronicotinamide adenine dinucleotide phosphate (NADPH) oxidase, exacerbate pancreatic injury, and even cause lung injury (11). Interestingly, neutrophils are also an important pathogenic factor in COVID-19 infection. COVID-19 triggers a severe pandemic with a multisystem inflammatory disorder. The characteristic of this disease is an acute syndrome in the respiratory system with cytokine-driven hyperinflammation and extensive transcriptional changes in leukocytes. Among them, neutrophils have been proven to be linked to COVID-19 immunopathogenesis, including a dysfunctional interferon (IFN) response and myeloid inflammation. However, the relationship between neutrophils and these two diseases has not been fully clarified.

Some case reports have shown that IFN has a strong relationship with AP (12). Additionally, IFN-γ could promote AP in a rat model (13). A meta-analysis systematically reviewed the literature related to the occurrence of AP after IFN treatment (AP-IFN), and the results indicated that AP and IFN have a probable or definite causal relationship (14). However, few studies have aimed to identify the potential pathogenic roles of IFN and clarify the pathogenic roles of neutrophils in COVID-19-induced AP.

Bioinformatic analysis of high-throughput sequencing data plays an important role in medical research. In fact, there are some studies based on bioinformatic analysis in AP (15–18). However, all of these analyses are based on the identification of differentially expressed genes (DEGs), and this method could result in the omission of large-scale information from unrecognized genes. Additionally, most of the studies are based on bulk sequencing data from the mouse pancreas, and studies based on human blood are rare. Weighted gene coexpression network analysis (WGCNA) is a novel bioinformatic method used to identify gene sets (gene modules) with similar expression patterns and to analyze the connection between gene sets and sample phenotypes. WGCNA can map the regulatory network among genes in gene sets and identify key regulatory genes without using differential gene analysis. Suitable for complex transcriptome data, WGCNA can be used to study developmental regulation at different stages and response mechanisms at different time points of biological and abiotic stresses. Single-cell sequencing is another novel bioinformatic analysis procedure. Traditional bulk sequencing examines the genome of a population of cells, such as cell cultures, tissues, organs, or entire organisms. Its output is the average genome of a cell population, whereas single-cell sequencing measures the genome of a single cell in a cell population (19). Using single-cell sequencing, we can identify new subpopulations, or cellular states, in a seemingly homogeneous population of cells.

In our current analysis, we performed WGCNA of sequencing data from more than 100 human blood samples divided into a healthy group, MAP group, MSAP group, and SAP group for the first time and identified functional gene modules. Then, we identified the pivotal genes in the functional gene modules with significant diagnostic value through a machine-learning method. Finally, we investigated the potential mechanism and pathogenic neutrophil subtype in COVID-19-induced AP.

## Materials and methods

### Data screening

High-throughput bulk sequencing datasets related to peripheral blood of AP and COVID-19 infection were screened in the Gene Expression Omnibus (GEO) database (http://www.ncbi.nlm.nih.gov/geo/). The GSE194331 dataset, which includes peripheral blood gene expression data from 87 patients with AP of varying severity (mild = 57, moderate to severe = 20, and severe = 10) within 24 h of presentation to the hospital and peripheral blood gene expression data from 32 healthy controls, was used in this study (18). Additionally, to explore the common biological mechanism between AP and COVID-19, the GSE152418 dataset was used in the following analysis as the COVID group, including 17 COVID-19 subjects and 17 healthy controls (20). Then, to identify the transcriptional change in neutrophils between COVID-19 and normal sepsis, the single-cell sequencing dataset GSE157789, which includes 14 samples of COVID-19-induced sepsis after 72 h, three samples of bacteria-induced sepsis after 72 h, seven samples of COVID-19-induced sepsis after 7 days, and two samples of bacteria-induced sepsis after 7 days, was analyzed. Five healthy controls in single-cell sequencing datasets were excluded from this study (21).

### Identification of differentially expressed genes

The raw gene count tables from the GSE194331 dataset were acquired, and ensemble gene names were converted into gene symbols using the org.Hs.eg.db package in R software. Then, the sum of each gene count number in each dataset was calculated, and low expression genes were excluded in the following research (sum of gene count number <100). Additionally, each table was subjected to differential expression analysis to compare COVID-19 *vs*. healthy controls using the DESeq2 package in R software (3). The criteria for differential expression analysis were |logFC| >1 and P value <0.05.

### Gene ontology and pathway enrichment analysis

Gene Ontology analysis, pathway enrichment, and hub pathway identification were executed using the Metascape online tool (https://metascape.org/) (22). Hub genes were identified through the STRING database (https://cn.string-db.org/) with a threshold score of 0.7, and protein−protein interactions (PPIs) were visualized using Cytoscape software. The cytoHubba plug-in was used to identify hub genes with their ranks (23).

### Weighted gene coexpression network analysis

To cluster the common modules among different subtypes of AP, WGCNA was executed on the GSE194331 dataset using the WGCNA package in R software. The significant advantage of WGCNA is grouping DEGs in modules by coexpression analysis and screening and identifying specific coexpression gene modules with significant correlations among different subtypes of AP. During the analysis, first, an outlier specimen was identified and excluded. Second, the coexpression network with soft thresholding power was constructed to obtain a higher level of scale-free R2 and mean connectivity (24). In the dynamic tree cut and module identification section, we chose 17 as the minimum number of gene modules. Clinical characteristic data of BD samples and COVID-19 samples from the GSE198533 and GSE152418 datasets were acquired, and then we calculated the gene significance (GS) and module membership (MM) through WGCNA. Then, the relationship between gene modules and clinical traits was represented in the form of a heatmap. After that, the module with coexpression patterns and significance was identified.

### Random forest algorithm manipulation

Random forest is a robust clustering supervised machine-learning algorithm for hub gene identification, and it can be used to calculate the significance of predictive variables distinguished from background noise (25). In the current study, a random forest algorithm was used to identify hub genes of two identified modules based on WGCNA through the randomForest package in R software (ntree = 1,500), grouping by healthy control, MAP, and MSAP&SAP.

### Immune infiltration estimation and receiver operating characteristic curve analysis

To estimate the immune subtype changes between the peripheral blood of AP patients and the peripheral blood of healthy controls, Immune Cell Abundance Identifier (ImmuCellAI) (http://bioinfo.life.hust.edu.cn/ImmuCellAI/) was used. ImmuCellAI is a tool to estimate the abundance of 24 immune cells from gene expression datasets, including RNA-Seq and microarray data, in which the 24 immune cells comprise 18 T-cell subtypes and 6 other immune cells: B cells, natural killer cells (NK cells), monocytes, macrophages, neutrophils, and dendritic cells (DCs) (26). Receiver operating characteristic (ROC) curves were rendered on DEGs using the R package pROC.

### Patient sample enrollment

Peripheral blood was obtained from AP patients within 24 h of admission to Changhai Hospital from 2017 to 2020. Ethics approval was obtained from the Shanghai Changhai Hospital Ethics Committee. AP diagnosis was made at the time of presentation to the emergency department. Severity was defined according to the Revised Atlanta classification. Specifically, patients were defined as having SAP if they developed persistent organ failure beyond 48 h. Patients were defined as having MSAP if they developed transient organ failure and/or local or systemic complications. Finally, 28 samples were included in our PCR analysis, including 14 MAP, 8 MSAP, and 6 SAP samples. These samples are divided into two groups, including the MAP and MSAP&SAP groups. The details of the patients are listed in **Table S1**.

### Polymerase chain reaction

Then, peripheral blood mononuclear cells (PBMCs) were isolated from peripheral blood by Ficoll density gradient centrifugation. RNA from PBMCs was isolated with TRIzol reagent (Invitrogen, USA). The concentration of the RNA was analyzed using a Nanodrop 1000 spectrophotometer (Thermo Fisher, USA) and then transcribed into cDNA using a high-capacity cDNA reverse transcription kit (Life Technology cooperation, USA) and amplified. The primer sequences, number of cycles, and annealing temperature are listed in **Table S2**. The expression levels were transformed into standard β-actin content and calculated by the 2^-ΔΔCt^ method. Then, GraphPad Prism 8 software was used to visualize the expression data, and a paired t test was executed to identify the paired groups with significance.

### Analysis of single-cell sequencing data

The single-cell transcriptome matrix dataset GSE157789 was acquired from the GEO database. The number of genes detected in each cell was limited from 0 to 8,000. Then, ribosome Unique Molecular Identifiers (UMI) rates below 60% and mitochondrial UMI rates below 20% passed cell quality filtering and were considered mitochondrial genes (**Figure S2**). The Seurat software package (version: 3.1.4, https://satijalab.org/seurat/) was used to perform cell normalization and regression to obtain scaled data. The standard for PCA construction was the top 2,000 highly variable genes, and the basis for the construction of t-distributed Stochastic Neighbor Embedding (tSNE) and Uniform Manifold Approximation and Projection (UMAP) was the top 10 PCA. We acquired the unsupervised cell cluster result based on the top 10 principal functions through the graph-based cluster method. The FindAllMarkers function and the Wilcoxon rank sum test algorithm were used to calculate the marker genes (logFC >0.25; p-value <0.05; min.pct >0.1). Genes with the top 10 log fold change (logFC) are visualized.

### Identification of significantly related pathways in different neutrophil cell types

To assess whether the gene set is enriched in a neutrophil cell subpopulation, the “irGSEA” package (https://github.com/chuiqin/irGSEA/) in R software was used. We used this package to score individual cells using multiple gene set enrichment methods and to generate a multiple gene set enrichment score matrix. Then, we used the Wilcoxon test to calculate the DEG sets of each cell subpopulation in the enrichment fraction matrix of each gene set. Some specific enriched pathways were marked and visualized in single plots.

### Pseudotime analysis

Monocle2 (http://cole-trapnell-lab.github.io/monocle-release) was used to execute the single-cell trajectory analysis utilizing DDR-Tree and default parameters. We selected marker genes of the Seurat (version: 3.1.4) clustering result and raw expression counts of the cell passed filtering. On the basis of pseudotemporal analysis, the branch expression analysis model (BEAM Analysis) was used to analyze branch fate-determining genes.

### Identification of significant metabolic pathways at the single-cell level

The “ScMetabolism” package in R software was used to calculate the metabolism status among different cell types in neutrophil datasets. “ScMetabolism” was designed to easily quantify single-cell metabolic activity using a single-line command and combining the published gene sets and manually reviewed gene sets from the Kyoto Encyclopedia of Genes and Genomes (KEGG) database and the Reactome database to generate the list of metabolic gene sets (27).

### Subtypes from single-cell sequencing estimation in bulk sequencing data from peripheral blood of acute pancreatitis patients and healthy controls

The downloaded bulk sequencing data (GSE194331) and neutrophil subtype matrix acquired from Seurat analysis were uploaded to cibersoftx (https://cibersortx.stanford.edu/runcibersortx.php). The relative proportion of Group 0 subtypes in the GSE194331 dataset was acquired through cibersoftx deconvolution analysis. Visualization of the proportion of the targeted subgroup in each group, including healthy controls, patients with MAP, patients with MSAP, and patients with SAP, was performed using GraphPad Prism 8. The significance analysis between each group was performed using a t test.

## Results

### Identification of differentially expressed genes with significant pathways and hub genes

**Figure 1:**
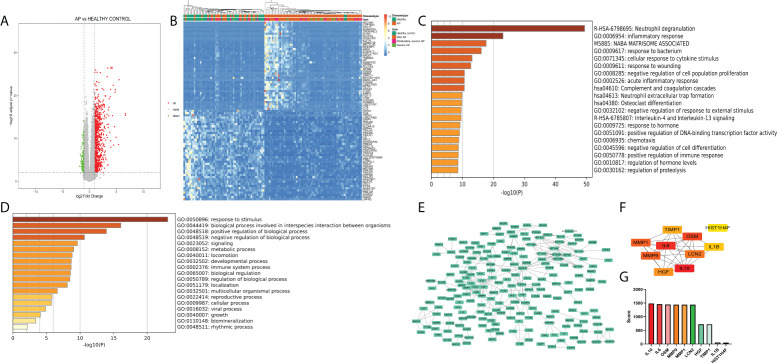
**(A)** Volcano plot of peripheral blood of acute pancreatitis datasets (logFC >1, p < 0.05). **(B)** Heatmap of the top 50 upregulated and downregulated genes with unsupervised clustering analysis. **(C)** Bar graph of enriched terms across input gene lists, colored by p-values. **(D)** Terms of the Gene Ontology list, colored by p-values. **(E)** Protein−protein interaction network of DEGs. **(F)** Network of the top 10 genes. **(G)** Enriched score of the top 10 genes.

The GSE194331 dataset, which includes mRNA expression data from peripheral blood of AP patients as the disease group (57 mild pancreatitis samples, 20 moderate severe pancreatitis samples, and 10 severe pancreatitis samples) and healthy volunteers as the healthy control group (32 samples), was subjected to differential expression analysis through the DEseq2 package (logFC >1, p < 0.05) (**Figure 1A**), and 1,064 DEGs were identified (**Table S3**). As shown in **Figure 1B**, the transcriptional expression of the top 50 upregulated and downregulated genes could distinguish most of the disease samples, especially severe samples, from healthy samples (**Figure 1B**). “Neutrophil degranulation” is a significant pathway identified in the bar graph of enriched terms across input gene lists (**Figure 1C**). “Response to stimulus” is the most distinct term in Gene Ontology analysis (**Figure 1D**). These terms emphasize the pivotal value of neutrophils in the occurrence of AP. PPI analysis indicated that there were some modules with significant density, including a module with IL-1R1, IL-1RN, IL-1R2, IL-10, and MMP1, a module with HIST family genes, and another module with MAPK-related genes (**Figure 1E**). IL-10, IL-6, OSM, MMP9, MMP1, LCN2, HGF, TIMP1, IL1B, and HIST1H4F were identified as the top 10 enriched genes (**Figures 1F, G**).

### Identification of pivotal gene modules in acute pancreatitis through Weighted Gene Co-Expression Network Analysis (WGCNA)

**Figure 2:**
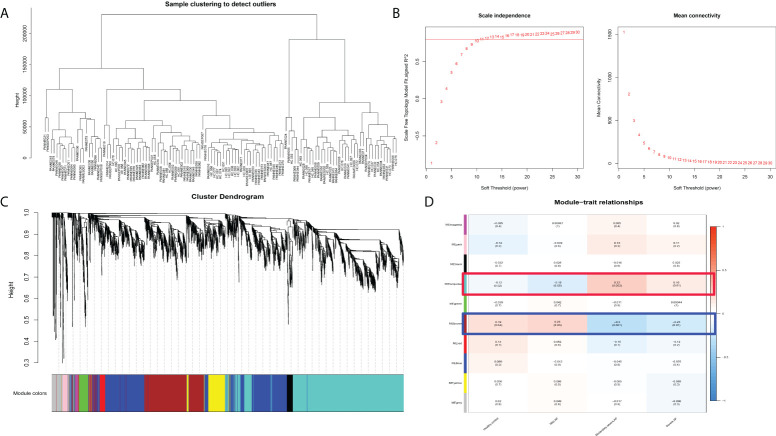
WGCNA results in different subtypes of acute pancreatitis. **(A)** Sample dendrogram. **(B)** The scale plot of WGCNA to identify optimal vector power (cutoff value = 0.8). **(C)** Sample dendrogram and trait heatmap. **(D)** Module–trait relationships: every module has its correlation coefficient and corresponding p-value.

To further clarify the potential mechanism and gene module among normal peripheral blood and differential subtypes of AP (MAP, MSAP, and SAP), WGCNA was executed on disease samples of the two datasets after batch normalization (**Figure 2A**). As shown in **Figures 2B, C**, all samples were included, and the optimal vector power was set at 11. The brown module and turquoise module are two significant gene modules with negative and positive correlations with the severity of AP, respectively (**Figure 2D**). Other gene modules had compact gene regulatory networks (**Figure 5C**). The gene list of the brown module and turquoise module is shown in **Table S4**. The WGCNA results suggest that genes in the brown module and turquoise module manipulate common biological processes in AP.

### Investigation of pivotal gene ontology terms and pathways in screened gene modules

**Figure 3:**
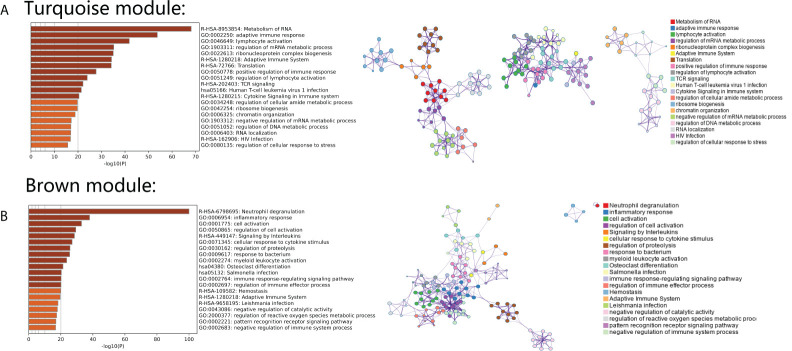
**(A)** Gene Ontology analysis and KEGG pathway analysis with an interaction network in genes from the turquoise module. **(B)** Gene Ontology analysis and KEGG pathway analysis with an interaction network in genes from the brown module.

To further investigate the gene functions and significant KEGG pathways in the identified modules, the Metascape database was used. “Metabolism of RNA” pathways related to T-cell activation and virus infection were significantly enriched in the turquoise module. The pathway network of the turquoise module revealed that these three aspects of related pathways acted independently (**Figure 3A**). Additionally, “Neutrophil degranulation,” “Inflammatory response,” and “Response to bacterium” were significantly enriched in the brown module. The pathway network of the turquoise module reveals that these related pathways act synergistically (**Figure 3B**).

### Identification of immune cell subtypes and reliable biomarkers in acute pancreatitis

**Figure 4:**
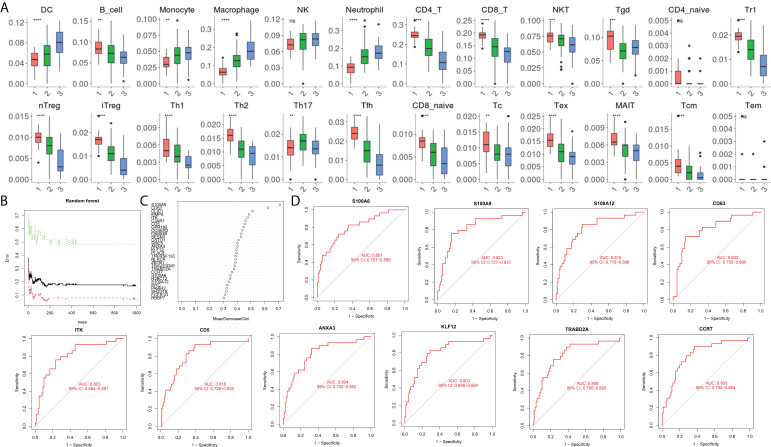
**(A)** Estimation of immune cell subtype infiltration in all samples, divided into three groups (1 = healthy control group, 2 = MAP group, and 3 = MSAP&SAP group). **(B)** Error plot of random forest analysis (Tree = 1,500). **(C)** Top 30 genes in random forest analysis. **(D)** ROC plot of genes with significant diagnostic value (AUC >0.8). **P < 0.01, ***P < 0.001, ****P < 0.0001, ns, P > 0.05.

During immune cell-type infiltration analysis, the results indicated that with the progression of AP, peripheral blood DCs, monocytes, macrophages, and neutrophils showed an increasing tendency, and B cells, CD4 T cells, CD8 T cells, Treg cells, T helper (Th) cells, Th1 cells, Th2 cells, and Tfh cells showed a decreasing tendency (**Figure 4A**). Then, random forest analysis was executed between the severe AP groups (MSAP&SAP) and the control group (healthy control and MAP) (**Figure 4B**). The top 30 genes are listed (**Figure 4C**). Among these genes, 10 genes, including *S100A6, S100A9, S100A12, CD63, ITK, CD5, ANXA3, KLF12, TRABD2A,* and *CCR7,* were identified with significant diagnostic value (AUC >0.8) (**Figure 4D**).

### Validation of reliable biomarkers to monitor the severity of acute pancreatitis

**Figure 5:**
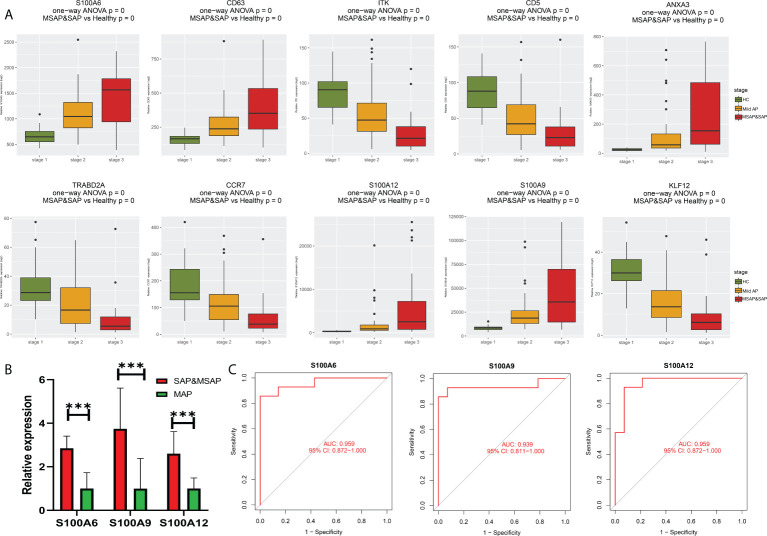
**(A)** Bar plot of 10 identified genes with prognostic value in the current datasets. **(B)** Expression of S100A6, S100A9, and S100A12 in the peripheral blood of our local cohort. **(C)** ROC plot of S100A6, S100A9, and S100A12 based on the peripheral blood expression of our local cohort. ***P < 0.001.

As shown in **Figure 5A**, all eight genes showed significant differences between the healthy control group, MAP group, and MSAP&SAP group. Genes with higher expression are more suitable for use as diagnostic factors because of the lower detection error rate. Following this criterion, three genes were screened with significant value as diagnostic factors (minimum relative expression >500), including S100A6, S100A9, and S100A12. To test the robustness of these genes for the identification of AP severity, 28 peripheral blood samples were collected, and the relative expression of these genes was detected. The details of the enrolled patients are listed in **Table S1**. The patients were grouped by the severity of AP (MSAP&SAP group and MAP group). Significant differences were identified between these two groups in the expression value of all genes (**Figure 5B**). ROC analysis also verified these genes with significant diagnostic value between SAP and MAP (**Figure 5C**).

### Identification of common biological processes and pivotal peripheral blood cell types between acute pancreatitis and COVID-19

**Figure 6:**
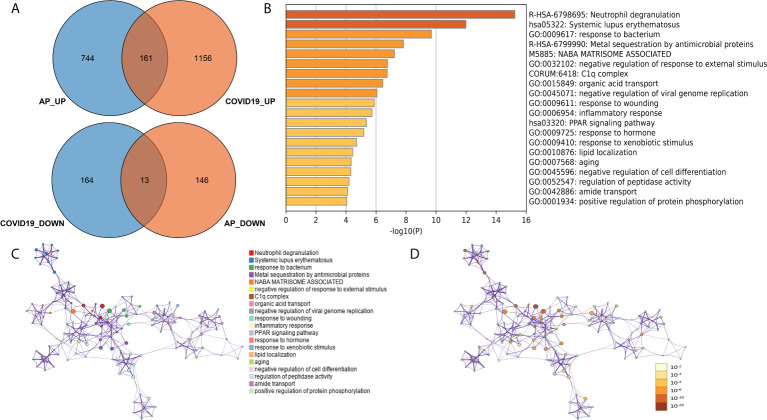
**(A)** Common DEGs between the peripheral blood of acute pancreatitis and COVID-19 patients. **(B)** Gene Ontology and KEGG pathways in the identified common DEGs. **(C)** Gene Ontology and KEGG pathway interaction network with each term. **(D)** Gene Ontology and KEGG pathway interaction network with p-values.

To further explore common biological processes and pivotal cell types in COVID-19-induced pancreatitis, the GSE152418 dataset was used in the following study. A total of 1,494 DEGs were identified through the DEseq2 package (**Figure S1**; **Table S5**). A total of 161 common upregulated genes and 12 downregulated genes were identified between the peripheral blood of AP and COVID-19 patients (**Figure 6A**). “Neutrophil degranulation” was significantly enriched in the KEGG pathway analysis of these common genes (**Figure 6B**). The “neutrophil degranulation” pathway was identified in the central roles of networks with the most significant value (**Figures 6C, D**).

### Identification of neutrophil cell types in single-cell datasets

**Figure 7:**
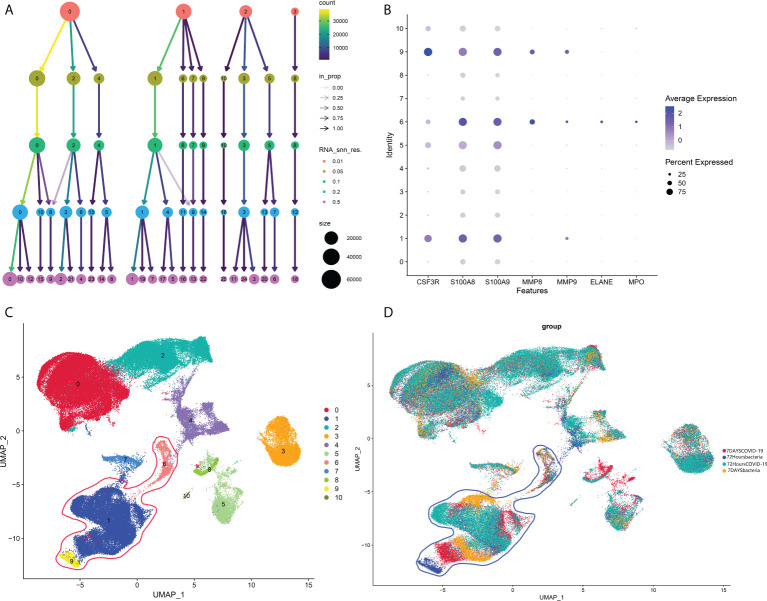
**(A)** Cluster tree at different resolutions. **(B)** Neutrophil markers in different subtypes of peripheral blood. **(C)** Identified neutrophil subtypes in the UMAP plot with subtypes. **(D)** Identified neutrophil subtypes in the UMAP plot with groups.

As mentioned in the introduction, a previous study showed that COVID-19-induced sepsis can potentially induce and accelerate AP (5–8). However, there is no report related to bacteria-induced sepsis, another common cause of sepsis. According to our analysis above, neutrophils could be an important subtype as a common pathogenic factor between COVID-19 infection and AP in peripheral blood. Hence, we next investigated the change in neutrophils between bacteria-induced sepsis and COVID-induced sepsis by analyzing single-cell sequencing data to identify the pathogenic factor of peripheral blood in COVID-19-induced AP. As shown in **Figure 7A**, 0.05 was selected as the resolution in the following steps. Groups 1, 6, and 9 were identified as neutrophils (**Figures 7B–D**).

### Identification of different neutrophil cell types

**Figure 8:**
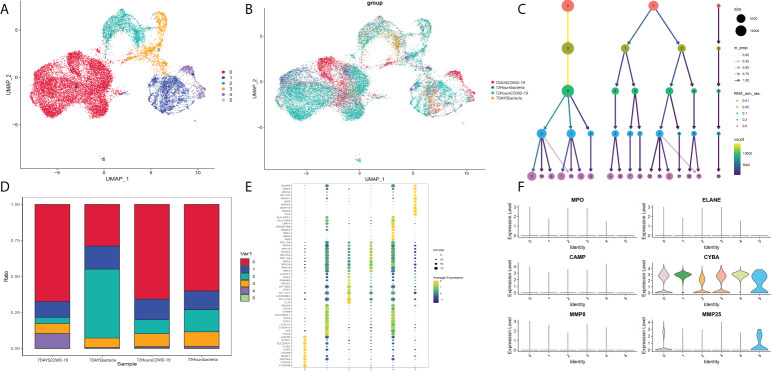
**(A)** Neutrophil markers in different subtypes of peripheral blood. **(B)** Identified neutrophil subtypes in the UMAP plot with subtypes. **(C)** Cluster tree at different resolutions. **(D)** Stacked plot of neutrophil subtypes. **(E)** Heatmap of the top 10 genes in each subtype. **(F)** Markers of neutrophil degranulation in each subtype.

Then, the identified neutrophils were screened and extracted into the following analysis. Five neutrophil subgroups were identified (**Figures 8A–C**). As shown in **Figure 8D**, Group 0 was significantly upregulated in the COVID-19 group, and Group 3 was significantly increased in bacteria-induced sepsis. The top 10 genes in each group were screened out (**Figure 8E**). Importantly, the top genes in Group 0 were associated with the IFN-reactive phenotype and inflammation. Groups 3 and 4 were more likely to exhibit a proliferative phenotype. Neutrophil degranulation is significantly related to the activation of neutrophils. The marker genes of neutrophil degranulation are MPO, ELANE, CAMP, CYBA, MMP8, and MMP25. As shown in **Figure 8F**, marker genes of neutrophil degranulation were significantly upregulated in Group 0. In summary, Group 0 represents a subtype of mature activated neutrophils, mainly enriched in COVID-19-induced sepsis, with high expression of IFN-related genes.

### Group 0 neutrophils are a potential pathogenic subtype of acute pancreatitis related to interferon secretion and are upregulated in the peripheral blood of patients with acute pancreatitis

**Figure 9:**
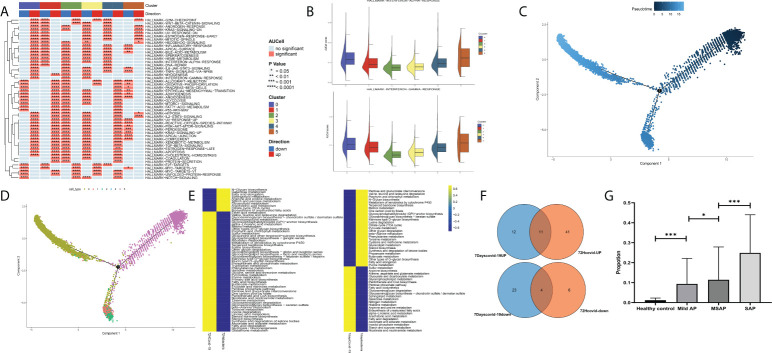
**(A)** Single-cell pathway analysis of the five subgroups. **(B)** The activated interferon-related pathway among all subgroups. **(C)** Pseudotime locus analysis of the five subgroups sorted by pseudotime. **(D)** Pseudotime locus analysis of the five subgroups sorted by subgroup. **(E)** Single-cell metabolism-related pathway analysis in Group 0 between the COVID-19-induced sepsis group and the bacteria-induced sepsis group. **(F)** The common identified metabolism-related pathways in Group 0. **(G)** The proportion of the identified subtype from single-cell sequencing data in all neutrophils among acute pancreatitis patients and healthy controls (based on the GSE194331 dataset) (*p < 0.05, ***p < 0.0001).

Finally, after validation through single-cell pathway analysis, Group 0 was identified as an IFN-related group (**Figures 9A, B**). Pseudotime locus analysis illustrated that Group 5 represents the naive and inactivated phenotypes of neutrophils in sepsis, and the evolutionary trajectory of Group 0 was different from that of other mature neutrophils (**Figures 9C, D**). Then, some common metabolism-related pathways were identified in Group 0, including “fatty acid degradation,” “alpha-linolenic acid metabolism,” “sulfur metabolism,” and “fatty acid biosynthesis” (**Figures 9E, F**; **Table S6**). Finally, to further validate the identified IFN-related neutrophil pathogenic subtype, cibersoftx software was used to estimate the proportion of the identified subtype from single-cell sequencing data in all neutrophils among AP patients and healthy controls (based on the former GEO bulk sequencing dataset: GSE194331). The proportion of this subtype was nearly 0 in peripheral blood from healthy controls and was significantly upregulated in peripheral blood of AP patients, rising with increasing disease severity (**Figure 9G**).

## Discussion

AP is a common clinical acute abdominal disease with pancreatic inflammation, including pancreatic edema, bleeding, and even necrosis. The clinical features of AP are acute epigastric pain, nausea, vomiting, fever, and even slipping into shock (1). The severity of pancreatitis is different, grouped into MAP, MSAP, and SAP according to the Atlanta classification (2). The key differentiator among these three types is AP with or without transient organ failure or persistent organ failure. In general, 20% of patients have severe AP, and the mortality rate is as high as 20%–40%. The cause of AP varies, including gallstone obstruction in the pancreatic duct (the most common cause of AP), alcohol abuse, endoscopic retrograde cholangiopancreatography (ERCP), and an imbalanced internal environment (28). Multiple biomarkers have been reported to have significant diagnostic value in AP. The types of these biomarkers vary, including biochemical indices (such as amylase and lipase) and other novel indicators (such as indicators from the metabolome, genes, and miRNA). However, these novel indicators have not been investigated (29). In our current research, we identified two pivotal gene modules through WGCNA methods based on the transcriptional expression data of peripheral blood from AP patients and healthy controls. Genes in the two modules play important roles in the exacerbation of AP. Then, 30 genes were identified through machine-learning methods with significant diagnostic value in AP. Additionally, we selected and verified three key genes, S100A6, S100A9, and S100A12, with robust diagnostic value for both the occurrence of AP and the severity of AP. S100A6, S100A9, and S100A12 encode low-molecular weight (9,000–14,000 Da) calcium-binding proteins with highly conserved amino acid sequences. They are named S100 because they can dissolve in 100% saturated ammonium sulfate solution. Most of the proteins in the S100 family can be released to intercellular substances, regulating different phenotypes of recipient cells, including the activation and proliferation of immune cells, with the acquisition of cytokine production ability. Specifically, S100A6 promotes the proliferation and motility of cancer cells and induces the activation of fibroblasts (30, 31). In inflammation-related diseases, S100A6 induces a sensational inflammatory response by directly combining heat shock protein 70 (HSP70) with heat shock protein 90 (HSP90) (32, 33). A study reported that S100A6 is elevated during the carcinogenesis of pancreatic cancer (34). S100A9 could serve as a damage-associated molecular pattern (DAMP) to stimulate TLR4 and induce a sensational inflammatory response (35). Moreover, a recent study reported that the upregulation of S100A9 induces pancreatic injury and an AP response *via* NLRP3 activation by targeting VNN1-mediated ROS release and that loss of S100A9 decreases AP injury (36). S100A12 is highly abundant in neutrophils and has been identified as an activator of long-term inflammation *via* the RAGE pathway (37). A previous animal study reported the diagnostic value of S100A12 in AP in rats. To the best of our knowledge, this is the first study to identify AP biomarkers based on the transcriptional expression pattern of peripheral blood through WGCNA and machine-learning methods, and this is the first human study emphasizing that S100A6, S100A9, and S100A12 have diagnostic value for the severity of AP. These findings could increase the value of our research.

COVID-19, caused by SARS-CoV-2, is an epidemic disease that poses a certain threat to humans (38). SARS-CoV-2 infection was first reported in Wuhan (China) in December 2019, and it has rapidly spread around the world, causing 524 million active cases with 6 million deaths as of May 2022. A great number of reports indicate that COVID-19 infection can potentially result in AP (3, 4). Some single-center studies have confirmed the relationship between COVID-19 and AP (5, 6). Some literature reviews have suggested that there is an increased prevalence of AP in patients infected with COVID-19 and that SARS-CoV-2 might itself cause AP in some patients (7, 8). However, the pathogenesis of AP concomitance with COVID-19 infection remains unclear. In our current studies, by analyzing bulk sequencing data from the peripheral blood of COVID-19 and AP patients, neutrophil-related pathways were identified as the most significant pathways. The results emphasized the potential roles of neutrophils in COVID-19-induced AP. However, there is no report related to bacteria-induced sepsis, another common cause of sepsis. Hence, the following analysis focused on the identification of potential pathogenic subtypes of neutrophils. During the analysis procedure, a subgroup of neutrophils was identified as significantly expressed in COVID-19-infected peripheral blood. The gene expression characteristic of these neutrophils is high expression of IFN with proinflammatory phenotypes. Additionally, the differentiation of this group was significantly different from that of the other groups. In fact, some clinical and basic studies have focused on the potential role of IFN as a promoter in AP. The pathogenesis of AP induced by an imbalanced internal environment usually results from drug mistakes. Among these drugs, there are some case reports that IFN could result in AP (12). Additionally, IFN-γ has been reported to act as a promoter in a rat model of AP (13). A meta-analysis systematically reviewed the literature related to the occurrence of AP after IFN treatment (AP-IFN). After reviewing 16 studies that reported AP-IFN in a total of 23 patients, the results indicated that AP and IFN had a probable or definite causal relationship according to the Naranjo scale (14). Therefore, according to our results of pathogenic neutrophils with high expression of IFN and proinflammatory phenotypes, upregulation of this subgroup of neutrophils in patients may stimulate the immune system, leading to pancreatic damage *via* an autoimmune mechanism. Moreover, the basic mechanism of this neutrophil subgroup in the progression of AP is currently being researched and will be illustrated in our future reports.

In conclusion, we performed WGCNA of sequencing data from more than 87 human blood samples divided into a healthy group, MAP group, MSAP group, and SAP group for the first time and identified two functional gene modules associated with the severity of AP. Next, we identified and verified some pivotal genes in functional gene modules with significant diagnostic value, including S100A6, S100A9, and S100A12, through machine-learning methods and experimental validation in blood samples from AP patients. Then, through analysis of single-cell sequencing data, we investigated the specific changes in neutrophils in the peripheral blood of COVID-19 patients and identified one pathogenic neutrophil subgroup with high expression of IFN and a proinflammatory phenotype in COVID-19. Finally, we observed that the upregulation of the pathogenic neutrophil subgroup was correlated with the severity of AP in bulk sequencing data. To the best of our knowledge, this is the first study to identify gene biomarkers in peripheral blood of AP using WGCNA and to propose a potential pathogenesis of COVID-19-induced pancreatitis through the identification of a specific functional subgroup in neutrophils. These findings could facilitate clinical severity diagnosis and basic research of AP.

However, this study has some shortcomings. First, the AP high-throughput data are from a public database, and specific clinical data of enrolled patients are difficult to collect. Second, the potential mechanisms of the screened genes and functional neutrophil subtypes in AP need to be further clarified in basic research in further *in vivo* experiments.

## Data availability statement

The original contributions presented in the study are included in the article/supplementary material. Further inquiries can be directed to the corresponding authors.

## Ethics statement

This study was reviewed and approved by the ethics committee of First Affiliated Hospital of Naval Medical University. The patients/participants provided their written informed consent to participate in this study.

## Author contributions

HH and XS contributed to the conception of the study. DZ, MW, YZ and KL performed and visualized the experiment *in silico*. CX, LP, SL and XY contributed significantly to analysis and manuscript preparation. DZ, MW, WL and HY performed the data analyses and wrote the manuscript. All authors contributed to the article and approved the submitted version.

## Conflict of interest

The authors declare that the research was conducted in the absence of any commercial or financial relationships that could be construed as a potential conflict of interest.

## Publisher’s note

All claims expressed in this article are solely those of the authors and do not necessarily represent those of their affiliated organizations, or those of the publisher, the editors and the reviewers. Any product that may be evaluated in this article, or claim that may be made by its manufacturer, is not guaranteed or endorsed by the publisher.
