# Supplementary material for: Novel insight on marker genes and pathogenic peripheral neutrophil subtypes in acute pancreatitis
Source: Front Immunol. 2022 Aug 22;13:964622. doi: 10.3389/fimmu.2022.964622 (PMC9444397; doi:10.3389/fimmu.2022.964622)
Supplement: Supplementary file 3 [file Table_1.docx]

Table S1. Characteristic of enrolled patients.

| **Characteristics** | **MAP (*n*=14)** | **MSAP(*n*=8)** | **SAP (*n*=6)** |
| --- | --- | --- | --- |
| **Sex** |  |  |  |
| Female | 6 | 3 | 2 |
| Male | 8 | 5 | 4 |
| **Age, years** |  |  |  |
| Mean (SD) | 45.6 (12.7) | 40.4 (22.7) | 47.6 (17.1) |
| **Pathogenesis** |  |  |  |
| hyperlipemia | 3 | 3 | 1 |
| alcohol | 7 | 2 | 3 |
| gallstone | 4 | 3 | 2 |
