# Supplementary material for: Novel insight on marker genes and pathogenic peripheral neutrophil subtypes in acute pancreatitis
Source: Front Immunol. 2022 Aug 22;13:964622. doi: 10.3389/fimmu.2022.964622 (PMC9444397; doi:10.3389/fimmu.2022.964622)
Supplement: Supplementary file 4 [file Table_2.docx]

Table S2 Primer sequences used for quantitative polymerase chain reaction

| Gene | Cycles | Tm (℃) | Forward primer | Reverse primer |
| --- | --- | --- | --- | --- |
| S100A6 | 40 | 60 | 5- GCCTCCCTACCGCTCCAA-3 | 5- CACCTCCTGGTCCTTGTTCC-3 |
| S100A9 | 40 | 60 | 5- CGGAAACCCTGATCCGGAAA-3 | 5- CCTGGCCACCAGCATAATGA-3 |
| S100A12 | 40 | 60 | 5-CCTGGATGCTAATCAAGATGAAC-3 | 5-CTACTCTTTGTGGGTGTGGTAA-3 |
| β-actin | 40 | 60 | 5-ACCACACCTTCTACAATGAG-3 | 5-ACGACCAGAGGCATACAG-3 |
