# Supplementary material for: Novel insight on marker genes and pathogenic peripheral neutrophil subtypes in acute pancreatitis
Source: Front Immunol. 2022 Aug 22;13:964622. doi: 10.3389/fimmu.2022.964622 (PMC9444397; doi:10.3389/fimmu.2022.964622)
Supplement: Supplementary file 5 [file Table_3.docx]

Table S3. The result of DEGs in acute pancreatitis datasets.

|  | baseMean | log2FoldChange | lfcSE | stat | pvalue | padj | sig |
| --- | --- | --- | --- | --- | --- | --- | --- |
| ARG1 | 683.832 | 4.186154 | 0.358197 | 11.68673 | 1.49E-31 | 2.51E-27 | up |
| MCEMP1 | 1136.004 | 4.197886 | 0.363588 | 11.54573 | 7.76E-31 | 3.90E-27 | up |
| S100A12 | 4159.984 | 3.964154 | 0.343798 | 11.53049 | 9.26E-31 | 3.90E-27 | up |
| S100A8 | 16191.49 | 3.390589 | 0.292902 | 11.57585 | 5.46E-31 | 3.90E-27 | up |
| CIR1 | 1724.321 | 1.139007 | 0.099722 | 11.42179 | 3.25E-30 | 1.10E-26 | up |
| AIF1 | 1324.743 | 1.426006 | 0.126366 | 11.28471 | 1.56E-29 | 3.76E-26 | up |
| GTF2B | 276.916 | 1.001171 | 0.088702 | 11.28692 | 1.52E-29 | 3.76E-26 | up |
| TXN | 461.5233 | 2.023361 | 0.179747 | 11.25674 | 2.15E-29 | 4.52E-26 | up |
| ANXA3 | 2057.514 | 3.295588 | 0.29819 | 11.05196 | 2.14E-28 | 4.01E-25 | up |
| CD177 | 2100.547 | 5.498531 | 0.504697 | 10.89472 | 1.22E-27 | 2.06E-24 | up |
| NDUFA1 | 163.8528 | 1.385933 | 0.128467 | 10.78825 | 3.91E-27 | 5.49E-24 | up |
| GMFG | 1716.413 | 1.326231 | 0.123121 | 10.7718 | 4.68E-27 | 6.06E-24 | up |
| CSTA | 244.2173 | 1.756357 | 0.163824 | 10.72103 | 8.11E-27 | 9.76E-24 | up |
| ATP6V1E1 | 701.2987 | 1.028845 | 0.096557 | 10.65528 | 1.65E-26 | 1.85E-23 | up |
| ZDHHC19 | 51.14221 | 4.423162 | 0.418734 | 10.56317 | 4.42E-26 | 4.13E-23 | up |
| ARPC3 | 3365.206 | 1.211768 | 0.114708 | 10.56396 | 4.38E-26 | 4.13E-23 | up |
| CSF2RA | 1695.939 | 1.229583 | 0.116742 | 10.53249 | 6.12E-26 | 5.42E-23 | up |
| S100A9 | 44449.95 | 2.370809 | 0.228236 | 10.38752 | 2.83E-25 | 2.38E-22 | up |
| FAM20A | 81.77636 | 3.9278 | 0.380113 | 10.33323 | 4.99E-25 | 3.86E-22 | up |
| OLAH | 86.48488 | 4.793523 | 0.465109 | 10.30623 | 6.60E-25 | 4.45E-22 | up |
| CLEC4A | 39.51478 | 1.479166 | 0.143498 | 10.3079 | 6.49E-25 | 4.45E-22 | up |
| SAT1 | 6159.922 | 1.259576 | 0.122158 | 10.31105 | 6.28E-25 | 4.45E-22 | up |
| SLPI | 218.7353 | 2.672242 | 0.259405 | 10.30142 | 6.94E-25 | 4.50E-22 | up |
| SLC51A | 38.36825 | 4.751378 | 0.467377 | 10.16606 | 2.81E-24 | 1.69E-21 | up |
| H2AZ1 | 805.3642 | 1.026258 | 0.101584 | 10.10257 | 5.38E-24 | 3.02E-21 | up |
| CARD16 | 389.0366 | 1.674014 | 0.167213 | 10.01128 | 1.36E-23 | 6.74E-21 | up |
| CYSTM1 | 902.4486 | 2.909267 | 0.292564 | 9.944044 | 2.68E-23 | 1.25E-20 | up |
| CLEC4D | 562.8986 | 3.047296 | 0.306591 | 9.939291 | 2.81E-23 | 1.28E-20 | up |
| CMTM2 | 520.8055 | 1.620488 | 0.163677 | 9.900499 | 4.14E-23 | 1.79E-20 | up |
| HP | 555.5345 | 3.869788 | 0.391494 | 9.884676 | 4.85E-23 | 2.04E-20 | up |
| MTX1 | 248.014 | 1.119846 | 0.113532 | 9.863738 | 5.98E-23 | 2.40E-20 | up |
| RGS2 | 9577.624 | 1.46308 | 0.149 | 9.819348 | 9.29E-23 | 3.64E-20 | up |
| MNDA | 31919.25 | 1.086207 | 0.110676 | 9.814276 | 9.77E-23 | 3.74E-20 | up |
| CKLF | 167.3361 | 1.217384 | 0.124141 | 9.806478 | 1.06E-22 | 3.95E-20 | up |
| GPR84 | 119.4673 | 3.663821 | 0.375305 | 9.762259 | 1.63E-22 | 5.86E-20 | up |
| ATP6V1D | 330.663 | 1.05026 | 0.10804 | 9.721032 | 2.45E-22 | 8.61E-20 | up |
| PLBD1 | 6095.366 | 1.71581 | 0.176999 | 9.693895 | 3.20E-22 | 1.10E-19 | up |
| BCL2A1 | 741.8642 | 2.621659 | 0.270685 | 9.685274 | 3.48E-22 | 1.17E-19 | up |
| LINC01270 | 195.2824 | 1.719015 | 0.178344 | 9.638776 | 5.48E-22 | 1.81E-19 | up |
| HMGB2 | 1037.33 | 1.896601 | 0.19717 | 9.619116 | 6.64E-22 | 2.11E-19 | up |
| LINC00671 | 49.41869 | 2.424258 | 0.25292 | 9.58506 | 9.24E-22 | 2.78E-19 | up |
| FCER1G | 3837.5 | 1.989467 | 0.208035 | 9.563152 | 1.14E-21 | 3.32E-19 | up |
| GLT1D1 | 2512.087 | 1.359222 | 0.14306 | 9.501079 | 2.08E-21 | 5.74E-19 | up |
| MIR223HG | 3024.342 | 1.615018 | 0.170186 | 9.489703 | 2.32E-21 | 6.29E-19 | up |
| NCF2 | 15193.7 | 1.040309 | 0.110206 | 9.439663 | 3.74E-21 | 9.69E-19 | up |
| SLC22A4 | 475.1906 | 1.621563 | 0.171993 | 9.428086 | 4.18E-21 | 1.05E-18 | up |
| MS4A4A | 136.5435 | 2.579304 | 0.273673 | 9.424784 | 4.31E-21 | 1.05E-18 | up |
| IL18 | 101.6633 | 1.103674 | 0.117224 | 9.415046 | 4.73E-21 | 1.12E-18 | up |
| LINC02207 | 63.38211 | 2.640805 | 0.280637 | 9.410038 | 4.96E-21 | 1.16E-18 | up |
| GADD45A | 275.4103 | 2.77874 | 0.295648 | 9.398815 | 5.52E-21 | 1.27E-18 | up |
| BMX | 269.8712 | 2.521124 | 0.268426 | 9.392234 | 5.87E-21 | 1.34E-18 | up |
| RGL4 | 271.0912 | 2.194502 | 0.234 | 9.378229 | 6.71E-21 | 1.49E-18 | up |
| DHRS7 | 1639.099 | 1.041835 | 0.111301 | 9.360554 | 7.93E-21 | 1.74E-18 | up |
| FCGR1A | 319.3353 | 2.42778 | 0.260396 | 9.323401 | 1.13E-20 | 2.43E-18 | up |
| NDUFB3 | 115.5393 | 1.71752 | 0.184391 | 9.314554 | 1.22E-20 | 2.61E-18 | up |
| SLC26A8 | 255.1227 | 1.905129 | 0.204579 | 9.312453 | 1.25E-20 | 2.63E-18 | up |
| VNN1 | 1460.757 | 2.74707 | 0.295074 | 9.309772 | 1.28E-20 | 2.63E-18 | up |
| KCNE1 | 190.4643 | 2.164539 | 0.23261 | 9.305462 | 1.33E-20 | 2.71E-18 | up |
| XRCC4 | 66.82848 | 1.613764 | 0.173722 | 9.289348 | 1.55E-20 | 3.11E-18 | up |
| NOP10 | 361.3023 | 1.272018 | 0.136995 | 9.285116 | 1.62E-20 | 3.18E-18 | up |
| CASP4 | 4299.71 | 1.132606 | 0.121987 | 9.284642 | 1.62E-20 | 3.18E-18 | up |
| LILRA5 | 1833.695 | 2.024725 | 0.218301 | 9.274908 | 1.78E-20 | 3.44E-18 | up |
| LMNB1 | 2106.593 | 1.676904 | 0.181031 | 9.263097 | 1.99E-20 | 3.80E-18 | up |
| POMP | 318.911 | 1.019577 | 0.110105 | 9.260032 | 2.04E-20 | 3.87E-18 | up |
| PYGL | 8286.577 | 1.666882 | 0.180236 | 9.248314 | 2.28E-20 | 4.22E-18 | up |
| HNRNPH2 | 1349.373 | 1.049103 | 0.113424 | 9.249423 | 2.26E-20 | 4.22E-18 | up |
| LY96 | 211.88 | 1.74929 | 0.189287 | 9.24145 | 2.43E-20 | 4.40E-18 | up |
| ASAP1-IT2 | 47.5062 | 1.264571 | 0.136824 | 9.242334 | 2.41E-20 | 4.40E-18 | up |
| IL1R2 | 1876.839 | 2.746945 | 0.297637 | 9.229175 | 2.73E-20 | 4.86E-18 | up |
| S100A6 | 2899.206 | 1.212476 | 0.131637 | 9.210775 | 3.24E-20 | 5.57E-18 | up |
| SLC1A3 | 77.72031 | 3.617309 | 0.392788 | 9.209309 | 3.28E-20 | 5.58E-18 | up |
| NLRC4 | 1178.495 | 1.892198 | 0.205974 | 9.186589 | 4.05E-20 | 6.76E-18 | up |
| RPL11P3 | 63.50413 | 1.276345 | 0.138933 | 9.186789 | 4.05E-20 | 6.76E-18 | up |
| GRB10 | 247.6376 | 2.273043 | 0.24777 | 9.174001 | 4.56E-20 | 7.53E-18 | up |
| IFNGR1 | 3708.041 | 1.416717 | 0.154462 | 9.171968 | 4.64E-20 | 7.60E-18 | up |
| MYBPC3 | 75.67548 | 1.298188 | 0.141727 | 9.159805 | 5.20E-20 | 8.42E-18 | up |
| HK3 | 1481.775 | 1.973812 | 0.216302 | 9.125242 | 7.16E-20 | 1.13E-17 | up |
| RAB32 | 453.0808 | 1.55959 | 0.171051 | 9.117705 | 7.67E-20 | 1.20E-17 | up |
| MBOAT2 | 1065.443 | 1.373958 | 0.151163 | 9.089234 | 9.97E-20 | 1.49E-17 | up |
| GYG1 | 1991.363 | 2.449873 | 0.269895 | 9.07713 | 1.11E-19 | 1.63E-17 | up |
| ZFAS1 | 290.2777 | 1.046349 | 0.115516 | 9.058015 | 1.33E-19 | 1.90E-17 | up |
| PPARG | 15.8698 | 3.52389 | 0.389902 | 9.037888 | 1.60E-19 | 2.24E-17 | up |
| IL18RAP | 2268.897 | 2.177629 | 0.241143 | 9.030464 | 1.71E-19 | 2.38E-17 | up |
| VNN2 | 14622.67 | 1.594813 | 0.176664 | 9.027361 | 1.76E-19 | 2.43E-17 | up |
| OSCAR | 600.3553 | 1.273274 | 0.141199 | 9.017566 | 1.92E-19 | 2.61E-17 | up |
| CLIC1 | 3219.855 | 1.19822 | 0.133395 | 8.982483 | 2.65E-19 | 3.51E-17 | up |
| PCMT1 | 644.1288 | 1.229722 | 0.137236 | 8.960651 | 3.23E-19 | 4.25E-17 | up |
| BST1 | 1293.974 | 1.281084 | 0.143158 | 8.94872 | 3.60E-19 | 4.70E-17 | up |
| TNFSF10 | 3392.735 | 1.057965 | 0.118377 | 8.937241 | 3.99E-19 | 5.17E-17 | up |
| TDRD9 | 272.8483 | 2.760101 | 0.308914 | 8.934862 | 4.08E-19 | 5.24E-17 | up |
| MGST1 | 200.9016 | 1.514408 | 0.169585 | 8.930105 | 4.26E-19 | 5.39E-17 | up |
| QPCT | 2204.919 | 1.461545 | 0.163949 | 8.914617 | 4.90E-19 | 6.02E-17 | up |
| ROPN1L | 156.7118 | 1.554479 | 0.174525 | 8.906915 | 5.25E-19 | 6.40E-17 | up |
| SLC11A1 | 3787.588 | 1.463625 | 0.164848 | 8.87865 | 6.77E-19 | 7.85E-17 | up |
| LILRB3 | 3935.548 | 1.219444 | 0.137354 | 8.87811 | 6.80E-19 | 7.85E-17 | up |
| LOC102725035 | 3935.548 | 1.219444 | 0.137354 | 8.87811 | 6.80E-19 | 7.85E-17 | up |
| LOC107987425 | 3935.548 | 1.219444 | 0.137354 | 8.87811 | 6.80E-19 | 7.85E-17 | up |
| LOC107987462 | 3935.548 | 1.219444 | 0.137354 | 8.87811 | 6.80E-19 | 7.85E-17 | up |
| NCF4 | 2512.707 | 1.141291 | 0.128542 | 8.878759 | 6.76E-19 | 7.85E-17 | up |
| ARL11 | 474.2962 | 1.080309 | 0.121724 | 8.875044 | 6.99E-19 | 8.01E-17 | up |
| MMP9 | 2353.107 | 2.568543 | 0.290068 | 8.854965 | 8.37E-19 | 9.53E-17 | up |
| CR1 | 4212.49 | 1.822506 | 0.205958 | 8.84891 | 8.84E-19 | 9.92E-17 | up |
| CA4 | 284.4342 | 2.027515 | 0.22916 | 8.847593 | 8.94E-19 | 9.96E-17 | up |
| LINC00173 | 155.9574 | 1.112529 | 0.125752 | 8.846977 | 8.99E-19 | 9.96E-17 | up |
| SRPK1 | 2695.324 | 1.442852 | 0.163735 | 8.812123 | 1.23E-18 | 1.33E-16 | up |
| IFNGR2 | 3009.109 | 1.005045 | 0.114086 | 8.809573 | 1.26E-18 | 1.35E-16 | up |
| MIR4435-2HG | 162.6156 | 1.164226 | 0.132166 | 8.808832 | 1.26E-18 | 1.35E-16 | up |
| ACSL1 | 26704.42 | 1.810954 | 0.206318 | 8.777481 | 1.67E-18 | 1.73E-16 | up |
| NECAB1 | 34.98132 | 6.678983 | 0.760999 | 8.7766 | 1.68E-18 | 1.73E-16 | up |
| NMI | 1405.188 | 1.058057 | 0.12089 | 8.752247 | 2.09E-18 | 2.12E-16 | up |
| RNASE2 | 287.9393 | 2.197444 | 0.251322 | 8.743544 | 2.26E-18 | 2.27E-16 | up |
| PLIN5 | 230.0125 | 1.56553 | 0.179075 | 8.742323 | 2.28E-18 | 2.28E-16 | up |
| CLEC4E | 1779.351 | 1.822951 | 0.208551 | 8.741016 | 2.31E-18 | 2.29E-16 | up |
| RETN | 55.98558 | 3.297962 | 0.377495 | 8.736448 | 2.41E-18 | 2.37E-16 | up |
| RAB24 | 83.42637 | 1.11834 | 0.12826 | 8.71935 | 2.80E-18 | 2.68E-16 | up |
| ATP5F1E | 810.1312 | 1.09281 | 0.125327 | 8.719648 | 2.79E-18 | 2.68E-16 | up |
| TLR5 | 946.0922 | 1.961825 | 0.225237 | 8.710037 | 3.04E-18 | 2.87E-16 | up |
| MYL6 | 2227.199 | 1.32066 | 0.151798 | 8.700136 | 3.31E-18 | 3.10E-16 | up |
| UPP1 | 1057 | 1.653809 | 0.190115 | 8.69901 | 3.35E-18 | 3.12E-16 | up |
| C1RL | 1249.942 | 1.028485 | 0.118338 | 8.691107 | 3.59E-18 | 3.32E-16 | up |
| ANKRD22 | 153.1737 | 2.79247 | 0.321849 | 8.67634 | 4.09E-18 | 3.74E-16 | up |
| RPL7P18 | 48.17437 | 1.327086 | 0.153019 | 8.672677 | 4.22E-18 | 3.82E-16 | up |
| FCAR | 861.1926 | 1.946527 | 0.224505 | 8.670305 | 4.31E-18 | 3.86E-16 | up |
| CYP19A1 | 22.86013 | 6.659776 | 0.769125 | 8.658896 | 4.76E-18 | 4.22E-16 | up |
| PGD | 5544.122 | 1.513864 | 0.175088 | 8.646279 | 5.32E-18 | 4.67E-16 | up |
| METTL7B | 20.16267 | 4.293175 | 0.496961 | 8.638854 | 5.68E-18 | 4.90E-16 | up |
| LOC101928143 | 83.91811 | 1.196541 | 0.138502 | 8.639167 | 5.66E-18 | 4.90E-16 | up |
| BEST1 | 980.492 | 1.05485 | 0.122092 | 8.639771 | 5.63E-18 | 4.90E-16 | up |
| AP3B2 | 43.01709 | 3.592225 | 0.416153 | 8.631983 | 6.03E-18 | 5.16E-16 | up |
| LSMEM1 | 195.5773 | 1.138802 | 0.131957 | 8.630095 | 6.13E-18 | 5.21E-16 | up |
| SF3B6 | 229.6965 | 1.177259 | 0.136812 | 8.604925 | 7.64E-18 | 6.43E-16 | up |
| GSTO1 | 433.8242 | 1.093152 | 0.127095 | 8.601071 | 7.90E-18 | 6.62E-16 | up |
| MMP25 | 5590.083 | 1.354351 | 0.157542 | 8.596743 | 8.20E-18 | 6.84E-16 | up |
| ALPL | 5302.786 | 1.887658 | 0.219795 | 8.588279 | 8.83E-18 | 7.33E-16 | up |
| BCL6 | 10379.67 | 1.537079 | 0.179141 | 8.580292 | 9.46E-18 | 7.74E-16 | up |
| HTATIP2 | 582.7136 | 1.021255 | 0.119016 | 8.580823 | 9.42E-18 | 7.74E-16 | up |
| DYNLT1 | 710.6449 | 1.00163 | 0.116795 | 8.575946 | 9.83E-18 | 7.96E-16 | up |
| SERPINB1 | 5335.116 | 1.751043 | 0.204318 | 8.570177 | 1.03E-17 | 8.25E-16 | up |
| DOCK8-AS1 | 25.47653 | 1.19813 | 0.139818 | 8.56923 | 1.04E-17 | 8.28E-16 | up |
| ST3GAL4 | 362.2094 | 1.569692 | 0.183199 | 8.568256 | 1.05E-17 | 8.31E-16 | up |
| C19orf38 | 638.0237 | 1.227462 | 0.143304 | 8.565414 | 1.08E-17 | 8.44E-16 | up |
| PFKFB2 | 1009.429 | 2.849614 | 0.332737 | 8.564164 | 1.09E-17 | 8.49E-16 | up |
| PRDX6-AS1 | 43.34417 | 1.095231 | 0.128124 | 8.548224 | 1.25E-17 | 9.70E-16 | up |
| LIPN | 152.5976 | 2.011115 | 0.23533 | 8.545946 | 1.27E-17 | 9.81E-16 | up |
| P4HA2 | 7.755992 | 1.565457 | 0.183295 | 8.540636 | 1.33E-17 | 1.02E-15 | up |
| SRGN | 16584.28 | 1.476283 | 0.172941 | 8.536341 | 1.39E-17 | 1.06E-15 | up |
| CAMP | 118.2798 | 1.856152 | 0.218105 | 8.510367 | 1.73E-17 | 1.29E-15 | up |
| GCA | 12688.84 | 1.744792 | 0.205449 | 8.492565 | 2.02E-17 | 1.49E-15 | up |
| FGF13 | 48.84167 | 3.105021 | 0.365923 | 8.485452 | 2.15E-17 | 1.58E-15 | up |
| CASP5 | 174.3596 | 2.010009 | 0.237631 | 8.458522 | 2.71E-17 | 1.97E-15 | up |
| ECHDC3 | 54.93967 | 2.318022 | 0.274514 | 8.444098 | 3.06E-17 | 2.20E-15 | up |
| IRAK3 | 3821.804 | 1.851487 | 0.219268 | 8.443947 | 3.07E-17 | 2.20E-15 | up |
| ADM | 713.0126 | 1.530364 | 0.181265 | 8.442695 | 3.10E-17 | 2.20E-15 | up |
| C1QB | 35.80432 | 2.710588 | 0.322027 | 8.417276 | 3.85E-17 | 2.69E-15 | up |
| CHMP5 | 357.6215 | 1.090658 | 0.129574 | 8.417236 | 3.85E-17 | 2.69E-15 | up |
| LILRA6 | 1728.43 | 1.626529 | 0.193533 | 8.404391 | 4.30E-17 | 2.95E-15 | up |
| NQO2 | 864.6937 | 1.332319 | 0.15856 | 8.402594 | 4.37E-17 | 2.98E-15 | up |
| SAMSN1 | 1037.265 | 2.062004 | 0.245648 | 8.394137 | 4.69E-17 | 3.15E-15 | up |
| LOC388813 | 1037.265 | 2.062004 | 0.245648 | 8.394137 | 4.69E-17 | 3.15E-15 | up |
| CAMKK2 | 1877.923 | 1.050329 | 0.12519 | 8.389896 | 4.87E-17 | 3.24E-15 | up |
| CD58 | 478.7183 | 1.132468 | 0.135003 | 8.388488 | 4.92E-17 | 3.27E-15 | up |
| FBXL5 | 5740.972 | 1.077803 | 0.128504 | 8.387318 | 4.97E-17 | 3.29E-15 | up |
| IMPA2 | 584.8793 | 1.218611 | 0.145351 | 8.383901 | 5.12E-17 | 3.37E-15 | up |
| SLC36A1 | 526.7413 | 1.169079 | 0.139804 | 8.362274 | 6.15E-17 | 3.99E-15 | up |
| ASPH | 795.3251 | 1.852862 | 0.221752 | 8.355558 | 6.51E-17 | 4.20E-15 | up |
| SNORD89 | 50.47163 | 1.104665 | 0.132213 | 8.355174 | 6.53E-17 | 4.20E-15 | up |
| SQOR | 599.4323 | 1.070177 | 0.128091 | 8.354803 | 6.55E-17 | 4.20E-15 | up |
| SLC12A9 | 1441.596 | 1.009871 | 0.120882 | 8.354172 | 6.59E-17 | 4.20E-15 | up |
| PFKFB3 | 2041 | 2.215118 | 0.265249 | 8.351096 | 6.76E-17 | 4.30E-15 | up |
| ALPK1 | 2294.75 | 1.107004 | 0.132604 | 8.34818 | 6.93E-17 | 4.39E-15 | up |
| NSUN7 | 251.756 | 1.848223 | 0.221621 | 8.339556 | 7.46E-17 | 4.67E-15 | up |
| CSF3R | 32134.62 | 1.002214 | 0.120514 | 8.316181 | 9.08E-17 | 5.60E-15 | up |
| LAMTOR5 | 326.9642 | 1.232901 | 0.148484 | 8.303279 | 1.01E-16 | 6.14E-15 | up |
| CAPZA2 | 1732.281 | 1.182789 | 0.142683 | 8.289601 | 1.14E-16 | 6.84E-15 | up |
| FFAR2 | 2907.341 | 1.154809 | 0.139361 | 8.286445 | 1.17E-16 | 6.99E-15 | up |
| MS4A6A | 1648.661 | 1.088967 | 0.131554 | 8.277737 | 1.26E-16 | 7.47E-15 | up |
| CD63 | 1919.988 | 1.348261 | 0.162963 | 8.273399 | 1.30E-16 | 7.69E-15 | up |
| NAIP | 573.8458 | 1.89295 | 0.229009 | 8.265827 | 1.39E-16 | 8.11E-15 | up |
| APMAP | 2803.852 | 1.237919 | 0.149779 | 8.264984 | 1.40E-16 | 8.11E-15 | up |
| SYTL2 | 218.8346 | -1.09186 | 0.132104 | -8.26517 | 1.39E-16 | 8.11E-15 | down |
| IL1R1 | 535.4591 | 1.478281 | 0.178918 | 8.262342 | 1.43E-16 | 8.27E-15 | up |
| SLC37A3 | 664.5479 | 1.581497 | 0.192098 | 8.232779 | 1.83E-16 | 1.04E-14 | up |
| DGAT2 | 1853.214 | 1.293586 | 0.157304 | 8.22347 | 1.98E-16 | 1.12E-14 | up |
| ADAM9 | 423.1101 | 1.591374 | 0.193572 | 8.221079 | 2.02E-16 | 1.14E-14 | up |
| FUT7 | 143.0238 | 1.448003 | 0.176257 | 8.215282 | 2.12E-16 | 1.18E-14 | up |
| CKAP2LP1 | 21.41287 | 1.492525 | 0.18184 | 8.20789 | 2.25E-16 | 1.24E-14 | up |
| ENTPD1 | 1317.248 | 1.040734 | 0.126855 | 8.204133 | 2.32E-16 | 1.27E-14 | up |
| S100A11 | 8113.728 | 1.297704 | 0.158314 | 8.197008 | 2.46E-16 | 1.35E-14 | up |
| RNF149 | 10528.01 | 1.093204 | 0.133419 | 8.193796 | 2.53E-16 | 1.38E-14 | up |
| SELL | 35741.79 | 1.10366 | 0.134719 | 8.192284 | 2.56E-16 | 1.38E-14 | up |
| CKAP4 | 1751.015 | 1.634675 | 0.199821 | 8.180706 | 2.82E-16 | 1.51E-14 | up |
| SDCBP | 13454.33 | 1.155224 | 0.141295 | 8.175974 | 2.93E-16 | 1.56E-14 | up |
| SEMA4A | 1354.523 | 1.14261 | 0.140089 | 8.156319 | 3.45E-16 | 1.81E-14 | up |
| MCTP1 | 618.5691 | 1.183557 | 0.145347 | 8.142974 | 3.86E-16 | 2.01E-14 | up |
| P2RY13 | 5852.433 | 1.030869 | 0.126627 | 8.141018 | 3.92E-16 | 2.03E-14 | up |
| CYP1B1-AS1 | 36.85078 | 1.964818 | 0.241766 | 8.126925 | 4.40E-16 | 2.25E-14 | up |
| PGM2 | 708.5159 | 1.074764 | 0.132277 | 8.125119 | 4.47E-16 | 2.28E-14 | up |
| RAB5IF | 65.78997 | 1.112855 | 0.137013 | 8.122257 | 4.58E-16 | 2.31E-14 | up |
| SLC2A3 | 7779.477 | 1.52901 | 0.1883 | 8.120074 | 4.66E-16 | 2.34E-14 | up |
| NAIPP3 | 17.16089 | 2.425335 | 0.298729 | 8.118843 | 4.71E-16 | 2.36E-14 | up |
| MANSC1 | 1125.77 | 1.422408 | 0.175547 | 8.102706 | 5.38E-16 | 2.67E-14 | up |
| RTN3 | 7082.118 | 1.083289 | 0.133823 | 8.094941 | 5.73E-16 | 2.84E-14 | up |
| CDKN2D | 544.0042 | 1.168142 | 0.144358 | 8.091997 | 5.87E-16 | 2.90E-14 | up |
| INSC | 18.0765 | 2.113561 | 0.261262 | 8.089808 | 5.98E-16 | 2.94E-14 | up |
| ORM1 | 168.7416 | 2.334686 | 0.288611 | 8.08939 | 6.00E-16 | 2.94E-14 | up |
| DOK3 | 2787.631 | 1.159538 | 0.143361 | 8.088222 | 6.05E-16 | 2.96E-14 | up |
| HCK | 5182.98 | 1.059448 | 0.131014 | 8.086534 | 6.14E-16 | 2.99E-14 | up |
| CACNA1E | 133.4978 | 1.805052 | 0.223408 | 8.079627 | 6.50E-16 | 3.13E-14 | up |
| TMCO3 | 566.967 | 1.234232 | 0.153334 | 8.049291 | 8.33E-16 | 3.97E-14 | up |
| C3AR1 | 694.5727 | 1.599221 | 0.198783 | 8.045046 | 8.62E-16 | 4.09E-14 | up |
| AQP9 | 17612.33 | 1.347305 | 0.167524 | 8.042442 | 8.81E-16 | 4.17E-14 | up |
| FPR1 | 10644.74 | 1.177789 | 0.146504 | 8.039296 | 9.04E-16 | 4.25E-14 | up |
| CCPG1 | 936.4013 | 1.214301 | 0.151143 | 8.034102 | 9.43E-16 | 4.40E-14 | up |
| MXD3 | 192.5556 | 1.069241 | 0.13315 | 8.030362 | 9.72E-16 | 4.51E-14 | up |
| MGAM | 7492.009 | 1.697655 | 0.211599 | 8.022966 | 1.03E-15 | 4.74E-14 | up |
| ASGR2 | 158.4801 | 1.434382 | 0.178835 | 8.020711 | 1.05E-15 | 4.81E-14 | up |
| MMP8 | 635.4955 | 3.511054 | 0.43797 | 8.016658 | 1.09E-15 | 4.95E-14 | up |
| HRH2 | 2448.512 | 1.387332 | 0.173371 | 8.002097 | 1.22E-15 | 5.49E-14 | up |
| GALNT14 | 83.2818 | 2.151963 | 0.269195 | 7.994078 | 1.31E-15 | 5.80E-14 | up |
| FKBP9 | 65.46947 | 1.612529 | 0.201734 | 7.993324 | 1.31E-15 | 5.80E-14 | up |
| SIGLEC5 | 158.6872 | 1.31747 | 0.164835 | 7.992666 | 1.32E-15 | 5.81E-14 | up |
| C1QC | 12.1939 | 3.275238 | 0.409841 | 7.991482 | 1.33E-15 | 5.85E-14 | up |
| CST7 | 1845.338 | 1.938377 | 0.242573 | 7.990908 | 1.34E-15 | 5.86E-14 | up |
| FGR | 5398.896 | 1.061425 | 0.132908 | 7.986157 | 1.39E-15 | 6.04E-14 | up |
| UPB1 | 48.48255 | 1.692153 | 0.211912 | 7.985159 | 1.40E-15 | 6.08E-14 | up |
| ALOX5 | 3845.252 | 1.233413 | 0.154522 | 7.982117 | 1.44E-15 | 6.18E-14 | up |
| ST6GALNAC3 | 104.3656 | 2.052979 | 0.25736 | 7.97708 | 1.50E-15 | 6.42E-14 | up |
| MGAM2 | 286.1491 | 1.657991 | 0.207899 | 7.974966 | 1.52E-15 | 6.47E-14 | up |
| HAUS4 | 305.0059 | 1.271632 | 0.159697 | 7.962791 | 1.68E-15 | 7.10E-14 | up |
| LINC01503 | 31.68822 | 1.390553 | 0.174708 | 7.959292 | 1.73E-15 | 7.29E-14 | up |
| NAMPT | 25799.02 | 1.379738 | 0.173549 | 7.950118 | 1.86E-15 | 7.81E-14 | up |
| SLC5A9 | 23.27281 | 1.904092 | 0.239732 | 7.942595 | 1.98E-15 | 8.27E-14 | up |
| MMADHC | 827.9756 | 1.131437 | 0.142589 | 7.934977 | 2.11E-15 | 8.69E-14 | up |
| HAT1 | 239.7802 | 1.181146 | 0.148898 | 7.932558 | 2.15E-15 | 8.78E-14 | up |
| WIPI1 | 399.2315 | 1.12809 | 0.142407 | 7.921571 | 2.35E-15 | 9.57E-14 | up |
| CES1 | 191.6751 | 1.71766 | 0.217025 | 7.914586 | 2.48E-15 | 1.00E-13 | up |
| KLHL2 | 1115.653 | 1.67745 | 0.211948 | 7.914448 | 2.48E-15 | 1.00E-13 | up |
| PFKFB4 | 759.1183 | 1.025615 | 0.129605 | 7.913385 | 2.50E-15 | 1.01E-13 | up |
| FCGR2A | 12228.32 | 1.102035 | 0.139517 | 7.898916 | 2.81E-15 | 1.12E-13 | up |
| LIN7A | 1025.685 | 1.21128 | 0.153678 | 7.881916 | 3.22E-15 | 1.27E-13 | up |
| MKNK1 | 1696.14 | 1.454231 | 0.184557 | 7.879577 | 3.28E-15 | 1.29E-13 | up |
| LTB4R | 910.8834 | 1.106333 | 0.140458 | 7.876624 | 3.36E-15 | 1.31E-13 | up |
| PHTF1 | 357.9717 | 1.393811 | 0.176997 | 7.874795 | 3.41E-15 | 1.33E-13 | up |
| SERPINA1 | 18828.16 | 1.214503 | 0.154365 | 7.867756 | 3.61E-15 | 1.40E-13 | up |
| AIM2 | 257.1283 | 1.510149 | 0.19199 | 7.865784 | 3.67E-15 | 1.42E-13 | up |
| MSRB1 | 1895.176 | 1.127943 | 0.143401 | 7.865674 | 3.67E-15 | 1.42E-13 | up |
| NFIL3 | 1172.292 | 1.221863 | 0.155428 | 7.861255 | 3.80E-15 | 1.46E-13 | up |
| ITGA7 | 22.204 | 2.786275 | 0.354897 | 7.850931 | 4.13E-15 | 1.56E-13 | up |
| FCGR1B | 235.1541 | 1.764113 | 0.224955 | 7.842066 | 4.43E-15 | 1.65E-13 | up |
| METTL9 | 2866.456 | 1.449524 | 0.185063 | 7.832618 | 4.78E-15 | 1.77E-13 | up |
| PCOLCE2 | 26.20235 | 4.663651 | 0.59587 | 7.82662 | 5.01E-15 | 1.85E-13 | up |
| RALB | 3515.382 | 1.255261 | 0.160427 | 7.824483 | 5.10E-15 | 1.87E-13 | up |
| AGTRAP | 513.3308 | 1.009065 | 0.129041 | 7.819748 | 5.29E-15 | 1.94E-13 | up |
| MAPK14 | 4908.136 | 1.497544 | 0.191646 | 7.814111 | 5.54E-15 | 2.02E-13 | up |
| LOC101927974 | 21.40189 | 1.230704 | 0.157613 | 7.808413 | 5.79E-15 | 2.10E-13 | up |
| TP53I11 | 826.7282 | 1.281291 | 0.164291 | 7.798904 | 6.24E-15 | 2.25E-13 | up |
| CC2D2B | 25.3172 | 1.191994 | 0.152852 | 7.798359 | 6.27E-15 | 2.26E-13 | up |
| C4orf3 | 1677.472 | 1.041144 | 0.133519 | 7.797712 | 6.30E-15 | 2.26E-13 | up |
| IDI1 | 895.5173 | 1.896774 | 0.243334 | 7.794942 | 6.44E-15 | 2.31E-13 | up |
| MSL3 | 2208.958 | 1.131025 | 0.145203 | 7.789284 | 6.74E-15 | 2.40E-13 | up |
| SLC12A5-AS1 | 9.81124 | 2.764547 | 0.355161 | 7.783917 | 7.03E-15 | 2.49E-13 | up |
| ALDH1A2 | 35.60664 | 1.483773 | 0.190731 | 7.779399 | 7.29E-15 | 2.57E-13 | up |
| HPGD | 290.3902 | 2.790889 | 0.358926 | 7.775662 | 7.51E-15 | 2.64E-13 | up |
| NFATC2 | 550.2787 | -1.01173 | 0.130123 | -7.77515 | 7.54E-15 | 2.65E-13 | down |
| NOL3 | 22.4668 | 1.174593 | 0.151176 | 7.769723 | 7.87E-15 | 2.73E-13 | up |
| SOCS3 | 925.2585 | 2.044482 | 0.263196 | 7.767908 | 7.98E-15 | 2.77E-13 | up |
| PLA2G4A | 87.40327 | 1.019152 | 0.131233 | 7.765962 | 8.10E-15 | 2.80E-13 | up |
| PROK2 | 5355.043 | 1.732422 | 0.223312 | 7.757871 | 8.64E-15 | 2.97E-13 | up |
| SPATC1 | 9.546626 | 3.491742 | 0.450302 | 7.75423 | 8.89E-15 | 3.05E-13 | up |
| DDX10P1 | 23.59795 | 1.66149 | 0.214391 | 7.749814 | 9.20E-15 | 3.14E-13 | up |
| CCDC17 | 50.30135 | 1.068952 | 0.138284 | 7.730095 | 1.07E-14 | 3.59E-13 | up |
| NME8 | 143.527 | 1.337631 | 0.173072 | 7.728763 | 1.09E-14 | 3.62E-13 | up |
| H2BC21 | 650.2201 | 1.339907 | 0.173604 | 7.718159 | 1.18E-14 | 3.88E-13 | up |
| PLIN3 | 794.0381 | 1.121843 | 0.145428 | 7.714073 | 1.22E-14 | 3.97E-13 | up |
| GPR27 | 489.5031 | 1.224932 | 0.158842 | 7.711638 | 1.24E-14 | 4.04E-13 | up |
| IRAG1 | 1531.48 | 1.424596 | 0.184787 | 7.709394 | 1.26E-14 | 4.10E-13 | up |
| PGLYRP1 | 209.2904 | 1.592964 | 0.206694 | 7.706882 | 1.29E-14 | 4.18E-13 | up |
| H2AC6 | 2548.302 | 1.11075 | 0.144175 | 7.704203 | 1.32E-14 | 4.24E-13 | up |
| ANO10 | 282.7164 | 1.167662 | 0.151647 | 7.699847 | 1.36E-14 | 4.38E-13 | up |
| RARA-AS1 | 49.81128 | 1.241426 | 0.161425 | 7.690443 | 1.47E-14 | 4.69E-13 | up |
| MTARC1 | 797.5722 | 1.29019 | 0.167825 | 7.687702 | 1.50E-14 | 4.78E-13 | up |
| COL9A3 | 55.27518 | 1.309062 | 0.1703 | 7.6868 | 1.51E-14 | 4.80E-13 | up |
| RNU4-62P | 60.39189 | 1.130866 | 0.147126 | 7.686353 | 1.51E-14 | 4.81E-13 | up |
| DRAM1 | 353.2617 | 1.270827 | 0.165386 | 7.684022 | 1.54E-14 | 4.88E-13 | up |
| CAPG | 580.327 | 1.095288 | 0.142648 | 7.678278 | 1.61E-14 | 5.09E-13 | up |
| IL10 | 14.77558 | 2.909367 | 0.378948 | 7.677483 | 1.62E-14 | 5.11E-13 | up |
| ADGRG3 | 2272.374 | 1.456948 | 0.189831 | 7.674972 | 1.65E-14 | 5.19E-13 | up |
| LINC01506 | 58.74122 | 1.022495 | 0.133396 | 7.665086 | 1.79E-14 | 5.57E-13 | up |
| TP53I3 | 54.15983 | 1.687844 | 0.220472 | 7.655587 | 1.92E-14 | 5.96E-13 | up |
| USB1 | 1165.62 | 1.128446 | 0.147632 | 7.643628 | 2.11E-14 | 6.49E-13 | up |
| FAM151B | 53.91511 | 1.226735 | 0.160655 | 7.635851 | 2.24E-14 | 6.87E-13 | up |
| SULT1B1 | 2879.214 | 1.469663 | 0.192504 | 7.634469 | 2.27E-14 | 6.93E-13 | up |
| GATA3 | 216.0508 | -1.01409 | 0.13289 | -7.63104 | 2.33E-14 | 7.11E-13 | down |
| CD55 | 5665.05 | 1.575357 | 0.206498 | 7.628935 | 2.37E-14 | 7.21E-13 | up |
| NLRP12 | 1106.072 | 1.046456 | 0.137614 | 7.604296 | 2.86E-14 | 8.60E-13 | up |
| ZNF438 | 492.8308 | 1.379948 | 0.1819 | 7.586285 | 3.29E-14 | 9.69E-13 | up |
| IL18R1 | 991.8458 | 2.259295 | 0.297826 | 7.585949 | 3.30E-14 | 9.70E-13 | up |
| SMPDL3A | 180.4395 | 1.785219 | 0.235371 | 7.584705 | 3.33E-14 | 9.76E-13 | up |
| SYN2 | 11.76354 | 2.347361 | 0.309628 | 7.581231 | 3.42E-14 | 9.99E-13 | up |
| ANXA1 | 3099.344 | 1.414857 | 0.18663 | 7.581065 | 3.43E-14 | 9.99E-13 | up |
| ATP9A | 263.9194 | 2.41643 | 0.318947 | 7.576282 | 3.56E-14 | 1.03E-12 | up |
| LOC400499 | 370.6891 | 1.118073 | 0.147616 | 7.574176 | 3.61E-14 | 1.05E-12 | up |
| NMRAL2P | 6.459989 | 2.529979 | 0.334526 | 7.562872 | 3.94E-14 | 1.12E-12 | up |
| PNPLA1 | 65.77119 | 1.413563 | 0.186919 | 7.562445 | 3.96E-14 | 1.12E-12 | up |
| HSDL2 | 1183.875 | 1.077634 | 0.142606 | 7.556718 | 4.13E-14 | 1.17E-12 | up |
| PADI2 | 4033.339 | 1.363838 | 0.180919 | 7.538384 | 4.76E-14 | 1.33E-12 | up |
| RNU6-1003P | 6.773577 | 1.922576 | 0.255161 | 7.534768 | 4.89E-14 | 1.36E-12 | up |
| SORT1 | 1329.412 | 1.502124 | 0.199401 | 7.533165 | 4.95E-14 | 1.38E-12 | up |
| CEBPA | 338.69 | 1.001903 | 0.133046 | 7.530481 | 5.06E-14 | 1.40E-12 | up |
| DDAH2 | 133.4972 | 1.303142 | 0.173102 | 7.528185 | 5.15E-14 | 1.42E-12 | up |
| APOBEC3A | 2195.016 | 1.039534 | 0.138099 | 7.527454 | 5.17E-14 | 1.42E-12 | up |
| APOBEC3A_B | 2195.016 | 1.039534 | 0.138099 | 7.527454 | 5.17E-14 | 1.42E-12 | up |
| LOC728488 | 176.8927 | 2.062009 | 0.27398 | 7.526124 | 5.23E-14 | 1.44E-12 | up |
| VAPA | 2232.681 | 1.043486 | 0.138895 | 7.51279 | 5.79E-14 | 1.58E-12 | up |
| MSRB2 | 151.8623 | 1.149842 | 0.153087 | 7.511048 | 5.87E-14 | 1.59E-12 | up |
| PPP1R3B | 2859.005 | 1.198719 | 0.159669 | 7.507505 | 6.03E-14 | 1.63E-12 | up |
| EXOC6 | 621.611 | 1.251743 | 0.167118 | 7.490176 | 6.88E-14 | 1.82E-12 | up |
| PPP1R3D | 689.2194 | 1.218526 | 0.162775 | 7.485937 | 7.10E-14 | 1.88E-12 | up |
| TLR2 | 5833.03 | 1.166431 | 0.155917 | 7.481128 | 7.37E-14 | 1.94E-12 | up |
| NCR3LG1 | 16.2894 | -1.03032 | 0.137762 | -7.47902 | 7.49E-14 | 1.97E-12 | down |
| QSOX1 | 575.0011 | 1.12529 | 0.15056 | 7.474038 | 7.78E-14 | 2.03E-12 | up |
| NMNAT2 | 6.66891 | 2.685104 | 0.359537 | 7.468221 | 8.13E-14 | 2.11E-12 | up |
| DBI | 176.472 | 1.019228 | 0.136568 | 7.46313 | 8.45E-14 | 2.19E-12 | up |
| DHRS13 | 453.1515 | 1.278487 | 0.171367 | 7.460531 | 8.62E-14 | 2.23E-12 | up |
| TNFAIP6 | 472.7526 | 1.710959 | 0.229399 | 7.458453 | 8.75E-14 | 2.26E-12 | up |
| FLOT1 | 2315.343 | 1.20942 | 0.162215 | 7.455677 | 8.94E-14 | 2.30E-12 | up |
| GRAMD1A | 977.9474 | 1.043496 | 0.140016 | 7.452699 | 9.14E-14 | 2.34E-12 | up |
| TRIM25 | 4459.241 | 1.048456 | 0.140736 | 7.449819 | 9.35E-14 | 2.38E-12 | up |
| DYSF | 5470.955 | 1.456765 | 0.196536 | 7.412198 | 1.24E-13 | 3.07E-12 | up |
| FAM169A | 75.5963 | -1.05099 | 0.141857 | -7.40879 | 1.27E-13 | 3.14E-12 | down |
| ITGAM | 3495.813 | 1.214122 | 0.163914 | 7.407079 | 1.29E-13 | 3.18E-12 | up |
| LOC105378819 | 59.24102 | 1.165889 | 0.157545 | 7.400334 | 1.36E-13 | 3.33E-12 | up |
| CD59 | 942.8432 | 1.349856 | 0.182886 | 7.380854 | 1.57E-13 | 3.81E-12 | up |
| LIMK2 | 3081.239 | 1.103304 | 0.149493 | 7.380325 | 1.58E-13 | 3.82E-12 | up |
| ADAMTS2 | 22.73608 | 3.786598 | 0.513215 | 7.378185 | 1.60E-13 | 3.87E-12 | up |
| KLF12 | 466.4972 | -1.08358 | 0.146945 | -7.37401 | 1.66E-13 | 3.98E-12 | down |
| CARD6 | 567.7312 | 1.284098 | 0.174184 | 7.372093 | 1.68E-13 | 4.03E-12 | up |
| F5 | 1672.943 | 1.506274 | 0.204474 | 7.366576 | 1.75E-13 | 4.18E-12 | up |
| ATP6V1C1 | 1163.014 | 1.322312 | 0.179706 | 7.358196 | 1.86E-13 | 4.38E-12 | up |
| MILR1 | 319.5106 | 1.312171 | 0.178411 | 7.354777 | 1.91E-13 | 4.48E-12 | up |
| VSIG4 | 44.50127 | 2.244127 | 0.305585 | 7.343707 | 2.08E-13 | 4.85E-12 | up |
| MAFG | 292.0906 | 1.048715 | 0.143083 | 7.32944 | 2.31E-13 | 5.33E-12 | up |
| RNU7-181P | 15.42394 | 1.308597 | 0.178677 | 7.32383 | 2.41E-13 | 5.50E-12 | up |
| MAK | 350.9009 | 1.287519 | 0.175801 | 7.323746 | 2.41E-13 | 5.50E-12 | up |
| CCNJL | 805.7488 | 1.167049 | 0.159393 | 7.321845 | 2.45E-13 | 5.56E-12 | up |
| MCTP2 | 2530.138 | 1.276612 | 0.174552 | 7.313635 | 2.60E-13 | 5.88E-12 | up |
| SLED1 | 19.56999 | 1.713003 | 0.234375 | 7.308805 | 2.70E-13 | 6.05E-12 | up |
| SH3GLB1 | 3447.531 | 1.14095 | 0.156186 | 7.305072 | 2.77E-13 | 6.20E-12 | up |
| LINC02649 | 74.34741 | 1.36389 | 0.186877 | 7.298322 | 2.91E-13 | 6.47E-12 | up |
| BAZ1A | 4017.048 | 1.035631 | 0.141945 | 7.295995 | 2.96E-13 | 6.58E-12 | up |
| RBP7 | 148.0583 | 1.003459 | 0.13775 | 7.284635 | 3.23E-13 | 7.13E-12 | up |
| PLP2 | 1494.762 | 1.158924 | 0.159157 | 7.281634 | 3.30E-13 | 7.25E-12 | up |
| UBXN2B | 2254.636 | 1.003266 | 0.137793 | 7.280971 | 3.31E-13 | 7.28E-12 | up |
| NECTIN2 | 94.757 | 2.208656 | 0.303574 | 7.275504 | 3.45E-13 | 7.55E-12 | up |
| NSMCE1-DT | 15.49008 | 1.275877 | 0.175383 | 7.274819 | 3.47E-13 | 7.58E-12 | up |
| PTCH1 | 111.8398 | -1.04747 | 0.1441 | -7.26906 | 3.62E-13 | 7.88E-12 | down |
| IL4R | 2840.473 | 1.20291 | 0.165788 | 7.255695 | 4.00E-13 | 8.67E-12 | up |
| CYYR1 | 22.45001 | 2.609306 | 0.359933 | 7.249428 | 4.19E-13 | 9.04E-12 | up |
| SLA | 6012.192 | 1.220237 | 0.168401 | 7.246032 | 4.29E-13 | 9.26E-12 | up |
| UBE2J1 | 2856.377 | 1.218096 | 0.168222 | 7.240997 | 4.45E-13 | 9.53E-12 | up |
| GPAT3 | 1116.725 | 1.051112 | 0.145248 | 7.236662 | 4.60E-13 | 9.80E-12 | up |
| PGS1 | 1163.43 | 1.204964 | 0.166775 | 7.225104 | 5.01E-13 | 1.06E-11 | up |
| FKBP5 | 4606.598 | 1.849121 | 0.256181 | 7.218015 | 5.28E-13 | 1.11E-11 | up |
| RN7SL600P | 92.55365 | 1.016419 | 0.140862 | 7.215733 | 5.36E-13 | 1.13E-11 | up |
| SH3PXD2B | 33.0014 | 2.124291 | 0.295226 | 7.195464 | 6.22E-13 | 1.29E-11 | up |
| SLC8A1 | 733.2909 | 1.037791 | 0.144581 | 7.177921 | 7.08E-13 | 1.45E-11 | up |
| LOC105375924 | 9.79253 | 2.23772 | 0.311758 | 7.177739 | 7.09E-13 | 1.45E-11 | up |
| TRAV24 | 10.91026 | -1.35731 | 0.189304 | -7.16999 | 7.50E-13 | 1.53E-11 | down |
| SEMA6B | 19.35264 | 2.598715 | 0.362621 | 7.166479 | 7.70E-13 | 1.56E-11 | up |
| S100P | 340.2385 | 1.829439 | 0.255283 | 7.166326 | 7.70E-13 | 1.56E-11 | up |
| BASP1-AS1 | 19.19295 | 1.80755 | 0.252287 | 7.164655 | 7.80E-13 | 1.58E-11 | up |
| AMPH | 9.525084 | 3.170869 | 0.443646 | 7.147296 | 8.85E-13 | 1.77E-11 | up |
| MAP2K6 | 690.0188 | 1.333506 | 0.186591 | 7.146661 | 8.89E-13 | 1.78E-11 | up |
| SLC4A4 | 19.51562 | -1.09275 | 0.152915 | -7.14614 | 8.93E-13 | 1.78E-11 | down |
| LINC00937 | 106.4078 | 1.455084 | 0.203711 | 7.142891 | 9.14E-13 | 1.82E-11 | up |
| C3orf86 | 201.8492 | 1.710248 | 0.240303 | 7.11704 | 1.10E-12 | 2.16E-11 | up |
| CEACAM4 | 579.9932 | 1.166549 | 0.164096 | 7.108961 | 1.17E-12 | 2.29E-11 | up |
| LRG1 | 1333.186 | 1.452866 | 0.204531 | 7.103393 | 1.22E-12 | 2.36E-11 | up |
| KCNJ15 | 4442.621 | 1.114095 | 0.156982 | 7.096955 | 1.28E-12 | 2.46E-11 | up |
| LRRN1 | 96.84291 | 1.830222 | 0.257898 | 7.09668 | 1.28E-12 | 2.46E-11 | up |
| PADI4 | 1616.09 | 1.261699 | 0.177929 | 7.091034 | 1.33E-12 | 2.56E-11 | up |
| CEBPD | 910.6775 | 1.225916 | 0.173022 | 7.085305 | 1.39E-12 | 2.65E-11 | up |
| LOC101927851 | 65.07642 | 1.263966 | 0.178453 | 7.082904 | 1.41E-12 | 2.69E-11 | up |
| IKZF2 | 186.5897 | -1.1516 | 0.162702 | -7.07797 | 1.46E-12 | 2.77E-11 | down |
| NR3C2 | 77.08239 | -1.05861 | 0.149738 | -7.06973 | 1.55E-12 | 2.92E-11 | down |
| TNFSF13B | 2391.354 | 1.327479 | 0.187826 | 7.067584 | 1.58E-12 | 2.96E-11 | up |
| CATIP-AS1 | 10.35392 | 1.49258 | 0.21142 | 7.059782 | 1.67E-12 | 3.11E-11 | up |
| IRAG1-AS1 | 12.96942 | 2.004777 | 0.284205 | 7.053993 | 1.74E-12 | 3.22E-11 | up |
| SLC49A4 | 242.9474 | 1.011702 | 0.143865 | 7.032324 | 2.03E-12 | 3.70E-11 | up |
| KREMEN1 | 834.3915 | 1.456433 | 0.207391 | 7.022635 | 2.18E-12 | 3.94E-11 | up |
| F12 | 11.11791 | 1.359525 | 0.19369 | 7.019082 | 2.23E-12 | 4.04E-11 | up |
| OMG | 27.41705 | 1.437005 | 0.204923 | 7.012408 | 2.34E-12 | 4.21E-11 | up |
| LOC101927057 | 27.41705 | 1.437005 | 0.204923 | 7.012408 | 2.34E-12 | 4.21E-11 | up |
| AOAH-IT1 | 12.81581 | 1.607752 | 0.229281 | 7.012131 | 2.35E-12 | 4.21E-11 | up |
| RAB31 | 3445.306 | 1.042168 | 0.148645 | 7.011127 | 2.36E-12 | 4.23E-11 | up |
| G0S2 | 19.81985 | 1.888939 | 0.26986 | 6.99971 | 2.56E-12 | 4.56E-11 | up |
| AGTPBP1 | 2099.071 | 1.050299 | 0.15031 | 6.987537 | 2.80E-12 | 4.91E-11 | up |
| CLEC5A | 186.9637 | 1.742591 | 0.249561 | 6.982611 | 2.90E-12 | 5.08E-11 | up |
| DUSP1 | 2316.863 | 1.22249 | 0.175101 | 6.981609 | 2.92E-12 | 5.11E-11 | up |
| H2BC5 | 138.4294 | 1.397727 | 0.20046 | 6.972591 | 3.11E-12 | 5.42E-11 | up |
| CD82 | 777.0381 | 1.010516 | 0.144957 | 6.971138 | 3.14E-12 | 5.46E-11 | up |
| TIMP1 | 544.5976 | 1.032672 | 0.148152 | 6.970339 | 3.16E-12 | 5.48E-11 | up |
| TNFAIP8L3 | 9.707822 | 4.965242 | 0.712495 | 6.968807 | 3.20E-12 | 5.54E-11 | up |
| CDA | 741.1192 | 1.108561 | 0.159109 | 6.967293 | 3.23E-12 | 5.59E-11 | up |
| LYVE1 | 29.36611 | 1.306629 | 0.187598 | 6.965067 | 3.28E-12 | 5.66E-11 | up |
| LOC100652833 | 10.72049 | 2.240069 | 0.32175 | 6.962146 | 3.35E-12 | 5.77E-11 | up |
| FLOT2 | 4449.763 | 1.141738 | 0.164035 | 6.960342 | 3.39E-12 | 5.84E-11 | up |
| TPST1 | 165.3796 | 1.781767 | 0.256326 | 6.951184 | 3.62E-12 | 6.19E-11 | up |
| GRIP1 | 5.123716 | -1.26831 | 0.182546 | -6.9479 | 3.71E-12 | 6.33E-11 | down |
| RN7SL473P | 81.45151 | 1.016475 | 0.146354 | 6.945302 | 3.78E-12 | 6.43E-11 | up |
| EFCAB2 | 104.5456 | 1.430632 | 0.20601 | 6.944471 | 3.80E-12 | 6.45E-11 | up |
| TGFA | 604.1897 | 1.30238 | 0.18756 | 6.943791 | 3.82E-12 | 6.47E-11 | up |
| LINC01093 | 10.94876 | 3.310611 | 0.477069 | 6.939478 | 3.94E-12 | 6.65E-11 | up |
| IL1RN | 1566.582 | 1.107472 | 0.159698 | 6.934809 | 4.07E-12 | 6.84E-11 | up |
| MYO10 | 38.46591 | 2.055136 | 0.296403 | 6.933588 | 4.10E-12 | 6.90E-11 | up |
| FAM157A | 233.8483 | 1.139169 | 0.164524 | 6.924043 | 4.39E-12 | 7.36E-11 | up |
| SLC9A7P1 | 51.71201 | 1.006687 | 0.145508 | 6.918416 | 4.57E-12 | 7.60E-11 | up |
| PLIN4 | 168.6712 | 1.22718 | 0.177675 | 6.906884 | 4.95E-12 | 8.20E-11 | up |
| TRPM2 | 123.7413 | 1.216989 | 0.176391 | 6.899387 | 5.22E-12 | 8.61E-11 | up |
| RNU7-45P | 14.63092 | 1.153944 | 0.167449 | 6.891316 | 5.53E-12 | 9.02E-11 | up |
| DAAM2 | 191.5065 | 2.72718 | 0.395879 | 6.888927 | 5.62E-12 | 9.16E-11 | up |
| PHC2 | 2465.587 | 1.123878 | 0.163639 | 6.868029 | 6.51E-12 | 1.05E-10 | up |
| GPR174 | 288.2467 | -1.05793 | 0.154197 | -6.86093 | 6.84E-12 | 1.10E-10 | down |
| NFE2 | 2401.558 | 1.071575 | 0.156263 | 6.857496 | 7.01E-12 | 1.12E-10 | up |
| CNIH4 | 428.1584 | 1.075362 | 0.156989 | 6.849938 | 7.39E-12 | 1.17E-10 | up |
| ZNF608 | 82.79371 | 1.564647 | 0.228637 | 6.843379 | 7.73E-12 | 1.22E-10 | up |
| SCN1B | 50.889 | 1.187426 | 0.173676 | 6.837026 | 8.09E-12 | 1.27E-10 | up |
| SIPA1L2 | 553.127 | 1.475588 | 0.216546 | 6.814194 | 9.48E-12 | 1.46E-10 | up |
| STXBP2 | 841.0201 | 1.02426 | 0.150436 | 6.808611 | 9.85E-12 | 1.51E-10 | up |
| GPER1 | 12.28195 | 2.133787 | 0.313889 | 6.797904 | 1.06E-11 | 1.61E-10 | up |
| ADAM22 | 29.47555 | -1.00925 | 0.148503 | -6.79619 | 1.07E-11 | 1.63E-10 | down |
| KIF1B | 1167.664 | 1.212919 | 0.178559 | 6.792828 | 1.10E-11 | 1.67E-10 | up |
| OAT | 550.6832 | 1.09193 | 0.160777 | 6.791602 | 1.11E-11 | 1.68E-10 | up |
| ZNF415P1 | 7.48527 | 1.355474 | 0.199693 | 6.787778 | 1.14E-11 | 1.72E-10 | up |
| IFITM2 | 10923.1 | 1.125148 | 0.165805 | 6.78597 | 1.15E-11 | 1.74E-10 | up |
| PSTPIP2 | 1186.642 | 1.37119 | 0.202727 | 6.763723 | 1.34E-11 | 2.00E-10 | up |
| TNNI2 | 32.54108 | 1.052476 | 0.155746 | 6.757654 | 1.40E-11 | 2.09E-10 | up |
| TSPO | 360.9259 | 1.301328 | 0.192777 | 6.750446 | 1.47E-11 | 2.19E-10 | up |
| PTEN | 5919.685 | 1.027776 | 0.152414 | 6.743303 | 1.55E-11 | 2.28E-10 | up |
| RAB30 | 166.2652 | -1.01157 | 0.150016 | -6.74309 | 1.55E-11 | 2.28E-10 | down |
| ORM2 | 10.75233 | 2.356735 | 0.349684 | 6.739608 | 1.59E-11 | 2.34E-10 | up |
| GAS7 | 2229.81 | 1.213248 | 0.180268 | 6.730228 | 1.69E-11 | 2.48E-10 | up |
| PLSCR1 | 1715.502 | 1.526545 | 0.226857 | 6.729117 | 1.71E-11 | 2.50E-10 | up |
| KCNMA1 | 33.78213 | 3.023095 | 0.449705 | 6.722394 | 1.79E-11 | 2.61E-10 | up |
| LINC00639 | 5.227513 | 2.051317 | 0.305406 | 6.716682 | 1.86E-11 | 2.71E-10 | up |
| STK3 | 172.7362 | 1.170261 | 0.174253 | 6.715886 | 1.87E-11 | 2.72E-10 | up |
| RPS2P14 | 7.847169 | 1.485022 | 0.221145 | 6.715153 | 1.88E-11 | 2.73E-10 | up |
| RN7SL105P | 9.297446 | 1.135513 | 0.169217 | 6.710411 | 1.94E-11 | 2.81E-10 | up |
| ENTPD7 | 112.942 | 2.181582 | 0.325559 | 6.70103 | 2.07E-11 | 2.96E-10 | up |
| BASP1 | 8493.614 | 1.351469 | 0.20168 | 6.701056 | 2.07E-11 | 2.96E-10 | up |
| PDCD10 | 289.6692 | 1.081619 | 0.161547 | 6.695397 | 2.15E-11 | 3.07E-10 | up |
| TGFBR3 | 282.3346 | -1.04692 | 0.156648 | -6.68326 | 2.34E-11 | 3.31E-10 | down |
| ALOX5AP | 3557.091 | 1.381314 | 0.206941 | 6.674904 | 2.47E-11 | 3.49E-10 | up |
| NR2E1 | 4.225533 | 3.933992 | 0.589652 | 6.671721 | 2.53E-11 | 3.55E-10 | up |
| PLAU | 16.67503 | 1.310635 | 0.196533 | 6.668794 | 2.58E-11 | 3.61E-10 | up |
| RORA | 739.371 | -1.02238 | 0.153326 | -6.66804 | 2.59E-11 | 3.62E-10 | down |
| LDHA | 2888.521 | 1.233951 | 0.185392 | 6.655918 | 2.82E-11 | 3.89E-10 | up |
| IL1B | 939.4304 | 1.09725 | 0.164898 | 6.654114 | 2.85E-11 | 3.94E-10 | up |
| CEBPB | 2200.453 | 1.218288 | 0.183234 | 6.648806 | 2.95E-11 | 4.07E-10 | up |
| LILRB4 | 257.5599 | 1.048128 | 0.157869 | 6.639213 | 3.15E-11 | 4.32E-10 | up |
| UGCG | 988.3456 | 1.661696 | 0.251042 | 6.619186 | 3.61E-11 | 4.89E-10 | up |
| RNU6-176P | 12.04674 | 1.040663 | 0.157575 | 6.604224 | 4.00E-11 | 5.34E-10 | up |
| ARHGEF40 | 928.24 | 1.022003 | 0.154963 | 6.595123 | 4.25E-11 | 5.65E-10 | up |
| PLB1 | 458.7318 | 1.382105 | 0.20967 | 6.591796 | 4.35E-11 | 5.76E-10 | up |
| NOXRED1 | 18.27649 | 1.041852 | 0.158097 | 6.58996 | 4.40E-11 | 5.83E-10 | up |
| SERPINB10 | 23.51634 | 1.902246 | 0.288687 | 6.589299 | 4.42E-11 | 5.85E-10 | up |
| OSM | 37.95454 | 1.641915 | 0.249324 | 6.585465 | 4.53E-11 | 5.99E-10 | up |
| KCNJ2 | 1981.851 | 1.007333 | 0.153206 | 6.575015 | 4.86E-11 | 6.39E-10 | up |
| TUBB4A | 8.877502 | -1.06266 | 0.161623 | -6.57491 | 4.87E-11 | 6.39E-10 | down |
| RNU6-917P | 6.803045 | 1.435788 | 0.220314 | 6.517013 | 7.17E-11 | 9.01E-10 | up |
| SNX3 | 1982.972 | 1.159147 | 0.178178 | 6.505552 | 7.74E-11 | 9.59E-10 | up |
| KLHL29 | 6.689785 | -1.3641 | 0.209718 | -6.50446 | 7.80E-11 | 9.64E-10 | down |
| FAM169B | 10.99876 | 1.341779 | 0.20642 | 6.500244 | 8.02E-11 | 9.90E-10 | up |
| SCARNA20 | 8.609869 | 1.134963 | 0.174657 | 6.498226 | 8.13E-11 | 1.00E-09 | up |
| B4GALT5 | 2521.231 | 1.064567 | 0.163835 | 6.497812 | 8.15E-11 | 1.00E-09 | up |
| TLR4 | 5050.218 | 1.100452 | 0.169606 | 6.488274 | 8.68E-11 | 1.07E-09 | up |
| CEP295NL | 16.60298 | 1.003338 | 0.154661 | 6.487347 | 8.74E-11 | 1.07E-09 | up |
| CSGALNACT2 | 1670.267 | 1.159275 | 0.178707 | 6.487001 | 8.76E-11 | 1.07E-09 | up |
| SCARNA7 | 3.36276 | 1.608116 | 0.247917 | 6.486513 | 8.78E-11 | 1.07E-09 | up |
| PIK3CD-AS1 | 22.99189 | 1.155148 | 0.178237 | 6.480955 | 9.11E-11 | 1.11E-09 | up |
| BTNL8 | 353.0205 | 1.092025 | 0.168522 | 6.480007 | 9.17E-11 | 1.12E-09 | up |
| FGD4 | 842.0121 | 1.224125 | 0.188968 | 6.477936 | 9.30E-11 | 1.13E-09 | up |
| INPP4B | 428.9776 | -1.01341 | 0.15648 | -6.47628 | 9.40E-11 | 1.14E-09 | down |
| MROH6 | 36.2407 | 1.336634 | 0.206553 | 6.471134 | 9.73E-11 | 1.18E-09 | up |
| PAQR6 | 34.05329 | 1.244921 | 0.192443 | 6.469022 | 9.86E-11 | 1.19E-09 | up |
| RPL21P123 | 30.80375 | 1.051844 | 0.162846 | 6.459122 | 1.05E-10 | 1.26E-09 | up |
| RNASE1 | 8.610957 | 2.493226 | 0.386114 | 6.457229 | 1.07E-10 | 1.28E-09 | up |
| TRIM9 | 35.72484 | 1.309824 | 0.203292 | 6.44308 | 1.17E-10 | 1.39E-09 | up |
| POR | 454.8791 | 1.108141 | 0.172112 | 6.438476 | 1.21E-10 | 1.43E-09 | up |
| PELATON | 749.5397 | 1.031432 | 0.160303 | 6.434287 | 1.24E-10 | 1.47E-09 | up |
| WSB1 | 3511.21 | 1.005391 | 0.156458 | 6.425936 | 1.31E-10 | 1.54E-09 | up |
| CACNA2D2 | 39.41544 | -1.15255 | 0.179624 | -6.41647 | 1.39E-10 | 1.63E-09 | down |
| DPY19L3 | 603.5075 | 1.134864 | 0.176909 | 6.414977 | 1.41E-10 | 1.64E-09 | up |
| ASGR1 | 43.97282 | 1.009458 | 0.157358 | 6.415031 | 1.41E-10 | 1.64E-09 | up |
| TRIQK | 303.1528 | 1.109644 | 0.173115 | 6.409868 | 1.46E-10 | 1.69E-09 | up |
| LINC01907 | 8.088449 | 1.813465 | 0.283095 | 6.405864 | 1.50E-10 | 1.73E-09 | up |
| TMEM88 | 26.86544 | 1.105431 | 0.172579 | 6.405375 | 1.50E-10 | 1.74E-09 | up |
| GCNT4 | 120.1301 | -1.10719 | 0.172994 | -6.40012 | 1.55E-10 | 1.79E-09 | down |
| TMLHE-AS1 | 3.390804 | 1.764803 | 0.27576 | 6.399767 | 1.56E-10 | 1.79E-09 | up |
| LOC101927830 | 3.390804 | 1.764803 | 0.27576 | 6.399767 | 1.56E-10 | 1.79E-09 | up |
| EXPH5 | 13.1187 | -1.05339 | 0.164868 | -6.38927 | 1.67E-10 | 1.90E-09 | down |
| SOCS3-DT | 6.788228 | 2.191125 | 0.343961 | 6.370279 | 1.89E-10 | 2.14E-09 | up |
| KIF3C | 145.8691 | 1.081146 | 0.169882 | 6.364094 | 1.96E-10 | 2.22E-09 | up |
| SDHAF3 | 90.78712 | 1.39209 | 0.219064 | 6.354715 | 2.09E-10 | 2.34E-09 | up |
| PRKG2 | 6.843499 | -1.33465 | 0.210248 | -6.34797 | 2.18E-10 | 2.44E-09 | down |
| ANXA9 | 12.74046 | 1.317545 | 0.207576 | 6.347273 | 2.19E-10 | 2.45E-09 | up |
| HPD | 7.733988 | 2.688312 | 0.423766 | 6.343859 | 2.24E-10 | 2.49E-09 | up |
| MERTK | 81.15595 | 1.517921 | 0.239369 | 6.341348 | 2.28E-10 | 2.53E-09 | up |
| HTRA1 | 16.96771 | 1.679263 | 0.264937 | 6.338345 | 2.32E-10 | 2.57E-09 | up |
| OPLAH | 36.81502 | 1.737968 | 0.274583 | 6.329471 | 2.46E-10 | 2.70E-09 | up |
| KL | 25.41229 | 1.847188 | 0.291902 | 6.328106 | 2.48E-10 | 2.72E-09 | up |
| DACH1 | 96.40557 | 1.609858 | 0.254744 | 6.319524 | 2.62E-10 | 2.86E-09 | up |
| LINC00211 | 94.91819 | 1.154375 | 0.182675 | 6.319271 | 2.63E-10 | 2.87E-09 | up |
| ADAMTS3 | 10.22082 | 5.409792 | 0.856185 | 6.318488 | 2.64E-10 | 2.88E-09 | up |
| AGFG1 | 1584.915 | 1.248696 | 0.197856 | 6.311125 | 2.77E-10 | 3.01E-09 | up |
| RFX2 | 299.5248 | 1.010097 | 0.160298 | 6.30139 | 2.95E-10 | 3.18E-09 | up |
| LINC02217 | 3.179942 | 2.62381 | 0.416674 | 6.29703 | 3.03E-10 | 3.26E-09 | up |
| RSPH9 | 9.704747 | 1.487203 | 0.236213 | 6.296025 | 3.05E-10 | 3.27E-09 | up |
| ACER3 | 807.9067 | 1.204991 | 0.191598 | 6.289168 | 3.19E-10 | 3.41E-09 | up |
| CYP1B1 | 1176.708 | 1.481928 | 0.23573 | 6.286557 | 3.25E-10 | 3.46E-09 | up |
| CD163 | 911.0424 | 1.165884 | 0.185486 | 6.285556 | 3.27E-10 | 3.48E-09 | up |
| KLF14 | 3.268254 | 4.505502 | 0.71728 | 6.28137 | 3.36E-10 | 3.58E-09 | up |
| B3GNT5 | 398.8926 | 1.296223 | 0.206821 | 6.267373 | 3.67E-10 | 3.86E-09 | up |
| WDFY3-AS2 | 19.75107 | 1.37047 | 0.219005 | 6.257705 | 3.91E-10 | 4.08E-09 | up |
| HSPA1A | 1558.934 | 1.056177 | 0.169239 | 6.240757 | 4.35E-10 | 4.51E-09 | up |
| ANKRD34B | 70.50267 | 2.069863 | 0.331709 | 6.239997 | 4.38E-10 | 4.52E-09 | up |
| LINC01801 | 6.559143 | -1.05193 | 0.168607 | -6.23892 | 4.41E-10 | 4.55E-09 | down |
| CRISP3 | 133.7075 | 1.997275 | 0.320398 | 6.233727 | 4.55E-10 | 4.69E-09 | up |
| TMTC1 | 265.3745 | 2.052496 | 0.329291 | 6.233071 | 4.57E-10 | 4.71E-09 | up |
| H4C4 | 10.03001 | 1.515474 | 0.243755 | 6.217191 | 5.06E-10 | 5.14E-09 | up |
| RNU6-226P | 16.10898 | 1.392478 | 0.224239 | 6.209796 | 5.31E-10 | 5.35E-09 | up |
| DPY19L2P2 | 14.5051 | -1.01191 | 0.163552 | -6.18711 | 6.13E-10 | 6.12E-09 | down |
| ABHD12B | 2.242266 | 2.52036 | 0.40748 | 6.185228 | 6.20E-10 | 6.19E-09 | up |
| SPINK8 | 8.554189 | 1.553177 | 0.251371 | 6.178824 | 6.46E-10 | 6.42E-09 | up |
| H4C8 | 111.8051 | 1.021264 | 0.165488 | 6.171223 | 6.78E-10 | 6.71E-09 | up |
| TBC1D8 | 384.6325 | 1.271426 | 0.206626 | 6.15327 | 7.59E-10 | 7.43E-09 | up |
| RPL7P24 | 3.238349 | 1.687668 | 0.274953 | 6.138018 | 8.36E-10 | 8.11E-09 | up |
| SHOX2 | 4.843637 | 2.823829 | 0.46018 | 6.136354 | 8.44E-10 | 8.19E-09 | up |
| DSC1 | 17.08185 | -1.51262 | 0.2469 | -6.12647 | 8.99E-10 | 8.62E-09 | down |
| LINC00862 | 31.33617 | 1.020447 | 0.166843 | 6.1162 | 9.58E-10 | 9.16E-09 | up |
| RN7SKP16 | 32.91905 | 1.250217 | 0.204659 | 6.108769 | 1.00E-09 | 9.54E-09 | up |
| GPR141 | 136.8453 | 1.137326 | 0.186525 | 6.097458 | 1.08E-09 | 1.02E-08 | up |
| GPR160 | 448.559 | 1.290301 | 0.2117 | 6.094958 | 1.09E-09 | 1.03E-08 | up |
| CTSL | 113.4999 | 1.018036 | 0.167178 | 6.089537 | 1.13E-09 | 1.06E-08 | up |
| FABP5 | 11.9418 | 1.019964 | 0.167558 | 6.087236 | 1.15E-09 | 1.07E-08 | up |
| TENM1 | 199.6051 | 1.676642 | 0.275943 | 6.076051 | 1.23E-09 | 1.14E-08 | up |
| OSBPL1A | 196.9355 | 1.175817 | 0.193525 | 6.07578 | 1.23E-09 | 1.14E-08 | up |
| FGF13-AS1 | 2.979798 | 3.956329 | 0.651195 | 6.075488 | 1.24E-09 | 1.14E-08 | up |
| LOXHD1 | 55.15005 | 1.19188 | 0.197111 | 6.046753 | 1.48E-09 | 1.34E-08 | up |
| PDSS1 | 61.43968 | 1.340722 | 0.221905 | 6.041874 | 1.52E-09 | 1.37E-08 | up |
| C1QA | 32.7934 | 1.642384 | 0.271921 | 6.039931 | 1.54E-09 | 1.39E-08 | up |
| TMEM119 | 12.91642 | 1.328269 | 0.220214 | 6.03173 | 1.62E-09 | 1.45E-08 | up |
| SHROOM4 | 9.530761 | 1.602686 | 0.265986 | 6.025448 | 1.69E-09 | 1.50E-08 | up |
| FZD5 | 16.59529 | 1.144317 | 0.19007 | 6.020509 | 1.74E-09 | 1.54E-08 | up |
| RPSAP36 | 5.727658 | 1.167544 | 0.194034 | 6.017213 | 1.77E-09 | 1.57E-08 | up |
| TCN2 | 65.95615 | 1.132218 | 0.188472 | 6.007368 | 1.89E-09 | 1.66E-08 | up |
| CEACAM1 | 970.7789 | 1.583637 | 0.263943 | 5.999922 | 1.97E-09 | 1.72E-08 | up |
| UBTD1 | 48.41059 | 1.010321 | 0.168445 | 5.997912 | 2.00E-09 | 1.74E-08 | up |
| SERPINB2 | 27.61117 | 1.335591 | 0.222711 | 5.996976 | 2.01E-09 | 1.75E-08 | up |
| LINC01271 | 2.779112 | 2.266698 | 0.378148 | 5.994202 | 2.04E-09 | 1.78E-08 | up |
| KANK1 | 58.82247 | -1.04077 | 0.173739 | -5.99041 | 2.09E-09 | 1.81E-08 | down |
| WDFY3 | 2146.174 | 1.028365 | 0.171797 | 5.98595 | 2.15E-09 | 1.85E-08 | up |
| EMILIN2 | 1211.747 | 1.06029 | 0.177389 | 5.977198 | 2.27E-09 | 1.95E-08 | up |
| SLC22A14 | 2.616476 | 1.93978 | 0.32477 | 5.972777 | 2.33E-09 | 2.00E-08 | up |
| DDIAS | 107.0273 | 1.113368 | 0.187038 | 5.952619 | 2.64E-09 | 2.22E-08 | up |
| PRDM5 | 150.3365 | 1.493218 | 0.250874 | 5.952056 | 2.65E-09 | 2.23E-08 | up |
| HGF | 191.1629 | 1.528378 | 0.256793 | 5.951783 | 2.65E-09 | 2.23E-08 | up |
| DUSP13 | 4.834121 | 2.378254 | 0.401044 | 5.930162 | 3.03E-09 | 2.52E-08 | up |
| DUSP8 | 12.87444 | -1.00236 | 0.169097 | -5.92771 | 3.07E-09 | 2.56E-08 | down |
| SLC8A1-AS1 | 2.346154 | 1.884095 | 0.318013 | 5.924592 | 3.13E-09 | 2.60E-08 | up |
| GRINA | 1083.995 | 1.053806 | 0.177959 | 5.921632 | 3.19E-09 | 2.65E-08 | up |
| LOC105370969 | 6.107704 | 1.55256 | 0.262472 | 5.915137 | 3.32E-09 | 2.75E-08 | up |
| GGH | 30.48684 | 1.157832 | 0.195744 | 5.915016 | 3.32E-09 | 2.75E-08 | up |
| GLB1L2 | 10.22052 | -1.5396 | 0.260759 | -5.90429 | 3.54E-09 | 2.92E-08 | down |
| FOLR3 | 215.1625 | 1.779508 | 0.301537 | 5.901457 | 3.60E-09 | 2.97E-08 | up |
| RPSAP22 | 4.936325 | 1.634532 | 0.277382 | 5.892713 | 3.80E-09 | 3.10E-08 | up |
| LOC100996442 | 5.116756 | 1.903596 | 0.323216 | 5.889551 | 3.87E-09 | 3.16E-08 | up |
| MTND6P11 | 6.646177 | 1.664502 | 0.28296 | 5.88247 | 4.04E-09 | 3.29E-08 | up |
| NUDT19P5 | 5.666161 | 1.287014 | 0.219416 | 5.865641 | 4.47E-09 | 3.60E-08 | up |
| DLC1 | 19.44892 | 2.022302 | 0.346197 | 5.841468 | 5.17E-09 | 4.11E-08 | up |
| FCGR1CP | 29.88271 | 1.742904 | 0.299784 | 5.81387 | 6.10E-09 | 4.78E-08 | up |
| TRIM71 | 6.962662 | 1.773022 | 0.305914 | 5.795813 | 6.80E-09 | 5.27E-08 | up |
| ACOX2 | 2.543802 | 2.457574 | 0.424453 | 5.789976 | 7.04E-09 | 5.44E-08 | up |
| COL17A1 | 16.46279 | 2.248737 | 0.388824 | 5.783434 | 7.32E-09 | 5.65E-08 | up |
| NT5DC4 | 14.22103 | 1.09529 | 0.190014 | 5.764242 | 8.20E-09 | 6.28E-08 | up |
| PTGR1 | 7.629125 | 1.891343 | 0.328409 | 5.75911 | 8.46E-09 | 6.45E-08 | up |
| ARHGAP29 | 17.01074 | 1.457019 | 0.253209 | 5.754206 | 8.70E-09 | 6.63E-08 | up |
| RN7SL587P | 6.947348 | 1.416964 | 0.246591 | 5.746205 | 9.13E-09 | 6.92E-08 | up |
| LINC01259 | 14.09852 | -1.15958 | 0.201909 | -5.74306 | 9.30E-09 | 7.03E-08 | down |
| GABRR2 | 15.27742 | 1.089555 | 0.189897 | 5.737619 | 9.60E-09 | 7.24E-08 | up |
| ACTA2-AS1 | 4.890793 | -1.03658 | 0.180983 | -5.72749 | 1.02E-08 | 7.64E-08 | down |
| TRAV38-2DV8 | 8.840652 | -1.1464 | 0.200804 | -5.70907 | 1.14E-08 | 8.44E-08 | down |
| CPE | 2.284689 | 2.971004 | 0.520958 | 5.702964 | 1.18E-08 | 8.73E-08 | up |
| LRRC70 | 10.48323 | 1.888223 | 0.331617 | 5.693979 | 1.24E-08 | 9.16E-08 | up |
| ATP11B | 2653.244 | 1.039783 | 0.18261 | 5.694018 | 1.24E-08 | 9.16E-08 | up |
| RNY1P16 | 5.978024 | 1.200518 | 0.211345 | 5.680363 | 1.34E-08 | 9.86E-08 | up |
| RN7SL491P | 6.954408 | 1.343036 | 0.236598 | 5.676443 | 1.38E-08 | 1.01E-07 | up |
| EDNRB | 5.918711 | 2.484082 | 0.437903 | 5.672678 | 1.41E-08 | 1.02E-07 | up |
| ADGRE1 | 694.6716 | 1.161976 | 0.205241 | 5.661513 | 1.50E-08 | 1.08E-07 | up |
| PI3 | 954.648 | 1.40006 | 0.248728 | 5.628877 | 1.81E-08 | 1.29E-07 | up |
| ACVR1C | 23.9034 | -1.08299 | 0.192786 | -5.61759 | 1.94E-08 | 1.37E-07 | down |
| ERLIN1 | 434.0345 | 1.083886 | 0.192948 | 5.617517 | 1.94E-08 | 1.37E-07 | up |
| APCDD1 | 14.75259 | 1.319297 | 0.235125 | 5.611042 | 2.01E-08 | 1.42E-07 | up |
| SPON1 | 11.85639 | -1.17717 | 0.210114 | -5.6025 | 2.11E-08 | 1.48E-07 | down |
| MYL12-AS1 | 10.98676 | 1.091658 | 0.195411 | 5.586464 | 2.32E-08 | 1.61E-07 | up |
| LINC00968 | 16.69114 | 1.03509 | 0.185336 | 5.584945 | 2.34E-08 | 1.62E-07 | up |
| STOM | 2170.063 | 1.280953 | 0.229616 | 5.578674 | 2.42E-08 | 1.67E-07 | up |
| RSPH14 | 7.826651 | 1.441836 | 0.258959 | 5.567818 | 2.58E-08 | 1.76E-07 | up |
| ADGRG2 | 2.956988 | 2.801005 | 0.503326 | 5.564989 | 2.62E-08 | 1.79E-07 | up |
| AKR1C1 | 11.63631 | 1.596105 | 0.287049 | 5.560387 | 2.69E-08 | 1.83E-07 | up |
| TRAJ12 | 1.92609 | -1.28714 | 0.232375 | -5.53906 | 3.04E-08 | 2.03E-07 | down |
| SCN9A | 41.84653 | 1.324423 | 0.239124 | 5.538641 | 3.05E-08 | 2.04E-07 | up |
| MIR7848 | 7.780515 | 1.107438 | 0.200089 | 5.534724 | 3.12E-08 | 2.08E-07 | up |
| SYNPO | 21.52425 | -1.00102 | 0.180889 | -5.53391 | 3.13E-08 | 2.09E-07 | down |
| KRT23 | 1356.549 | 1.037163 | 0.187585 | 5.529029 | 3.22E-08 | 2.14E-07 | up |
| KLLN | 25.5632 | 1.04779 | 0.189596 | 5.526435 | 3.27E-08 | 2.17E-07 | up |
| MYOSLID | 5.726524 | 2.222277 | 0.402225 | 5.524953 | 3.30E-08 | 2.19E-07 | up |
| ZNF516-DT | 3.320633 | 1.339692 | 0.242572 | 5.522853 | 3.34E-08 | 2.21E-07 | up |
| MIR635 | 4.710458 | 1.220837 | 0.221051 | 5.522863 | 3.34E-08 | 2.21E-07 | up |
| VCAN-AS1 | 4.356156 | 1.499815 | 0.271615 | 5.521835 | 3.35E-08 | 2.22E-07 | up |
| MTND6P5 | 5.461928 | 1.311313 | 0.238284 | 5.50316 | 3.73E-08 | 2.43E-07 | up |
| MARCO | 120.5988 | 1.306646 | 0.237496 | 5.501761 | 3.76E-08 | 2.45E-07 | up |
| MIR3605 | 9.207255 | 1.364602 | 0.248554 | 5.490171 | 4.02E-08 | 2.60E-07 | up |
| FRMD4B | 285.437 | 1.010723 | 0.184242 | 5.485842 | 4.12E-08 | 2.66E-07 | up |
| EPPK1 | 11.95818 | -1.12872 | 0.206398 | -5.46866 | 4.53E-08 | 2.90E-07 | down |
| TMIGD3 | 15.75564 | 1.537044 | 0.281245 | 5.465148 | 4.63E-08 | 2.95E-07 | up |
| HSD3B7 | 16.62831 | 1.232408 | 0.225768 | 5.458736 | 4.80E-08 | 3.05E-07 | up |
| RNASE3 | 23.47302 | 1.481549 | 0.271423 | 5.458447 | 4.80E-08 | 3.05E-07 | up |
| LOH12CR2 | 4.299221 | 1.270048 | 0.232816 | 5.455165 | 4.89E-08 | 3.10E-07 | up |
| NTSR1 | 11.98933 | 1.486531 | 0.273016 | 5.444851 | 5.18E-08 | 3.28E-07 | up |
| PLPP3 | 10.41683 | 1.209242 | 0.222096 | 5.44469 | 5.19E-08 | 3.28E-07 | up |
| TCN1 | 141.3503 | 1.307317 | 0.240711 | 5.431064 | 5.60E-08 | 3.52E-07 | up |
| MTHFS | 29.49467 | 1.104637 | 0.203525 | 5.427539 | 5.71E-08 | 3.58E-07 | up |
| CHIT1 | 26.64868 | 1.287634 | 0.237828 | 5.414147 | 6.16E-08 | 3.83E-07 | up |
| IRS2 | 904.2369 | 1.03842 | 0.192532 | 5.393493 | 6.91E-08 | 4.25E-07 | up |
| CLEC1B | 40.73869 | 1.335881 | 0.248731 | 5.370787 | 7.84E-08 | 4.79E-07 | up |
| SAP30 | 73.46229 | 1.010806 | 0.188653 | 5.358018 | 8.41E-08 | 5.10E-07 | up |
| RAB13 | 14.26407 | 1.204147 | 0.224799 | 5.356559 | 8.48E-08 | 5.12E-07 | up |
| SGCD | 4.601553 | -1.67387 | 0.312574 | -5.35512 | 8.55E-08 | 5.16E-07 | down |
| MYO7A | 26.22448 | 1.056719 | 0.197361 | 5.354253 | 8.59E-08 | 5.17E-07 | up |
| PTGFR | 11.89358 | 2.022576 | 0.3779 | 5.352151 | 8.69E-08 | 5.23E-07 | up |
| IFITM3 | 1873.292 | 1.32806 | 0.248185 | 5.351081 | 8.74E-08 | 5.26E-07 | up |
| CXCR3 | 34.81309 | -1.01363 | 0.189541 | -5.3478 | 8.90E-08 | 5.35E-07 | down |
| MAOA | 11.94303 | 2.18453 | 0.409397 | 5.335967 | 9.50E-08 | 5.68E-07 | up |
| NUDT16-DT | 21.44409 | 1.187095 | 0.222753 | 5.329194 | 9.86E-08 | 5.88E-07 | up |
| PIPSL | 3.525669 | 1.015343 | 0.190576 | 5.327754 | 9.94E-08 | 5.92E-07 | up |
| BCAT1 | 200.0302 | 1.160747 | 0.218118 | 5.321649 | 1.03E-07 | 6.11E-07 | up |
| MIR4271 | 2.173227 | 2.101102 | 0.395118 | 5.31766 | 1.05E-07 | 6.23E-07 | up |
| BEND7 | 57.87888 | 1.063503 | 0.200125 | 5.314205 | 1.07E-07 | 6.34E-07 | up |
| KLRC4 | 7.044803 | -1.12518 | 0.212176 | -5.30307 | 1.14E-07 | 6.72E-07 | down |
| TRBV15 | 10.12212 | -1.25332 | 0.236417 | -5.3013 | 1.15E-07 | 6.78E-07 | down |
| SRGAP1 | 8.641737 | 1.418762 | 0.267963 | 5.294617 | 1.19E-07 | 6.99E-07 | up |
| H2BC6 | 19.97693 | 1.004804 | 0.190006 | 5.288277 | 1.23E-07 | 7.21E-07 | up |
| NDST3 | 2.052442 | 2.887762 | 0.546481 | 5.284284 | 1.26E-07 | 7.35E-07 | up |
| KCNE1B | 5.727318 | 2.129532 | 0.40393 | 5.272038 | 1.35E-07 | 7.81E-07 | up |
| PGDP1 | 4.46679 | 1.404894 | 0.266623 | 5.269223 | 1.37E-07 | 7.91E-07 | up |
| CT75 | 1.421923 | 2.67073 | 0.506897 | 5.268777 | 1.37E-07 | 7.92E-07 | up |
| SIGLEC11 | 6.912862 | 1.434001 | 0.272368 | 5.264931 | 1.40E-07 | 8.07E-07 | up |
| DHRS9 | 452.8651 | 1.23584 | 0.235244 | 5.253448 | 1.49E-07 | 8.55E-07 | up |
| PPBP | 826.1691 | 1.441177 | 0.274399 | 5.252117 | 1.50E-07 | 8.59E-07 | up |
| RAD54B | 2.989138 | -1.08313 | 0.206406 | -5.24758 | 1.54E-07 | 8.80E-07 | down |
| SAMD15 | 7.274424 | 1.313169 | 0.250271 | 5.246991 | 1.55E-07 | 8.82E-07 | up |
| DOCK4 | 282.7931 | 1.036825 | 0.197632 | 5.246242 | 1.55E-07 | 8.85E-07 | up |
| DSC2 | 1087.877 | 1.127749 | 0.2152 | 5.240458 | 1.60E-07 | 9.10E-07 | up |
| SMIM10 | 3.839757 | 1.498997 | 0.286329 | 5.235223 | 1.65E-07 | 9.34E-07 | up |
| TREML3P | 44.75583 | 1.270527 | 0.24319 | 5.224414 | 1.75E-07 | 9.85E-07 | up |
| RPS10P1 | 10.52562 | 1.102376 | 0.211007 | 5.224351 | 1.75E-07 | 9.85E-07 | up |
| BIK | 10.59702 | 1.06684 | 0.204762 | 5.210148 | 1.89E-07 | 1.06E-06 | up |
| MYLK3 | 6.206628 | 1.40514 | 0.270009 | 5.204042 | 1.95E-07 | 1.09E-06 | up |
| DYNLT5 | 9.858619 | 2.249813 | 0.432497 | 5.201918 | 1.97E-07 | 1.10E-06 | up |
| TRGV9 | 22.0852 | -1.16111 | 0.223252 | -5.20092 | 1.98E-07 | 1.10E-06 | down |
| SLC16A10 | 46.15205 | -1.0971 | 0.21105 | -5.19831 | 2.01E-07 | 1.12E-06 | down |
| IGFBP2 | 5.171509 | 2.105985 | 0.405156 | 5.197964 | 2.01E-07 | 1.12E-06 | up |
| LCN2 | 275.7599 | 1.682561 | 0.323838 | 5.195693 | 2.04E-07 | 1.13E-06 | up |
| RNU6-37P | 2.203194 | 1.928108 | 0.371208 | 5.194152 | 2.06E-07 | 1.14E-06 | up |
| PTPRK | 33.46566 | -1.01887 | 0.19619 | -5.1933 | 2.07E-07 | 1.14E-06 | down |
| ARHGAP20 | 1.755098 | -1.33219 | 0.257027 | -5.18309 | 2.18E-07 | 1.20E-06 | down |
| TRAJ6 | 1.873493 | -1.19511 | 0.231084 | -5.17174 | 2.32E-07 | 1.27E-06 | down |
| IMPDH1P10 | 2.586139 | 1.366978 | 0.265031 | 5.15781 | 2.50E-07 | 1.35E-06 | up |
| OLFM4 | 206.7876 | 2.149456 | 0.416766 | 5.157469 | 2.50E-07 | 1.35E-06 | up |
| TRAJ45 | 1.123689 | -1.63355 | 0.316753 | -5.15717 | 2.51E-07 | 1.36E-06 | down |
| INHBB | 19.71055 | 1.394553 | 0.270706 | 5.151539 | 2.58E-07 | 1.39E-06 | up |
| HNRNPA1P70 | 6.354754 | -1.16444 | 0.226087 | -5.15041 | 2.60E-07 | 1.40E-06 | down |
| RNU1-134P | 3.093446 | 1.199911 | 0.234151 | 5.124527 | 2.98E-07 | 1.59E-06 | up |
| KRTAP5-AS1 | 3.78451 | -1.09611 | 0.213943 | -5.12339 | 3.00E-07 | 1.60E-06 | down |
| PTGES | 13.01097 | 1.591056 | 0.311234 | 5.112091 | 3.19E-07 | 1.69E-06 | up |
| TRAJ13 | 1.407392 | -1.41865 | 0.277547 | -5.1114 | 3.20E-07 | 1.69E-06 | down |
| BPI | 156.6357 | 1.423422 | 0.278728 | 5.106856 | 3.28E-07 | 1.73E-06 | up |
| NDUFB8P2 | 3.262807 | 1.267274 | 0.248676 | 5.096084 | 3.47E-07 | 1.82E-06 | up |
| TRMT9B | 4.652453 | 2.040578 | 0.400874 | 5.090326 | 3.57E-07 | 1.87E-06 | up |
| RNU6-196P | 4.506244 | 1.476354 | 0.290029 | 5.090357 | 3.57E-07 | 1.87E-06 | up |
| RBM20 | 2.85704 | -1.21205 | 0.238336 | -5.08547 | 3.67E-07 | 1.91E-06 | down |
| ATP2C2 | 22.45218 | 1.95134 | 0.384283 | 5.077874 | 3.82E-07 | 1.98E-06 | up |
| PRUNE2 | 76.03902 | 1.664996 | 0.328186 | 5.073336 | 3.91E-07 | 2.03E-06 | up |
| KAZN | 100.4495 | 1.039 | 0.204913 | 5.07045 | 3.97E-07 | 2.05E-06 | up |
| CAVIN3 | 4.196012 | 1.804742 | 0.356091 | 5.068204 | 4.02E-07 | 2.07E-06 | up |
| SNORA77 | 4.307696 | 1.128465 | 0.22298 | 5.06083 | 4.17E-07 | 2.15E-06 | up |
| POU5F1 | 8.451045 | 1.185195 | 0.234627 | 5.051404 | 4.39E-07 | 2.25E-06 | up |
| EXOSC4 | 18.84135 | 1.17007 | 0.231652 | 5.050981 | 4.40E-07 | 2.25E-06 | up |
| RPS3AP43 | 5.803247 | 1.468455 | 0.290996 | 5.046302 | 4.50E-07 | 2.30E-06 | up |
| DNAAF4 | 2.115847 | 1.651012 | 0.327227 | 5.045464 | 4.52E-07 | 2.31E-06 | up |
| OVCH1 | 1.952301 | 2.489559 | 0.494721 | 5.032252 | 4.85E-07 | 2.46E-06 | up |
| MIR765 | 0.926889 | 1.940053 | 0.385885 | 5.02754 | 4.97E-07 | 2.52E-06 | up |
| FRMPD3 | 15.30912 | -1.02188 | 0.203402 | -5.02393 | 5.06E-07 | 2.56E-06 | down |
| LINC01303 | 16.25537 | 1.006761 | 0.200415 | 5.023373 | 5.08E-07 | 2.57E-06 | up |
| CLU | 957.1203 | 1.136172 | 0.226927 | 5.006764 | 5.54E-07 | 2.77E-06 | up |
| LIPC-AS1 | 1.793536 | 2.01775 | 0.403014 | 5.006651 | 5.54E-07 | 2.77E-06 | up |
| LINC01191 | 4.582214 | 1.17267 | 0.234308 | 5.004829 | 5.59E-07 | 2.79E-06 | up |
| MMP19 | 6.768197 | 1.144249 | 0.229229 | 4.991739 | 5.98E-07 | 2.97E-06 | up |
| CLEC6A | 15.98145 | 1.166296 | 0.234647 | 4.97042 | 6.68E-07 | 3.28E-06 | up |
| FCGBP | 36.03731 | -1.15216 | 0.232297 | -4.95984 | 7.06E-07 | 3.46E-06 | down |
| CYB5R2 | 7.187744 | 1.558425 | 0.314548 | 4.954495 | 7.25E-07 | 3.54E-06 | up |
| DKK3 | 4.640739 | -1.13694 | 0.229506 | -4.95385 | 7.28E-07 | 3.55E-06 | down |
| FFAR3 | 3.919218 | 1.700023 | 0.343703 | 4.9462 | 7.57E-07 | 3.67E-06 | up |
| NT5C3AP1 | 18.9938 | 1.038368 | 0.210107 | 4.942084 | 7.73E-07 | 3.74E-06 | up |
| LINC01267 | 1.531173 | 3.003358 | 0.608658 | 4.934396 | 8.04E-07 | 3.88E-06 | up |
| TMEM92-AS1 | 13.19303 | 1.092072 | 0.221894 | 4.92159 | 8.58E-07 | 4.12E-06 | up |
| HPN | 2.709897 | 1.799248 | 0.366973 | 4.90295 | 9.44E-07 | 4.49E-06 | up |
| TRAJ14 | 1.049507 | -1.4338 | 0.292507 | -4.90178 | 9.50E-07 | 4.51E-06 | down |
| RPL23AP21 | 15.54761 | 1.078586 | 0.220223 | 4.897694 | 9.70E-07 | 4.60E-06 | up |
| NELL2 | 399.7545 | -1.02613 | 0.209566 | -4.89647 | 9.76E-07 | 4.62E-06 | down |
| SEMA3B | 6.302067 | 1.093128 | 0.223323 | 4.894826 | 9.84E-07 | 4.65E-06 | up |
| LHX4 | 3.088551 | 2.540093 | 0.52059 | 4.87926 | 1.06E-06 | 5.00E-06 | up |
| NSG2 | 2.668143 | 1.824009 | 0.374199 | 4.874436 | 1.09E-06 | 5.12E-06 | up |
| DGUOK-AS1 | 3.04228 | 1.220428 | 0.25068 | 4.868468 | 1.12E-06 | 5.26E-06 | up |
| LINC01554 | 2.8784 | 1.077818 | 0.221708 | 4.861423 | 1.17E-06 | 5.43E-06 | up |
| EDAR | 29.88021 | -1.06046 | 0.218985 | -4.84261 | 1.28E-06 | 5.91E-06 | down |
| SLC28A3 | 12.27525 | 1.310333 | 0.271497 | 4.82632 | 1.39E-06 | 6.37E-06 | up |
| ERCC6L | 5.249217 | -1.08994 | 0.22632 | -4.81593 | 1.47E-06 | 6.67E-06 | down |
| RNU6-790P | 3.355242 | 1.062873 | 0.221237 | 4.804218 | 1.55E-06 | 7.04E-06 | up |
| IL27 | 3.742255 | 1.365177 | 0.285138 | 4.787777 | 1.69E-06 | 7.58E-06 | up |
| RPL10AP6 | 17.68695 | -1.01323 | 0.211627 | -4.78782 | 1.69E-06 | 7.58E-06 | down |
| LINC01305 | 1.548472 | 2.260802 | 0.472436 | 4.785412 | 1.71E-06 | 7.66E-06 | up |
| LOC100129931 | 6.469247 | 1.004096 | 0.210446 | 4.771273 | 1.83E-06 | 8.15E-06 | up |
| ALOX15B | 9.791133 | 1.340553 | 0.281777 | 4.757489 | 1.96E-06 | 8.67E-06 | up |
| SLCO2B1 | 1.123309 | 2.300037 | 0.483623 | 4.755851 | 1.98E-06 | 8.74E-06 | up |
| TRAJ5 | 2.054994 | -1.10205 | 0.231865 | -4.75299 | 2.00E-06 | 8.86E-06 | down |
| CAPN13 | 3.237207 | 1.906063 | 0.401698 | 4.745018 | 2.08E-06 | 9.18E-06 | up |
| MUC16 | 1.26593 | -1.46783 | 0.30934 | -4.74505 | 2.08E-06 | 9.18E-06 | down |
| PLBD1-AS1 | 14.96711 | 1.037629 | 0.219003 | 4.737963 | 2.16E-06 | 9.44E-06 | up |
| FIGN | 2.074253 | 3.08312 | 0.651445 | 4.732738 | 2.22E-06 | 9.66E-06 | up |
| SLC9A4 | 1.304185 | 2.588396 | 0.547208 | 4.730188 | 2.24E-06 | 9.77E-06 | up |
| ROM1 | 7.718485 | 1.031443 | 0.218278 | 4.725361 | 2.30E-06 | 9.99E-06 | up |
| EPCAM | 1.862787 | 1.896268 | 0.401372 | 4.724464 | 2.31E-06 | 1.00E-05 | up |
| GPR141BP | 2.936772 | 1.508393 | 0.319967 | 4.71422 | 2.43E-06 | 1.05E-05 | up |
| TSPAN16 | 2.258723 | 1.31586 | 0.279345 | 4.710522 | 2.47E-06 | 1.07E-05 | up |
| RNU6-1005P | 10.64835 | 1.039065 | 0.220766 | 4.706624 | 2.52E-06 | 1.09E-05 | up |
| LINC02777 | 3.448066 | 1.196959 | 0.254356 | 4.705843 | 2.53E-06 | 1.09E-05 | up |
| PROS1 | 55.92976 | 1.161455 | 0.248135 | 4.680734 | 2.86E-06 | 1.22E-05 | up |
| FSTL4 | 6.832805 | 1.711189 | 0.365622 | 4.680209 | 2.87E-06 | 1.22E-05 | up |
| LINC00664 | 14.76374 | 1.456636 | 0.311291 | 4.679337 | 2.88E-06 | 1.22E-05 | up |
| RNA5SP207 | 2.414054 | 1.630643 | 0.348754 | 4.675624 | 2.93E-06 | 1.24E-05 | up |
| TRAJ30 | 0.874806 | -1.40814 | 0.301626 | -4.66851 | 3.03E-06 | 1.28E-05 | down |
| LSMEM2 | 1.651161 | 1.508408 | 0.323342 | 4.665055 | 3.09E-06 | 1.31E-05 | up |
| OTX1 | 46.54605 | 1.13773 | 0.244112 | 4.660696 | 3.15E-06 | 1.33E-05 | up |
| TMEM45A | 3.472849 | 1.612576 | 0.346077 | 4.659589 | 3.17E-06 | 1.34E-05 | up |
| NKD1 | 16.94854 | -1.0389 | 0.224001 | -4.63792 | 3.52E-06 | 1.47E-05 | down |
| CNTNAP3 | 348.7132 | 1.084289 | 0.234333 | 4.627124 | 3.71E-06 | 1.55E-05 | up |
| NXNL2 | 1.365699 | 1.871346 | 0.405569 | 4.614123 | 3.95E-06 | 1.64E-05 | up |
| KCNE5 | 2.69503 | 1.267941 | 0.275069 | 4.609538 | 4.04E-06 | 1.67E-05 | up |
| ANOS1 | 6.962244 | 1.71541 | 0.373069 | 4.598107 | 4.26E-06 | 1.75E-05 | up |
| RASGRF1 | 2.487068 | -1.45556 | 0.317376 | -4.58623 | 4.51E-06 | 1.84E-05 | down |
| FOSL1 | 1.757931 | 1.644888 | 0.359937 | 4.569928 | 4.88E-06 | 1.98E-05 | up |
| LOC100129734 | 2.318947 | 1.184269 | 0.259383 | 4.565715 | 4.98E-06 | 2.02E-05 | up |
| LOC100422497 | 2.206491 | 1.298755 | 0.284514 | 4.56482 | 5.00E-06 | 2.02E-05 | up |
| NTN4 | 6.162053 | -1.10828 | 0.243037 | -4.56015 | 5.11E-06 | 2.07E-05 | down |
| PRMT5-AS1 | 3.265048 | 1.072169 | 0.235145 | 4.55961 | 5.12E-06 | 2.07E-05 | up |
| PODN | 4.575094 | -1.21835 | 0.26749 | -4.55474 | 5.25E-06 | 2.12E-05 | down |
| HSD11B1-AS1 | 3.801996 | 1.200711 | 0.263926 | 4.549423 | 5.38E-06 | 2.17E-05 | up |
| IL6 | 1.234004 | -1.30354 | 0.286649 | -4.54749 | 5.43E-06 | 2.18E-05 | down |
| TRAJ40 | 1.132874 | -1.40018 | 0.308958 | -4.53194 | 5.84E-06 | 2.34E-05 | down |
| LINC00853 | 5.610574 | 1.194047 | 0.263566 | 4.530347 | 5.89E-06 | 2.35E-05 | up |
| NAMPTP1 | 492.7019 | 1.034184 | 0.228411 | 4.527742 | 5.96E-06 | 2.38E-05 | up |
| PDZD3 | 4.909695 | 1.050554 | 0.232104 | 4.52622 | 6.00E-06 | 2.40E-05 | up |
| NPAS2 | 10.04559 | -1.04052 | 0.230204 | -4.51996 | 6.19E-06 | 2.46E-05 | down |
| TRAJ7 | 0.921154 | -1.39652 | 0.309396 | -4.51371 | 6.37E-06 | 2.52E-05 | down |
| FARP1 | 18.58393 | 1.113484 | 0.247479 | 4.499303 | 6.82E-06 | 2.69E-05 | up |
| ERBB3 | 3.695049 | -1.13043 | 0.251324 | -4.4979 | 6.86E-06 | 2.70E-05 | down |
| LOC100130044 | 1.62475 | 2.198537 | 0.490721 | 4.480222 | 7.46E-06 | 2.91E-05 | up |
| SHISA2 | 1.866617 | -1.65041 | 0.368799 | -4.4751 | 7.64E-06 | 2.97E-05 | down |
| RPL26P5 | 2.244273 | 1.396946 | 0.312197 | 4.474568 | 7.66E-06 | 2.98E-05 | up |
| PDGFC | 76.24845 | 1.145096 | 0.256033 | 4.472447 | 7.73E-06 | 3.00E-05 | up |
| LINC01980 | 1.114394 | -1.26485 | 0.282992 | -4.46955 | 7.84E-06 | 3.04E-05 | down |
| TFPI | 12.05769 | 1.15699 | 0.259555 | 4.457598 | 8.29E-06 | 3.19E-05 | up |
| WFDC1 | 1.160011 | 2.705551 | 0.60774 | 4.451822 | 8.51E-06 | 3.27E-05 | up |
| RPL35AP26 | 3.044662 | 1.338168 | 0.301403 | 4.439791 | 9.00E-06 | 3.43E-05 | up |
| CD300H | 66.56612 | 1.1304 | 0.254745 | 4.437377 | 9.11E-06 | 3.47E-05 | up |
| MTND2P28 | 863.7412 | -1.04976 | 0.236707 | -4.43485 | 9.21E-06 | 3.51E-05 | down |
| BMP2 | 6.709878 | 1.290135 | 0.291041 | 4.43283 | 9.30E-06 | 3.54E-05 | up |
| IGHEP2 | 5.814998 | 1.431021 | 0.323308 | 4.426182 | 9.59E-06 | 3.64E-05 | up |
| CATIP | 2.50359 | 1.078871 | 0.244239 | 4.417268 | 1.00E-05 | 3.78E-05 | up |
| LHFPL6 | 5.054659 | 1.407366 | 0.318933 | 4.412735 | 1.02E-05 | 3.86E-05 | up |
| PRL | 3.032405 | 2.082546 | 0.472552 | 4.407021 | 1.05E-05 | 3.95E-05 | up |
| FLJ46284 | 4.848026 | 1.106734 | 0.251144 | 4.406766 | 1.05E-05 | 3.95E-05 | up |
| RNU6ATAC39P | 5.073341 | 1.224765 | 0.279201 | 4.386684 | 1.15E-05 | 4.30E-05 | up |
| DRC7 | 5.515717 | 1.082449 | 0.247148 | 4.37976 | 1.19E-05 | 4.42E-05 | up |
| TWIST2 | 2.923873 | 2.302206 | 0.526027 | 4.376595 | 1.21E-05 | 4.48E-05 | up |
| MIR6131 | 1.256021 | 1.715649 | 0.392222 | 4.374179 | 1.22E-05 | 4.52E-05 | up |
| LRRC75B | 4.49669 | 1.124107 | 0.257735 | 4.361481 | 1.29E-05 | 4.77E-05 | up |
| KLRC3 | 5.272457 | -1.10907 | 0.254786 | -4.35295 | 1.34E-05 | 4.94E-05 | down |
| NFASC | 1.020356 | 1.946039 | 0.447685 | 4.346897 | 1.38E-05 | 5.06E-05 | up |
| FBLN2 | 5.200517 | -1.03515 | 0.23877 | -4.33535 | 1.46E-05 | 5.30E-05 | down |
| ZSCAN23 | 1.256056 | -1.35261 | 0.312045 | -4.33467 | 1.46E-05 | 5.32E-05 | down |
| MIR3155A | 1.30573 | 1.655985 | 0.382437 | 4.330088 | 1.49E-05 | 5.42E-05 | up |
| H2AC8 | 5.799079 | 1.063064 | 0.245793 | 4.325044 | 1.53E-05 | 5.54E-05 | up |
| MIR5690 | 4.332766 | 1.374376 | 0.317947 | 4.322661 | 1.54E-05 | 5.59E-05 | up |
| MTCYBP3 | 2.727223 | 1.318759 | 0.305913 | 4.310892 | 1.63E-05 | 5.87E-05 | up |
| TRAJ18 | 1.635829 | -1.18099 | 0.274017 | -4.30993 | 1.63E-05 | 5.89E-05 | down |
| RCVRN | 9.313731 | 1.296248 | 0.300967 | 4.306952 | 1.66E-05 | 5.96E-05 | up |
| MTND6P3 | 2.758104 | 1.262241 | 0.293548 | 4.299955 | 1.71E-05 | 6.14E-05 | up |
| MAOB | 5.727375 | 1.804848 | 0.419955 | 4.297722 | 1.73E-05 | 6.20E-05 | up |
| ANKRD35 | 5.874365 | 1.028343 | 0.239533 | 4.293116 | 1.76E-05 | 6.32E-05 | up |
| PKD2L1 | 1.634931 | 1.536331 | 0.358618 | 4.284029 | 1.84E-05 | 6.55E-05 | up |
| TPRG1-AS1 | 5.255897 | 1.038317 | 0.242653 | 4.279024 | 1.88E-05 | 6.69E-05 | up |
| TRAJ10 | 1.874958 | -1.0277 | 0.240329 | -4.27622 | 1.90E-05 | 6.76E-05 | down |
| IGHG1 | 277.5892 | -1.26031 | 0.295291 | -4.26802 | 1.97E-05 | 6.98E-05 | down |
| SLC4A10 | 45.82866 | -1.28223 | 0.300503 | -4.26695 | 1.98E-05 | 7.01E-05 | down |
| AREG | 2.578725 | 1.637382 | 0.384526 | 4.258186 | 2.06E-05 | 7.26E-05 | up |
| SCRG1 | 1.905498 | 2.010934 | 0.47502 | 4.233364 | 2.30E-05 | 8.05E-05 | up |
| SYN3 | 0.674933 | -1.53334 | 0.362239 | -4.23294 | 2.31E-05 | 8.06E-05 | down |
| IGHJ5 | 4.600627 | -1.12614 | 0.2661 | -4.23202 | 2.32E-05 | 8.08E-05 | down |
| SHOC1 | 1.106231 | 1.87733 | 0.444124 | 4.227044 | 2.37E-05 | 8.24E-05 | up |
| TEAD3 | 3.596746 | 1.72107 | 0.407635 | 4.222084 | 2.42E-05 | 8.40E-05 | up |
| TRAJ20 | 0.925919 | -1.28779 | 0.305908 | -4.20973 | 2.56E-05 | 8.84E-05 | down |
| TACSTD2 | 14.18402 | 1.528455 | 0.363324 | 4.206869 | 2.59E-05 | 8.92E-05 | up |
| RAB19 | 10.8652 | 1.09053 | 0.259246 | 4.20655 | 2.59E-05 | 8.93E-05 | up |
| TUB | 1.98818 | -1.26159 | 0.300192 | -4.20262 | 2.64E-05 | 9.07E-05 | down |
| NOG | 31.82179 | -1.1914 | 0.283896 | -4.19659 | 2.71E-05 | 9.30E-05 | down |
| NRCAM | 15.58506 | -1.40352 | 0.336548 | -4.17034 | 3.04E-05 | 0.000104 | down |
| CD248 | 11.05434 | -1.06501 | 0.255619 | -4.16641 | 3.09E-05 | 0.000105 | down |
| IFI27 | 51.0196 | 1.519978 | 0.364961 | 4.164766 | 3.12E-05 | 0.000106 | up |
| BEST3 | 1.26516 | 2.13638 | 0.514423 | 4.152966 | 3.28E-05 | 0.000111 | up |
| RN7SL271P | 1.783772 | 1.738114 | 0.419024 | 4.148003 | 3.35E-05 | 0.000113 | up |
| H2AC13 | 2.52434 | 1.397982 | 0.337904 | 4.137223 | 3.52E-05 | 0.000118 | up |
| TMEM200B | 19.21099 | 1.208298 | 0.293421 | 4.117967 | 3.82E-05 | 0.000128 | up |
| RNU6-1111P | 1.423049 | 1.307238 | 0.318177 | 4.108523 | 3.98E-05 | 0.000132 | up |
| SFRP1 | 3.551719 | 2.100682 | 0.511621 | 4.105936 | 4.03E-05 | 0.000134 | up |
| MIR124-1HG | 1.262885 | 3.466456 | 0.845704 | 4.098899 | 4.15E-05 | 0.000137 | up |
| TRAJ1 | 2.491291 | -1.01374 | 0.247837 | -4.09036 | 4.31E-05 | 0.000142 | down |
| H3C8 | 1.860179 | 1.259213 | 0.307885 | 4.089885 | 4.32E-05 | 0.000142 | up |
| LOC105375589 | 1.195476 | -1.36929 | 0.334972 | -4.08776 | 4.36E-05 | 0.000143 | down |
| HMGN1P32 | 2.105432 | 1.338174 | 0.327378 | 4.087556 | 4.36E-05 | 0.000143 | up |
| TRAJ11 | 1.420948 | -1.29532 | 0.316918 | -4.08722 | 4.37E-05 | 0.000144 | down |
| IFTAP | 4.482105 | 1.037221 | 0.254008 | 4.083424 | 4.44E-05 | 0.000146 | up |
| SH2D4B | 5.032058 | 1.03415 | 0.253275 | 4.083113 | 4.44E-05 | 0.000146 | up |
| RNU1-91P | 2.45688 | 1.131852 | 0.278113 | 4.069758 | 4.71E-05 | 0.000153 | up |
| RN7SKP26 | 2.841008 | 1.277218 | 0.31401 | 4.067442 | 4.75E-05 | 0.000155 | up |
| FDPSP3 | 1.966783 | 1.204347 | 0.296358 | 4.063828 | 4.83E-05 | 0.000157 | up |
| ARMC12 | 5.637483 | 1.083689 | 0.267206 | 4.05563 | 5.00E-05 | 0.000162 | up |
| NRN1 | 16.95841 | 1.200723 | 0.296108 | 4.05502 | 5.01E-05 | 0.000162 | up |
| TRAJ48 | 0.922498 | -1.26907 | 0.312983 | -4.05475 | 5.02E-05 | 0.000163 | down |
| TRBJ2-1 | 1.990752 | -1.09479 | 0.270243 | -4.05114 | 5.10E-05 | 0.000165 | down |
| POTEF | 1.032631 | 1.413516 | 0.349056 | 4.049538 | 5.13E-05 | 0.000166 | up |
| FOXQ1 | 2.96118 | 2.310586 | 0.570762 | 4.048251 | 5.16E-05 | 0.000167 | up |
| RNA5SP68 | 1.455485 | 1.251568 | 0.309468 | 4.044264 | 5.25E-05 | 0.000169 | up |
| MAFA-AS1 | 1.920213 | 3.420115 | 0.845817 | 4.043562 | 5.26E-05 | 0.00017 | up |
| SYNC | 2.73663 | 1.626109 | 0.402517 | 4.039855 | 5.35E-05 | 0.000172 | up |
| TRAJ34 | 1.678989 | -1.24291 | 0.307862 | -4.03725 | 5.41E-05 | 0.000174 | down |
| FKBP9P1 | 1.497609 | 1.98358 | 0.49147 | 4.036017 | 5.44E-05 | 0.000174 | up |
| TRAJ39 | 1.745006 | -1.03765 | 0.257169 | -4.0349 | 5.46E-05 | 0.000175 | down |
| RNU6-313P | 1.041335 | 2.128016 | 0.527771 | 4.032082 | 5.53E-05 | 0.000177 | up |
| CCNA1 | 5.777579 | 1.539134 | 0.382649 | 4.022311 | 5.76E-05 | 0.000184 | up |
| TEAD2 | 4.10151 | -1.12383 | 0.279506 | -4.02076 | 5.80E-05 | 0.000185 | down |
| COL5A1 | 2.312167 | -1.13916 | 0.283816 | -4.01373 | 5.98E-05 | 0.00019 | down |
| MIR4802 | 1.611891 | 1.395419 | 0.351007 | 3.975474 | 7.02E-05 | 0.00022 | up |
| TRDJ3 | 0.856068 | -1.17057 | 0.295596 | -3.96004 | 7.49E-05 | 0.000234 | down |
| GPRC5C | 3.154813 | 1.25331 | 0.317008 | 3.953566 | 7.70E-05 | 0.00024 | up |
| TRIM72 | 1.55201 | 1.347766 | 0.341068 | 3.951606 | 7.76E-05 | 0.000241 | up |
| OSBPL6 | 10.23655 | 1.024871 | 0.259589 | 3.948055 | 7.88E-05 | 0.000244 | up |
| RNA5SP180 | 0.962936 | 1.721314 | 0.436606 | 3.942485 | 8.06E-05 | 0.00025 | up |
| MIR1203 | 1.444845 | -1.11403 | 0.282679 | -3.94096 | 8.12E-05 | 0.000251 | down |
| DSCAML1 | 0.944785 | -1.23231 | 0.312776 | -3.93992 | 8.15E-05 | 0.000252 | down |
| PDCD1LG2 | 15.18778 | 1.100144 | 0.279817 | 3.931654 | 8.44E-05 | 0.000261 | up |
| GTF2IRD1P1 | 2.123822 | 1.120222 | 0.285192 | 3.927958 | 8.57E-05 | 0.000264 | up |
| MIR4772 | 1.492365 | 1.769109 | 0.451688 | 3.916658 | 8.98E-05 | 0.000276 | up |
| LTF | 713.0483 | 1.344353 | 0.3433 | 3.915975 | 9.00E-05 | 0.000276 | up |
| LINC01918 | 1.006243 | -1.35538 | 0.346249 | -3.91447 | 9.06E-05 | 0.000278 | down |
| SERPINC1 | 2.134022 | 1.168264 | 0.298479 | 3.914056 | 9.08E-05 | 0.000278 | up |
| BEX1 | 2.366465 | 1.352524 | 0.346531 | 3.903037 | 9.50E-05 | 0.00029 | up |
| MIR26A2 | 1.856127 | 1.117128 | 0.286253 | 3.902587 | 9.52E-05 | 0.00029 | up |
| RPL36AP45 | 1.668055 | 1.075225 | 0.27574 | 3.899421 | 9.64E-05 | 0.000294 | up |
| CFAP126 | 1.710061 | 2.083732 | 0.534526 | 3.898282 | 9.69E-05 | 0.000295 | up |
| NTRK3 | 1.538193 | 1.882958 | 0.483984 | 3.890539 | 0.0001 | 0.000304 | up |
| BAMBI | 4.050322 | 1.140402 | 0.293403 | 3.886816 | 0.000102 | 0.000308 | up |
| CPNE4 | 3.392228 | 1.464272 | 0.377207 | 3.881877 | 0.000104 | 0.000314 | up |
| SAG | 1.407277 | 1.262983 | 0.325413 | 3.881167 | 0.000104 | 0.000314 | up |
| DAB2IP | 2.172634 | -1.01407 | 0.261368 | -3.87985 | 0.000105 | 0.000316 | down |
| MIR5009 | 0.885506 | 1.546299 | 0.399945 | 3.866283 | 0.000111 | 0.000332 | up |
| GPR42 | 1.280885 | 1.691525 | 0.437588 | 3.865568 | 0.000111 | 0.000333 | up |
| LRFN2 | 0.800687 | -1.81625 | 0.470324 | -3.8617 | 0.000113 | 0.000337 | down |
| TNFRSF19 | 1.947385 | -1.01464 | 0.263241 | -3.85442 | 0.000116 | 0.000347 | down |
| SDR42E1P5 | 1.15954 | 2.359972 | 0.612738 | 3.851517 | 0.000117 | 0.00035 | up |
| OR2L13 | 0.777852 | -1.3363 | 0.347576 | -3.84462 | 0.000121 | 0.00036 | down |
| PEX11G | 3.670051 | 1.537257 | 0.40054 | 3.83796 | 0.000124 | 0.000368 | up |
| MKNK1-AS1 | 1.907982 | 1.585391 | 0.413192 | 3.836938 | 0.000125 | 0.000369 | up |
| TRPV4 | 4.666408 | 1.022746 | 0.267335 | 3.82571 | 0.00013 | 0.000385 | up |
| MIR6753 | 1.681662 | 1.10915 | 0.290577 | 3.81706 | 0.000135 | 0.000397 | up |
| SLC44A3 | 5.002231 | 1.156885 | 0.303405 | 3.813002 | 0.000137 | 0.000403 | up |
| KRT5 | 3.053947 | -1.08266 | 0.28428 | -3.80842 | 0.00014 | 0.00041 | down |
| AGAP12P | 1.96029 | -1.53896 | 0.404132 | -3.80805 | 0.00014 | 0.00041 | down |
| COX6B2 | 2.219321 | 2.037831 | 0.536673 | 3.797156 | 0.000146 | 0.000427 | up |
| XCR1 | 8.628957 | 1.197667 | 0.316579 | 3.783157 | 0.000155 | 0.00045 | up |
| ADAM32 | 1.039816 | 2.096328 | 0.554876 | 3.778012 | 0.000158 | 0.000459 | up |
| NXPH4 | 1.834307 | -1.28514 | 0.340461 | -3.7747 | 0.00016 | 0.000464 | down |
| C2 | 7.561938 | 1.057075 | 0.280625 | 3.766859 | 0.000165 | 0.000478 | up |
| MROCKI | 2.397508 | 1.567798 | 0.416682 | 3.762574 | 0.000168 | 0.000485 | up |
| CFAP97D2 | 6.142869 | -1.14994 | 0.30603 | -3.75762 | 0.000172 | 0.000494 | down |
| CRISP2 | 3.706574 | 1.282668 | 0.341536 | 3.755589 | 0.000173 | 0.000497 | up |
| HEY1 | 29.53293 | 1.025916 | 0.273653 | 3.748964 | 0.000178 | 0.00051 | up |
| EPHX4 | 2.824368 | -1.17656 | 0.314275 | -3.74372 | 0.000181 | 0.000519 | down |
| SPAG6 | 4.083477 | 1.117512 | 0.298682 | 3.741481 | 0.000183 | 0.000524 | up |
| GPR17 | 1.300164 | 1.413253 | 0.37838 | 3.735012 | 0.000188 | 0.000536 | up |
| TRAJ22 | 1.010511 | -1.21099 | 0.324223 | -3.73505 | 0.000188 | 0.000536 | down |
| MIR3150BHG | 5.789133 | 1.060911 | 0.284534 | 3.728591 | 0.000193 | 0.000548 | up |
| ITGA2B | 261.9641 | 1.059749 | 0.285332 | 3.714089 | 0.000204 | 0.000578 | up |
| IGKV1-6 | 6.099859 | -1.14771 | 0.309156 | -3.7124 | 0.000205 | 0.000582 | down |
| CD207 | 1.242701 | -1.2792 | 0.346023 | -3.69686 | 0.000218 | 0.000615 | down |
| LILRB5 | 40.10544 | 1.545062 | 0.418148 | 3.695008 | 0.00022 | 0.000618 | up |
| CMBL | 24.24206 | 1.201342 | 0.325205 | 3.694103 | 0.000221 | 0.00062 | up |
| PPL | 3.003345 | 1.125377 | 0.304716 | 3.693204 | 0.000221 | 0.000622 | up |
| LOC102724323 | 1.197377 | 1.418474 | 0.384567 | 3.688494 | 0.000226 | 0.000632 | up |
| GJB6 | 8.62099 | 1.081966 | 0.293561 | 3.68566 | 0.000228 | 0.000639 | up |
| OR2A7 | 1.194821 | -1.01594 | 0.275859 | -3.6828 | 0.000231 | 0.000645 | down |
| AQP10 | 18.76973 | 1.055579 | 0.286742 | 3.681291 | 0.000232 | 0.000648 | up |
| LOC100128770 | 1.07987 | 1.515412 | 0.412152 | 3.676827 | 0.000236 | 0.000659 | up |
| EHF | 1.171888 | -1.22709 | 0.333833 | -3.67577 | 0.000237 | 0.000661 | down |
| RNA5SP498 | 0.804602 | 1.574982 | 0.429056 | 3.670803 | 0.000242 | 0.000673 | up |
| APOBEC3B | 22.26784 | 1.269275 | 0.346121 | 3.667141 | 0.000245 | 0.000681 | up |
| TAS2R18P | 1.541976 | -1.03142 | 0.284704 | -3.62278 | 0.000291 | 0.000798 | down |
| IFITM10 | 1.756616 | 1.068368 | 0.295172 | 3.61948 | 0.000295 | 0.000807 | up |
| FOXC1 | 3.401044 | 1.275905 | 0.352702 | 3.617511 | 0.000297 | 0.000813 | up |
| GPC1 | 1.497828 | -1.04803 | 0.290084 | -3.61286 | 0.000303 | 0.000826 | down |
| GSDMA | 4.774423 | -1.08252 | 0.300061 | -3.60767 | 0.000309 | 0.000841 | down |
| ENHO | 2.317843 | -1.01315 | 0.281045 | -3.60495 | 0.000312 | 0.000849 | down |
| MAPK10 | 3.653955 | 1.111713 | 0.308714 | 3.601108 | 0.000317 | 0.000861 | up |
| SFN | 1.983397 | 1.061285 | 0.294766 | 3.600432 | 0.000318 | 0.000863 | up |
| LINC00222 | 2.815895 | 1.005121 | 0.279605 | 3.59479 | 0.000325 | 0.00088 | up |
| SPTBN2 | 0.97506 | -1.15015 | 0.320163 | -3.5924 | 0.000328 | 0.000888 | down |
| MTCO3P5 | 0.793645 | 1.791754 | 0.499572 | 3.58658 | 0.000335 | 0.000906 | up |
| CRYGN | 1.248948 | -1.41926 | 0.395754 | -3.58622 | 0.000335 | 0.000907 | down |
| STOX2 | 2.5196 | 1.174596 | 0.327733 | 3.584007 | 0.000338 | 0.000914 | up |
| TIMP4 | 1.073851 | 2.793322 | 0.779688 | 3.582614 | 0.00034 | 0.000918 | up |
| MST1L | 1.319659 | -1.24984 | 0.349193 | -3.57921 | 0.000345 | 0.000928 | down |
| ARHGEF28 | 4.165322 | -1.00937 | 0.282868 | -3.56835 | 0.000359 | 0.000963 | down |
| GEM | 1.118129 | -1.19573 | 0.335301 | -3.56613 | 0.000362 | 0.00097 | down |
| MIR6124 | 1.364298 | 1.147738 | 0.321955 | 3.5649 | 0.000364 | 0.000974 | up |
| RPS2P40 | 1.358683 | -1.23283 | 0.345996 | -3.56313 | 0.000366 | 0.00098 | down |
| GPC3 | 1.339297 | -1.31714 | 0.371131 | -3.54899 | 0.000387 | 0.00103 | down |
| CCN2 | 2.401622 | -1.23039 | 0.3467 | -3.54887 | 0.000387 | 0.00103 | down |
| DUXAP1 | 1.851188 | -1.25084 | 0.353627 | -3.53718 | 0.000404 | 0.001072 | down |
| CA12 | 2.940838 | 1.186446 | 0.335472 | 3.536649 | 0.000405 | 0.001074 | up |
| GNG10 | 0.841425 | 1.458168 | 0.412795 | 3.532427 | 0.000412 | 0.00109 | up |
| PRB3 | 0.95816 | -1.38933 | 0.393972 | -3.52647 | 0.000421 | 0.001112 | down |
| NUP210L | 5.970647 | 1.497223 | 0.424788 | 3.524636 | 0.000424 | 0.001118 | up |
| HMGB1P19 | 0.86883 | -1.20035 | 0.341382 | -3.51616 | 0.000438 | 0.001152 | down |
| C15orf65 | 2.957041 | 1.110415 | 0.315869 | 3.515432 | 0.000439 | 0.001154 | up |
| LRRN3 | 140.6951 | -1.05242 | 0.300176 | -3.506 | 0.000455 | 0.001192 | down |
| SMOC1 | 0.880631 | -1.11209 | 0.317678 | -3.50068 | 0.000464 | 0.001213 | down |
| TVP23CP1 | 1.006713 | 1.452643 | 0.415597 | 3.495321 | 0.000473 | 0.001235 | up |
| CIR1P2 | 0.936298 | 1.265315 | 0.362486 | 3.490655 | 0.000482 | 0.001255 | up |
| IGKV1-27 | 6.863565 | -1.07354 | 0.308274 | -3.48243 | 0.000497 | 0.00129 | down |
| ZMAT4 | 2.446878 | -1.19906 | 0.345924 | -3.46626 | 0.000528 | 0.001363 | down |
| CBS | 1.763939 | 1.463984 | 0.42312 | 3.459973 | 0.00054 | 0.001392 | up |
| TM4SF1 | 1.601882 | 1.497179 | 0.433141 | 3.456562 | 0.000547 | 0.001408 | up |
| ZNF683 | 37.66927 | -1.00933 | 0.292922 | -3.44574 | 0.000569 | 0.001459 | down |
| RPL31P18 | 1.426944 | 1.126454 | 0.326944 | 3.445399 | 0.00057 | 0.001461 | up |
| ARHGAP39 | 1.056937 | -1.09496 | 0.318112 | -3.44205 | 0.000577 | 0.001478 | down |
| RNU6-268P | 1.650152 | 1.179729 | 0.342883 | 3.440615 | 0.00058 | 0.001485 | up |
| LINC01300 | 0.875903 | 1.863347 | 0.5417 | 3.439813 | 0.000582 | 0.001489 | up |
| RNU6-757P | 0.827399 | 1.398728 | 0.407173 | 3.435222 | 0.000592 | 0.001512 | up |
| EIF1AY | 163.1671 | 2.218895 | 0.645993 | 3.43486 | 0.000593 | 0.001514 | up |
| RPS20P29 | 0.98082 | 1.441613 | 0.42123 | 3.42239 | 0.000621 | 0.001577 | up |
| RALBP1P1 | 0.904351 | 1.331444 | 0.389356 | 3.419609 | 0.000627 | 0.001592 | up |
| C9orf152 | 2.25966 | 1.329747 | 0.389439 | 3.414523 | 0.000639 | 0.001618 | up |
| RN7SL344P | 1.39637 | 1.112895 | 0.326626 | 3.407248 | 0.000656 | 0.001657 | up |
| CD46P1 | 1.486564 | 1.292915 | 0.379703 | 3.405073 | 0.000661 | 0.001669 | up |
| MIR3945 | 2.140608 | 1.333188 | 0.392572 | 3.396034 | 0.000684 | 0.00172 | up |
| RN7SL681P | 1.558863 | 1.060337 | 0.312507 | 3.392998 | 0.000691 | 0.001738 | up |
| CLEC18A | 2.768615 | 1.067211 | 0.314542 | 3.392908 | 0.000692 | 0.001738 | up |
| SEPT5-GP1BB | 1.853847 | 1.212076 | 0.357835 | 3.387245 | 0.000706 | 0.001771 | up |
| AICDA | 3.849644 | 1.082624 | 0.32042 | 3.378769 | 0.000728 | 0.001823 | up |
| SLC4A9 | 0.763894 | 1.364833 | 0.405526 | 3.36559 | 0.000764 | 0.001903 | up |
| MMP1 | 3.88745 | 1.559133 | 0.46391 | 3.36085 | 0.000777 | 0.001932 | up |
| SLC1A2 | 2.022113 | 1.55013 | 0.461274 | 3.360543 | 0.000778 | 0.001934 | up |
| FAM90A1 | 5.268306 | 1.251362 | 0.374028 | 3.345639 | 0.000821 | 0.002033 | up |
| GPR1 | 1.307494 | 1.8605 | 0.556643 | 3.342358 | 0.000831 | 0.002056 | up |
| MIR1250 | 1.369969 | 1.013013 | 0.303116 | 3.342002 | 0.000832 | 0.002058 | up |
| RPS15AP18 | 1.194125 | 1.106439 | 0.331319 | 3.339497 | 0.000839 | 0.002073 | up |
| IFI44L | 778.1816 | 1.225776 | 0.367823 | 3.332517 | 0.000861 | 0.00212 | up |
| LOC101927018 | 1.984839 | 1.148859 | 0.345026 | 3.329777 | 0.000869 | 0.002137 | up |
| AFAP1L1 | 0.752503 | -1.39124 | 0.419173 | -3.31902 | 0.000903 | 0.002214 | down |
| LINC01736 | 2.223182 | 1.098784 | 0.331178 | 3.317808 | 0.000907 | 0.002223 | up |
| HCG9 | 3.013528 | 1.023003 | 0.308708 | 3.313823 | 0.00092 | 0.002252 | up |
| H1-4 | 0.938279 | 1.186853 | 0.358719 | 3.308593 | 0.000938 | 0.002289 | up |
| TCTE1 | 0.72009 | -1.17626 | 0.35655 | -3.299 | 0.00097 | 0.002359 | down |
| MXRA8 | 1.725939 | -1.14418 | 0.348005 | -3.28782 | 0.00101 | 0.002446 | down |
| SAMD14 | 9.063343 | 1.002239 | 0.305196 | 3.283914 | 0.001024 | 0.002478 | up |
| KCNH7 | 1.407839 | 1.57879 | 0.481135 | 3.281389 | 0.001033 | 0.002499 | up |
| SEPTIN5 | 1.4374 | 1.234613 | 0.37701 | 3.274746 | 0.001058 | 0.00255 | up |
| LOC284798 | 0.94145 | -1.02503 | 0.315148 | -3.25255 | 0.001144 | 0.002733 | down |
| KIR2DS4 | 28.79175 | -1.06152 | 0.327237 | -3.24389 | 0.001179 | 0.00281 | down |
| NDUFB4P11 | 1.287028 | 1.199634 | 0.371144 | 3.232264 | 0.001228 | 0.002916 | up |
| FAM124A | 0.877547 | 1.298948 | 0.401883 | 3.232151 | 0.001229 | 0.002916 | up |
| CD300LD | 13.90712 | 1.132779 | 0.350819 | 3.228957 | 0.001242 | 0.002946 | up |
| PXT1 | 1.611598 | 1.07023 | 0.331909 | 3.22447 | 0.001262 | 0.002987 | up |
| TMED11P | 1.026612 | 1.502046 | 0.466397 | 3.220529 | 0.00128 | 0.003024 | up |
| TAS2R3 | 1.117923 | -1.09746 | 0.340783 | -3.22041 | 0.00128 | 0.003024 | down |
| RN7SL57P | 1.274429 | 1.089788 | 0.340743 | 3.198272 | 0.001383 | 0.003241 | up |
| MTND5P14 | 1.174815 | 1.104459 | 0.345393 | 3.197684 | 0.001385 | 0.003246 | up |
| ZFY-AS1 | 1.165872 | 1.643056 | 0.513908 | 3.197182 | 0.001388 | 0.003251 | up |
| LOC157273 | 4.768648 | 1.447578 | 0.453162 | 3.194396 | 0.001401 | 0.00328 | up |
| LOC101929128 | 4.768648 | 1.447578 | 0.453162 | 3.194396 | 0.001401 | 0.00328 | up |
| AOC1 | 14.031 | 1.20512 | 0.377314 | 3.193943 | 0.001403 | 0.003285 | up |
| MAP1B | 3.754282 | 1.430023 | 0.447775 | 3.193623 | 0.001405 | 0.003287 | up |
| DEFA4 | 27.66655 | 1.196327 | 0.375903 | 3.18254 | 0.00146 | 0.003404 | up |
| RPL23AP81 | 0.763876 | -1.04129 | 0.327393 | -3.18056 | 0.00147 | 0.003424 | down |
| SNX7 | 1.665104 | 1.461464 | 0.460312 | 3.174945 | 0.001499 | 0.003485 | up |
| RNU7-19P | 0.885357 | 1.173743 | 0.371832 | 3.156652 | 0.001596 | 0.00369 | up |
| SLC2A14 | 36.97042 | 1.20012 | 0.380462 | 3.154378 | 0.001608 | 0.003717 | up |
| CFAP97D1 | 1.604387 | 1.230752 | 0.390407 | 3.152482 | 0.001619 | 0.003736 | up |
| CHMP4C | 2.074584 | 1.976162 | 0.627252 | 3.150508 | 0.00163 | 0.003758 | up |
| IHO1 | 1.335843 | 1.259358 | 0.401106 | 3.139716 | 0.001691 | 0.003881 | up |
| SFRP5 | 2.393017 | -1.19513 | 0.381091 | -3.13607 | 0.001712 | 0.003927 | down |
| SIAH3 | 1.533301 | -1.02487 | 0.327417 | -3.13017 | 0.001747 | 0.003999 | down |
| PAPPA2 | 0.822491 | 1.399764 | 0.448624 | 3.120132 | 0.001808 | 0.004125 | up |
| TRDV3 | 1.349739 | -1.26174 | 0.404655 | -3.11805 | 0.001821 | 0.004151 | down |
| NOS1AP | 2.983282 | 1.346643 | 0.432194 | 3.115828 | 0.001834 | 0.00418 | up |
| INHBA | 6.042314 | 1.166253 | 0.375111 | 3.109089 | 0.001877 | 0.00427 | up |
| ELANE | 7.367014 | 1.134859 | 0.366357 | 3.097689 | 0.00195 | 0.004424 | up |
| RSAD2 | 960.1417 | 1.169606 | 0.379078 | 3.085394 | 0.002033 | 0.004584 | up |
| PKD1L3 | 1.637012 | 1.279605 | 0.416916 | 3.069215 | 0.002146 | 0.004815 | up |
| DRC1 | 1.707362 | 1.143928 | 0.375226 | 3.048636 | 0.002299 | 0.005124 | up |
| RNU6-90P | 1.219856 | 1.039707 | 0.341549 | 3.044094 | 0.002334 | 0.005193 | up |
| HHATL | 0.986797 | 1.079351 | 0.354659 | 3.043349 | 0.00234 | 0.005204 | up |
| CFAP73 | 1.291324 | -1.0165 | 0.334817 | -3.03598 | 0.002398 | 0.005314 | down |
| C1QTNF12 | 0.913208 | 1.091815 | 0.361798 | 3.017745 | 0.002547 | 0.005604 | up |
| KCTD19 | 0.996648 | -1.08005 | 0.358091 | -3.01613 | 0.00256 | 0.00563 | down |
| MYO16-AS1 | 4.134478 | 1.054979 | 0.350145 | 3.012977 | 0.002587 | 0.005685 | up |
| LY6G6C | 1.762709 | 1.016469 | 0.337586 | 3.010989 | 0.002604 | 0.005718 | up |
| RPL27P10 | 0.866433 | 1.536756 | 0.511192 | 3.006218 | 0.002645 | 0.005798 | up |
| CYP1A1 | 0.89505 | 1.570375 | 0.523448 | 3.000062 | 0.002699 | 0.005905 | up |
| XIRP2 | 1.282146 | 1.244265 | 0.414978 | 2.998385 | 0.002714 | 0.005931 | up |
| WNT6 | 0.836284 | 1.216773 | 0.405813 | 2.998356 | 0.002714 | 0.005931 | up |
| ZNF727 | 0.97585 | -1.06349 | 0.355036 | -2.99544 | 0.002741 | 0.005981 | down |
| CCDC39 | 1.724718 | 1.103798 | 0.368856 | 2.992487 | 0.002767 | 0.006031 | up |
| IGHV4-4 | 5.800355 | -1.13093 | 0.378306 | -2.98946 | 0.002795 | 0.006086 | down |
| BEND6 | 1.005526 | -1.00273 | 0.335669 | -2.98726 | 0.002815 | 0.006125 | down |
| TRHDE | 2.404038 | 1.258057 | 0.421274 | 2.986314 | 0.002824 | 0.006142 | up |
| CHRNA9 | 0.694335 | -1.20284 | 0.406272 | -2.96069 | 0.00307 | 0.006624 | down |
| PRSS35 | 3.139467 | -1.10796 | 0.379157 | -2.92216 | 0.003476 | 0.007398 | down |
| SCN2B | 2.989306 | 1.130386 | 0.388194 | 2.911908 | 0.003592 | 0.007616 | up |
| TRDJ4 | 0.730156 | -1.02923 | 0.353478 | -2.91173 | 0.003594 | 0.007619 | down |
| U2AF1L5 | 44.75775 | 1.069705 | 0.368536 | 2.902582 | 0.003701 | 0.007819 | up |
| NEBL | 45.98695 | 1.425754 | 0.494014 | 2.886059 | 0.003901 | 0.008179 | up |
| GPR85 | 2.200854 | 1.004444 | 0.348567 | 2.881638 | 0.003956 | 0.008278 | up |
| GPR37L1 | 1.034485 | 1.225639 | 0.430112 | 2.849578 | 0.004378 | 0.009071 | up |
| USP6 | 2.507923 | -1.01849 | 0.358124 | -2.84397 | 0.004456 | 0.009219 | down |
| SNORA74B | 1.208906 | 1.033866 | 0.363795 | 2.841892 | 0.004485 | 0.009271 | up |
| PDE6B-AS1 | 0.770564 | -1.18128 | 0.41638 | -2.83701 | 0.004554 | 0.009393 | down |
| LOC101929823 | 1.446695 | 1.300133 | 0.458566 | 2.835213 | 0.00458 | 0.00944 | up |
| CFAP46 | 1.381407 | 1.437602 | 0.507155 | 2.83464 | 0.004588 | 0.009455 | up |
| EZR-AS1 | 0.914951 | -1.02962 | 0.363921 | -2.82925 | 0.004666 | 0.009604 | down |
| ERFE | 2.733151 | 1.259396 | 0.447146 | 2.816519 | 0.004855 | 0.009961 | up |
| EPB41L4B | 1.504535 | 2.528008 | 0.897579 | 2.816474 | 0.004855 | 0.009962 | up |
