# Supplementary material for: Novel insight on marker genes and pathogenic peripheral neutrophil subtypes in acute pancreatitis
Source: Front Immunol. 2022 Aug 22;13:964622. doi: 10.3389/fimmu.2022.964622 (PMC9444397; doi:10.3389/fimmu.2022.964622)
Supplement: Supplementary file 6 [file Table_4.docx]

Table S4. The gene list in gene modules.

| Brown | Turquoise |
| --- | --- |
| FGR | CD99 |
| SNX11 | M6PR |
| SKAP2 | CASP10 |
| FAM214B | RBM6 |
| MSL3 | RECQL |
| GDE1 | SLC25A5 |
| GAS7 | MCUB |
| PGLYRP1 | MYCBP2 |
| MMP25 | ITGAL |
| SEC62 | AP2B1 |
| TYROBP | RALA |
| ALOX5 | ADIPOR2 |
| MVP | KIAA0100 |
| WAS | CD79B |
| SLC11A1 | LUC7L |
| MARCO | PSMB1 |
| SERPINB1 | SYPL1 |
| RTF2 | ST3GAL1 |
| VIM | IL32 |
| CD44 | HEATR5B |
| IFNGR1 | CSDE1 |
| GRN | TRAF3IP3 |
| MAP2K3 | BAZ1B |
| GABARAPL2 | CD4 |
| TIMP2 | FYN |
| VCAN | HIVEP2 |
| CAPG | UTP18 |
| CTNNA1 | RNF216 |
| VAMP3 | PTBP1 |
| CYBA | CD22 |
| RRP12 | CALCOCO1 |
| KIF1B | PSMC4 |
| ATP9A | CD6 |
| ATP11B | UFL1 |
| ATP2B4 | CCDC88C |
| YIPF1 | ZMYND11 |
| SLC2A3 | CD74 |
| ACAA1 | RUNX3 |
| DGAT2 | RNF10 |
| CTSA | BIRC3 |
| CNN2 | AKAP11 |
| SBNO2 | STRAP |
| GNAI3 | DEF6 |
| OAT | PLEKHO1 |
| CYB5R4 | PHF20 |
| YBX1 | KPNA6 |
| SPI1 | SLAMF7 |
| IDI1 | BTN3A1 |
| TNFRSF1A | PRKCH |
| PKM | POU2F2 |
| RHOA | RABEP1 |
| MEF2A | FAM13B |
| TFE3 | SARS1 |
| ACSL4 | TMSB10 |
| LAPTM4A | ADSS2 |
| BCL3 | STAP1 |
| RAB27A | RFC1 |
| TMEM260 | NSUN2 |
| WIPI1 | MAT2B |
| PRKACA | MTREX |
| UBE2D1 | LSG1 |
| IRAG1 | AP2S1 |
| ALPK1 | JADE2 |
| DHRS9 | GOPC |
| ENO1 | USP28 |
| SLC25A40 | MRPS10 |
| FNDC3B | NOP58 |
| FOSL2 | SZRD1 |
| ACTB | CUL1 |
| RAB7A | CYFIP2 |
| ARHGAP15 | TRAF1 |
| PAG1 | PRDM1 |
| STXBP2 | PITHD1 |
| IL4R | SEC61A1 |
| APBB1IP | WNK1 |
| CAPZB | CCAR1 |
| CST7 | MPC1 |
| NRDC | MRPS35 |
| MKNK1 | CS |
| CEACAM1 | EIF4B |
| APLP2 | RPL18 |
| CD59 | U2AF2 |
| CD82 | RNF4 |
| SLC25A24 | SUGP2 |
| DNAJA1 | BTBD1 |
| CHMP5 | DGKA |
| NME8 | KARS1 |
| LAT2 | NDUFB4 |
| FTL | PRKCQ |
| PGS1 | SMARCD1 |
| LPCAT2 | CD84 |
| EDEM2 | ZBTB11 |
| FKBP1A | THUMPD1 |
| XRN2 | TRAM1 |
| PXN | IARS2 |
| GRAMD1A | CBFB |
| IRAK3 | RASSF1 |
| NLRC4 | OTUD5 |
| HAUS4 | RASGRP2 |
| MYL6 | NUCKS1 |
| FKBP5 | CLEC2D |
| SRPK1 | RORA |
| JAK2 | ATP1B3 |
| SH3GLB1 | GBA2 |
| GADD45B | PABPC1 |
| CYTH4 | MGAT4A |
| LGALS1 | TRIB2 |
| FBXO7 | DAZAP1 |
| HMOX1 | PTPN18 |
| TSPO | SMC1A |
| ASCC2 | NFATC3 |
| MTMR3 | ACADVL |
| KIAA0930 | GSDMB |
| NCF4 | ST6GAL1 |
| PRMT5 | TBX21 |
| SOS2 | MRPS34 |
| PYGL | ATP2A3 |
| TMED8 | LMAN1 |
| RIN3 | IPCEF1 |
| DHRS7 | TXK |
| GMPR2 | SLC25A3 |
| MMP9 | ARHGEF1 |
| TPD52L2 | TOP2B |
| DNAJC5 | SNRPA |
| CTSZ | ARAF |
| SIRPB1 | PPP2R5C |
| HCK | P2RY10 |
| CHMP4B | ITM2A |
| DNTTIP1 | VDAC3 |
| APMAP | PCM1 |
| VAPA | SDF4 |
| MYL12A | SAR1A |
| TLR8 | FDFT1 |
| PLP2 | SMARCA2 |
| BMX | CLDND1 |
| PGK1 | TCF7 |
| TIMP1 | TRAF5 |
| TNFSF13B | XPO1 |
| DNAJC3 | CYLD |
| OLFM4 | RPS5 |
| MAPK3 | STARD7 |
| LYRM1 | NOA1 |
| USB1 | GSTP1 |
| COTL1 | WBP11 |
| PYCARD | TXLNA |
| AQP9 | HADHA |
| LACTB | WDFY1 |
| CPQ | AKR1B1 |
| GSR | RRN3 |
| ASAH1 | NFX1 |
| LYL1 | TXLNG |
| RETN | HUWE1 |
| PLIN3 | SRRT |
| SIGLEC5 | PSMC5 |
| RAB3D | NOP14 |
| JAK3 | ADD1 |
| NAMPT | SF3B2 |
| IMPDH1 | DDX18 |
| BAG1 | KHSRP |
| UBE2R2 | ANKRD10 |
| ERLIN1 | MAVS |
| VSIR | DYNLL1 |
| MICU1 | SNX5 |
| P2RX1 | SIRPG |
| DUSP3 | ANAPC5 |
| MAP2K6 | SLC23A2 |
| WSB1 | TMEM230 |
| CHIC2 | PEBP1 |
| KLHL2 | ERP29 |
| BST1 | FUS |
| HTATIP2 | IGBP1 |
| CTSC | FXYD5 |
| EHD1 | GANAB |
| MS4A6A | DDX24 |
| MS4A4A | ARHGAP4 |
| ST3GAL4 | CCNK |
| RNF141 | NUDC |
| SELPLG | PDCD7 |
| LIN7A | PABPC4 |
| LTA4H | PDPR |
| ARPC3 | GLG1 |
| MANSC1 | ITGA6 |
| ALDH2 | SEL1L3 |
| ARHGDIB | CDV3 |
| GAPDH | ALKBH5 |
| LPCAT3 | NLRP1 |
| TDP2 | OSGEP |
| SLC26A8 | SUPT16H |
| MAPK14 | EZR |
| FBXO9 | DPYSL2 |
| TREML2 | ADA2 |
| VNN1 | ARCN1 |
| VNN2 | PSMD5 |
| SNX3 | BTAF1 |
| CCND3 | HSP90AB1 |
| RNF130 | SIRT1 |
| BTNL8 | ABLIM1 |
| LMNB1 | PSMD8 |
| ATP6V0E1 | CIRBP |
| HRH2 | HNRNPM |
| BCL6 | NDUFB7 |
| PDCD10 | CABIN1 |
| PFKFB4 | SF3A1 |
| GNAI2 | MFNG |
| EIF1B | LGALS2 |
| ACTR3 | PIK3IP1 |
| SF3B6 | TFIP11 |
| GCA | EIF3L |
| RTN4 | SNU13 |
| GALNT3 | TCF20 |
| IL1R2 | TOMM22 |
| IL1R1 | XBP1 |
| IL18R1 | RTCB |
| IL18RAP | POLDIP3 |
| QPCT | PPP6R2 |
| PLEK | SUN2 |
| ARID3A | CBX7 |
| QSOX1 | TXN2 |
| CAPZA1 | GRAP2 |
| RHOU | EIF3D |
| NCF2 | ST13 |
| GADD45A | IL2RB |
| RGS2 | CERK |
| PHTF1 | FKBP3 |
| CD58 | GZMH |
| SIPA1L2 | GZMB |
| PADI2 | TIMM9 |
| CTBS | AHSA1 |
| ECE1 | SRSF5 |
| RPS6KA1 | TCL1A |
| CTSD | PPP4R3A |
| MMP8 | YY1 |
| ATF6 | ACIN1 |
| KLF7 | APEX1 |
| RAB32 | PABPN1 |
| RNF146 | PNN |
| ARG1 | PCIF1 |
| FBXL5 | PYGB |
| MYL12B | NDRG3 |
| NDUFB3 | SLA2 |
| UBE2B | NFATC2 |
| RAD23B | ADNP |
| HSDL2 | PRPF6 |
| CSF3R | GID8 |
| IRF2BPL | CDC25B |
| DUSP1 | TM9SF4 |
| PCMT1 | SAMHD1 |
| TASL | NOP56 |
| CYSTM1 | IDH3B |
| FAM53C | CST3 |
| PTK2B | PPP1R16B |
| TRIM25 | USP14 |
| SCPEP1 | RNMT |
| PLBD1 | RNF125 |
| CSTA | PRPS2 |
| CAT | RBBP7 |
| TNFSF10 | PIM2 |
| GLIPR2 | SMS |
| SRGN | HTATSF1 |
| OPTN | CD40LG |
| NFE2 | MAGED2 |
| NMI | RBM3 |
| TNFAIP6 | PBDC1 |
| KCNJ2 | BEX4 |
| PFKFB2 | STK24 |
| SLPI | KPNA3 |
| ATP5F1E | RGCC |
| NQO2 | LONP2 |
| NDUFA1 | ARL2BP |
| MBOAT7 | CTCF |
| IL1B | SLC7A6 |
| TNFSF14 | GGA2 |
| VASP | HMOX2 |
| PSMF1 | RBL2 |
| S1PR4 | MAZ |
| FFAR2 | CDIPT |
| HNRNPH2 | HERC1 |
| ADGRE2 | EIF3J |
| HIP1 | IKBKB |
| POR | EEF1D |
| STYXL1 | LEPROTL1 |
| ZFP36 | MAN2B1 |
| TPST2 | MAP4K1 |
| RAC2 | HNRNPL |
| LRRC4 | SNRNP70 |
| CDKN2D | CD37 |
| SIGLEC9 | SF3A2 |
| SAT1 | FCER2 |
| COLGALT1 | TLE5 |
| MLLT1 | C19orf53 |
| CHMP2A | IL27RA |
| GMFG | PPP6R1 |
| THEMIS2 | RASAL3 |
| LILRB2 | URI1 |
| ATP6V1E1 | RPS16 |
| SH3BGRL | FBL |
| CAP1 | PRKD2 |
| SH3BP5 | HNRNPUL1 |
| RARA | CD79A |
| CHSY1 | RPS19 |
| EMILIN2 | NOP53 |
| CARD6 | NKG7 |
| H3-3B | CD33 |
| SDF2 | CDC37 |
| FLOT2 | KDELR1 |
| ARHGEF11 | CAPS |
| PPP1R3D | DNASE2 |
| POMP | RPL18A |
| ALOX5AP | ARRDC2 |
| RTN3 | USF2 |
| AMPD3 | KXD1 |
| SORT1 | CBLL1 |
| LAMTOR5 | OGDH |
| LDHA | H2AZ2 |
| PHC2 | LFNG |
| DSC2 | CPVL |
| TMEM165 | CASP2 |
| OSTF1 | CHCHD2 |
| ANXA1 | HSPB1 |
| AGTPBP1 | PDAP1 |
| CD36 | EIF3B |
| AKIRIN2 | TSPAN13 |
| PHF21A | GIMAP2 |
| CD63 | BLVRA |
| ACVR1B | TMEM248 |
| OS9 | TRIM14 |
| CD164 | AKNA |
| MICAL1 | EDF1 |
| STX11 | RAPGEF1 |
| DYSF | PDLIM1 |
| GNS | GATA3 |
| NIBAN1 | EIF3A |
| CAB39 | DDX50 |
| CKAP4 | SPOCK2 |
| PLXNC1 | CCSER2 |
| DRAM1 | RPL28 |
| LCP1 | RPL19 |
| RAC1 | UBTF |
| CIDEB | C1QBP |
| IL1RN | SMARCD2 |
| TXN | ICAM2 |
| TLR4 | CYTH1 |
| FLOT1 | ABI3 |
| IER3 | EZH1 |
| RNF144B | PPP1R9B |
| SDCBP | LUC7L3 |
| CASP1 | SLC9A3R1 |
| CASP5 | SUPT6H |
| SQOR | TBC1D9 |
| CYP1B1 | ZNF330 |
| RAB1A | DHX15 |
| EXOC6 | MTCH2 |
| IDH1 | FNBP4 |
| SLC40A1 | HSPA8 |
| SLC49A4 | LPXN |
| GPAT3 | OSBP |
| ANXA3 | CPT1A |
| PAPSS1 | PRPF19 |
| FGD4 | TMEM109 |
| C1RL | IL10RA |
| GLTP | CD5 |
| GPR84 | C11orf21 |
| BNIP2 | AIP |
| ANP32A | MLEC |
| PSTPIP1 | ATP5F1B |
| BCL2A1 | PTGES3 |
| MCTP2 | METAP2 |
| FURIN | ELK3 |
| IGSF6 | SH2B3 |
| CMTM2 | CREBL2 |
| IMPA2 | EIF2B1 |
| ARRB2 | SLC38A1 |
| SLC16A3 | COPZ1 |
| NARF | CPSF6 |
| SECTM1 | GNPTAB |
| VAV1 | CMAS |
| IFNAR1 | C2CD5 |
| NLRP12 | BTN3A3 |
| EFHD2 | ASF1A |
| PGD | SRSF3 |
| C1orf162 | MRPL18 |
| CD53 | MCM3 |
| CREG1 | BACH2 |
| COP1 | PHF1 |
| FCGR2A | CUTA |
| S100A8 | PRPF4B |
| RIT1 | TENT5A |
| GALNT2 | LY86 |
| CNIH4 | HARS2 |
| MBOAT2 | MAN2A1 |
| ARL8A | HSPA9 |
| RALB | GZMK |
| CSRNP1 | IK |
| IQSEC1 | ITK |
| ATG3 | CNOT6 |
| PLAC8 | CCNG1 |
| TIFA | TARS1 |
| ROPN1L | LNPEP |
| OTULINL | PPWD1 |
| FEM1C | TCERG1 |
| TNIP1 | CD86 |
| ZMAT2 | NCBP2 |
| DOK3 | SSR3 |
| PPP1R18 | NKTR |
| DYNLT1 | EIF4G1 |
| CREB5 | SPCS1 |
| SLC12A9 | DGUOK |
| SLC25A37 | NCL |
| UGCG | ZAP70 |
| STOM | ITGA4 |
| GSN | SNX17 |
| LCN2 | PPM1G |
| MSRB2 | RPS15 |
| NRBF2 | SPTBN1 |
| GSTO1 | AUP1 |
| ITPRIP | LANCL1 |
| RGS10 | GLS |
| BTBD10 | CCT4 |
| ADM | GNLY |
| LAMTOR1 | CNPPD1 |
| FERMT3 | HDLBP |
| FCGR1A | PPP1R7 |
| TMCO3 | ID2 |
| RILPL2 | ODC1 |
| UBC | BIRC6 |
| ADAM8 | GORASP2 |
| ACSL1 | CEBPZ |
| GLT1D1 | DARS1 |
| ARL11 | SRSF7 |
| PSTPIP2 | TIA1 |
| ANKRD22 | RALGPS2 |
| HHEX | RPL22 |
| RBMS1 | PARK7 |
| CYRIB | AMPD2 |
| RPIA | SRSF4 |
| LY96 | HDAC1 |
| ATP6V1C1 | SFPQ |
| PIP4P2 | MEF2D |
| SLC25A28 | BCAS2 |
| SAMSN1 | SRSF11 |
| PIK3AP1 | CD2 |
| VSIG4 | GNPAT |
| FAM126B | SLAMF1 |
| SLA | RPF1 |
| GNAQ | RBBP5 |
| BATF | RAB29 |
| TDRD9 | PRPF3 |
| ST3GAL2 | APH1A |
| KCNJ15 | PRDX1 |
| ETS2 | PRRC2C |
| SLC37A3 | RCAN3 |
| H2BC5 | RPA2 |
| B4GALT5 | PPP1R8 |
| CDA | ESYT2 |
| PINK1 | HMGN3 |
| FCER1G | SGK1 |
| IFNGR2 | VAMP8 |
| PSMD4 | RPN2 |
| RGL4 | KLF12 |
| ATP6V0D1 | CCND2 |
| ZYX | SET |
| G6PD | DLST |
| CSTB | RBM25 |
| ITGB2 | SUPT7L |
| SHKBP1 | NUP43 |
| ZBTB7B | TCP1 |
| SLC25A44 | UFM1 |
| NBEAL2 | HSPH1 |
| HK3 | TGFBI |
| PCYT1A | TMPO |
| CXCL16 | TNFRSF10B |
| ASGR2 | PPP3CC |
| LAPTM5 | TARDBP |
| ALPL | SPOP |
| ARPC5 | FAM117A |
| NLRP3 | TMEM131L |
| NCSTN | ADCY7 |
| H3-3A | KHDRBS1 |
| TNFAIP8L2 | POLR3GL |
| RNF149 | PDS5A |
| CDC42EP3 | TMEM156 |
| S100A11 | GTF3A |
| S100A9 | LAX1 |
| S100A12 | KIAA1191 |
| TGFA | CD244 |
| EIF4E3 | RBBP6 |
| PROK2 | TRMT13 |
| IGFBP7 | SMU1 |
| CXCR1 | CLTA |
| ARPC2 | HVCN1 |
| MNDA | WWP1 |
| AIM2 | PKN1 |
| PPP4R2 | TRIR |
| WDFY3 | ZC3H13 |
| TIPARP | NCOA5 |
| RBM47 | PLCG1 |
| CXCL1 | SRSF6 |
| GYG1 | CSE1L |
| PLB1 | RNF114 |
| CCR1 | DDX27 |
| TKT | BCAS4 |
| PRKCD | NAGK |
| ZDHHC19 | TRERF1 |
| S100P | RRP36 |
| PPM1M | SNRPC |
| C4orf3 | TBCC |
| HMGB2 | KLHDC3 |
| ANXA5 | GLO1 |
| HPGD | AHNAK |
| GASK1B | CNOT1 |
| CGAS | GOT2 |
| BRI3 | GPR18 |
| CTSB | RNF113A |
| UBAP1 | SEPTIN6 |
| SYK | DMAC2L |
| NFIL3 | PTGER2 |
| TMEM71 | TTF1 |
| NDUFB6 | PSD4 |
| PDZD8 | GTF2F1 |
| HPRT1 | THOC2 |
| ARHGEF40 | CENPB |
| FRAT1 | DSTN |
| SMCO4 | SNRPB2 |
| CLEC4D | HNRNPR |
| ANPEP | PLAGL2 |
| CCNDBP1 | PSMB2 |
| CA4 | UBA2 |
| TPM4 | HCST |
| MIDN | CCR7 |
| DHRS13 | IRF3 |
| TUBA1A | SBDS |
| LAIR1 | DNAJC8 |
| CD300C | UXT |
| FTH1 | ZBTB1 |
| FADD | SGPP1 |
| RBPJ | PLEKHG3 |
| MMADHC | FAM78A |
| PXK | CANX |
| MLKL | BCL11B |
| RAB31 | HELB |
| SIGLEC7 | DYRK2 |
| XPO6 | HP1BP3 |
| RGS14 | KLF2 |
| PGM2 | ECHS1 |
| RNASE2 | ATF4 |
| CSGALNACT2 | APOL3 |
| ITGAM | ATP6V1F |
| CD14 | ORMDL1 |
| PFKFB3 | PSMG2 |
| EMB | VPS13C |
| GPR27 | SUMF2 |
| TSEN34 | DCTD |
| OSCAR | CCNT1 |
| CEACAM3 | KRI1 |
| FPR2 | ILF3 |
| FPR1 | ARHGEF6 |
| C1GALT1C1 | PHF10 |
| TRIM8 | PRKCSH |
| JUNB | CDC16 |
| LRG1 | SAFB2 |
| PGAM1 | RPL36 |
| RGS19 | JUND |
| RASGRP4 | ATXN10 |
| C3AR1 | ZNF337 |
| PTEN | ADRM1 |
| CEBPB | TRIM28 |
| GNB2 | EIF2S3 |
| SMPDL3A | ATP5IF1 |
| CFL1 | EIF3G |
| MYD88 | DKC1 |
| HPSE | MPP1 |
| HSPA6 | NOL11 |
| MTX1 | COX4I1 |
| PPP1R3B | MRPS25 |
| TRIB1 | RFTN1 |
| C1QB | NAPSB |
| C1QA | ACLY |
| EHBP1L1 | DIAPH1 |
| SULT1B1 | NDFIP1 |
| AGFG1 | UBE2D2 |
| STAT5B | PEX11B |
| GPR160 | SNRPA1 |
| SLCO4C1 | DNAJB1 |
| CEP19 | NUP210 |
| TLR1 | FCRLA |
| ADGRE1 | TIMM10B |
| TP53I11 | IMMT |
| LRRC25 | RAN |
| KCNE3 | RAP1GAP2 |
| B3GNT5 | RPA1 |
| BASP1 | UBE2G1 |
| RHOG | UTP3 |
| TALDO1 | ZRANB2 |
| CD163 | EIF5A |
| GBA | PCNA |
| IL17RA | PTPRA |
| AGTRAP | DAP3 |
| AP3S1 | KHDC4 |
| GRB2 | FCRL2 |
| GRINA | DCAF8 |
| DPY19L3 | USPL1 |
| CYBC1 | MPRIP |
| SNX18 | SRRM1 |
| RAD23A | CSNK1G2 |
| NSUN7 | PLAAT4 |
| MAF1 | GIMAP6 |
| SEPHS2 | GIMAP4 |
| FUT7 | SWAP70 |
| H2AC6 | DGLUCY |
| H2BC4 | CD180 |
| PCGF5 | VHL |
| YOD1 | BHLHE40 |
| ZDHHC20 | YWHAQ |
| ST20 | IL6ST |
| FRAT2 | NARS1 |
| ZNF467 | RBM17 |
| RNF135 | KLRD1 |
| P2RY13 | STK26 |
| NOP10 | PUM1 |
| FES | PRPF38A |
| LIMK2 | ARGLU1 |
| HCAR2 | TPP2 |
| C16orf72 | ETS1 |
| ADGRG3 | UBQLN1 |
| MCEMP1 | TAOK3 |
| PTTG1IP | DMTF1 |
| ZNF438 | TES |
| UPP1 | MDFIC |
| KREMEN1 | SYNCRIP |
| TREML3P | NAT10 |
| SOCS3 | CAPRIN1 |
| H2BC21 | ATP5MC2 |
| APOBR | TESPA1 |
| IFITM2 | TFCP2 |
| UBALD2 | HNRNPA1 |
| EVI2B | CCT7 |
| CD300LF | DHX9 |
| MTARC1 | DOCK10 |
| FCAR | MRPS9 |
| UBE2H | ISCU |
| LILRA5 | PWP1 |
| CHP1 | VPS36 |
| TLR5 | TBC1D4 |
| H1-2 | RPL13AP25 |
| PEAK3 | KDELR2 |
| PLSCR1 | DDX56 |
| SELL | CCM2 |
| RALGAPA2 | MYO1G |
| MTF1 | DCAF7 |
| LITAF | LIMD2 |
| TMEM120A | EPRS1 |
| ANKRD34B | BIN1 |
| JPT1 | CDK9 |
| S100A4 | PSMB7 |
| SEMA4A | ANP32B |
| FAM217B | DERL1 |
| CD55 | MYC |
| SDHAF3 | SIT1 |
| TECPR2 | IRF4 |
| GM2A | HMGA1 |
| ARID5A | MTCH1 |
| PDLIM7 | FGFBP2 |
| CASP4 | NUMA1 |
| METTL9 | CREBZF |
| SLC22A4 | TGS1 |
| SERPINA1 | SLTM |
| LRP10 | RPLP1 |
| C5AR1 | PLCB2 |
| SGTB | ATRAID |
| H2BC12 | ATIC |
| S100A6 | STAT4 |
| FCGR1B | SSB |
| SIRPA | FAM117B |
| GRK6 | HNRNPD |
| TFDP1 | SEC31A |
| HSD17B11 | NAAA |
| UBL5 | G3BP2 |
| PIM3 | CNOT6L |
| ASPH | LEF1 |
| TXNRD1 | GUCD1 |
| BAZ1A | PARVG |
| F5 | KLRG1 |
| MSRB1 | VAMP1 |
| GK | CD27 |
| UBE2J1 | SMARCC2 |
| SELENOT | ITGB7 |
| CES1 | ESYT1 |
| R3HDM4 | RBM26 |
| CAPZA2 | CUL4A |
| RNY1P16 | DCAF5 |
| CR1 | MFAP1 |
| FCGR3A | UBE2Q2 |
| LINC01270 | SCAMP2 |
| ZDHHC18 | HAPLN3 |
| HSPA1B | UQCRC2 |
| HSPA1A | CDR2 |
| CARD16 | NLRC5 |
| AIF1 | IRF8 |
| LILRB3 | RPS2 |
| TBC1D8 | NDUFB10 |
| CD177 | TCF25 |
| PRR13 | NCOR1 |
| RNU6-196P | COPS3 |
| RNU6-1005P | NOB1 |
| SNORD89 | SKAP1 |
| SRA1 | SS18 |
| GPSM3 | SLC39A6 |
| CLIC1 | RMC1 |
| DDAH2 | SAT2 |
| LTB4R | TP53 |
| C19orf38 | ARHGDIA |
| FIS1 | WDR45B |
| PLIN5 | CBX4 |
| UBXN2B | RETREG3 |
| CKLF | CCDC97 |
| CEBPZOS | PGGHG |
| CEBPD | TMEM50B |
| UBA52 | APP |
| RNU4-62P | AKT1 |
| PELATON | EMP3 |
| C3orf86 | SAE1 |
| NAMPTP1 | PSMB6 |
| FTH1P2 | RPS11 |
| NFAM1 | NOSIP |
| RNU7-45P | SYTL1 |
| LILRA2 | SERBP1 |
| ARPC4 | PRKACB |
| PLEKHO2 | ALDH9A1 |
| ARFGAP3 | UFC1 |
| RPL7P18 | PFDN2 |
| LILRA6 | RFX5 |
| CEBPA | PI4KB |
| NAIP | ANP32E |
| H3P14 | MRPL9 |
| RNU6-917P | ARNT |
| RNU6-1003P | POGZ |
| LYN | GOLPH3L |
| SIGLEC14 | ATP8B2 |
| AP5B1 | UBAP2L |
| HCAR3 | HAX1 |
| HP | ILF2 |
| MGAM | TTC13 |
| MGAM2 | ACP1 |
| CLEC5A | SDE2 |
| CCPG1 | ITPKB |
| MILR1 | PYCR2 |
| NATD1 | PDIA6 |
| MIR223HG | EML4 |
| MIR7848 | CIAO1 |
| MARCKS | SNRNP200 |
| PSMB3 | TEX261 |
|  | SLC20A1 |
|  | HSPD1 |
|  | CNOT9 |
|  | SLC25A38 |
|  | GOLGA4 |
|  | ABHD10 |
|  | EIF2A |
|  | LYAR |
|  | NDUFS6 |
|  | PIK3R1 |
|  | TNFAIP8 |
|  | DDX46 |
|  | G3BP1 |
|  | NHP2 |
|  | HIGD2A |
|  | FAM193B |
|  | TMEM181 |
|  | RPL32P18 |
|  | MDH2 |
|  | CCT6A |
|  | GIGYF1 |
|  | TRIM4 |
|  | MEPCE |
|  | GPR174 |
|  | NONO |
|  | OGT |
|  | IL2RG |
|  | RBMX |
|  | RPL10 |
|  | DOK2 |
|  | CHMP7 |
|  | PLPBP |
|  | PLPP5 |
|  | NSD3 |
|  | MTDH |
|  | UHRF2 |
|  | POLE3 |
|  | SURF4 |
|  | SURF1 |
|  | RPL7A |
|  | RSU1 |
|  | DGKZ |
|  | EIF3M |
|  | SSRP1 |
|  | ARFGAP2 |
|  | CELF1 |
|  | SESN3 |
|  | NPAT |
|  | ATM |
|  | MTA2 |
|  | LSM14B |
|  | YTHDF1 |
|  | KLRF1 |
|  | ITGB1 |
|  | SAP18 |
|  | PDCD4 |
|  | PIP4K2A |
|  | FOXO1 |
|  | VPS26B |
|  | TIAL1 |
|  | RABGAP1L |
|  | FAM168B |
|  | ATP5F1A |
|  | UHMK1 |
|  | CWF19L2 |
|  | CAMK4 |
|  | ZFP36L2 |
|  | TMEM123 |
|  | HNRNPDL |
|  | UTRN |
|  | BANK1 |
|  | SMARCA5 |
|  | HNRNPU |
|  | RANBP2 |
|  | TMEM87B |
|  | CD96 |
|  | UBP1 |
|  | CD8A |
|  | GRAP |
|  | TBRG1 |
|  | TOMM70 |
|  | PITPNC1 |
|  | MIA3 |
|  | BUB3 |
|  | XPC |
|  | CCDC174 |
|  | PLCL2 |
|  | KLF10 |
|  | GTF3C6 |
|  | NIFK |
|  | OXA1L |
|  | LARP1 |
|  | PDIA4 |
|  | RNF20 |
|  | DCK |
|  | DRAM2 |
|  | USP16 |
|  | CCT8 |
|  | SCAF4 |
|  | PTDSS1 |
|  | PABIR2 |
|  | HK1 |
|  | MS4A1 |
|  | EIF4A2 |
|  | NECAP2 |
|  | SSBP3 |
|  | SUSD3 |
|  | TNFRSF14 |
|  | RER1 |
|  | SKI |
|  | LDLRAP1 |
|  | PAFAH2 |
|  | UBXN11 |
|  | MITD1 |
|  | EIF5B |
|  | CNOT11 |
|  | TMED4 |
|  | ELK4 |
|  | RNF166 |
|  | ZNF276 |
|  | CCAR2 |
|  | CDC42SE2 |
|  | EPB41 |
|  | PAXBP1 |
|  | SON |
|  | UBE2Z |
|  | ADPGK |
|  | PSMB4 |
|  | THEM4 |
|  | TMEM69 |
|  | EFCAB14 |
|  | CTBP1 |
|  | CCDC117 |
|  | TNFRSF13C |
|  | CALM3 |
|  | RRP1B |
|  | MCM3AP |
|  | PRMT2 |
|  | PCSK7 |
|  | SAFB |
|  | CD3G |
|  | CHTOP |
|  | UBE2Q1 |
|  | CRTC2 |
|  | CCR5 |
|  | FCRL3 |
|  | VPS28 |
|  | SQSTM1 |
|  | RPL8 |
|  | AP2M1 |
|  | IKZF3 |
|  | SRSF2 |
|  | ZNF385A |
|  | ALOX15 |
|  | SCIMP |
|  | UBXN1 |
|  | NXF1 |
|  | RPL29 |
|  | CMPK1 |
|  | USP24 |
|  | NOL9 |
|  | RBBP4 |
|  | USP1 |
|  | FUBP1 |
|  | PEA15 |
|  | SLAMF6 |
|  | DENND2D |
|  | IER5 |
|  | CNST |
|  | FCMR |
|  | CAPN2 |
|  | SLC66A3 |
|  | COQ8A |
|  | VPS72 |
|  | IWS1 |
|  | DCAF16 |
|  | PBXIP1 |
|  | PYGO2 |
|  | ATP1A1 |
|  | CCT3 |
|  | SSR2 |
|  | EOMES |
|  | ANKZF1 |
|  | TRAT1 |
|  | STT3B |
|  | FCRL1 |
|  | PYHIN1 |
|  | SNHG16 |
|  | PPM1K |
|  | U2SURP |
|  | TTC14 |
|  | WDR43 |
|  | GNL3 |
|  | PBRM1 |
|  | TASOR |
|  | ARHGEF3 |
|  | SLBP |
|  | RNF168 |
|  | METAP1 |
|  | PGRMC2 |
|  | WDR82 |
|  | ABCE1 |
|  | SERINC5 |
|  | ERAP1 |
|  | ERAP2 |
|  | NSA2 |
|  | CITED2 |
|  | TAGAP |
|  | WASHC5 |
|  | SNAPC3 |
|  | PSIP1 |
|  | ALDH1A1 |
|  | AQP3 |
|  | INTS6L |
|  | NEMF |
|  | ARF6 |
|  | VDAC2 |
|  | DDX21 |
|  | PSMC3 |
|  | TC2N |
|  | MOAP1 |
|  | TAF1D |
|  | BAG5 |
|  | API5 |
|  | NOLC1 |
|  | CCT2 |
|  | ANAPC16 |
|  | TRIM44 |
|  | LEO1 |
|  | MCM7 |
|  | HSP90B1 |
|  | SLFN5 |
|  | PPIB |
|  | FAM111A |
|  | TMEM170A |
|  | TERF2IP |
|  | VPS39 |
|  | PIP4K2C |
|  | MS4A7 |
|  | MS4A14 |
|  | SMAD3 |
|  | MAPRE2 |
|  | MARS1 |
|  | PDIA3 |
|  | NUDT21 |
|  | FAM102A |
|  | SP2 |
|  | CD3D |
|  | STIM1 |
|  | GPX4 |
|  | NIBAN3 |
|  | ANKRD11 |
|  | RPL13 |
|  | LENG8 |
|  | ZNF146 |
|  | EEF2 |
|  | ZNF83 |
|  | PRDX2 |
|  | ATP5PD |
|  | TMC8 |
|  | SRRM2 |
|  | NLRC3 |
|  | POLR2G |
|  | SF1 |
|  | COPS6 |
|  | MAPK1IP1L |
|  | TTC39C |
|  | IRF2BP2 |
|  | MGAT2 |
|  | CX3CR1 |
|  | SEPTIN2 |
|  | RHOH |
|  | STIP1 |
|  | ATXN2L |
|  | IL7R |
|  | AHCYL1 |
|  | CHTF8 |
|  | DERPC |
|  | ANKRD49 |
|  | MAT2A |
|  | INPP5D |
|  | HNRNPH1 |
|  | SLC25A6 |
|  | ADRB2 |
|  | PLEKHA2 |
|  | GPR183 |
|  | HNRNPF |
|  | TRIM56 |
|  | SF3B5 |
|  | CHD3 |
|  | UBE2E1 |
|  | HNRNPA3 |
|  | CRTAP |
|  | TMED10 |
|  | PA2G4 |
|  | SERPINB9 |
|  | HSPA4 |
|  | RNF34 |
|  | TRABD |
|  | MTSS1 |
|  | RPS9 |
|  | PAQR8 |
|  | S1PR1 |
|  | GIMAP8 |
|  | RSL1D1 |
|  | LRRC8D |
|  | PTGER4 |
|  | CLSTN1 |
|  | BPTF |
|  | ATF7IP |
|  | BCL2 |
|  | EXOSC10 |
|  | PRNP |
|  | MAL |
|  | QARS1 |
|  | ORMDL3 |
|  | MALT1 |
|  | ISG20 |
|  | SUCLG2 |
|  | PPP1CA |
|  | CTSW |
|  | RASGRP1 |
|  | WASHC2C |
|  | THEMIS |
|  | SLFN11 |
|  | RAB37 |
|  | RPL38 |
|  | RELA |
|  | TRMT112 |
|  | LRRN3 |
|  | PARP15 |
|  | AHSA2P |
|  | IQCB1 |
|  | SMARCC1 |
|  | ZNF622 |
|  | CHD2 |
|  | EIF1AX |
|  | TOMM20 |
|  | CD7 |
|  | EIF1 |
|  | PHC3 |
|  | PRPF8 |
|  | DDX23 |
|  | ZBTB4 |
|  | ZNF266 |
|  | NR1D2 |
|  | SRP72 |
|  | GPR171 |
|  | PACS1 |
|  | MARCKSL1 |
|  | PSMD2 |
|  | GOLGA8A |
|  | PHYKPL |
|  | BANF1 |
|  | TMEM9B |
|  | TBC1D10C |
|  | UCP2 |
|  | C11orf68 |
|  | CTDNEP1 |
|  | CLK2 |
|  | NFATC2IP |
|  | SEC24C |
|  | ARIH2 |
|  | ZBTB33 |
|  | RPLP2 |
|  | CSTF2T |
|  | HNRNPA0 |
|  | UBE2N |
|  | BET1L |
|  | IMP3 |
|  | IMPDH2 |
|  | WDR6 |
|  | CD28 |
|  | COX5A |
|  | ZHX2 |
|  | CD300LB |
|  | ZBTB7A |
|  | TUFM |
|  | CTC1 |
|  | EIF3K |
|  | MRFAP1L1 |
|  | RCC2 |
|  | FUCA1 |
|  | ZNF664 |
|  | CALR |
|  | HLA-DQB1 |
|  | FCER1A |
|  | PCED1B |
|  | SERTAD2 |
|  | R3HDM2 |
|  | C14orf119 |
|  | SEPTIN1 |
|  | ARHGAP45 |
|  | PRF1 |
|  | S1PR5 |
|  | PITPNB |
|  | FCRL6 |
|  | POLR2A |
|  | RAP2B |
|  | ZNF101 |
|  | IDH2 |
|  | TMEM259 |
|  | MRPL41 |
|  | P2RY8 |
|  | UBA7 |
|  | SATB1 |
|  | CSF1R |
|  | TTC3 |
|  | LCK |
|  | RBM10 |
|  | SRPRA |
|  | EWSR1 |
|  | HMGN4 |
|  | SMDT1 |
|  | KIAA2026 |
|  | SF3A3 |
|  | COA5 |
|  | HMCES |
|  | CCR3 |
|  | TRIM52 |
|  | CCR4 |
|  | TOB2 |
|  | SH2D1A |
|  | PRKX |
|  | PTP4A2 |
|  | UQCR10 |
|  | PPP1R2 |
|  | TSPYL2 |
|  | MAML2 |
|  | STING1 |
|  | NELL2 |
|  | SEPTIN9 |
|  | UBE2G2 |
|  | H1-10 |
|  | SUMO3 |
|  | AP3M1 |
|  | CIB1 |
|  | SIGIRR |
|  | RAB11B |
|  | PRPF39 |
|  | IL3RA |
|  | SP140L |
|  | METTL7A |
|  | TMEM179B |
|  | UBE2L3 |
|  | MYBL1 |
|  | IKZF1 |
|  | RASA3 |
|  | CENATAC |
|  | BCL9L |
|  | BTLA |
|  | PPP1CC |
|  | BTN3A2 |
|  | GPATCH8 |
|  | ZFP91 |
|  | HEXIM1 |
|  | TRABD2A |
|  | NAP1L1 |
|  | FNBP1 |
|  | PTMA |
|  | USP7 |
|  | TMEM203 |
|  | TCEA1 |
|  | SECISBP2 |
|  | ARL4C |
|  | SRSF10 |
|  | RPL14 |
|  | SF3B3 |
|  | TSPYL1 |
|  | FAM53B |
|  | SLC35E2B |
|  | HLA-DRB1 |
|  | TMEM63A |
|  | MPHOSPH8 |
|  | TUBB |
|  | SUPT5H |
|  | GIMAP5 |
|  | EVL |
|  | XRCC6 |
|  | NACA |
|  | PLXNB2 |
|  | HSH2D |
|  | HLA-DQA1 |
|  | CD47 |
|  | ILRUN |
|  | FLNA |
|  | ANXA6 |
|  | PCBP2 |
|  | ACSL5 |
|  | SND1 |
|  | ZNF655 |
|  | GSTK1 |
|  | HNRNPAB |
|  | SPN |
|  | MPEG1 |
|  | SPTAN1 |
|  | S100A10 |
|  | RPL37A |
|  | RPL12 |
|  | AKAP17A |
|  | RPS4X |
|  | ZNF770 |
|  | QRICH1 |
|  | DDX42 |
|  | RPL23A |
|  | STYX |
|  | CARD11 |
|  | SDAD1 |
|  | ZKSCAN8 |
|  | GET3 |
|  | YTHDF2 |
|  | HLA-DRB5 |
|  | DDX39B |
|  | SH2D1B |
|  | TLK1 |
|  | STK39 |
|  | CALM1 |
|  | LDB1 |
|  | CTR9 |
|  | FOXJ3 |
|  | CD247 |
|  | HMGN2 |
|  | DENND4B |
|  | CD3E |
|  | OSTC |
|  | SMC5 |
|  | APRT |
|  | NAGA |
|  | SMG5 |
|  | MIRLET7D |
|  | RNA5SP383 |
|  | SNORA33 |
|  | SNORD14E |
|  | SNORD6 |
|  | PCMTD2 |
|  | TMEM273 |
|  | DAXX |
|  | SMIM40 |
|  | RING1 |
|  | RXRB |
|  | HLA-DOA |
|  | BRD2 |
|  | HLA-DMA |
|  | HLA-DRA |
|  | RNF5 |
|  | LSM2 |
|  | BAG6 |
|  | PRRC2A |
|  | MICB |
|  | DHX16 |
|  | MRPS18B |
|  | ABCF1 |
|  | RACK1 |
|  | HLA-F |
|  | PDE7A |
|  | IPO7 |
|  | NAP1L4 |
|  | HMGN1 |
|  | DENND1C |
|  | ARRDC5 |
|  | HCP5 |
|  | RAB12 |
|  | SNORD59A |
|  | MIR570 |
|  | MIR573 |
|  | MIR186 |
|  | MIR590 |
|  | TSN |
|  | IGKJ5 |
|  | IGKJ4 |
|  | IGKJ3 |
|  | IGKJ1 |
|  | TRGC1 |
|  | TRBV6-1 |
|  | TRBV4-1 |
|  | TRBV9 |
|  | TRBV6-5 |
|  | TRBV6-6 |
|  | TRBV5-1 |
|  | TRBV4-2 |
|  | TRBV19 |
|  | TRBV20-1 |
|  | TRBV23-1 |
|  | TRBV24-1 |
|  | TRBC1 |
|  | TRBV27 |
|  | TRBV28 |
|  | TRBJ2-1 |
|  | TRBJ2-2 |
|  | TRBJ2-2P |
|  | TRBJ2-3 |
|  | TRBC2 |
|  | TRAV4 |
|  | TRAV5 |
|  | TRAV6 |
|  | TRAV12-1 |
|  | TRAV8-3 |
|  | TRAV13-1 |
|  | TRAV12-2 |
|  | TRAV13-2 |
|  | TRAV14DV4 |
|  | TRAV9-2 |
|  | TRAV12-3 |
|  | TRAV16 |
|  | TRAV17 |
|  | TRAV19 |
|  | TRAV20 |
|  | TRAV21 |
|  | TRAV23DV6 |
|  | TRDV1 |
|  | TRAV24 |
|  | TRAV25 |
|  | TRAV26-1 |
|  | TRAV29DV5 |
|  | TRAV35 |
|  | TRAV41 |
|  | TRDJ1 |
|  | TRAJ45 |
|  | TRAJ42 |
|  | TRAJ40 |
|  | TRAJ39 |
|  | TRAJ35 |
|  | TRAJ34 |
|  | TRAJ33 |
|  | TRAJ31 |
|  | TRAJ23 |
|  | TRAJ21 |
|  | TRAJ19 |
|  | TRAJ18 |
|  | TRAJ17 |
|  | TRAJ16 |
|  | TRAJ14 |
|  | TRAJ13 |
|  | TRAJ12 |
|  | TRAJ11 |
|  | TRAJ10 |
|  | TRAJ9 |
|  | TRAJ7 |
|  | TRAJ6 |
|  | TRAJ5 |
|  | TRAJ4 |
|  | TRAJ3 |
|  | TRAJ2 |
|  | TRAJ1 |
|  | IGHD |
|  | IGHM |
|  | IGHJ6 |
|  | SNORA26 |
|  | SCAF8 |
|  | GIMAP1 |
|  | VDAC1 |
|  | TMX2 |
|  | LBH |
|  | ATF6B |
|  | RPL7AP64 |
|  | AP1G2 |
|  | SMIM7 |
|  | PTMAP5 |
|  | HNRNPUL2 |
|  | PHB2 |
|  | RPL13P12 |
|  | GOLGA8B |
|  | TNFRSF25 |
|  | SLC35E2A |
|  | VAMP2 |
|  | SNORD99 |
|  | CCNL2 |
|  | SNORD19C |
|  | HLA-DPB1 |
|  | MIAT |
|  | NOL7 |
|  | TRBV2 |
|  | TRGC2 |
|  | LTB |
|  | XIST |
|  | HLA-DPA1 |
|  | RPL3P4 |
|  | TRBV29-1 |
|  | RPS28 |
|  | TRIM26 |
|  | RPL23AP42 |
|  | RPL13AP5 |
|  | IGHJ3P |
|  | TRBV3-1 |
|  | RNU7-57P |
|  | SNORD4A |
|  | APOBEC3G |
|  | IGHJ4 |
|  | RPS2P5 |
|  | HLA-DOB |
|  | RPL7AP6 |
|  | SDHAP2 |
|  | IGHJ5 |
|  | HLA-DMB |
|  | IGHJ3 |
|  | ZNF512 |
|  | DDOST |
|  | RBM12 |
|  | APOBEC3C |
|  | LINC00861 |
|  | RAD51-AS1 |
|  | SNHG6 |
|  | ZCCHC3 |
|  | OIP5-AS1 |
|  | PCED1B-AS1 |
|  | LINC00926 |
|  | CDK11B |
|  | MTATP6P1 |
|  | TRGV7 |
|  | CHCHD10 |
|  | RNU6-1053P |
|  | RNU6-322P |
|  | RNU4ATAC18P |
|  | RNU6-118P |
|  | ATXN7L3B |
|  | GVINP1 |
|  | EID1 |
|  | SNHG1 |
|  | TRAV1-2 |
|  | DENND11 |
|  | LSM14A |
|  | SRSF8 |
|  | DYNLL2 |
|  | MIR4537 |
|  | TSPOAP1-AS1 |
|  | RBM8A |
|  | TIMM23 |
|  | MIR4539 |
|  | MIR4645 |
|  | FMNL1-DT |
|  | SCARNA17 |
|  | ADGRE4P |
|  | TAF15 |
|  | CCL5 |
|  | LIX1L |
|  | RNU6-94P |
|  | LINC01215 |
|  | SNORD14A |
|  | NUDT3 |
|  | MLLT6 |
|  | SYNRG |
|  | TRBV10-3 |
|  | PIP4K2B |
|  | TRBV18 |
|  | TRAJ36 |
|  | TRBV15 |
|  | TRAC |
|  | TRBV7-9 |
|  | TRAJ37 |
|  | EBLN3P |
|  | TRBJ1-4 |
|  | TRBJ1-3 |
|  | TRBJ1-5 |
|  | TRBJ1-1 |
|  | TRBJ1-2 |
|  | TRBD1 |
|  | TRBJ1-6 |
|  | TRBV7-2 |
|  | MIR1244-2 |
