# Supplementary material for: Novel insight on marker genes and pathogenic peripheral neutrophil subtypes in acute pancreatitis
Source: Front Immunol. 2022 Aug 22;13:964622. doi: 10.3389/fimmu.2022.964622 (PMC9444397; doi:10.3389/fimmu.2022.964622)
Supplement: Supplementary file 7 [file Table_5.docx]

|  | baseMean | log2FoldChange | lfcSE | stat | pvalue | padj | sig |
| --- | --- | --- | --- | --- | --- | --- | --- |
| TK1 | 462.9778 | 3.131768 | 0.20789 | 15.06457 | 2.77E-51 | 4.65E-47 | up |
| PLK1 | 185.4731 | 3.80119 | 0.262521 | 14.47957 | 1.63E-47 | 1.37E-43 | up |
| RRM2 | 846.8545 | 3.743139 | 0.259132 | 14.44489 | 2.70E-47 | 1.51E-43 | up |
| IFI27 | 3925.889 | 8.640079 | 0.610031 | 14.16335 | 1.54E-45 | 6.48E-42 | up |
| UBE2C | 141.5451 | 3.488594 | 0.252766 | 13.80167 | 2.49E-43 | 8.36E-40 | up |
| FOXM1 | 90.09365 | 3.007524 | 0.219618 | 13.69433 | 1.10E-42 | 3.07E-39 | up |
| CCNA2 | 179.9109 | 3.673472 | 0.271263 | 13.5421 | 8.82E-42 | 1.91E-38 | up |
| TPX2 | 225.4599 | 3.089184 | 0.228159 | 13.53963 | 9.12E-42 | 1.91E-38 | up |
| AURKB | 118.6742 | 3.137006 | 0.234201 | 13.39452 | 6.51E-41 | 1.21E-37 | up |
| IGHV1-24 | 1309.801 | 5.748487 | 0.433379 | 13.26434 | 3.73E-40 | 6.25E-37 | up |
| CDC20 | 227.5903 | 3.580581 | 0.272468 | 13.14127 | 1.91E-39 | 2.91E-36 | up |
| HJURP | 86.60461 | 3.158277 | 0.24113 | 13.09784 | 3.39E-39 | 4.74E-36 | up |
| IGHG1 | 47224.03 | 5.199842 | 0.40375 | 12.87886 | 5.92E-38 | 7.64E-35 | up |
| PKMYT1 | 114.9459 | 3.139756 | 0.244088 | 12.86323 | 7.25E-38 | 8.69E-35 | up |
| KIF4A | 52.8425 | 3.439383 | 0.267765 | 12.8448 | 9.20E-38 | 1.03E-34 | up |
| MKI67 | 559.5648 | 3.085067 | 0.2428 | 12.7062 | 5.46E-37 | 5.73E-34 | up |
| SHCBP1 | 194.8005 | 3.132073 | 0.247048 | 12.67797 | 7.83E-37 | 7.73E-34 | up |
| BIRC5 | 234.2459 | 3.762577 | 0.29812 | 12.62101 | 1.62E-36 | 1.51E-33 | up |
| ASPM | 83.91815 | 2.896557 | 0.2296 | 12.61564 | 1.73E-36 | 1.53E-33 | up |
| DTL | 139.4967 | 3.085121 | 0.245739 | 12.55446 | 3.76E-36 | 3.15E-33 | up |
| MZB1 | 1862.549 | 3.585175 | 0.289514 | 12.38342 | 3.21E-35 | 2.57E-32 | up |
| IGHV4-59 | 1058.765 | 3.725776 | 0.30228 | 12.32558 | 6.60E-35 | 5.03E-32 | up |
| BUB1 | 231.8549 | 3.04763 | 0.24771 | 12.3032 | 8.71E-35 | 6.35E-32 | up |
| UHRF1 | 227.2587 | 2.730186 | 0.222404 | 12.27579 | 1.22E-34 | 8.55E-32 | up |
| H1-5 | 67.17907 | 3.192831 | 0.262459 | 12.16507 | 4.77E-34 | 3.20E-31 | up |
| CDT1 | 96.32474 | 2.711244 | 0.227257 | 11.93029 | 8.23E-33 | 5.31E-30 | up |
| CCNB2 | 147.2977 | 3.349743 | 0.283659 | 11.80906 | 3.50E-32 | 2.10E-29 | up |
| ESPL1 | 65.5707 | 2.7523 | 0.233054 | 11.80969 | 3.48E-32 | 2.10E-29 | up |
| KIF20A | 64.53391 | 3.421996 | 0.29091 | 11.76309 | 6.05E-32 | 3.50E-29 | up |
| H1-2 | 418.6391 | 1.797861 | 0.153254 | 11.73127 | 8.81E-32 | 4.93E-29 | up |
| PBK | 30.71643 | 3.994658 | 0.340814 | 11.72093 | 9.96E-32 | 5.39E-29 | up |
| GINS2 | 114.0445 | 2.733006 | 0.237864 | 11.48979 | 1.48E-30 | 7.79E-28 | up |
| MCM10 | 96.08017 | 3.435619 | 0.299916 | 11.45528 | 2.21E-30 | 1.12E-27 | up |
| TOP2A | 370.7072 | 3.099001 | 0.270575 | 11.45338 | 2.26E-30 | 1.12E-27 | up |
| CENPA | 35.80995 | 3.533903 | 0.309193 | 11.42944 | 2.98E-30 | 1.39E-27 | up |
| ZWINT | 293.8784 | 2.299964 | 0.201218 | 11.43019 | 2.95E-30 | 1.39E-27 | up |
| GTSE1 | 54.93877 | 3.037473 | 0.266646 | 11.39139 | 4.62E-30 | 2.09E-27 | up |
| MYBL2 | 958.3355 | 3.358381 | 0.296263 | 11.33582 | 8.72E-30 | 3.85E-27 | up |
| RNASE1 | 311.0017 | 5.038391 | 0.447552 | 11.25767 | 2.12E-29 | 9.14E-27 | up |
| CLSPN | 88.40232 | 2.574259 | 0.22879 | 11.2516 | 2.27E-29 | 9.54E-27 | up |
| CDCA2 | 66.06784 | 3.311809 | 0.295563 | 11.20508 | 3.85E-29 | 1.58E-26 | up |
| CDCA5 | 106.4208 | 2.79627 | 0.249846 | 11.19196 | 4.46E-29 | 1.78E-26 | up |
| CEP55 | 69.45171 | 3.39044 | 0.303598 | 11.16754 | 5.88E-29 | 2.29E-26 | up |
| IGHV1-18 | 1012.128 | 3.590949 | 0.32178 | 11.15965 | 6.42E-29 | 2.45E-26 | up |
| NCAPG | 89.48984 | 3.151265 | 0.282944 | 11.13741 | 8.25E-29 | 3.08E-26 | up |
| IGHV4-34 | 3050.09 | 4.195216 | 0.376941 | 11.12962 | 9.00E-29 | 3.28E-26 | up |
| BUB1B | 70.31037 | 2.719611 | 0.244773 | 11.11076 | 1.11E-28 | 3.97E-26 | up |
| DLGAP5 | 94.21512 | 3.739228 | 0.338748 | 11.03838 | 2.49E-28 | 8.72E-26 | up |
| HMMR | 45.63763 | 2.855443 | 0.259425 | 11.00681 | 3.54E-28 | 1.21E-25 | up |
| TROAP | 60.29201 | 3.347786 | 0.305617 | 10.95417 | 6.35E-28 | 2.13E-25 | up |
| KIF2C | 103.5911 | 2.834808 | 0.258827 | 10.95252 | 6.46E-28 | 2.13E-25 | up |
| RAD54L | 36.77557 | 2.796221 | 0.256117 | 10.91773 | 9.48E-28 | 3.06E-25 | up |
| KIFC1 | 75.45875 | 2.629148 | 0.241305 | 10.89554 | 1.21E-27 | 3.83E-25 | up |
| CDC45 | 106.7615 | 2.956438 | 0.27149 | 10.88969 | 1.29E-27 | 4.01E-25 | up |
| PYCR1 | 81.6008 | 3.104969 | 0.285703 | 10.86783 | 1.64E-27 | 5.01E-25 | up |
| IGHV1-46 | 998.3887 | 3.456208 | 0.318592 | 10.84837 | 2.03E-27 | 6.08E-25 | up |
| CDC6 | 109.7684 | 2.866952 | 0.264705 | 10.83075 | 2.46E-27 | 7.25E-25 | up |
| TYMS | 70.83977 | 2.895602 | 0.268263 | 10.7939 | 3.68E-27 | 1.06E-24 | up |
| DERL3 | 137.9964 | 2.641895 | 0.244855 | 10.78961 | 3.85E-27 | 1.10E-24 | up |
| SPC25 | 24.64995 | 3.800139 | 0.353215 | 10.75871 | 5.39E-27 | 1.51E-24 | up |
| TRIP13 | 89.17188 | 2.990049 | 0.27931 | 10.70512 | 9.63E-27 | 2.65E-24 | up |
| NCAPH | 114.1835 | 1.975176 | 0.18594 | 10.62267 | 2.34E-26 | 6.33E-24 | up |
| CDCA3 | 66.9619 | 2.343679 | 0.220918 | 10.60882 | 2.71E-26 | 7.11E-24 | up |
| IGKC | 40817.75 | 3.068355 | 0.289734 | 10.59025 | 3.31E-26 | 8.54E-24 | up |
| KCNN3 | 72.04689 | 3.90099 | 0.368889 | 10.57498 | 3.89E-26 | 9.90E-24 | up |
| CDC25A | 32.38257 | 3.023594 | 0.287748 | 10.50779 | 7.95E-26 | 1.99E-23 | up |
| E2F2 | 322.8703 | 1.764636 | 0.168314 | 10.48421 | 1.02E-25 | 2.52E-23 | up |
| IGLV9-49 | 251.7933 | 4.238885 | 0.404719 | 10.47365 | 1.14E-25 | 2.78E-23 | up |
| IGLV3-25 | 3140.042 | 4.409617 | 0.423945 | 10.40139 | 2.44E-25 | 5.78E-23 | up |
| ANLN | 50.49609 | 2.796102 | 0.2688 | 10.40217 | 2.42E-25 | 5.78E-23 | up |
| SPC24 | 40.26354 | 2.864793 | 0.276651 | 10.35527 | 3.96E-25 | 9.23E-23 | up |
| GPRC5D | 34.73392 | 3.339294 | 0.322928 | 10.34068 | 4.61E-25 | 1.06E-22 | up |
| IGLC3 | 6988.218 | 3.554419 | 0.345296 | 10.29382 | 7.51E-25 | 1.70E-22 | up |
| JCHAIN | 7604.117 | 3.498312 | 0.341191 | 10.25323 | 1.14E-24 | 2.56E-22 | up |
| SLC38A5 | 561.5491 | 1.521077 | 0.14859 | 10.23674 | 1.36E-24 | 3.00E-22 | up |
| CKAP2L | 43.2439 | 3.119949 | 0.30521 | 10.2223 | 1.58E-24 | 3.43E-22 | up |
| CDKN3 | 84.70302 | 2.690802 | 0.263816 | 10.19955 | 1.99E-24 | 4.29E-22 | up |
| POLQ | 74.87462 | 2.117871 | 0.207674 | 10.19803 | 2.02E-24 | 4.30E-22 | up |
| ESCO2 | 35.15393 | 2.548978 | 0.250158 | 10.18948 | 2.21E-24 | 4.64E-22 | up |
| SKA1 | 50.52427 | 2.955031 | 0.290479 | 10.17295 | 2.62E-24 | 5.43E-22 | up |
| RAD51 | 100.8076 | 2.454867 | 0.241565 | 10.16234 | 2.92E-24 | 5.98E-22 | up |
| DEPDC1 | 18.95776 | 3.4633 | 0.341079 | 10.15395 | 3.18E-24 | 6.44E-22 | up |
| TSHR | 26.82558 | 2.380997 | 0.235302 | 10.11891 | 4.55E-24 | 9.10E-22 | up |
| TXNDC5 | 245.2651 | 2.523535 | 0.249778 | 10.10312 | 5.35E-24 | 1.06E-21 | up |
| SKA3 | 56.74008 | 3.201237 | 0.317362 | 10.08703 | 6.31E-24 | 1.23E-21 | up |
| KIF11 | 180.3667 | 2.240335 | 0.222545 | 10.0669 | 7.74E-24 | 1.49E-21 | up |
| POC1A | 91.87002 | 1.599906 | 0.159171 | 10.05148 | 9.05E-24 | 1.73E-21 | up |
| MELK | 61.28363 | 3.044243 | 0.30454 | 9.996208 | 1.58E-23 | 2.95E-21 | up |
| KIF15 | 83.74715 | 2.350756 | 0.235376 | 9.987232 | 1.73E-23 | 3.20E-21 | up |
| IGKV3-15 | 1773.513 | 3.229871 | 0.324014 | 9.968314 | 2.10E-23 | 3.83E-21 | up |
| CCNB1 | 178.1835 | 2.213958 | 0.222853 | 9.934615 | 2.94E-23 | 5.31E-21 | up |
| NUF2 | 54.72401 | 2.061333 | 0.20781 | 9.919331 | 3.43E-23 | 6.13E-21 | up |
| GGH | 144.559 | 2.343972 | 0.236606 | 9.906647 | 3.89E-23 | 6.88E-21 | up |
| CDC25C | 13.82843 | 3.926079 | 0.397241 | 9.883357 | 4.92E-23 | 8.59E-21 | up |
| IGLV2-14 | 2537.97 | 3.217942 | 0.326445 | 9.857533 | 6.36E-23 | 1.10E-20 | up |
| KIF14 | 35.65839 | 2.552671 | 0.259205 | 9.848079 | 6.99E-23 | 1.20E-20 | up |
| IGLV2-23 | 1416.741 | 3.626069 | 0.369583 | 9.811238 | 1.01E-22 | 1.71E-20 | up |
| TNFRSF17 | 257.2954 | 3.314797 | 0.337905 | 9.809857 | 1.02E-22 | 1.71E-20 | up |
| PRDX4 | 546.0473 | 1.101752 | 0.112508 | 9.792669 | 1.21E-22 | 2.01E-20 | up |
| SDC1 | 88.00426 | 5.056675 | 0.517022 | 9.780383 | 1.37E-22 | 2.25E-20 | up |
| GLDC | 148.1671 | 3.778775 | 0.388464 | 9.727486 | 2.30E-22 | 3.75E-20 | up |
| PCLAF | 27.90623 | 3.089801 | 0.319524 | 9.670004 | 4.04E-22 | 6.53E-20 | up |
| IGKV4-1 | 3559.351 | 3.051774 | 0.317595 | 9.609002 | 7.33E-22 | 1.17E-19 | up |
| CENPF | 138.559 | 2.085851 | 0.217454 | 9.592166 | 8.63E-22 | 1.37E-19 | up |
| IGHV3-33 | 1177.355 | 3.390667 | 0.353687 | 9.586623 | 9.10E-22 | 1.43E-19 | up |
| IGKV1-17 | 167.3927 | 2.931794 | 0.306779 | 9.556693 | 1.22E-21 | 1.89E-19 | up |
| UCHL1 | 30.04624 | 5.143353 | 0.538705 | 9.547617 | 1.33E-21 | 2.04E-19 | up |
| DIAPH3 | 24.66544 | 3.089486 | 0.323708 | 9.544056 | 1.37E-21 | 2.10E-19 | up |
| IGLC2 | 14037.65 | 3.502167 | 0.367983 | 9.517208 | 1.78E-21 | 2.69E-19 | up |
| TICRR | 19.03178 | 2.503438 | 0.264254 | 9.473613 | 2.70E-21 | 4.05E-19 | up |
| IGLV3-27 | 245.3132 | 4.630905 | 0.490629 | 9.438702 | 3.77E-21 | 5.61E-19 | up |
| DEPDC1B | 49.12986 | 2.780591 | 0.295746 | 9.401949 | 5.36E-21 | 7.79E-19 | up |
| NEK2 | 33.34005 | 2.754953 | 0.293037 | 9.401396 | 5.38E-21 | 7.79E-19 | up |
| EXO1 | 53.61947 | 2.613882 | 0.277977 | 9.403221 | 5.29E-21 | 7.79E-19 | up |
| IGLV4-69 | 911.3821 | 3.586283 | 0.382187 | 9.383579 | 6.38E-21 | 8.99E-19 | up |
| IGHV3-21 | 1428.752 | 3.033397 | 0.323249 | 9.384085 | 6.35E-21 | 8.99E-19 | up |
| CDCA8 | 114.5364 | 1.808221 | 0.192692 | 9.383998 | 6.35E-21 | 8.99E-19 | up |
| IGHV5-51 | 917.9044 | 2.969241 | 0.316901 | 9.369623 | 7.28E-21 | 1.02E-18 | up |
| IGKV3-11 | 1903.229 | 2.782114 | 0.297095 | 9.364399 | 7.65E-21 | 1.06E-18 | up |
| TEDC2 | 32.62318 | 2.309748 | 0.24771 | 9.324415 | 1.12E-20 | 1.54E-18 | up |
| B9D1 | 33.10423 | 1.572427 | 0.169054 | 9.301341 | 1.39E-20 | 1.89E-18 | up |
| IGHV3-20 | 1255.863 | 4.759095 | 0.513647 | 9.265299 | 1.95E-20 | 2.63E-18 | up |
| IGLV2-11 | 990.1902 | 3.574564 | 0.386255 | 9.254403 | 2.15E-20 | 2.89E-18 | up |
| TTK | 43.35578 | 2.442368 | 0.264707 | 9.226692 | 2.79E-20 | 3.72E-18 | up |
| IGHV3-23 | 3293.978 | 3.140595 | 0.340646 | 9.219531 | 2.98E-20 | 3.92E-18 | up |
| MCM2 | 469.6472 | 1.702802 | 0.184701 | 9.219231 | 2.99E-20 | 3.92E-18 | up |
| ACOT7 | 264.5217 | 1.533671 | 0.166803 | 9.194528 | 3.77E-20 | 4.90E-18 | up |
| IGLV7-46 | 334.8656 | 3.752376 | 0.40994 | 9.153487 | 5.51E-20 | 7.12E-18 | up |
| IGKV1-5 | 2844.957 | 3.212971 | 0.351843 | 9.131839 | 6.73E-20 | 8.63E-18 | up |
| CDKN2C | 126.8054 | 1.161063 | 0.127269 | 9.122901 | 7.31E-20 | 9.30E-18 | up |
| IGKV5-2 | 30.94588 | 3.31794 | 0.365291 | 9.083007 | 1.06E-19 | 1.32E-17 | up |
| H1-3 | 446.3323 | 1.39821 | 0.153937 | 9.083017 | 1.06E-19 | 1.32E-17 | up |
| CDK1 | 123.5972 | 2.655832 | 0.293878 | 9.037192 | 1.61E-19 | 2.00E-17 | up |
| IGKV3D-11 | 62.79698 | 3.006115 | 0.332675 | 9.036192 | 1.62E-19 | 2.00E-17 | up |
| NME1 | 212.8337 | 1.165081 | 0.129267 | 9.013011 | 2.00E-19 | 2.46E-17 | up |
| IGHG3 | 7802.123 | 4.12549 | 0.45801 | 9.00743 | 2.11E-19 | 2.57E-17 | up |
| ORC1 | 76.86329 | 2.497911 | 0.278641 | 8.964626 | 3.11E-19 | 3.76E-17 | up |
| PIMREG | 20.12441 | 2.522075 | 0.281791 | 8.95016 | 3.55E-19 | 4.26E-17 | up |
| ACOXL | 16.81337 | 3.876867 | 0.435411 | 8.903921 | 5.39E-19 | 6.42E-17 | up |
| H2BC4 | 133.3995 | 1.986955 | 0.223445 | 8.892362 | 5.98E-19 | 7.07E-17 | up |
| H2AC14 | 15.27222 | 3.258439 | 0.367202 | 8.873696 | 7.08E-19 | 8.30E-17 | up |
| UBE2T | 79.65751 | 1.513684 | 0.171169 | 8.843204 | 9.30E-19 | 1.08E-16 | up |
| GALNT14 | 21.20335 | 3.169719 | 0.358541 | 8.840615 | 9.52E-19 | 1.10E-16 | up |
| E2F1 | 292.7727 | 2.643868 | 0.299513 | 8.827209 | 1.07E-18 | 1.23E-16 | up |
| IGLV6-57 | 1478.097 | 4.00668 | 0.454635 | 8.812952 | 1.22E-18 | 1.37E-16 | up |
| IGLV1-40 | 2144.099 | 3.004314 | 0.34213 | 8.781198 | 1.62E-18 | 1.81E-16 | up |
| IGHV1-14 | 58.88717 | 6.479103 | 0.739216 | 8.764828 | 1.87E-18 | 2.07E-16 | up |
| SLCO4A1 | 113.6587 | 2.670146 | 0.304892 | 8.757685 | 1.99E-18 | 2.17E-16 | up |
| LOC283710 | 49.85756 | 2.187911 | 0.249819 | 8.757986 | 1.99E-18 | 2.17E-16 | up |
| KNL1 | 84.46267 | 1.980506 | 0.226991 | 8.725049 | 2.66E-18 | 2.88E-16 | up |
| FABP5 | 117.43 | 1.250351 | 0.143908 | 8.688539 | 3.67E-18 | 3.95E-16 | up |
| IGLV1-51 | 126.3433 | 3.459891 | 0.398921 | 8.673119 | 4.20E-18 | 4.49E-16 | up |
| KIF18B | 33.34988 | 2.911902 | 0.336448 | 8.654837 | 4.94E-18 | 5.24E-16 | up |
| METTL7B | 178.1671 | 5.546713 | 0.641663 | 8.644272 | 5.41E-18 | 5.72E-16 | up |
| IGHV3-11 | 599.1419 | 3.025131 | 0.350247 | 8.637136 | 5.76E-18 | 6.05E-16 | up |
| IGLV1-47 | 1616.912 | 3.561058 | 0.414809 | 8.584806 | 9.10E-18 | 9.43E-16 | up |
| C1QC | 114.9918 | 3.48787 | 0.407565 | 8.557832 | 1.15E-17 | 1.18E-15 | up |
| OIP5 | 11.87962 | 2.16879 | 0.253962 | 8.53981 | 1.34E-17 | 1.38E-15 | up |
| H2AC12 | 21.38492 | 2.566353 | 0.300757 | 8.532976 | 1.43E-17 | 1.44E-15 | up |
| CTNNAL1 | 86.8015 | 2.111687 | 0.247668 | 8.526276 | 1.51E-17 | 1.52E-15 | up |
| C1QB | 389.3575 | 2.837358 | 0.333215 | 8.515102 | 1.66E-17 | 1.66E-15 | up |
| DHFR | 55.41116 | 1.694756 | 0.199133 | 8.51067 | 1.73E-17 | 1.72E-15 | up |
| IGHV1-2 | 2493.142 | 3.609142 | 0.42568 | 8.478531 | 2.28E-17 | 2.23E-15 | up |
| H2BC7 | 23.4828 | 2.304834 | 0.271826 | 8.479084 | 2.27E-17 | 2.23E-15 | up |
| CLU | 5041.621 | 3.165573 | 0.37447 | 8.453472 | 2.83E-17 | 2.74E-15 | up |
| IGHV3-69-1 | 52.49229 | 3.378548 | 0.400589 | 8.433951 | 3.34E-17 | 3.22E-15 | up |
| IGLV3-10 | 1158.186 | 4.416262 | 0.524756 | 8.415839 | 3.90E-17 | 3.73E-15 | up |
| PIF1 | 67.83954 | 1.604063 | 0.190623 | 8.41483 | 3.93E-17 | 3.73E-15 | up |
| IGKV1-12 | 135.7765 | 3.270156 | 0.38872 | 8.41262 | 4.01E-17 | 3.78E-15 | up |
| IGHV4-61 | 438.0179 | 3.492477 | 0.416199 | 8.39137 | 4.81E-17 | 4.48E-15 | up |
| ITM2C | 1918.641 | 1.708531 | 0.203598 | 8.391686 | 4.79E-17 | 4.48E-15 | up |
| CENPE | 81.72229 | 1.679245 | 0.200201 | 8.387794 | 4.95E-17 | 4.59E-15 | up |
| SEC11C | 1142.711 | 1.338934 | 0.159851 | 8.376161 | 5.47E-17 | 5.04E-15 | up |
| RECQL4 | 187.9131 | 1.525568 | 0.182637 | 8.35299 | 6.66E-17 | 6.07E-15 | up |
| BHLHA15 | 43.91335 | 3.510762 | 0.420356 | 8.351879 | 6.72E-17 | 6.10E-15 | up |
| IFI27L1 | 83.90675 | 1.348082 | 0.161625 | 8.340795 | 7.38E-17 | 6.62E-15 | up |
| MYL9 | 679.4207 | 3.683782 | 0.441773 | 8.338634 | 7.52E-17 | 6.67E-15 | up |
| H1-4 | 682.3707 | 1.420088 | 0.171134 | 8.298118 | 1.06E-16 | 9.29E-15 | up |
| CAV1 | 43.01747 | 3.249535 | 0.392145 | 8.286571 | 1.17E-16 | 1.02E-14 | up |
| XK | 150.5992 | 3.31471 | 0.401916 | 8.247265 | 1.62E-16 | 1.41E-14 | up |
| IGHV4-28 | 107.9411 | 3.013952 | 0.365474 | 8.246688 | 1.63E-16 | 1.41E-14 | up |
| IGLV5-45 | 224.7901 | 3.858704 | 0.468469 | 8.236845 | 1.77E-16 | 1.52E-14 | up |
| SDF2L1 | 1015.785 | 1.181876 | 0.143615 | 8.229462 | 1.88E-16 | 1.61E-14 | up |
| IGHV3-30 | 1754.301 | 3.021228 | 0.367195 | 8.22785 | 1.91E-16 | 1.62E-14 | up |
| ADAMTS2 | 263.4793 | 6.293283 | 0.765132 | 8.225091 | 1.95E-16 | 1.65E-14 | up |
| HMGB3 | 123.8243 | 1.801968 | 0.219087 | 8.224893 | 1.95E-16 | 1.65E-14 | up |
| NDC80 | 136.365 | 1.54536 | 0.187902 | 8.224298 | 1.96E-16 | 1.65E-14 | up |
| MCM4 | 756.3013 | 1.578313 | 0.19218 | 8.212663 | 2.16E-16 | 1.81E-14 | up |
| DYNC2I2 | 293.4494 | 1.192768 | 0.145349 | 8.206254 | 2.28E-16 | 1.90E-14 | up |
| IGKV2-30 | 488.178 | 3.766867 | 0.460859 | 8.17357 | 2.99E-16 | 2.42E-14 | up |
| NEIL3 | 19.46001 | 2.172426 | 0.265908 | 8.169848 | 3.09E-16 | 2.48E-14 | up |
| MYL6B | 55.56194 | 1.352523 | 0.166133 | 8.141179 | 3.91E-16 | 3.13E-14 | up |
| CA1 | 603.117 | 7.075478 | 0.869593 | 8.136541 | 4.07E-16 | 3.24E-14 | up |
| PLK4 | 96.49521 | 1.397062 | 0.172412 | 8.103038 | 5.36E-16 | 4.18E-14 | up |
| IGHA2 | 6055.875 | 3.338776 | 0.412826 | 8.087604 | 6.08E-16 | 4.73E-14 | up |
| DNAH8 | 15.54667 | 1.781249 | 0.220411 | 8.081488 | 6.40E-16 | 4.95E-14 | up |
| MND1 | 8.971408 | 3.111052 | 0.385485 | 8.070495 | 7.00E-16 | 5.39E-14 | up |
| HBD | 795.4028 | 6.384435 | 0.791229 | 8.069015 | 7.09E-16 | 5.43E-14 | up |
| E2F7 | 19.51114 | 2.687231 | 0.333057 | 8.068383 | 7.12E-16 | 5.43E-14 | up |
| CENPW | 98.40612 | 1.4088 | 0.17541 | 8.031444 | 9.63E-16 | 7.28E-14 | up |
| IGLV3-16 | 91.82287 | 3.631652 | 0.452948 | 8.017811 | 1.08E-15 | 8.10E-14 | up |
| IGHA1 | 42792.68 | 3.22115 | 0.402312 | 8.006593 | 1.18E-15 | 8.80E-14 | up |
| IGKV2-24 | 166.7122 | 4.080915 | 0.513503 | 7.947201 | 1.91E-15 | 1.40E-13 | up |
| GMPR | 469.3709 | 2.660921 | 0.335029 | 7.94235 | 1.98E-15 | 1.45E-13 | up |
| TGFBR1 | 987.9112 | -1.0217 | 0.12895 | -7.92321 | 2.31E-15 | 1.68E-13 | down |
| CENPU | 103.1454 | 1.887696 | 0.238991 | 7.898597 | 2.82E-15 | 2.04E-13 | up |
| POLE2 | 34.9471 | 1.998541 | 0.253111 | 7.895918 | 2.88E-15 | 2.08E-13 | up |
| IGHV3-49 | 506.5836 | 3.035861 | 0.384599 | 7.89357 | 2.94E-15 | 2.11E-13 | up |
| MYDGF | 2031.012 | 1.044339 | 0.132398 | 7.887871 | 3.07E-15 | 2.20E-13 | up |
| LINC01482 | 7.429522 | 3.052774 | 0.387283 | 7.882547 | 3.21E-15 | 2.27E-13 | up |
| FEN1 | 512.5739 | 1.132278 | 0.14376 | 7.876162 | 3.38E-15 | 2.38E-13 | up |
| STIL | 85.74438 | 1.720986 | 0.218921 | 7.861228 | 3.80E-15 | 2.67E-13 | up |
| CHEK1 | 75.13942 | 1.637822 | 0.208381 | 7.859752 | 3.85E-15 | 2.68E-13 | up |
| IGLV5-37 | 114.3323 | 4.62932 | 0.58949 | 7.853088 | 4.06E-15 | 2.82E-13 | up |
| IGKV1-16 | 271.9773 | 2.880802 | 0.366908 | 7.851562 | 4.11E-15 | 2.84E-13 | up |
| NT5DC2 | 167.6671 | 2.121546 | 0.270295 | 7.848992 | 4.19E-15 | 2.87E-13 | up |
| H2AC13 | 18.59752 | 2.426636 | 0.309386 | 7.843393 | 4.39E-15 | 2.98E-13 | up |
| IGLV1-44 | 1601.16 | 3.152707 | 0.402148 | 7.839674 | 4.52E-15 | 3.06E-13 | up |
| PTGR1 | 21.76166 | 2.683305 | 0.343103 | 7.820704 | 5.25E-15 | 3.54E-13 | up |
| ZC3HAV1L | 20.88848 | 2.451142 | 0.313684 | 7.814054 | 5.54E-15 | 3.72E-13 | up |
| GAS6 | 424.6884 | 1.804054 | 0.231752 | 7.784404 | 7.00E-15 | 4.65E-13 | up |
| TRIM58 | 1244.653 | 2.848823 | 0.366267 | 7.777998 | 7.37E-15 | 4.87E-13 | up |
| TREML1 | 956.576 | 2.889752 | 0.37215 | 7.765008 | 8.16E-15 | 5.37E-13 | up |
| CHPF | 420.2379 | 1.472628 | 0.190032 | 7.749363 | 9.24E-15 | 6.01E-13 | up |
| KIF23 | 94.09045 | 1.733099 | 0.223939 | 7.739173 | 1.00E-14 | 6.46E-13 | up |
| WDR62 | 99.19261 | 1.28323 | 0.1659 | 7.734979 | 1.03E-14 | 6.65E-13 | up |
| SLC6A9 | 21.05004 | 3.36294 | 0.435411 | 7.723597 | 1.13E-14 | 7.19E-13 | up |
| ALDH1L2 | 21.07837 | 2.04798 | 0.265212 | 7.722059 | 1.14E-14 | 7.25E-13 | up |
| FBXO43 | 7.786467 | 2.601286 | 0.338549 | 7.683634 | 1.55E-14 | 9.65E-13 | up |
| H2BC21 | 794.9786 | 1.687572 | 0.220268 | 7.661453 | 1.84E-14 | 1.14E-12 | up |
| IGKV3D-20 | 505.4028 | 4.825504 | 0.629926 | 7.660435 | 1.85E-14 | 1.15E-12 | up |
| MTFR2 | 26.49716 | 1.785955 | 0.233418 | 7.651333 | 1.99E-14 | 1.23E-12 | up |
| ITGA2B | 5114.755 | 3.045113 | 0.398799 | 7.635706 | 2.25E-14 | 1.38E-12 | up |
| ASF1B | 300.7428 | 1.179054 | 0.154569 | 7.628021 | 2.38E-14 | 1.46E-12 | up |
| H2BC12 | 178.4901 | 1.259077 | 0.165613 | 7.602518 | 2.90E-14 | 1.77E-12 | up |
| E2F8 | 24.61007 | 2.761021 | 0.36324 | 7.601086 | 2.94E-14 | 1.79E-12 | up |
| IGHV3-43 | 318.4983 | 3.529418 | 0.464452 | 7.599099 | 2.98E-14 | 1.81E-12 | up |
| EGF | 111.6085 | 2.764079 | 0.364342 | 7.586501 | 3.29E-14 | 1.98E-12 | up |
| MBLAC2 | 62.77732 | -1.05157 | 0.139059 | -7.56207 | 3.97E-14 | 2.36E-12 | down |
| H3C3 | 5.181708 | 3.505769 | 0.463966 | 7.556098 | 4.15E-14 | 2.46E-12 | up |
| SGO1 | 32.08211 | 1.711214 | 0.226598 | 7.551751 | 4.29E-14 | 2.54E-12 | up |
| FAM20A | 367.1991 | 3.263267 | 0.433943 | 7.520029 | 5.48E-14 | 3.20E-12 | up |
| IGHV3-66 | 118.0074 | 2.881179 | 0.385804 | 7.467984 | 8.14E-14 | 4.68E-12 | up |
| IGHV2-5 | 600.8507 | 2.846472 | 0.381249 | 7.466172 | 8.26E-14 | 4.73E-12 | up |
| SPAG5 | 273.8227 | 1.644046 | 0.220244 | 7.464659 | 8.35E-14 | 4.77E-12 | up |
| CMTM5 | 197.3981 | 2.755257 | 0.369936 | 7.44793 | 9.48E-14 | 5.39E-12 | up |
| IGKV1-27 | 184.7799 | 3.047338 | 0.409325 | 7.444794 | 9.71E-14 | 5.51E-12 | up |
| PHGDH | 195.3927 | 1.413063 | 0.189992 | 7.437469 | 1.03E-13 | 5.80E-12 | up |
| EGFL7 | 101.5846 | 2.415537 | 0.32496 | 7.433335 | 1.06E-13 | 5.96E-12 | up |
| KLF1 | 59.69837 | 3.203461 | 0.431136 | 7.430274 | 1.08E-13 | 6.07E-12 | up |
| MT1G | 5.813149 | 3.190062 | 0.429337 | 7.430202 | 1.08E-13 | 6.07E-12 | up |
| ABLIM3 | 239.3606 | 2.752418 | 0.371332 | 7.412284 | 1.24E-13 | 6.90E-12 | up |
| MCM6 | 661.1503 | 1.038321 | 0.140095 | 7.41154 | 1.25E-13 | 6.92E-12 | up |
| IGLV3-19 | 3157.433 | 3.88139 | 0.524857 | 7.395136 | 1.41E-13 | 7.75E-12 | up |
| H2AJ | 562.8759 | 1.090229 | 0.148186 | 7.357164 | 1.88E-13 | 1.01E-11 | up |
| H2BC11 | 68.47745 | 2.488349 | 0.338399 | 7.353289 | 1.93E-13 | 1.04E-11 | up |
| AURKA | 94.08882 | 1.332865 | 0.181319 | 7.350951 | 1.97E-13 | 1.06E-11 | up |
| SPATS2 | 123.5772 | 1.105128 | 0.150482 | 7.343939 | 2.07E-13 | 1.11E-11 | up |
| SELENBP1 | 265.9845 | 6.012801 | 0.819512 | 7.337053 | 2.18E-13 | 1.16E-11 | up |
| SLC16A14 | 24.86646 | 2.512669 | 0.342659 | 7.332845 | 2.25E-13 | 1.20E-11 | up |
| IGLV3-21 | 1726.828 | 2.852583 | 0.389749 | 7.319026 | 2.50E-13 | 1.32E-11 | up |
| SPARC | 3553.491 | 2.568664 | 0.351037 | 7.317355 | 2.53E-13 | 1.33E-11 | up |
| TTLL7 | 24.61314 | 2.513268 | 0.34364 | 7.313676 | 2.60E-13 | 1.36E-11 | up |
| MAOB | 48.79007 | 4.491204 | 0.614878 | 7.304215 | 2.79E-13 | 1.45E-11 | up |
| MAD2L1 | 184.6261 | 1.390255 | 0.190521 | 7.297104 | 2.94E-13 | 1.52E-11 | up |
| PTTG1 | 204.0463 | 1.514002 | 0.207987 | 7.279309 | 3.36E-13 | 1.73E-11 | up |
| MANF | 443.1408 | 1.130045 | 0.155499 | 7.267209 | 3.67E-13 | 1.88E-11 | up |
| IGHV3-7 | 1116.044 | 2.561943 | 0.352981 | 7.258012 | 3.93E-13 | 2.00E-11 | up |
| RAD51AP1 | 54.73371 | 1.444941 | 0.199427 | 7.245477 | 4.31E-13 | 2.19E-11 | up |
| CYSLTR2 | 194.5357 | -1.77411 | 0.244963 | -7.24237 | 4.41E-13 | 2.24E-11 | down |
| H3C8 | 6.984291 | 3.34818 | 0.462832 | 7.234121 | 4.69E-13 | 2.36E-11 | up |
| PPARG | 43.23741 | 3.101831 | 0.428858 | 7.232777 | 4.73E-13 | 2.38E-11 | up |
| CD38 | 941.1678 | 1.622397 | 0.224608 | 7.223242 | 5.08E-13 | 2.53E-11 | up |
| TP73 | 27.75719 | 2.144623 | 0.297353 | 7.212381 | 5.50E-13 | 2.73E-11 | up |
| MIXL1 | 13.7171 | 3.265174 | 0.452846 | 7.210344 | 5.58E-13 | 2.76E-11 | up |
| PDIA4 | 4586.825 | 1.003363 | 0.139163 | 7.209977 | 5.60E-13 | 2.76E-11 | up |
| IGKV1-8 | 224.3779 | 2.792119 | 0.387536 | 7.204801 | 5.81E-13 | 2.86E-11 | up |
| MDK | 19.85011 | 1.56697 | 0.217579 | 7.201834 | 5.94E-13 | 2.90E-11 | up |
| EPB42 | 133.4009 | 5.47748 | 0.762253 | 7.185911 | 6.68E-13 | 3.24E-11 | up |
| DCC | 8.447734 | 3.743665 | 0.523481 | 7.151477 | 8.58E-13 | 4.15E-11 | up |
| SCN1B | 57.76851 | 1.718669 | 0.240978 | 7.13206 | 9.89E-13 | 4.73E-11 | up |
| CIT | 48.7449 | 1.695361 | 0.238516 | 7.10796 | 1.18E-12 | 5.60E-11 | up |
| MT1E | 59.39957 | 1.975457 | 0.278228 | 7.100135 | 1.25E-12 | 5.89E-11 | up |
| SMOX | 603.9626 | 2.311348 | 0.3258 | 7.094376 | 1.30E-12 | 6.13E-11 | up |
| TGFB1I1 | 67.8044 | 2.581165 | 0.363951 | 7.092074 | 1.32E-12 | 6.19E-11 | up |
| TNNT1 | 217.038 | 2.990545 | 0.421891 | 7.088426 | 1.36E-12 | 6.34E-11 | up |
| FBXO5 | 134.2486 | 1.079829 | 0.152935 | 7.060712 | 1.66E-12 | 7.70E-11 | up |
| H2BC8 | 50.79196 | 1.95434 | 0.276923 | 7.057339 | 1.70E-12 | 7.87E-11 | up |
| CACNA2D3 | 109.9677 | -1.93689 | 0.27544 | -7.03198 | 2.04E-12 | 9.31E-11 | down |
| PIR | 6.298788 | 2.81364 | 0.400184 | 7.030865 | 2.05E-12 | 9.36E-11 | up |
| SELP | 650.3036 | 2.480979 | 0.353216 | 7.023975 | 2.16E-12 | 9.81E-11 | up |
| STMN1 | 1287.001 | 1.341114 | 0.191259 | 7.012027 | 2.35E-12 | 1.06E-10 | up |
| FAM72A | 11.73844 | 1.732498 | 0.247286 | 7.006039 | 2.45E-12 | 1.11E-10 | up |
| SH3TC2 | 75.48891 | 2.165666 | 0.309387 | 6.999853 | 2.56E-12 | 1.15E-10 | up |
| PLOD2 | 30.84899 | 2.569798 | 0.367398 | 6.994579 | 2.66E-12 | 1.19E-10 | up |
| IGKV1-6 | 156.439 | 2.884286 | 0.41264 | 6.989835 | 2.75E-12 | 1.22E-10 | up |
| RAP1GAP | 39.82903 | 5.65327 | 0.809159 | 6.9866 | 2.82E-12 | 1.25E-10 | up |
| FAM111B | 76.3673 | 1.714935 | 0.24601 | 6.970985 | 3.15E-12 | 1.38E-10 | up |
| IGKV1D-12 | 10.60865 | 3.981419 | 0.571994 | 6.9606 | 3.39E-12 | 1.47E-10 | up |
| FBXO30 | 161.6573 | -1.16488 | 0.167447 | -6.95674 | 3.48E-12 | 1.51E-10 | down |
| FAM72B | 9.582871 | 2.173806 | 0.312706 | 6.951591 | 3.61E-12 | 1.56E-10 | up |
| LGALS1 | 4175.952 | 1.239477 | 0.178336 | 6.950242 | 3.65E-12 | 1.57E-10 | up |
| PSAT1 | 137.1683 | 1.459803 | 0.210189 | 6.945176 | 3.78E-12 | 1.62E-10 | up |
| SAMD14 | 46.7732 | 3.251306 | 0.468193 | 6.944373 | 3.80E-12 | 1.63E-10 | up |
| RHAG | 14.45101 | 5.905168 | 0.850493 | 6.943229 | 3.83E-12 | 1.64E-10 | up |
| ZNF304 | 108.5115 | -1.59728 | 0.230209 | -6.93838 | 3.97E-12 | 1.69E-10 | down |
| SPTB | 148.6147 | 2.92194 | 0.422028 | 6.923576 | 4.40E-12 | 1.87E-10 | up |
| AUNIP | 15.22889 | 1.794566 | 0.259405 | 6.918015 | 4.58E-12 | 1.94E-10 | up |
| PDIA5 | 220.6576 | 1.183103 | 0.171083 | 6.915391 | 4.67E-12 | 1.97E-10 | up |
| DSCC1 | 20.57312 | 2.123237 | 0.307084 | 6.91418 | 4.71E-12 | 1.98E-10 | up |
| ZNF284 | 29.12592 | -1.08481 | 0.157247 | -6.89874 | 5.25E-12 | 2.20E-10 | down |
| CDC42BPA | 53.85503 | 1.829211 | 0.265282 | 6.895343 | 5.37E-12 | 2.24E-10 | up |
| LNCAROD | 8.660757 | 3.044707 | 0.441911 | 6.889864 | 5.58E-12 | 2.31E-10 | up |
| LOC105378305 | 8.660757 | 3.044707 | 0.441911 | 6.889864 | 5.58E-12 | 2.31E-10 | up |
| MOXD1 | 27.64899 | 1.708327 | 0.247957 | 6.889613 | 5.59E-12 | 2.31E-10 | up |
| LOC102723407 | 1510.696 | 3.599063 | 0.522963 | 6.882064 | 5.90E-12 | 2.41E-10 | up |
| MAPK12 | 16.8764 | 1.788113 | 0.260024 | 6.87673 | 6.12E-12 | 2.50E-10 | up |
| ZNF419 | 93.45592 | -1.10476 | 0.161133 | -6.8562 | 7.07E-12 | 2.88E-10 | down |
| MFSD2B | 110.8677 | 2.593927 | 0.379058 | 6.84308 | 7.75E-12 | 3.14E-10 | up |
| MPIG6B | 1283.336 | 2.4093 | 0.352182 | 6.841059 | 7.86E-12 | 3.18E-10 | up |
| IGF1 | 6.586919 | 4.070859 | 0.595192 | 6.839575 | 7.94E-12 | 3.20E-10 | up |
| IGHV4-30-2 | 294.5128 | 2.747512 | 0.402582 | 6.824728 | 8.81E-12 | 3.52E-10 | up |
| ANXA3 | 34.23215 | 3.871024 | 0.568202 | 6.812764 | 9.57E-12 | 3.82E-10 | up |
| IGKV1D-16 | 57.21561 | 3.635317 | 0.535329 | 6.790806 | 1.12E-11 | 4.40E-10 | up |
| IGHV3-15 | 729.1569 | 2.546359 | 0.375226 | 6.786202 | 1.15E-11 | 4.54E-10 | up |
| SNCA | 630.0643 | 2.459792 | 0.363283 | 6.771011 | 1.28E-11 | 4.98E-10 | up |
| H4C8 | 109.9767 | 2.451188 | 0.362294 | 6.765734 | 1.33E-11 | 5.15E-10 | up |
| IGHV1-69-2 | 579.6414 | 5.53356 | 0.819223 | 6.754647 | 1.43E-11 | 5.52E-10 | up |
| HPDL | 18.56574 | 1.748318 | 0.258888 | 6.753174 | 1.45E-11 | 5.57E-10 | up |
| CAV2 | 31.0662 | 2.750136 | 0.407652 | 6.746278 | 1.52E-11 | 5.80E-10 | up |
| IGHV3-48 | 1291.733 | 2.938683 | 0.435774 | 6.743589 | 1.55E-11 | 5.89E-10 | up |
| RNASE2 | 1373.98 | 2.48547 | 0.368653 | 6.742027 | 1.56E-11 | 5.94E-10 | up |
| IGHV4-39 | 1889.225 | 3.327279 | 0.49445 | 6.729252 | 1.71E-11 | 6.45E-10 | up |
| RHCE | 24.24975 | 2.158803 | 0.321515 | 6.714477 | 1.89E-11 | 7.07E-10 | up |
| HBA2 | 7622.481 | 6.726699 | 1.002442 | 6.710314 | 1.94E-11 | 7.24E-10 | up |
| LCN2 | 61.96194 | 2.667827 | 0.397865 | 6.705354 | 2.01E-11 | 7.48E-10 | up |
| IFITM10 | 4.786057 | 2.92317 | 0.436953 | 6.689903 | 2.23E-11 | 8.24E-10 | up |
| IGHV6-1 | 222.0374 | 2.575717 | 0.385232 | 6.686144 | 2.29E-11 | 8.42E-10 | up |
| HBG2 | 6.015032 | 4.326956 | 0.647514 | 6.682413 | 2.35E-11 | 8.59E-10 | up |
| SCUBE1 | 31.41842 | 3.204207 | 0.479675 | 6.679958 | 2.39E-11 | 8.70E-10 | up |
| PARPBP | 60.54307 | 1.254505 | 0.18785 | 6.678219 | 2.42E-11 | 8.79E-10 | up |
| ARNTL2 | 53.84299 | 2.006826 | 0.300795 | 6.671747 | 2.53E-11 | 9.14E-10 | up |
| GATA1 | 89.67858 | 2.533966 | 0.379944 | 6.669308 | 2.57E-11 | 9.28E-10 | up |
| OSBP2 | 446.1586 | 3.077089 | 0.461921 | 6.661503 | 2.71E-11 | 9.74E-10 | up |
| APOBEC3B | 85.7099 | 2.190813 | 0.329217 | 6.654619 | 2.84E-11 | 1.02E-09 | up |
| ARHGAP18 | 447.1365 | 1.034527 | 0.155829 | 6.638874 | 3.16E-11 | 1.12E-09 | up |
| CYP19A1 | 17.52911 | 5.248576 | 0.79258 | 6.622138 | 3.54E-11 | 1.25E-09 | up |
| ZNF607 | 68.27281 | -1.29665 | 0.195868 | -6.62 | 3.59E-11 | 1.26E-09 | down |
| BRCA1 | 111.3456 | 1.088501 | 0.164502 | 6.616956 | 3.67E-11 | 1.28E-09 | up |
| H3C2 | 9.287206 | 2.808707 | 0.424976 | 6.609087 | 3.87E-11 | 1.34E-09 | up |
| BMP6 | 187.4563 | 1.808289 | 0.274329 | 6.591686 | 4.35E-11 | 1.50E-09 | up |
| PF4V1 | 248.9585 | 2.898721 | 0.440743 | 6.576898 | 4.80E-11 | 1.65E-09 | up |
| HSP90B1 | 7946.555 | 1.012371 | 0.154113 | 6.569025 | 5.06E-11 | 1.73E-09 | up |
| H3C10 | 636.9616 | 2.308307 | 0.352336 | 6.551438 | 5.70E-11 | 1.93E-09 | up |
| FAM71F2 | 6.798822 | 1.816609 | 0.277962 | 6.535468 | 6.34E-11 | 2.14E-09 | up |
| KAZALD1 | 9.755702 | 1.869603 | 0.286154 | 6.533549 | 6.42E-11 | 2.16E-09 | up |
| H4C9 | 69.62982 | 1.158178 | 0.177441 | 6.527101 | 6.71E-11 | 2.25E-09 | up |
| H2AC6 | 2320.294 | 1.915846 | 0.293815 | 6.520577 | 7.00E-11 | 2.34E-09 | up |
| IGKV2D-40 | 20.05598 | 3.19252 | 0.490078 | 6.514311 | 7.30E-11 | 2.42E-09 | up |
| WFDC1 | 7.966866 | 4.850812 | 0.745875 | 6.503516 | 7.85E-11 | 2.58E-09 | up |
| H2AC11 | 152.9342 | 1.614754 | 0.248305 | 6.503121 | 7.87E-11 | 2.58E-09 | up |
| MAP1B | 44.47731 | 1.562288 | 0.240459 | 6.497096 | 8.19E-11 | 2.68E-09 | up |
| GRB14 | 19.34452 | 2.781195 | 0.429097 | 6.481509 | 9.08E-11 | 2.94E-09 | up |
| SLC4A1 | 157.3198 | 5.593654 | 0.863682 | 6.476524 | 9.39E-11 | 3.01E-09 | up |
| ABCB9 | 103.2518 | 1.381253 | 0.213268 | 6.476618 | 9.38E-11 | 3.01E-09 | up |
| CD63 | 5646.543 | 1.260922 | 0.194851 | 6.471203 | 9.72E-11 | 3.10E-09 | up |
| H2AC8 | 35.77229 | 2.032463 | 0.314296 | 6.466722 | 1.00E-10 | 3.19E-09 | up |
| LIMS1 | 2083.481 | 1.114919 | 0.172555 | 6.461234 | 1.04E-10 | 3.30E-09 | up |
| STRIP2 | 28.15516 | 1.774135 | 0.274653 | 6.45954 | 1.05E-10 | 3.33E-09 | up |
| ORC6 | 48.31844 | 1.260444 | 0.195141 | 6.459129 | 1.05E-10 | 3.33E-09 | up |
| MSRB3 | 73.81349 | 1.714115 | 0.265468 | 6.456967 | 1.07E-10 | 3.37E-09 | up |
| DMC1 | 6.880378 | 2.51532 | 0.39004 | 6.448885 | 1.13E-10 | 3.53E-09 | up |
| MPL | 48.95411 | 2.204873 | 0.342026 | 6.446504 | 1.14E-10 | 3.58E-09 | up |
| TFPI | 50.68233 | 2.426313 | 0.376668 | 6.441513 | 1.18E-10 | 3.69E-09 | up |
| B4GALT2 | 77.39028 | 1.244388 | 0.193187 | 6.441362 | 1.18E-10 | 3.69E-09 | up |
| PDE2A | 57.48402 | 2.109472 | 0.327593 | 6.439316 | 1.20E-10 | 3.73E-09 | up |
| IGHV3-53 | 294.2743 | 2.495051 | 0.38802 | 6.430216 | 1.27E-10 | 3.94E-09 | up |
| IGHV2-26 | 123.6925 | 2.321824 | 0.361382 | 6.424846 | 1.32E-10 | 4.07E-09 | up |
| RPL31P43 | 19.74637 | -1.98308 | 0.308648 | -6.42507 | 1.32E-10 | 4.07E-09 | down |
| ALAS2 | 2522.871 | 6.283507 | 0.978507 | 6.421527 | 1.35E-10 | 4.14E-09 | up |
| KLRK1 | 71.02174 | -1.7259 | 0.269267 | -6.40963 | 1.46E-10 | 4.44E-09 | down |
| KLRC4-KLRK1 | 71.02174 | -1.7259 | 0.269267 | -6.40963 | 1.46E-10 | 4.44E-09 | down |
| H2BC17 | 22.24024 | 3.667958 | 0.572526 | 6.406626 | 1.49E-10 | 4.52E-09 | up |
| GYPB | 28.93248 | 6.828924 | 1.069386 | 6.385835 | 1.70E-10 | 5.07E-09 | up |
| IGHV3-74 | 830.9703 | 2.589625 | 0.405628 | 6.384243 | 1.72E-10 | 5.12E-09 | up |
| NT5M | 155.2521 | 2.054055 | 0.322514 | 6.368893 | 1.90E-10 | 5.60E-09 | up |
| PPBP | 4915.831 | 2.226064 | 0.350038 | 6.359501 | 2.02E-10 | 5.93E-09 | up |
| CLEC2L | 31.27793 | 3.123332 | 0.491176 | 6.358892 | 2.03E-10 | 5.94E-09 | up |
| PF4 | 1180.558 | 2.031132 | 0.319715 | 6.35295 | 2.11E-10 | 6.17E-09 | up |
| PKHD1L1 | 64.07887 | 1.773007 | 0.279303 | 6.347977 | 2.18E-10 | 6.36E-09 | up |
| GP9 | 531.4445 | 2.098878 | 0.33066 | 6.347546 | 2.19E-10 | 6.36E-09 | up |
| MAFB | 5873.458 | 1.507789 | 0.237802 | 6.340523 | 2.29E-10 | 6.65E-09 | up |
| FANCI | 400.7176 | 1.044631 | 0.164911 | 6.334509 | 2.38E-10 | 6.89E-09 | up |
| KLRK1-AS1 | 90.32223 | -1.26049 | 0.199204 | -6.32762 | 2.49E-10 | 7.17E-09 | down |
| CCL25 | 5.406198 | 2.915665 | 0.4615 | 6.317799 | 2.65E-10 | 7.59E-09 | up |
| ELOVL7 | 167.5711 | 2.178465 | 0.344912 | 6.316008 | 2.68E-10 | 7.64E-09 | up |
| ITGA1 | 140.0396 | 2.049227 | 0.324431 | 6.316375 | 2.68E-10 | 7.64E-09 | up |
| SLC12A8 | 14.81998 | 1.934274 | 0.306602 | 6.308747 | 2.81E-10 | 7.91E-09 | up |
| ITGB3 | 1595.839 | 2.474448 | 0.392754 | 6.300254 | 2.97E-10 | 8.28E-09 | up |
| PLEKHH3 | 26.45676 | 1.145571 | 0.181872 | 6.298759 | 3.00E-10 | 8.35E-09 | up |
| TTC7B | 170.3104 | 1.869497 | 0.297034 | 6.293872 | 3.10E-10 | 8.58E-09 | up |
| TMEM119 | 45.19804 | 1.96656 | 0.312556 | 6.291874 | 3.14E-10 | 8.67E-09 | up |
| CHAC2 | 44.07549 | 1.381617 | 0.219855 | 6.284211 | 3.30E-10 | 9.10E-09 | up |
| TNFAIP8L3 | 8.091126 | 3.375753 | 0.537472 | 6.280797 | 3.37E-10 | 9.28E-09 | up |
| TERT | 8.71226 | 2.048421 | 0.326258 | 6.27853 | 3.42E-10 | 9.40E-09 | up |
| IGHV4-55 | 80.93251 | 2.590252 | 0.413232 | 6.26828 | 3.65E-10 | 9.99E-09 | up |
| TMEM158 | 90.67125 | 2.049006 | 0.326895 | 6.26808 | 3.66E-10 | 9.99E-09 | up |
| IQGAP3 | 10.76608 | 2.794415 | 0.446803 | 6.254251 | 3.99E-10 | 1.08E-08 | up |
| IGHV2-70 | 201.3753 | 3.93742 | 0.630802 | 6.241928 | 4.32E-10 | 1.16E-08 | up |
| CENPM | 213.3313 | 1.151977 | 0.18467 | 6.238018 | 4.43E-10 | 1.19E-08 | up |
| H3C12 | 4.558375 | 2.955953 | 0.474149 | 6.234231 | 4.54E-10 | 1.21E-08 | up |
| SLC4A10 | 61.19847 | -2.57453 | 0.413083 | -6.23247 | 4.59E-10 | 1.22E-08 | down |
| IGLC6 | 35.53235 | 2.892868 | 0.46453 | 6.227514 | 4.74E-10 | 1.26E-08 | up |
| CASS4 | 211.6213 | -1.06091 | 0.170461 | -6.22374 | 4.85E-10 | 1.28E-08 | down |
| IGKV2D-29 | 176.3828 | 3.234922 | 0.520186 | 6.218781 | 5.01E-10 | 1.31E-08 | up |
| ASS1 | 4.303399 | 3.69153 | 0.594014 | 6.214547 | 5.15E-10 | 1.34E-08 | up |
| TRIM7 | 121.3198 | 1.533573 | 0.24717 | 6.204522 | 5.49E-10 | 1.42E-08 | up |
| TSPAN9 | 26.50592 | 2.208164 | 0.355956 | 6.20347 | 5.52E-10 | 1.43E-08 | up |
| IGLV4-60 | 135.0562 | 2.819181 | 0.454745 | 6.199472 | 5.67E-10 | 1.46E-08 | up |
| GSTM5 | 13.54929 | 3.218939 | 0.519721 | 6.193585 | 5.88E-10 | 1.51E-08 | up |
| LOC105372321 | 7.972604 | 2.218727 | 0.359142 | 6.177847 | 6.50E-10 | 1.67E-08 | up |
| LAPTM4B | 116.2944 | 1.315636 | 0.213064 | 6.174829 | 6.62E-10 | 1.69E-08 | up |
| SMIM1 | 15.57789 | 2.718365 | 0.440287 | 6.174072 | 6.66E-10 | 1.70E-08 | up |
| IGLV3-9 | 146.9125 | 2.861613 | 0.463565 | 6.173052 | 6.70E-10 | 1.71E-08 | up |
| IGF2BP3 | 48.64463 | 1.742107 | 0.282798 | 6.160261 | 7.26E-10 | 1.84E-08 | up |
| MT2A | 977.0809 | 1.34391 | 0.218526 | 6.149899 | 7.75E-10 | 1.96E-08 | up |
| C21orf58 | 59.1239 | 1.015797 | 0.165193 | 6.149138 | 7.79E-10 | 1.96E-08 | up |
| NRGN | 2043.628 | 1.825444 | 0.297169 | 6.142789 | 8.11E-10 | 2.04E-08 | up |
| H2BC9 | 83.0813 | 2.376106 | 0.386994 | 6.13991 | 8.26E-10 | 2.07E-08 | up |
| H2BC5 | 76.3674 | 2.026869 | 0.330169 | 6.138878 | 8.31E-10 | 2.07E-08 | up |
| ZFHX4 | 5.921302 | 2.759981 | 0.449673 | 6.137756 | 8.37E-10 | 2.08E-08 | up |
| S100A12 | 3704.764 | 2.500972 | 0.408457 | 6.122973 | 9.18E-10 | 2.27E-08 | up |
| GRAP | 47.80153 | -1.04295 | 0.170504 | -6.11688 | 9.54E-10 | 2.35E-08 | down |
| MET | 15.9948 | 2.792286 | 0.45736 | 6.105229 | 1.03E-09 | 2.52E-08 | up |
| SPATS2L | 230.9898 | 1.473861 | 0.241689 | 6.098171 | 1.07E-09 | 2.62E-08 | up |
| IFIT1B | 145.2718 | 6.061954 | 0.996413 | 6.083779 | 1.17E-09 | 2.85E-08 | up |
| MMRN1 | 249.9473 | 2.199972 | 0.361771 | 6.081111 | 1.19E-09 | 2.89E-08 | up |
| RHOBTB1 | 190.7101 | 2.136544 | 0.35214 | 6.067312 | 1.30E-09 | 3.13E-08 | up |
| TRAJ33 | 7.600549 | -1.78079 | 0.293815 | -6.06091 | 1.35E-09 | 3.25E-08 | down |
| IGHV1-67 | 5.166613 | 4.387306 | 0.724283 | 6.057451 | 1.38E-09 | 3.30E-08 | up |
| PGRMC1 | 748.8175 | 1.06182 | 0.175569 | 6.047884 | 1.47E-09 | 3.49E-08 | up |
| IGHV1-3 | 294.8849 | 3.114155 | 0.51579 | 6.037647 | 1.56E-09 | 3.70E-08 | up |
| PEAR1 | 286.9951 | 2.128953 | 0.352959 | 6.031736 | 1.62E-09 | 3.81E-08 | up |
| IGKV3-20 | 3170.115 | 2.444665 | 0.406074 | 6.020243 | 1.74E-09 | 4.08E-08 | up |
| LTF | 10.42393 | 4.392313 | 0.729923 | 6.0175 | 1.77E-09 | 4.13E-08 | up |
| IFITM3 | 6778.362 | 2.084595 | 0.346464 | 6.016775 | 1.78E-09 | 4.14E-08 | up |
| SLC2A5 | 107.1255 | 1.754754 | 0.291979 | 6.009858 | 1.86E-09 | 4.32E-08 | up |
| IGHV3-13 | 274.7063 | 3.348959 | 0.557838 | 6.003462 | 1.93E-09 | 4.47E-08 | up |
| RMI2 | 82.57715 | 1.284411 | 0.21403 | 6.001078 | 1.96E-09 | 4.53E-08 | up |
| GP6 | 222.4111 | 2.097549 | 0.349617 | 5.999554 | 1.98E-09 | 4.56E-08 | up |
| PRKAR2B | 1291.485 | 1.913263 | 0.318909 | 5.999409 | 1.98E-09 | 4.56E-08 | up |
| ITGB5 | 609.8166 | 2.085341 | 0.347655 | 5.998302 | 1.99E-09 | 4.58E-08 | up |
| LAMC1 | 237.1263 | 1.617441 | 0.269799 | 5.994992 | 2.03E-09 | 4.67E-08 | up |
| ADGRD1 | 77.23096 | -1.32189 | 0.220811 | -5.98654 | 2.14E-09 | 4.89E-08 | down |
| HASPIN | 11.5707 | 1.825884 | 0.305099 | 5.984567 | 2.17E-09 | 4.95E-08 | up |
| HBM | 217.9705 | 6.436452 | 1.075842 | 5.982712 | 2.19E-09 | 4.99E-08 | up |
| SMIM5 | 131.5676 | 2.169638 | 0.362928 | 5.978146 | 2.26E-09 | 5.11E-08 | up |
| SLC6A8 | 81.64608 | 2.555924 | 0.42799 | 5.971919 | 2.34E-09 | 5.30E-08 | up |
| SAPCD2 | 143.5676 | 1.631596 | 0.273391 | 5.967997 | 2.40E-09 | 5.41E-08 | up |
| IGHV1OR15-9 | 3.984086 | 4.881491 | 0.818552 | 5.963572 | 2.47E-09 | 5.55E-08 | up |
| TMEM255B | 38.85464 | 1.59739 | 0.267903 | 5.962576 | 2.48E-09 | 5.57E-08 | up |
| SPTA1 | 19.5096 | 3.247914 | 0.54501 | 5.959363 | 2.53E-09 | 5.67E-08 | up |
| IGLV2-8 | 534.5345 | 2.359975 | 0.396288 | 5.955209 | 2.60E-09 | 5.77E-08 | up |
| LOC107986898 | 9.41133 | 2.322319 | 0.390249 | 5.950859 | 2.67E-09 | 5.91E-08 | up |
| CENPN | 82.92795 | 1.030335 | 0.173403 | 5.941863 | 2.82E-09 | 6.22E-08 | up |
| TMEM40 | 200.5417 | 2.102616 | 0.353915 | 5.941017 | 2.83E-09 | 6.24E-08 | up |
| MMP8 | 7.924678 | 4.334361 | 0.729771 | 5.939341 | 2.86E-09 | 6.28E-08 | up |
| LINC00964 | 5.138662 | 2.4988 | 0.420862 | 5.937333 | 2.90E-09 | 6.34E-08 | up |
| S100A8 | 42873.82 | 1.91293 | 0.322235 | 5.93645 | 2.91E-09 | 6.37E-08 | up |
| ESAM | 593.1839 | 1.996219 | 0.336321 | 5.935457 | 2.93E-09 | 6.40E-08 | up |
| FER1L4 | 23.28448 | 2.668786 | 0.450003 | 5.930595 | 3.02E-09 | 6.55E-08 | up |
| PCSK6 | 227.9599 | 2.272201 | 0.383583 | 5.923622 | 3.15E-09 | 6.82E-08 | up |
| GNG11 | 595.9626 | 1.763068 | 0.297673 | 5.922832 | 3.16E-09 | 6.84E-08 | up |
| SLC35D3 | 16.95497 | 3.071662 | 0.519695 | 5.91051 | 3.41E-09 | 7.33E-08 | up |
| RAB3IL1 | 11.62145 | 2.884804 | 0.488748 | 5.902435 | 3.58E-09 | 7.65E-08 | up |
| TNS1 | 286.0603 | 2.345625 | 0.397397 | 5.902467 | 3.58E-09 | 7.65E-08 | up |
| OTOF | 145.3443 | 2.878479 | 0.487719 | 5.901919 | 3.59E-09 | 7.66E-08 | up |
| FAXDC2 | 492.3484 | 1.993451 | 0.338187 | 5.894527 | 3.76E-09 | 7.97E-08 | up |
| MAGI2-AS3 | 37.89911 | 2.475294 | 0.420254 | 5.889995 | 3.86E-09 | 8.16E-08 | up |
| LINC01814 | 22.78005 | 1.218998 | 0.207104 | 5.885921 | 3.96E-09 | 8.35E-08 | up |
| ARHGAP6 | 94.62234 | 2.04315 | 0.347584 | 5.878146 | 4.15E-09 | 8.70E-08 | up |
| TMCC2 | 249.8436 | 2.021979 | 0.344018 | 5.877541 | 4.16E-09 | 8.71E-08 | up |
| MYLK | 520.7358 | 2.079186 | 0.35404 | 5.872746 | 4.29E-09 | 8.93E-08 | up |
| RGS6 | 67.59425 | 1.919084 | 0.326988 | 5.868978 | 4.38E-09 | 9.10E-08 | up |
| SEMA4C | 290.8234 | -1.06633 | 0.181771 | -5.86635 | 4.45E-09 | 9.21E-08 | down |
| GINS1 | 32.57103 | 1.609972 | 0.274558 | 5.863874 | 4.52E-09 | 9.32E-08 | up |
| DCHS1 | 97.63103 | -1.29376 | 0.220926 | -5.85607 | 4.74E-09 | 9.74E-08 | down |
| ASB15 | 7.707497 | 1.808408 | 0.309046 | 5.851575 | 4.87E-09 | 9.98E-08 | up |
| LINC00887 | 3.152973 | 2.94398 | 0.503155 | 5.851035 | 4.89E-09 | 1.00E-07 | up |
| IGLV8-61 | 374.6267 | 2.862212 | 0.489924 | 5.842159 | 5.15E-09 | 1.05E-07 | up |
| TRBV6-4 | 14.88058 | -1.5962 | 0.273308 | -5.8403 | 5.21E-09 | 1.06E-07 | down |
| IGHV3-64 | 102.2563 | 3.514506 | 0.602064 | 5.837428 | 5.30E-09 | 1.08E-07 | up |
| TMOD1 | 56.30424 | 2.313074 | 0.396733 | 5.830302 | 5.53E-09 | 1.12E-07 | up |
| HSPA1A | 470.1819 | -1.17978 | 0.202439 | -5.82785 | 5.61E-09 | 1.13E-07 | down |
| RADIL | 3.467619 | 2.852753 | 0.490145 | 5.820221 | 5.88E-09 | 1.18E-07 | up |
| CDCA7 | 183.2935 | 1.465107 | 0.251734 | 5.820059 | 5.88E-09 | 1.18E-07 | up |
| EME1 | 25.68543 | 1.080354 | 0.186195 | 5.802287 | 6.54E-09 | 1.31E-07 | up |
| IGLV1-36 | 74.34455 | 2.921322 | 0.504097 | 5.795159 | 6.83E-09 | 1.36E-07 | up |
| GYPA | 4.452076 | 5.396961 | 0.931591 | 5.79327 | 6.90E-09 | 1.37E-07 | up |
| LOC728975 | 96.38501 | 1.833018 | 0.316723 | 5.787444 | 7.15E-09 | 1.41E-07 | up |
| IGKV2D-30 | 26.00285 | 3.827904 | 0.661551 | 5.786257 | 7.20E-09 | 1.42E-07 | up |
| TOX2 | 77.06271 | 1.225928 | 0.211953 | 5.783953 | 7.30E-09 | 1.44E-07 | up |
| CTSA | 5542.949 | 1.140824 | 0.197268 | 5.783122 | 7.33E-09 | 1.44E-07 | up |
| CHST8 | 17.7423 | 2.672427 | 0.462308 | 5.780617 | 7.44E-09 | 1.46E-07 | up |
| CALD1 | 67.11336 | 2.129756 | 0.368549 | 5.778754 | 7.53E-09 | 1.47E-07 | up |
| TUBA1C | 232.3777 | 1.171699 | 0.203061 | 5.770183 | 7.92E-09 | 1.55E-07 | up |
| ZNF543 | 94.733 | -1.0852 | 0.188238 | -5.76504 | 8.16E-09 | 1.59E-07 | down |
| CCDC150 | 16.50907 | 1.48444 | 0.257726 | 5.75976 | 8.42E-09 | 1.64E-07 | up |
| CTTN | 236.9764 | 1.82009 | 0.3165 | 5.750673 | 8.89E-09 | 1.72E-07 | up |
| IGKV3-7 | 12.56016 | 2.881227 | 0.501141 | 5.749337 | 8.96E-09 | 1.73E-07 | up |
| KLC3 | 12.35445 | 4.589096 | 0.798613 | 5.746336 | 9.12E-09 | 1.76E-07 | up |
| RIPOR3 | 77.49499 | 1.492635 | 0.259935 | 5.742329 | 9.34E-09 | 1.80E-07 | up |
| CAVIN1 | 19.5063 | 1.822569 | 0.317882 | 5.73347 | 9.84E-09 | 1.89E-07 | up |
| TNFRSF13B | 246.6516 | 1.521106 | 0.265295 | 5.733647 | 9.83E-09 | 1.89E-07 | up |
| ACRBP | 659.2104 | 1.559724 | 0.272417 | 5.725494 | 1.03E-08 | 1.98E-07 | up |
| MESP1 | 11.07187 | 1.480835 | 0.259246 | 5.712075 | 1.12E-08 | 2.13E-07 | up |
| OR2W3 | 63.75825 | 2.588851 | 0.453632 | 5.706943 | 1.15E-08 | 2.19E-07 | up |
| SLC24A3 | 54.24276 | 2.30854 | 0.404529 | 5.706731 | 1.15E-08 | 2.19E-07 | up |
| HBQ1 | 28.08959 | 2.811912 | 0.493015 | 5.703503 | 1.17E-08 | 2.23E-07 | up |
| CD200R1 | 220.139 | -1.20596 | 0.211491 | -5.70215 | 1.18E-08 | 2.24E-07 | down |
| ADM | 859.3115 | 2.223765 | 0.390216 | 5.698812 | 1.21E-08 | 2.28E-07 | up |
| CABLES1 | 25.72919 | 1.563741 | 0.274427 | 5.698208 | 1.21E-08 | 2.28E-07 | up |
| COL24A1 | 31.83279 | 1.067845 | 0.187834 | 5.685035 | 1.31E-08 | 2.44E-07 | up |
| NLGN1 | 4.399029 | 2.435505 | 0.428684 | 5.681352 | 1.34E-08 | 2.49E-07 | up |
| MS4A4A | 686.253 | 1.748561 | 0.308073 | 5.675807 | 1.38E-08 | 2.57E-07 | up |
| LIPH | 23.69544 | 2.723089 | 0.480169 | 5.671105 | 1.42E-08 | 2.62E-07 | up |
| KCNA2 | 17.59373 | 1.205109 | 0.2125 | 5.671104 | 1.42E-08 | 2.62E-07 | up |
| TNFRSF12A | 89.29845 | 2.187484 | 0.385796 | 5.670047 | 1.43E-08 | 2.63E-07 | up |
| IGKJ4 | 18.17535 | 1.729463 | 0.305835 | 5.654888 | 1.56E-08 | 2.85E-07 | up |
| IGKV1D-8 | 81.59565 | 2.361376 | 0.418391 | 5.64394 | 1.66E-08 | 3.02E-07 | up |
| ECM1 | 19.25723 | 2.00318 | 0.355407 | 5.636291 | 1.74E-08 | 3.14E-07 | up |
| WASF1 | 112.4787 | 1.439195 | 0.25558 | 5.631096 | 1.79E-08 | 3.23E-07 | up |
| PLAC8 | 2819.346 | 1.044202 | 0.185552 | 5.627548 | 1.83E-08 | 3.29E-07 | up |
| FHL2 | 65.03055 | 1.948692 | 0.346547 | 5.623172 | 1.87E-08 | 3.36E-07 | up |
| OXTR | 15.80519 | 2.043466 | 0.363436 | 5.622624 | 1.88E-08 | 3.37E-07 | up |
| SPOCD1 | 82.27495 | 2.168117 | 0.386154 | 5.614637 | 1.97E-08 | 3.51E-07 | up |
| ELL2 | 618.4676 | 1.342944 | 0.239387 | 5.609937 | 2.02E-08 | 3.60E-07 | up |
| CCNE2 | 33.09031 | 1.286204 | 0.229346 | 5.608138 | 2.05E-08 | 3.63E-07 | up |
| KCP | 23.88232 | 1.290249 | 0.230128 | 5.606646 | 2.06E-08 | 3.66E-07 | up |
| WNT11 | 31.51729 | 2.204661 | 0.393625 | 5.600918 | 2.13E-08 | 3.76E-07 | up |
| GALNT5 | 4.464041 | 2.5894 | 0.462412 | 5.599767 | 2.15E-08 | 3.78E-07 | up |
| FSTL1 | 108.5717 | 2.146023 | 0.383279 | 5.599111 | 2.15E-08 | 3.79E-07 | up |
| FN1 | 176.0325 | 2.787491 | 0.49814 | 5.595792 | 2.20E-08 | 3.86E-07 | up |
| DHCR24 | 573.8136 | 1.310973 | 0.234303 | 5.595198 | 2.20E-08 | 3.87E-07 | up |
| LTBP1 | 392.1857 | 1.965701 | 0.351466 | 5.592861 | 2.23E-08 | 3.92E-07 | up |
| TJP1 | 3.50493 | 2.962491 | 0.529808 | 5.591629 | 2.25E-08 | 3.94E-07 | up |
| IGKV1-9 | 966.2388 | 2.798031 | 0.501104 | 5.583738 | 2.35E-08 | 4.11E-07 | up |
| S100A9 | 73429.57 | 1.662901 | 0.297921 | 5.581692 | 2.38E-08 | 4.15E-07 | up |
| ABCC13 | 9.900018 | 3.916235 | 0.702448 | 5.575127 | 2.47E-08 | 4.29E-07 | up |
| IGHV1-69 | 271.3382 | 2.816201 | 0.505575 | 5.570289 | 2.54E-08 | 4.40E-07 | up |
| HSPA1B | 149.0159 | -2.84199 | 0.510424 | -5.56789 | 2.58E-08 | 4.46E-07 | down |
| IGLV7-43 | 166.4979 | 2.277251 | 0.409404 | 5.562357 | 2.66E-08 | 4.57E-07 | up |
| NTRK3 | 5.895476 | 2.678176 | 0.481648 | 5.560448 | 2.69E-08 | 4.61E-07 | up |
| C2orf88 | 319.9282 | 1.728631 | 0.310879 | 5.560459 | 2.69E-08 | 4.61E-07 | up |
| IGHM | 33900.36 | 1.540082 | 0.277023 | 5.559397 | 2.71E-08 | 4.64E-07 | up |
| GP1BA | 308.9671 | 1.907835 | 0.343226 | 5.558533 | 2.72E-08 | 4.65E-07 | up |
| COL23A1 | 18.10111 | 1.778757 | 0.320007 | 5.558499 | 2.72E-08 | 4.65E-07 | up |
| IGHV1-45 | 78.53625 | 4.709132 | 0.847891 | 5.553932 | 2.79E-08 | 4.75E-07 | up |
| OR2B6 | 10.44735 | 3.678835 | 0.662593 | 5.552175 | 2.82E-08 | 4.79E-07 | up |
| RPS15AP27 | 2.705839 | -2.92151 | 0.526241 | -5.55166 | 2.83E-08 | 4.80E-07 | down |
| PRKG1 | 13.65022 | 2.192242 | 0.39499 | 5.550125 | 2.85E-08 | 4.83E-07 | up |
| OTOGL | 3.299512 | 3.237685 | 0.584258 | 5.541536 | 3.00E-08 | 5.05E-07 | up |
| SPX | 83.22174 | 1.96392 | 0.354917 | 5.533456 | 3.14E-08 | 5.28E-07 | up |
| GRIN2A | 4.235706 | 3.292854 | 0.596089 | 5.524102 | 3.31E-08 | 5.53E-07 | up |
| AHSP | 170.1534 | 6.050768 | 1.095859 | 5.521486 | 3.36E-08 | 5.60E-07 | up |
| LANCL3 | 42.14854 | 1.671337 | 0.303112 | 5.513927 | 3.51E-08 | 5.81E-07 | up |
| IGLV5-48 | 12.50497 | 3.347651 | 0.607216 | 5.513116 | 3.53E-08 | 5.83E-07 | up |
| BEX3 | 385.215 | 1.304454 | 0.236694 | 5.511132 | 3.57E-08 | 5.89E-07 | up |
| LOC101927533 | 3.392134 | 2.405702 | 0.436881 | 5.506534 | 3.66E-08 | 6.03E-07 | up |
| LILRP2 | 20.486 | 2.208896 | 0.401791 | 5.497625 | 3.85E-08 | 6.31E-07 | up |
| MIR34AHG | 6.957022 | 1.685609 | 0.306742 | 5.495208 | 3.90E-08 | 6.37E-07 | up |
| PTCRA | 155.7883 | 1.667136 | 0.303373 | 5.49533 | 3.90E-08 | 6.37E-07 | up |
| ITGA7 | 47.67949 | 1.791205 | 0.326023 | 5.494104 | 3.93E-08 | 6.40E-07 | up |
| PTPRT | 3.35282 | 2.795041 | 0.50892 | 5.492101 | 3.97E-08 | 6.45E-07 | up |
| IGHV4-4 | 263.0567 | 3.811081 | 0.693987 | 5.491573 | 3.98E-08 | 6.45E-07 | up |
| IGHJ5 | 23.30258 | 1.685404 | 0.307001 | 5.489889 | 4.02E-08 | 6.51E-07 | up |
| DEPP1 | 22.98164 | 2.549046 | 0.464789 | 5.484309 | 4.15E-08 | 6.71E-07 | up |
| SIGLEC11 | 38.61304 | 2.339358 | 0.42787 | 5.467451 | 4.57E-08 | 7.30E-07 | up |
| ADAMTS10 | 240.7763 | -1.10639 | 0.20274 | -5.45719 | 4.84E-08 | 7.68E-07 | down |
| ANKRD9 | 231.9066 | 1.530681 | 0.280551 | 5.455981 | 4.87E-08 | 7.72E-07 | up |
| ACSS3 | 8.560946 | 1.663239 | 0.305092 | 5.451606 | 4.99E-08 | 7.90E-07 | up |
| TARM1 | 20.00917 | 3.27929 | 0.601579 | 5.451136 | 5.00E-08 | 7.91E-07 | up |
| GMNN | 155.6279 | 1.107714 | 0.203228 | 5.450582 | 5.02E-08 | 7.92E-07 | up |
| PROS1 | 148.573 | 2.181958 | 0.400455 | 5.4487 | 5.07E-08 | 7.99E-07 | up |
| GFI1B | 285.2313 | 1.725536 | 0.316914 | 5.444812 | 5.19E-08 | 8.16E-07 | up |
| BFSP2 | 13.32044 | 1.615321 | 0.297207 | 5.435007 | 5.48E-08 | 8.58E-07 | up |
| KEL | 12.15735 | 2.290074 | 0.422026 | 5.42638 | 5.75E-08 | 8.98E-07 | up |
| ITGA8 | 4.860143 | 2.943201 | 0.542702 | 5.423238 | 5.85E-08 | 9.12E-07 | up |
| SLC4A11 | 18.72714 | 1.71492 | 0.316513 | 5.418161 | 6.02E-08 | 9.36E-07 | up |
| MPP1 | 2488.217 | 1.083388 | 0.20017 | 5.412351 | 6.22E-08 | 9.62E-07 | up |
| SLC8A3 | 24.40184 | 2.185028 | 0.404491 | 5.401923 | 6.59E-08 | 1.01E-06 | up |
| ZNF385D | 17.38841 | 2.26752 | 0.419924 | 5.399836 | 6.67E-08 | 1.02E-06 | up |
| RNF17 | 11.89021 | 4.717376 | 0.873763 | 5.398917 | 6.70E-08 | 1.03E-06 | up |
| PTPRN | 24.26457 | 2.045877 | 0.379384 | 5.392625 | 6.94E-08 | 1.06E-06 | up |
| SIAE | 164.9768 | 1.181862 | 0.219237 | 5.390796 | 7.01E-08 | 1.07E-06 | up |
| TMEM163 | 16.33835 | 1.211893 | 0.225038 | 5.385272 | 7.23E-08 | 1.10E-06 | up |
| COL10A1 | 18.16922 | 2.443746 | 0.454015 | 5.382527 | 7.34E-08 | 1.11E-06 | up |
| LY6G6F | 17.1242 | 1.943072 | 0.36135 | 5.377256 | 7.56E-08 | 1.14E-06 | up |
| IGHJ6 | 19.47491 | 1.59342 | 0.296329 | 5.3772 | 7.57E-08 | 1.14E-06 | up |
| LOC101929698 | 21.2062 | -1.81952 | 0.3385 | -5.37525 | 7.65E-08 | 1.15E-06 | down |
| C1QA | 396.5708 | 1.658336 | 0.308739 | 5.371315 | 7.82E-08 | 1.18E-06 | up |
| ZNF239 | 21.58097 | -1.13659 | 0.211659 | -5.3699 | 7.88E-08 | 1.18E-06 | down |
| H4C12 | 8.426456 | 1.265902 | 0.235811 | 5.368279 | 7.95E-08 | 1.19E-06 | up |
| PLXNB3 | 61.37995 | 3.011666 | 0.561092 | 5.36751 | 7.98E-08 | 1.20E-06 | up |
| CDKN2A | 42.03755 | 1.143787 | 0.213282 | 5.362782 | 8.19E-08 | 1.22E-06 | up |
| SLC25A37 | 1139.754 | 1.449987 | 0.27043 | 5.361779 | 8.24E-08 | 1.23E-06 | up |
| MAPK10 | 17.02684 | 1.608319 | 0.300016 | 5.360771 | 8.29E-08 | 1.23E-06 | up |
| CAVIN2 | 2142.674 | 1.619022 | 0.302761 | 5.347521 | 8.92E-08 | 1.32E-06 | up |
| CD109 | 50.2289 | 1.444629 | 0.270302 | 5.344504 | 9.07E-08 | 1.34E-06 | up |
| ARFGEF3 | 4.772197 | 2.931862 | 0.548685 | 5.343431 | 9.12E-08 | 1.34E-06 | up |
| MEIS1 | 120.1623 | 1.503309 | 0.281483 | 5.340676 | 9.26E-08 | 1.36E-06 | up |
| CLEC1B | 165.0172 | 1.859741 | 0.348459 | 5.337037 | 9.45E-08 | 1.38E-06 | up |
| PCYT1B | 53.10007 | 2.452727 | 0.459834 | 5.333935 | 9.61E-08 | 1.41E-06 | up |
| CPE | 11.15391 | 3.528447 | 0.661561 | 5.333516 | 9.63E-08 | 1.41E-06 | up |
| SLCO5A1 | 14.94669 | 1.391515 | 0.26091 | 5.333305 | 9.64E-08 | 1.41E-06 | up |
| MMD | 940.5426 | 1.512502 | 0.28391 | 5.327394 | 9.96E-08 | 1.45E-06 | up |
| LINC00534 | 20.41809 | 2.055044 | 0.386048 | 5.323287 | 1.02E-07 | 1.48E-06 | up |
| PARD3 | 100.4362 | 1.725548 | 0.324255 | 5.321586 | 1.03E-07 | 1.49E-06 | up |
| SH3BGRL2 | 580.3125 | 1.733628 | 0.326073 | 5.316689 | 1.06E-07 | 1.53E-06 | up |
| DYNLT5 | 8.716091 | 2.740792 | 0.515911 | 5.312525 | 1.08E-07 | 1.56E-06 | up |
| IGHJ3P | 31.59318 | 1.556506 | 0.293445 | 5.304257 | 1.13E-07 | 1.62E-06 | up |
| KAZN | 8.26134 | 2.155163 | 0.406415 | 5.302864 | 1.14E-07 | 1.63E-06 | up |
| CNKSR3 | 8.267929 | 2.456896 | 0.463703 | 5.298426 | 1.17E-07 | 1.66E-06 | up |
| RBP4 | 3.859713 | 3.795898 | 0.716492 | 5.297895 | 1.17E-07 | 1.66E-06 | up |
| HYDIN | 7.138024 | 1.848315 | 0.348892 | 5.29767 | 1.17E-07 | 1.66E-06 | up |
| IGHG2 | 5138.727 | 2.146216 | 0.405576 | 5.291775 | 1.21E-07 | 1.71E-06 | up |
| IGLVI-70 | 30.97494 | 2.912635 | 0.550607 | 5.289863 | 1.22E-07 | 1.73E-06 | up |
| ELANE | 39.64326 | 2.798586 | 0.529331 | 5.287024 | 1.24E-07 | 1.75E-06 | up |
| DGKI | 8.647263 | 2.946912 | 0.559042 | 5.271363 | 1.35E-07 | 1.88E-06 | up |
| ALOX12 | 255.5334 | 1.466405 | 0.278518 | 5.265031 | 1.40E-07 | 1.94E-06 | up |
| ADAMTS5 | 22.17581 | -2.23143 | 0.424424 | -5.25755 | 1.46E-07 | 2.01E-06 | down |
| EBI3 | 24.08516 | 1.253188 | 0.238397 | 5.256723 | 1.47E-07 | 2.02E-06 | up |
| DNM1 | 60.32582 | 1.542208 | 0.293482 | 5.254869 | 1.48E-07 | 2.03E-06 | up |
| FCRL4 | 3.692278 | 3.074179 | 0.585307 | 5.252247 | 1.50E-07 | 2.05E-06 | up |
| IGLV2-18 | 124.7176 | 3.137403 | 0.597442 | 5.25139 | 1.51E-07 | 2.06E-06 | up |
| CD5L | 3.35783 | 3.432555 | 0.653694 | 5.251013 | 1.51E-07 | 2.06E-06 | up |
| IGHV1-58 | 52.19431 | 2.424168 | 0.461668 | 5.250888 | 1.51E-07 | 2.06E-06 | up |
| CETP | 43.27495 | 1.821162 | 0.347142 | 5.24616 | 1.55E-07 | 2.11E-06 | up |
| EHD2 | 16.2061 | 2.05666 | 0.393606 | 5.225177 | 1.74E-07 | 2.34E-06 | up |
| PKD1L2 | 5.223657 | 2.158602 | 0.413149 | 5.224748 | 1.74E-07 | 2.34E-06 | up |
| PIEZO2 | 4.443966 | 2.778789 | 0.531932 | 5.223955 | 1.75E-07 | 2.35E-06 | up |
| ADORA1 | 14.54332 | -2.48678 | 0.476125 | -5.22297 | 1.76E-07 | 2.36E-06 | down |
| CCNE1 | 122.1373 | 1.074073 | 0.205676 | 5.222167 | 1.77E-07 | 2.37E-06 | up |
| DENND5B | 215.5649 | 1.049635 | 0.201083 | 5.219914 | 1.79E-07 | 2.40E-06 | up |
| ZNF563 | 87.56619 | -1.03521 | 0.198498 | -5.21521 | 1.84E-07 | 2.45E-06 | down |
| SMTN | 131.2033 | 1.145726 | 0.220002 | 5.207792 | 1.91E-07 | 2.54E-06 | up |
| JSRP1 | 18.7085 | 1.717415 | 0.330132 | 5.2022 | 1.97E-07 | 2.61E-06 | up |
| LOC105372240 | 18.7085 | 1.717415 | 0.330132 | 5.2022 | 1.97E-07 | 2.61E-06 | up |
| EHD3 | 794.6404 | 1.510223 | 0.290594 | 5.197023 | 2.03E-07 | 2.68E-06 | up |
| DENND5B-AS1 | 3.899757 | 1.896979 | 0.365263 | 5.193466 | 2.06E-07 | 2.73E-06 | up |
| LINC02777 | 8.018072 | 1.411409 | 0.271802 | 5.192781 | 2.07E-07 | 2.74E-06 | up |
| TRPM6 | 3.56855 | 2.532182 | 0.488187 | 5.186905 | 2.14E-07 | 2.82E-06 | up |
| TP53I3 | 240.0965 | 1.142849 | 0.220345 | 5.186637 | 2.14E-07 | 2.82E-06 | up |
| RHEX | 6.574857 | 1.802563 | 0.347797 | 5.182797 | 2.19E-07 | 2.87E-06 | up |
| GPR132 | 959.4949 | -1.11643 | 0.21547 | -5.18139 | 2.20E-07 | 2.89E-06 | down |
| LINC00877 | 1235.135 | -1.14001 | 0.220037 | -5.181 | 2.21E-07 | 2.89E-06 | down |
| SLCO2B1 | 11.70994 | 2.345385 | 0.452779 | 5.179974 | 2.22E-07 | 2.91E-06 | up |
| SYN2 | 11.23505 | 2.533173 | 0.489533 | 5.17467 | 2.28E-07 | 2.99E-06 | up |
| F2RL3 | 50.68061 | 1.74625 | 0.337589 | 5.172706 | 2.31E-07 | 3.02E-06 | up |
| LOC105375743 | 12.47723 | 1.42449 | 0.275799 | 5.164949 | 2.41E-07 | 3.14E-06 | up |
| WDR5B | 40.72542 | -1.0206 | 0.197648 | -5.16372 | 2.42E-07 | 3.15E-06 | down |
| LINC00923 | 5.147883 | 1.84542 | 0.357771 | 5.158098 | 2.49E-07 | 3.24E-06 | up |
| IGKV1D-13 | 237.3125 | 4.076815 | 0.790793 | 5.155349 | 2.53E-07 | 3.28E-06 | up |
| TMEM45A | 8.949823 | 2.172944 | 0.421645 | 5.15349 | 2.56E-07 | 3.31E-06 | up |
| PCOLCE2 | 4.227984 | 4.604866 | 0.893655 | 5.152844 | 2.57E-07 | 3.32E-06 | up |
| SCD | 226.8004 | 1.12542 | 0.218651 | 5.147103 | 2.65E-07 | 3.41E-06 | up |
| CENPI | 13.27315 | 1.468015 | 0.285269 | 5.146073 | 2.66E-07 | 3.42E-06 | up |
| MYBPC1 | 4.052088 | 2.344082 | 0.456613 | 5.133631 | 2.84E-07 | 3.62E-06 | up |
| ADGRE4P | 39.86871 | -1.38159 | 0.269239 | -5.13148 | 2.87E-07 | 3.66E-06 | down |
| AP1M2 | 15.47945 | 1.994644 | 0.388831 | 5.129848 | 2.90E-07 | 3.69E-06 | up |
| PSME2P1 | 42.93948 | 1.241791 | 0.242137 | 5.12846 | 2.92E-07 | 3.71E-06 | up |
| IGHG4 | 1693.192 | 2.56926 | 0.501393 | 5.124247 | 2.99E-07 | 3.79E-06 | up |
| MTURN | 298.4564 | 1.351223 | 0.263859 | 5.12101 | 3.04E-07 | 3.84E-06 | up |
| POU2AF1 | 370.158 | 1.191151 | 0.233008 | 5.112053 | 3.19E-07 | 4.00E-06 | up |
| SMIM24 | 10.58049 | 2.394959 | 0.468584 | 5.111056 | 3.20E-07 | 4.02E-06 | up |
| EDNRB | 11.09978 | 2.884201 | 0.564395 | 5.110254 | 3.22E-07 | 4.03E-06 | up |
| TUBB1 | 4246.194 | 1.640616 | 0.321051 | 5.11014 | 3.22E-07 | 4.03E-06 | up |
| MMP2 | 13.78411 | 3.395903 | 0.665112 | 5.105764 | 3.29E-07 | 4.11E-06 | up |
| TAGAP | 5205.494 | -1.61651 | 0.316765 | -5.10318 | 3.34E-07 | 4.16E-06 | down |
| CRYM | 23.20795 | 2.016306 | 0.395449 | 5.098778 | 3.42E-07 | 4.24E-06 | up |
| GRB10 | 186.5059 | 1.596208 | 0.3131 | 5.098072 | 3.43E-07 | 4.26E-06 | up |
| MACC1 | 11.08989 | 1.275027 | 0.250295 | 5.094089 | 3.50E-07 | 4.33E-06 | up |
| IL21-AS1 | 5.848911 | 2.437764 | 0.478675 | 5.092728 | 3.53E-07 | 4.36E-06 | up |
| SLC25A39 | 3088.604 | 1.372657 | 0.269607 | 5.091321 | 3.56E-07 | 4.39E-06 | up |
| DQX1 | 6.846481 | 1.898373 | 0.373018 | 5.089231 | 3.60E-07 | 4.43E-06 | up |
| ZNF571 | 97.41492 | -1.05069 | 0.20665 | -5.08439 | 3.69E-07 | 4.53E-06 | down |
| ANG | 43.99442 | 1.38695 | 0.273061 | 5.079267 | 3.79E-07 | 4.65E-06 | up |
| HBB | 101191.1 | 4.836088 | 0.952171 | 5.07901 | 3.79E-07 | 4.65E-06 | up |
| LRP2 | 5.127757 | 2.61605 | 0.515311 | 5.07664 | 3.84E-07 | 4.70E-06 | up |
| IGKJ3 | 12.01504 | 1.622774 | 0.319674 | 5.076338 | 3.85E-07 | 4.70E-06 | up |
| CRIP2 | 365.4186 | -1.36594 | 0.269114 | -5.07569 | 3.86E-07 | 4.72E-06 | down |
| STRADB | 671.3617 | 1.092242 | 0.215427 | 5.070135 | 3.98E-07 | 4.82E-06 | up |
| IGKV1-39 | 3.151891 | 3.184167 | 0.628749 | 5.064291 | 4.10E-07 | 4.95E-06 | up |
| IGHV3-65 | 5.841834 | 2.763884 | 0.546555 | 5.056917 | 4.26E-07 | 5.12E-06 | up |
| DUX4L19 | 3.384867 | 2.885197 | 0.570787 | 5.054773 | 4.31E-07 | 5.17E-06 | up |
| ITGA9-AS1 | 12.867 | 1.492155 | 0.295314 | 5.052773 | 4.35E-07 | 5.21E-06 | up |
| SPHK1 | 481.9868 | 1.227175 | 0.243035 | 5.049383 | 4.43E-07 | 5.29E-06 | up |
| H4C11 | 8.94913 | 1.286465 | 0.25494 | 5.046144 | 4.51E-07 | 5.37E-06 | up |
| F13A1 | 9318.847 | 1.461125 | 0.289581 | 5.045656 | 4.52E-07 | 5.38E-06 | up |
| CREB3L1 | 5.041325 | 3.006666 | 0.597464 | 5.032382 | 4.84E-07 | 5.72E-06 | up |
| PART1 | 14.69487 | -1.02052 | 0.202908 | -5.02947 | 4.92E-07 | 5.79E-06 | down |
| PLAAT1 | 15.21939 | 1.969275 | 0.391838 | 5.025732 | 5.02E-07 | 5.89E-06 | up |
| DUX4L26 | 9.812776 | 2.25062 | 0.447906 | 5.024765 | 5.04E-07 | 5.91E-06 | up |
| CTSD | 3801.071 | 1.202104 | 0.239703 | 5.014978 | 5.30E-07 | 6.19E-06 | up |
| LOC101928834 | 26.64077 | 1.911335 | 0.381279 | 5.01295 | 5.36E-07 | 6.25E-06 | up |
| PTGS1 | 2325.983 | 1.4239 | 0.284417 | 5.006385 | 5.55E-07 | 6.45E-06 | up |
| KIR2DL4 | 90.54678 | 1.371958 | 0.274106 | 5.005217 | 5.58E-07 | 6.48E-06 | up |
| ZNF597 | 87.69813 | -1.0032 | 0.20047 | -5.00422 | 5.61E-07 | 6.51E-06 | down |
| NHS | 40.18352 | -1.03139 | 0.206261 | -5.0004 | 5.72E-07 | 6.62E-06 | down |
| ZNF540 | 64.29646 | -1.12737 | 0.225451 | -5.00051 | 5.72E-07 | 6.62E-06 | down |
| AQP10 | 73.00679 | 1.882861 | 0.37757 | 4.986793 | 6.14E-07 | 7.08E-06 | up |
| ARHGEF12 | 372.3711 | 1.231094 | 0.247166 | 4.98084 | 6.33E-07 | 7.27E-06 | up |
| ADCY2 | 4.543609 | 2.068019 | 0.415253 | 4.980146 | 6.35E-07 | 7.29E-06 | up |
| PLS3 | 23.74592 | 1.638992 | 0.329256 | 4.977859 | 6.43E-07 | 7.37E-06 | up |
| IGKV1-13 | 5.227917 | 3.19187 | 0.64158 | 4.975014 | 6.52E-07 | 7.46E-06 | up |
| VEPH1 | 39.71458 | 1.760615 | 0.354335 | 4.968788 | 6.74E-07 | 7.68E-06 | up |
| FAH | 247.8518 | 1.094305 | 0.220331 | 4.966637 | 6.81E-07 | 7.76E-06 | up |
| H2AC16 | 12.78398 | 1.388267 | 0.27959 | 4.965372 | 6.86E-07 | 7.80E-06 | up |
| SSC4D | 17.25642 | 1.436136 | 0.289502 | 4.960712 | 7.02E-07 | 7.97E-06 | up |
| IGHV3-73 | 141.3454 | 2.197491 | 0.443348 | 4.956578 | 7.17E-07 | 8.12E-06 | up |
| CNKSR1 | 60.69017 | 1.305561 | 0.263509 | 4.954516 | 7.25E-07 | 8.20E-06 | up |
| SYTL4 | 49.34938 | 1.992795 | 0.403238 | 4.941983 | 7.73E-07 | 8.66E-06 | up |
| IL23R | 23.20386 | -1.84074 | 0.372517 | -4.94137 | 7.76E-07 | 8.67E-06 | down |
| DNAJC5B | 6.022945 | 2.25951 | 0.457389 | 4.940022 | 7.81E-07 | 8.73E-06 | up |
| CPNE5 | 618.6312 | 1.075463 | 0.217828 | 4.937202 | 7.93E-07 | 8.84E-06 | up |
| LOC100288637 | 19.90094 | 1.017485 | 0.206148 | 4.935693 | 7.99E-07 | 8.91E-06 | up |
| H3C7 | 3.391215 | 2.204937 | 0.447133 | 4.931274 | 8.17E-07 | 9.09E-06 | up |
| CSPG4 | 4.205151 | 2.110243 | 0.428042 | 4.929987 | 8.22E-07 | 9.13E-06 | up |
| KIR3DX1 | 40.49049 | 1.475214 | 0.299728 | 4.921842 | 8.57E-07 | 9.45E-06 | up |
| RNASE3 | 62.18496 | 2.69705 | 0.549049 | 4.912221 | 9.01E-07 | 9.85E-06 | up |
| LOC107984360 | 163.9003 | 1.748392 | 0.356783 | 4.900431 | 9.56E-07 | 1.04E-05 | up |
| TLL2 | 4.394988 | 1.888082 | 0.385774 | 4.894271 | 9.87E-07 | 1.07E-05 | up |
| TMEM272 | 20.50217 | -1.36661 | 0.279373 | -4.8917 | 1.00E-06 | 1.08E-05 | down |
| CXCL5 | 100.0603 | 2.044398 | 0.418133 | 4.889349 | 1.01E-06 | 1.09E-05 | up |
| GCSAML | 12.95708 | 2.05427 | 0.420493 | 4.885378 | 1.03E-06 | 1.11E-05 | up |
| INAVA | 3.357076 | 2.508577 | 0.513618 | 4.884131 | 1.04E-06 | 1.11E-05 | up |
| APOBEC3A | 3174.73 | 1.293385 | 0.265253 | 4.876039 | 1.08E-06 | 1.16E-05 | up |
| APOBEC3A_B | 3174.73 | 1.293385 | 0.265253 | 4.876039 | 1.08E-06 | 1.16E-05 | up |
| PCLO | 4.193896 | 2.471891 | 0.50707 | 4.874855 | 1.09E-06 | 1.16E-05 | up |
| PARM1 | 157.4497 | 1.013551 | 0.207935 | 4.87436 | 1.09E-06 | 1.16E-05 | up |
| SLPI | 7.675055 | 2.147105 | 0.440515 | 4.874079 | 1.09E-06 | 1.16E-05 | up |
| ANO2 | 7.170464 | 2.851376 | 0.585059 | 4.873656 | 1.10E-06 | 1.17E-05 | up |
| RETN | 732.696 | 2.508335 | 0.514721 | 4.873198 | 1.10E-06 | 1.17E-05 | up |
| ARHGAP23 | 14.347 | 1.112651 | 0.228324 | 4.873135 | 1.10E-06 | 1.17E-05 | up |
| SLC4A5 | 6.401965 | -1.20419 | 0.247275 | -4.86984 | 1.12E-06 | 1.18E-05 | down |
| TCN2 | 1086.155 | 1.451662 | 0.298223 | 4.867707 | 1.13E-06 | 1.20E-05 | up |
| DOCK1 | 34.31044 | 1.302302 | 0.267597 | 4.866655 | 1.14E-06 | 1.20E-05 | up |
| HBA1 | 1527.735 | 4.610563 | 0.948267 | 4.862091 | 1.16E-06 | 1.23E-05 | up |
| IGKJ5 | 21.66413 | 1.637711 | 0.337011 | 4.859522 | 1.18E-06 | 1.24E-05 | up |
| STON2 | 190.431 | 1.690548 | 0.347893 | 4.859393 | 1.18E-06 | 1.24E-05 | up |
| GPR18 | 371.3526 | -1.45579 | 0.30043 | -4.84568 | 1.26E-06 | 1.32E-05 | down |
| RTN1 | 1118.407 | -1.26291 | 0.260894 | -4.84069 | 1.29E-06 | 1.35E-05 | down |
| KBTBD12 | 5.123258 | 2.282701 | 0.471758 | 4.838712 | 1.31E-06 | 1.36E-05 | up |
| ANK1 | 429.3062 | 1.81359 | 0.374842 | 4.838276 | 1.31E-06 | 1.36E-05 | up |
| GATA2 | 85.4944 | 1.290191 | 0.266723 | 4.837198 | 1.32E-06 | 1.37E-05 | up |
| ZNF10 | 116.4506 | -1.05422 | 0.218623 | -4.82208 | 1.42E-06 | 1.47E-05 | down |
| PTGER3 | 9.051235 | 2.174655 | 0.451115 | 4.820625 | 1.43E-06 | 1.48E-05 | up |
| AMPH | 39.20493 | 1.780855 | 0.369483 | 4.819857 | 1.44E-06 | 1.48E-05 | up |
| MUC5AC | 5.168104 | 2.737885 | 0.568351 | 4.817241 | 1.46E-06 | 1.50E-05 | up |
| LOC101928932 | 4.292203 | 4.072784 | 0.845504 | 4.816988 | 1.46E-06 | 1.50E-05 | up |
| SLC1A3 | 56.41701 | 1.948185 | 0.404587 | 4.815241 | 1.47E-06 | 1.51E-05 | up |
| PDE6H | 8.75714 | 1.282277 | 0.266587 | 4.809978 | 1.51E-06 | 1.54E-05 | up |
| GPR174 | 318.118 | -1.02212 | 0.212796 | -4.80328 | 1.56E-06 | 1.59E-05 | down |
| LOC101927745 | 9.600758 | 1.929656 | 0.40186 | 4.801817 | 1.57E-06 | 1.60E-05 | up |
| LOC105369302 | 9.600758 | 1.929656 | 0.40186 | 4.801817 | 1.57E-06 | 1.60E-05 | up |
| CROCC2 | 50.00623 | -2.00711 | 0.418012 | -4.80156 | 1.57E-06 | 1.60E-05 | down |
| CLDN5 | 325.1028 | 1.669567 | 0.347778 | 4.800667 | 1.58E-06 | 1.60E-05 | up |
| FLRT2 | 7.062022 | 2.697166 | 0.561968 | 4.799503 | 1.59E-06 | 1.61E-05 | up |
| POGLUT2 | 18.55359 | 1.227755 | 0.255848 | 4.79876 | 1.60E-06 | 1.62E-05 | up |
| IGKV3D-15 | 14.03502 | 2.807642 | 0.585297 | 4.796948 | 1.61E-06 | 1.63E-05 | up |
| ELOVL4 | 21.63973 | -1.49733 | 0.31221 | -4.79592 | 1.62E-06 | 1.64E-05 | down |
| ZBTB32 | 201.4828 | 1.534174 | 0.3199 | 4.795796 | 1.62E-06 | 1.64E-05 | up |
| TF | 7.459713 | 1.573761 | 0.32822 | 4.79484 | 1.63E-06 | 1.64E-05 | up |
| CCR1 | 2455.914 | 1.100214 | 0.229571 | 4.792478 | 1.65E-06 | 1.66E-05 | up |
| CFAP54 | 13.91538 | 1.306632 | 0.272855 | 4.788741 | 1.68E-06 | 1.69E-05 | up |
| CTDSPL | 205.8858 | 1.455825 | 0.30426 | 4.784799 | 1.71E-06 | 1.72E-05 | up |
| HROB | 35.4836 | 1.195154 | 0.249934 | 4.78188 | 1.74E-06 | 1.74E-05 | up |
| PKHD1 | 4.510083 | 2.462062 | 0.515244 | 4.778436 | 1.77E-06 | 1.77E-05 | up |
| IGKV2D-24 | 13.20265 | 3.551968 | 0.744463 | 4.771178 | 1.83E-06 | 1.82E-05 | up |
| IGHV7-4-1 | 626.8917 | 5.097999 | 1.068874 | 4.769502 | 1.85E-06 | 1.84E-05 | up |
| FFAR2 | 319.3651 | 1.319107 | 0.276653 | 4.768094 | 1.86E-06 | 1.84E-05 | up |
| LEKR1 | 47.83481 | -1.13968 | 0.239093 | -4.76669 | 1.87E-06 | 1.86E-05 | down |
| SULT1C4 | 7.674306 | 2.471461 | 0.518588 | 4.765753 | 1.88E-06 | 1.86E-05 | up |
| PHLDA3 | 25.0905 | 1.422959 | 0.298583 | 4.765703 | 1.88E-06 | 1.86E-05 | up |
| AMIGO1 | 249.3197 | -1.05225 | 0.22079 | -4.76585 | 1.88E-06 | 1.86E-05 | down |
| HLX | 207.7283 | 1.470523 | 0.308775 | 4.762445 | 1.91E-06 | 1.89E-05 | up |
| ACOX2 | 31.42118 | 1.848762 | 0.388274 | 4.761486 | 1.92E-06 | 1.90E-05 | up |
| H2AC17 | 3.660419 | 1.902409 | 0.399668 | 4.759972 | 1.94E-06 | 1.91E-05 | up |
| FCGR1A | 308.959 | 1.527348 | 0.321261 | 4.754228 | 1.99E-06 | 1.95E-05 | up |
| IGKV1D-43 | 46.64354 | 3.376933 | 0.710775 | 4.751055 | 2.02E-06 | 1.98E-05 | up |
| PTPRF | 26.17183 | 1.9756 | 0.416552 | 4.742746 | 2.11E-06 | 2.06E-05 | up |
| TNNC2 | 17.84348 | 1.274614 | 0.269191 | 4.734984 | 2.19E-06 | 2.12E-05 | up |
| SLC14A2 | 8.623635 | 1.181386 | 0.249592 | 4.733273 | 2.21E-06 | 2.14E-05 | up |
| SPIC | 4.104895 | 3.063248 | 0.647284 | 4.732461 | 2.22E-06 | 2.14E-05 | up |
| ZNF521 | 24.54501 | 1.793888 | 0.379182 | 4.730939 | 2.23E-06 | 2.15E-05 | up |
| TAL1 | 89.58522 | 1.559319 | 0.329696 | 4.729561 | 2.25E-06 | 2.17E-05 | up |
| STOX2 | 3.37902 | 2.395946 | 0.506976 | 4.725958 | 2.29E-06 | 2.20E-05 | up |
| BPGM | 731.525 | 1.223951 | 0.259015 | 4.725398 | 2.30E-06 | 2.20E-05 | up |
| RBKS | 186.0363 | -1.10756 | 0.234385 | -4.72539 | 2.30E-06 | 2.20E-05 | down |
| KREMEN1 | 34.49637 | 1.864065 | 0.394619 | 4.723709 | 2.32E-06 | 2.22E-05 | up |
| SEZ6L | 69.34306 | -1.23349 | 0.261123 | -4.72379 | 2.31E-06 | 2.22E-05 | down |
| IGKV2-29 | 198.4548 | 2.83691 | 0.600796 | 4.721915 | 2.34E-06 | 2.24E-05 | up |
| SNX7 | 20.28536 | 2.397862 | 0.508068 | 4.719569 | 2.36E-06 | 2.26E-05 | up |
| FFAR1 | 6.505421 | 2.305424 | 0.489136 | 4.713259 | 2.44E-06 | 2.32E-05 | up |
| AR | 11.69247 | 1.590169 | 0.337392 | 4.713123 | 2.44E-06 | 2.32E-05 | up |
| H2BC13 | 5.323858 | 1.998223 | 0.425508 | 4.696092 | 2.65E-06 | 2.51E-05 | up |
| HEMGN | 108.428 | 1.624476 | 0.345917 | 4.69614 | 2.65E-06 | 2.51E-05 | up |
| XIRP2 | 14.94153 | 2.52594 | 0.53834 | 4.692094 | 2.70E-06 | 2.56E-05 | up |
| IGHV2-70D | 110.8039 | 3.355463 | 0.715635 | 4.68879 | 2.75E-06 | 2.59E-05 | up |
| SLC27A2 | 31.00451 | 1.495133 | 0.318875 | 4.688774 | 2.75E-06 | 2.59E-05 | up |
| USP18 | 328.656 | 2.112236 | 0.450971 | 4.683748 | 2.82E-06 | 2.65E-05 | up |
| FAM83A | 3.718053 | 2.852874 | 0.609379 | 4.681606 | 2.85E-06 | 2.67E-05 | up |
| ADRA2A | 26.99934 | 1.887994 | 0.403303 | 4.681326 | 2.85E-06 | 2.67E-05 | up |
| SPSB4 | 3.360839 | 3.371755 | 0.72131 | 4.674485 | 2.95E-06 | 2.76E-05 | up |
| BHLHE41 | 33.62295 | 1.269059 | 0.27191 | 4.667207 | 3.05E-06 | 2.84E-05 | up |
| PROM1 | 18.62992 | 2.064437 | 0.442398 | 4.666467 | 3.06E-06 | 2.85E-05 | up |
| GRIN2B | 5.426775 | 2.216325 | 0.475635 | 4.659718 | 3.17E-06 | 2.94E-05 | up |
| ASAP2 | 69.64581 | 1.235451 | 0.265191 | 4.658723 | 3.18E-06 | 2.95E-05 | up |
| IGLV4-3 | 14.93838 | 2.625085 | 0.563605 | 4.657666 | 3.20E-06 | 2.96E-05 | up |
| EPDR1 | 62.99122 | 1.18821 | 0.255114 | 4.657568 | 3.20E-06 | 2.96E-05 | up |
| APOE | 8.901487 | 2.230505 | 0.479065 | 4.655958 | 3.22E-06 | 2.98E-05 | up |
| IGLL5 | 204.2619 | 1.497115 | 0.321542 | 4.656052 | 3.22E-06 | 2.98E-05 | up |
| SEC14L5 | 64.19227 | 2.005312 | 0.431011 | 4.652572 | 3.28E-06 | 3.02E-05 | up |
| IL23A | 141.389 | -1.37666 | 0.295917 | -4.65218 | 3.28E-06 | 3.02E-05 | down |
| RYR2 | 24.20026 | 1.788437 | 0.384448 | 4.65196 | 3.29E-06 | 3.03E-05 | up |
| VWF | 133.0213 | 1.674908 | 0.360349 | 4.64801 | 3.35E-06 | 3.08E-05 | up |
| FST | 11.5891 | 3.231651 | 0.695441 | 4.646911 | 3.37E-06 | 3.09E-05 | up |
| FRMD3 | 191.6253 | 1.245307 | 0.268158 | 4.643927 | 3.42E-06 | 3.13E-05 | up |
| SAP30 | 461.9925 | 1.337133 | 0.287973 | 4.643253 | 3.43E-06 | 3.14E-05 | up |
| SLFN14 | 44.29702 | 1.417212 | 0.305249 | 4.642799 | 3.44E-06 | 3.14E-05 | up |
| LOC150051 | 3.668654 | 2.272367 | 0.489674 | 4.640571 | 3.47E-06 | 3.17E-05 | up |
| MGLL | 504.4377 | 1.274511 | 0.274718 | 4.639346 | 3.50E-06 | 3.18E-05 | up |
| HCAR2 | 492.1405 | -1.50794 | 0.325041 | -4.63924 | 3.50E-06 | 3.18E-05 | down |
| BCL2L1 | 720.1669 | 1.255969 | 0.270992 | 4.634713 | 3.57E-06 | 3.25E-05 | up |
| TPST1 | 248.0382 | 1.983407 | 0.428004 | 4.634086 | 3.59E-06 | 3.25E-05 | up |
| BEND7 | 7.404306 | 1.945453 | 0.419904 | 4.633086 | 3.60E-06 | 3.27E-05 | up |
| ITGA9 | 29.32405 | 1.716011 | 0.370551 | 4.630973 | 3.64E-06 | 3.30E-05 | up |
| FGD5 | 5.407347 | 1.995879 | 0.431256 | 4.628063 | 3.69E-06 | 3.33E-05 | up |
| DCAF12 | 1419.684 | 1.566657 | 0.338668 | 4.625934 | 3.73E-06 | 3.36E-05 | up |
| RUNDC3A | 10.71258 | 3.424861 | 0.741174 | 4.620857 | 3.82E-06 | 3.43E-05 | up |
| IFI6 | 2763.759 | 1.988916 | 0.430746 | 4.617374 | 3.89E-06 | 3.49E-05 | up |
| GNAZ | 202.7568 | 1.360551 | 0.294856 | 4.614286 | 3.94E-06 | 3.53E-05 | up |
| IGHV1-17 | 4.931871 | 2.293246 | 0.497068 | 4.613541 | 3.96E-06 | 3.54E-05 | up |
| IGKV6D-21 | 28.64634 | 3.053645 | 0.662269 | 4.610884 | 4.01E-06 | 3.58E-05 | up |
| NFE2L2 | 1998.745 | -1.05534 | 0.228949 | -4.60949 | 4.04E-06 | 3.60E-05 | down |
| MTCO1P12 | 209.3377 | 2.618785 | 0.569076 | 4.601823 | 4.19E-06 | 3.72E-05 | up |
| SLC6A4 | 43.45375 | 1.4491 | 0.31497 | 4.600756 | 4.21E-06 | 3.74E-05 | up |
| ACCSL | 5.761244 | 2.972362 | 0.647183 | 4.592773 | 4.37E-06 | 3.87E-05 | up |
| IGHV3-62 | 4.174091 | 2.764623 | 0.602251 | 4.590485 | 4.42E-06 | 3.90E-05 | up |
| ASPH | 508.7709 | 1.020571 | 0.222357 | 4.589784 | 4.44E-06 | 3.91E-05 | up |
| PRSS27 | 10.88691 | 1.25254 | 0.273149 | 4.585558 | 4.53E-06 | 3.98E-05 | up |
| ASXL3 | 4.038718 | 2.426819 | 0.529562 | 4.582687 | 4.59E-06 | 4.03E-05 | up |
| KIF3C | 105.0651 | 1.057182 | 0.23076 | 4.581304 | 4.62E-06 | 4.05E-05 | up |
| PDE10A | 4.335433 | 2.695823 | 0.590146 | 4.568057 | 4.92E-06 | 4.29E-05 | up |
| LINC00473 | 4.335433 | 2.695823 | 0.590146 | 4.568057 | 4.92E-06 | 4.29E-05 | up |
| XRCC2 | 20.73151 | 1.292736 | 0.282986 | 4.568193 | 4.92E-06 | 4.29E-05 | up |
| MARCHF2 | 1534.061 | 1.032994 | 0.226223 | 4.566258 | 4.97E-06 | 4.32E-05 | up |
| CACNA1E | 5.948997 | 2.024106 | 0.443641 | 4.562485 | 5.06E-06 | 4.40E-05 | up |
| ALDH1L1 | 3.304095 | 2.341727 | 0.513428 | 4.560966 | 5.09E-06 | 4.43E-05 | up |
| CFAP161 | 21.09533 | 1.682474 | 0.3695 | 4.553378 | 5.28E-06 | 4.58E-05 | up |
| DLL3 | 3.852773 | 1.519916 | 0.333872 | 4.552391 | 5.30E-06 | 4.59E-05 | up |
| SEPTIN4 | 72.60921 | 1.342082 | 0.294859 | 4.551603 | 5.32E-06 | 4.60E-05 | up |
| IGHV3-72 | 163.299 | 2.339559 | 0.514244 | 4.549508 | 5.38E-06 | 4.64E-05 | up |
| EPHB2 | 169.088 | 1.409933 | 0.310153 | 4.545932 | 5.47E-06 | 4.71E-05 | up |
| MED12L | 58.13377 | 1.210177 | 0.266646 | 4.53852 | 5.67E-06 | 4.86E-05 | up |
| SLC16A10 | 77.27667 | -1.00497 | 0.22156 | -4.53586 | 5.74E-06 | 4.92E-05 | down |
| HMCN2 | 5.764132 | 2.221973 | 0.49001 | 4.534547 | 5.77E-06 | 4.94E-05 | up |
| PPP1R14A | 56.34192 | 1.207748 | 0.26636 | 4.534264 | 5.78E-06 | 4.95E-05 | up |
| YOD1 | 237.6728 | 1.041752 | 0.230081 | 4.527758 | 5.96E-06 | 5.08E-05 | up |
| TINCR | 5.680814 | 1.410228 | 0.312224 | 4.516723 | 6.28E-06 | 5.32E-05 | up |
| DYSF | 2098.444 | 1.443608 | 0.319828 | 4.513696 | 6.37E-06 | 5.39E-05 | up |
| TACC2 | 3.974583 | 2.435737 | 0.539974 | 4.510839 | 6.46E-06 | 5.45E-05 | up |
| PRTN3 | 4.246745 | 3.931514 | 0.872448 | 4.506301 | 6.60E-06 | 5.56E-05 | up |
| TGM2 | 137.3165 | 1.171226 | 0.260136 | 4.502362 | 6.72E-06 | 5.64E-05 | up |
| VSIG4 | 487.1659 | 1.869699 | 0.415451 | 4.500403 | 6.78E-06 | 5.69E-05 | up |
| PDE5A | 207.2165 | 1.163766 | 0.258601 | 4.500245 | 6.79E-06 | 5.69E-05 | up |
| VWCE | 6.530354 | 2.084023 | 0.463354 | 4.497694 | 6.87E-06 | 5.75E-05 | up |
| HMGB1P5 | 123.6999 | 1.33448 | 0.296799 | 4.496239 | 6.92E-06 | 5.78E-05 | up |
| TMEM198 | 5.639187 | 1.305737 | 0.290397 | 4.496388 | 6.91E-06 | 5.78E-05 | up |
| GPC1 | 30.6431 | 1.137976 | 0.253132 | 4.495579 | 6.94E-06 | 5.79E-05 | up |
| NPW | 8.736676 | 1.01323 | 0.225664 | 4.489994 | 7.12E-06 | 5.94E-05 | up |
| CALHM5 | 15.56694 | 1.913526 | 0.426415 | 4.487469 | 7.21E-06 | 6.00E-05 | up |
| CACNA1A | 33.9258 | 1.044317 | 0.232764 | 4.486592 | 7.24E-06 | 6.02E-05 | up |
| ADCY1 | 3.850124 | 2.066475 | 0.461108 | 4.481542 | 7.41E-06 | 6.15E-05 | up |
| FCGR3B | 83.7541 | 2.091282 | 0.466899 | 4.479085 | 7.50E-06 | 6.21E-05 | up |
| SHANK2 | 4.330421 | 2.049326 | 0.457631 | 4.478121 | 7.53E-06 | 6.22E-05 | up |
| IL18RAP | 575.0994 | -1.1139 | 0.248953 | -4.47434 | 7.66E-06 | 6.33E-05 | down |
| ABCC8 | 4.079192 | 2.046558 | 0.457902 | 4.469424 | 7.84E-06 | 6.47E-05 | up |
| LOC112267895 | 3.785003 | 3.726158 | 0.833938 | 4.468147 | 7.89E-06 | 6.50E-05 | up |
| IGKV2-26 | 15.59552 | 2.199039 | 0.49372 | 4.454022 | 8.43E-06 | 6.90E-05 | up |
| IGLV2-28 | 3.342762 | 2.236277 | 0.5027 | 4.448528 | 8.65E-06 | 7.07E-05 | up |
| FGF9 | 19.96291 | -1.02524 | 0.230587 | -4.44622 | 8.74E-06 | 7.13E-05 | down |
| LOC101926892 | 4.155217 | 3.88559 | 0.87508 | 4.44027 | 8.98E-06 | 7.31E-05 | up |
| APOA1 | 5.177098 | 1.320386 | 0.297702 | 4.435268 | 9.20E-06 | 7.46E-05 | up |
| TCEAL9 | 20.27159 | 1.215605 | 0.274123 | 4.434525 | 9.23E-06 | 7.47E-05 | up |
| S100A2 | 3.818534 | 1.643715 | 0.370688 | 4.434231 | 9.24E-06 | 7.48E-05 | up |
| LINC01801 | 21.60229 | -1.21573 | 0.274177 | -4.43412 | 9.24E-06 | 7.48E-05 | down |
| SLC26A8 | 21.92728 | 1.723603 | 0.388948 | 4.431448 | 9.36E-06 | 7.57E-05 | up |
| VSIG10L | 32.71824 | 1.071065 | 0.241926 | 4.427243 | 9.54E-06 | 7.70E-05 | up |
| ITGA2 | 16.39229 | 1.383854 | 0.312733 | 4.425031 | 9.64E-06 | 7.77E-05 | up |
| HMCN1 | 7.960066 | 2.007854 | 0.453962 | 4.422958 | 9.74E-06 | 7.83E-05 | up |
| FA2H | 16.49862 | 1.490643 | 0.337049 | 4.422635 | 9.75E-06 | 7.83E-05 | up |
| TRPC1 | 29.01505 | -1.0433 | 0.236143 | -4.41809 | 9.96E-06 | 7.97E-05 | down |
| LINC00853 | 10.63463 | 1.744584 | 0.395033 | 4.416296 | 1.00E-05 | 8.02E-05 | up |
| CDR2L | 7.688928 | 2.113303 | 0.478865 | 4.413148 | 1.02E-05 | 8.13E-05 | up |
| GAS2L1 | 857.1265 | 1.210358 | 0.274275 | 4.412943 | 1.02E-05 | 8.13E-05 | up |
| LINC01606 | 3.948741 | 3.31846 | 0.752621 | 4.409205 | 1.04E-05 | 8.25E-05 | up |
| MAGI2 | 12.14675 | 1.416615 | 0.321409 | 4.407514 | 1.05E-05 | 8.31E-05 | up |
| MUC3A | 6.254227 | 2.437627 | 0.55361 | 4.403153 | 1.07E-05 | 8.46E-05 | up |
| GAL3ST4 | 59.29813 | -1.13741 | 0.25849 | -4.40021 | 1.08E-05 | 8.57E-05 | down |
| IGHD6-25 | 3.884422 | 1.972113 | 0.448339 | 4.398713 | 1.09E-05 | 8.62E-05 | up |
| MUC4 | 4.375344 | 1.827198 | 0.415447 | 4.398147 | 1.09E-05 | 8.63E-05 | up |
| IGHV3-22 | 4.209384 | 2.254367 | 0.512881 | 4.395493 | 1.11E-05 | 8.71E-05 | up |
| BTN1A1 | 10.29419 | 1.799606 | 0.409866 | 4.390719 | 1.13E-05 | 8.87E-05 | up |
| DLGAP2 | 4.025616 | 2.247527 | 0.512038 | 4.38938 | 1.14E-05 | 8.91E-05 | up |
| LINC02861 | 3.262041 | 1.538427 | 0.350516 | 4.38903 | 1.14E-05 | 8.92E-05 | up |
| IGHD3-3 | 10.24049 | 1.540102 | 0.350973 | 4.388093 | 1.14E-05 | 8.94E-05 | up |
| KRT8 | 5.283233 | 1.214363 | 0.276795 | 4.387223 | 1.15E-05 | 8.97E-05 | up |
| MYL4 | 19.35876 | 3.106879 | 0.708377 | 4.385912 | 1.16E-05 | 9.02E-05 | up |
| IFI44L | 1416.555 | 2.405476 | 0.548975 | 4.381757 | 1.18E-05 | 9.16E-05 | up |
| LILRB4 | 1108.603 | 1.030047 | 0.235073 | 4.38181 | 1.18E-05 | 9.16E-05 | up |
| ARL5B | 842.4753 | -2.00572 | 0.457851 | -4.38072 | 1.18E-05 | 9.20E-05 | down |
| APOBEC3H | 65.88586 | 1.087701 | 0.248435 | 4.378216 | 1.20E-05 | 9.28E-05 | up |
| NMNAT2 | 3.637788 | 2.086181 | 0.476613 | 4.377096 | 1.20E-05 | 9.32E-05 | up |
| NOG | 46.30692 | -1.77652 | 0.405962 | -4.37608 | 1.21E-05 | 9.35E-05 | down |
| MEIS3 | 7.261557 | 2.045749 | 0.467507 | 4.375867 | 1.21E-05 | 9.35E-05 | up |
| IGHV3-71 | 4.284205 | 2.288615 | 0.523395 | 4.372634 | 1.23E-05 | 9.48E-05 | up |
| LRRN3 | 287.5758 | -1.53001 | 0.349917 | -4.37248 | 1.23E-05 | 9.48E-05 | down |
| IGLV10-54 | 167.4332 | 2.475474 | 0.566435 | 4.37027 | 1.24E-05 | 9.55E-05 | up |
| CSMD2 | 5.828903 | 2.35109 | 0.538836 | 4.363279 | 1.28E-05 | 9.81E-05 | up |
| MS4A3 | 3.433445 | 2.211266 | 0.507076 | 4.36082 | 1.30E-05 | 9.91E-05 | up |
| H4C14 | 8.90412 | 1.986853 | 0.455692 | 4.360078 | 1.30E-05 | 9.93E-05 | up |
| ZNF462 | 3.941076 | 1.586975 | 0.364053 | 4.359182 | 1.31E-05 | 9.97E-05 | up |
| DYTN | 9.469135 | 1.794455 | 0.411785 | 4.357747 | 1.31E-05 | 0.0001 | up |
| RNF152 | 7.528551 | 1.832129 | 0.420627 | 4.355707 | 1.33E-05 | 0.000101 | up |
| LINC01748 | 3.776202 | 2.350096 | 0.540384 | 4.348939 | 1.37E-05 | 0.000104 | up |
| GUCY1B1 | 242.6917 | 1.244654 | 0.286242 | 4.34825 | 1.37E-05 | 0.000104 | up |
| SLCO2A1 | 4.240658 | 2.402757 | 0.553017 | 4.344816 | 1.39E-05 | 0.000105 | up |
| KLHDC8A | 8.06969 | 3.144938 | 0.723987 | 4.343916 | 1.40E-05 | 0.000106 | up |
| DNAH3 | 8.11941 | 1.504274 | 0.34638 | 4.34285 | 1.41E-05 | 0.000106 | up |
| BIRC7 | 7.037719 | -2.98856 | 0.688154 | -4.34286 | 1.41E-05 | 0.000106 | down |
| SUCNR1 | 29.95701 | 1.708566 | 0.393577 | 4.341126 | 1.42E-05 | 0.000107 | up |
| WDFY3-AS2 | 43.66493 | 1.007839 | 0.232274 | 4.339018 | 1.43E-05 | 0.000108 | up |
| CPS1 | 5.222608 | 1.715269 | 0.395417 | 4.337878 | 1.44E-05 | 0.000108 | up |
| CAMP | 12.44965 | 1.2478 | 0.287717 | 4.336896 | 1.45E-05 | 0.000109 | up |
| GJA4 | 5.887133 | 2.942121 | 0.678738 | 4.334693 | 1.46E-05 | 0.00011 | up |
| ITGB8-AS1 | 6.100869 | 1.586654 | 0.366119 | 4.333707 | 1.47E-05 | 0.00011 | up |
| GPR27 | 209.5289 | 1.265566 | 0.292361 | 4.328776 | 1.50E-05 | 0.000112 | up |
| TOM1L1 | 4.571273 | 2.00115 | 0.462334 | 4.328368 | 1.50E-05 | 0.000112 | up |
| HTRA1 | 60.16615 | 1.802655 | 0.416517 | 4.327928 | 1.51E-05 | 0.000112 | up |
| VIL1 | 74.94158 | 1.565682 | 0.362092 | 4.32399 | 1.53E-05 | 0.000114 | up |
| CNN1 | 12.6304 | 2.841148 | 0.65721 | 4.323046 | 1.54E-05 | 0.000115 | up |
| EDAR | 138.7044 | -1.22031 | 0.282289 | -4.3229 | 1.54E-05 | 0.000115 | down |
| PBX1 | 49.32185 | 1.286938 | 0.297874 | 4.320413 | 1.56E-05 | 0.000116 | up |
| MAP3K7CL | 690.3081 | 1.25167 | 0.289928 | 4.317183 | 1.58E-05 | 0.000117 | up |
| LOC112694756 | 6.337591 | 1.539974 | 0.356974 | 4.313972 | 1.60E-05 | 0.000119 | up |
| ADGRE3 | 189.7926 | -1.37844 | 0.319607 | -4.31293 | 1.61E-05 | 0.000119 | down |
| GFAP | 5.5452 | 1.471062 | 0.341185 | 4.311632 | 1.62E-05 | 0.00012 | up |
| CRACD | 5.019684 | 1.943918 | 0.451502 | 4.305444 | 1.67E-05 | 0.000123 | up |
| SLC1A2 | 14.55882 | 1.212496 | 0.281712 | 4.304029 | 1.68E-05 | 0.000124 | up |
| MPZL3 | 410.4532 | -1.07256 | 0.249236 | -4.30338 | 1.68E-05 | 0.000124 | down |
| HCAR3 | 339.9177 | -1.17963 | 0.274315 | -4.30029 | 1.71E-05 | 0.000126 | down |
| KCTD14 | 5.343708 | 2.50853 | 0.583966 | 4.295677 | 1.74E-05 | 0.000128 | up |
| TSC22D1 | 1321.866 | 1.053908 | 0.245358 | 4.295382 | 1.74E-05 | 0.000128 | up |
| FAM230C | 14.61393 | 2.393444 | 0.557271 | 4.294939 | 1.75E-05 | 0.000128 | up |
| MIR573 | 3.742775 | 1.620575 | 0.37739 | 4.294165 | 1.75E-05 | 0.000129 | up |
| JAM3 | 195.0796 | 1.369994 | 0.319262 | 4.291125 | 1.78E-05 | 0.00013 | up |
| SLED1 | 22.64953 | 1.614798 | 0.376509 | 4.288865 | 1.80E-05 | 0.000131 | up |
| CEL | 5.589418 | 2.324451 | 0.542317 | 4.286147 | 1.82E-05 | 0.000133 | up |
| PWRN1 | 6.648187 | 2.703969 | 0.631227 | 4.283673 | 1.84E-05 | 0.000134 | up |
| PWRN3 | 6.648187 | 2.703969 | 0.631227 | 4.283673 | 1.84E-05 | 0.000134 | up |
| CFAP251 | 7.316305 | 1.545825 | 0.360897 | 4.283288 | 1.84E-05 | 0.000134 | up |
| TRMT9B | 6.68192 | 1.974727 | 0.461442 | 4.279469 | 1.87E-05 | 0.000136 | up |
| UNC80 | 4.637474 | 2.166606 | 0.506684 | 4.276048 | 1.90E-05 | 0.000138 | up |
| MAP1A | 328.9244 | 1.36816 | 0.320138 | 4.273661 | 1.92E-05 | 0.000139 | up |
| ALDH1A1 | 447.4232 | -1.24098 | 0.290667 | -4.26943 | 1.96E-05 | 0.000142 | down |
| FZD5 | 43.68884 | 1.157923 | 0.271515 | 4.264669 | 2.00E-05 | 0.000144 | up |
| SLC19A2 | 83.5628 | -1.21898 | 0.286061 | -4.26126 | 2.03E-05 | 0.000146 | down |
| BBS10 | 53.67297 | -1.04326 | 0.245042 | -4.25748 | 2.07E-05 | 0.000148 | down |
| GPT2 | 129.2299 | 1.056555 | 0.248228 | 4.25639 | 2.08E-05 | 0.000149 | up |
| NTSR1 | 37.43212 | 2.015412 | 0.473717 | 4.254468 | 2.10E-05 | 0.00015 | up |
| H2AC4 | 3.562151 | 1.39605 | 0.328294 | 4.252431 | 2.11E-05 | 0.000151 | up |
| COL27A1 | 5.957623 | 1.451137 | 0.341298 | 4.251823 | 2.12E-05 | 0.000151 | up |
| HTR1F | 5.448517 | 2.144014 | 0.504305 | 4.25142 | 2.12E-05 | 0.000152 | up |
| CCDC80 | 13.1774 | 1.168886 | 0.274951 | 4.251253 | 2.13E-05 | 0.000152 | up |
| CD209 | 25.65463 | -1.21017 | 0.284699 | -4.2507 | 2.13E-05 | 0.000152 | down |
| HKDC1 | 56.30938 | -1.14274 | 0.26917 | -4.2454 | 2.18E-05 | 0.000155 | down |
| FBN1 | 13.43156 | 1.445414 | 0.340554 | 4.244306 | 2.19E-05 | 0.000156 | up |
| DAAM2 | 2.319896 | 2.504524 | 0.590764 | 4.239467 | 2.24E-05 | 0.000159 | up |
| NEXN | 111.8675 | 1.237345 | 0.291947 | 4.238259 | 2.25E-05 | 0.00016 | up |
| H4C4 | 3.623593 | 1.796982 | 0.424434 | 4.23383 | 2.30E-05 | 0.000162 | up |
| CPT1C | 9.118628 | 1.636191 | 0.386958 | 4.228339 | 2.35E-05 | 0.000166 | up |
| SPRY2 | 115.0031 | 1.609834 | 0.38093 | 4.226064 | 2.38E-05 | 0.000167 | up |
| TMC1 | 3.910064 | 1.784976 | 0.422842 | 4.221377 | 2.43E-05 | 0.00017 | up |
| CCR3 | 17.41985 | 1.115294 | 0.264321 | 4.21947 | 2.45E-05 | 0.000172 | up |
| NPR3 | 15.08082 | 1.874469 | 0.444834 | 4.213862 | 2.51E-05 | 0.000176 | up |
| FILIP1L | 38.14402 | -1.59333 | 0.378453 | -4.2101 | 2.55E-05 | 0.000178 | down |
| HERC2P2 | 330.6026 | -1.09852 | 0.261039 | -4.20826 | 2.57E-05 | 0.00018 | down |
| IGLV3-6 | 3.421943 | 2.492677 | 0.592456 | 4.207363 | 2.58E-05 | 0.00018 | up |
| NTRK2 | 5.93577 | 1.552218 | 0.36898 | 4.20678 | 2.59E-05 | 0.000181 | up |
| TMPRSS9 | 5.653106 | 1.909496 | 0.454274 | 4.203403 | 2.63E-05 | 0.000183 | up |
| CCDC137P | 5.667155 | -1.48186 | 0.352571 | -4.20301 | 2.63E-05 | 0.000183 | down |
| CCRL2 | 311.0854 | -1.65012 | 0.392596 | -4.20309 | 2.63E-05 | 0.000183 | down |
| DNAH9 | 6.536376 | 2.357766 | 0.561155 | 4.201631 | 2.65E-05 | 0.000184 | up |
| AICDA | 6.057899 | 1.564602 | 0.37251 | 4.200166 | 2.67E-05 | 0.000185 | up |
| LINC00293 | 4.692183 | 2.551935 | 0.608106 | 4.196533 | 2.71E-05 | 0.000188 | up |
| TRDV2 | 106.8642 | -2.47696 | 0.591785 | -4.18556 | 2.84E-05 | 0.000196 | down |
| LINC01359 | 23.8917 | 1.016592 | 0.243017 | 4.183211 | 2.87E-05 | 0.000197 | up |
| MYH15 | 4.677139 | 1.629624 | 0.389668 | 4.18208 | 2.89E-05 | 0.000198 | up |
| FMN1 | 129.5338 | 1.374744 | 0.328992 | 4.178661 | 2.93E-05 | 0.000201 | up |
| SERPING1 | 601.2983 | 1.721546 | 0.412317 | 4.175297 | 2.98E-05 | 0.000204 | up |
| TMDD1 | 3.843862 | 1.348172 | 0.323114 | 4.172431 | 3.01E-05 | 0.000206 | up |
| PDGFA | 24.76407 | 1.750698 | 0.419768 | 4.170637 | 3.04E-05 | 0.000207 | up |
| IGKV1D-17 | 38.06657 | 3.95972 | 0.950388 | 4.166426 | 3.09E-05 | 0.00021 | up |
| EMID1 | 13.07778 | 1.553115 | 0.37286 | 4.165405 | 3.11E-05 | 0.000211 | up |
| EMP1 | 290.3147 | 1.667988 | 0.40081 | 4.161547 | 3.16E-05 | 0.000214 | up |
| RGL3 | 8.226477 | 1.700075 | 0.408581 | 4.160925 | 3.17E-05 | 0.000215 | up |
| VSIG2 | 86.08389 | 1.158521 | 0.278417 | 4.161095 | 3.17E-05 | 0.000215 | up |
| GPR3 | 12.25555 | 1.125666 | 0.270777 | 4.157169 | 3.22E-05 | 0.000217 | up |
| COBL | 5.077347 | 1.33448 | 0.321759 | 4.147448 | 3.36E-05 | 0.000226 | up |
| UNC79 | 9.781912 | 1.052565 | 0.254276 | 4.139451 | 3.48E-05 | 0.000233 | up |
| FREM2 | 3.237934 | 2.724605 | 0.658494 | 4.13763 | 3.51E-05 | 0.000234 | up |
| ZNF600 | 672.9365 | -1.02245 | 0.247131 | -4.13729 | 3.51E-05 | 0.000234 | down |
| SDC2 | 30.57373 | 1.612349 | 0.390638 | 4.127475 | 3.67E-05 | 0.000243 | up |
| ACSBG1 | 10.31966 | 1.357889 | 0.3293 | 4.123555 | 3.73E-05 | 0.000247 | up |
| ITGB8 | 12.46127 | 1.18495 | 0.287472 | 4.121963 | 3.76E-05 | 0.000248 | up |
| ENPP6 | 3.32456 | 2.103962 | 0.510491 | 4.121451 | 3.76E-05 | 0.000248 | up |
| FAM20C | 122.0885 | 1.123741 | 0.272949 | 4.117039 | 3.84E-05 | 0.000252 | up |
| RGMA | 16.11195 | 1.487729 | 0.361503 | 4.115399 | 3.87E-05 | 0.000254 | up |
| DNAH2 | 12.04003 | 1.919146 | 0.466975 | 4.109742 | 3.96E-05 | 0.00026 | up |
| MGST1 | 436.1866 | 1.152398 | 0.280466 | 4.108872 | 3.98E-05 | 0.000261 | up |
| COL4A1 | 5.14358 | 1.598441 | 0.389077 | 4.10829 | 3.99E-05 | 0.000261 | up |
| IDH1-AS1 | 3.816988 | 1.370532 | 0.333593 | 4.108399 | 3.98E-05 | 0.000261 | up |
| BEST3 | 4.950313 | 2.314268 | 0.563341 | 4.10811 | 3.99E-05 | 0.000261 | up |
| SELENOP | 4.526088 | 1.581202 | 0.384974 | 4.107299 | 4.00E-05 | 0.000262 | up |
| MMP1 | 4.062707 | 2.940024 | 0.716599 | 4.102745 | 4.08E-05 | 0.000266 | up |
| BEND2 | 57.21249 | 1.446273 | 0.352792 | 4.099505 | 4.14E-05 | 0.000269 | up |
| SLC7A11 | 13.33219 | 1.39512 | 0.340528 | 4.096932 | 4.19E-05 | 0.000272 | up |
| TREML3P | 24.67509 | 1.364847 | 0.333281 | 4.095188 | 4.22E-05 | 0.000274 | up |
| PRSS35 | 4.323346 | -2.60365 | 0.63606 | -4.0934 | 4.25E-05 | 0.000276 | down |
| TRAJ32 | 14.3109 | -1.0617 | 0.259456 | -4.09202 | 4.28E-05 | 0.000277 | down |
| FDPSP3 | 7.153233 | -1.38499 | 0.338486 | -4.09174 | 4.28E-05 | 0.000277 | down |
| FAM124A | 8.291171 | 1.78526 | 0.436794 | 4.087188 | 4.37E-05 | 0.000283 | up |
| MUC19 | 8.910305 | 2.204893 | 0.539724 | 4.085223 | 4.40E-05 | 0.000285 | up |
| ABCA4 | 4.049273 | 2.21619 | 0.542631 | 4.08416 | 4.42E-05 | 0.000286 | up |
| TGIF1 | 807.6337 | -1.07091 | 0.262472 | -4.0801 | 4.50E-05 | 0.00029 | down |
| ELAVL4 | 3.435274 | 1.707058 | 0.418832 | 4.075757 | 4.59E-05 | 0.000295 | up |
| FAM81B | 6.041105 | 1.426684 | 0.350037 | 4.075811 | 4.59E-05 | 0.000295 | up |
| HECW1 | 3.234833 | 1.878224 | 0.460887 | 4.075235 | 4.60E-05 | 0.000295 | up |
| F3 | 21.75101 | 1.890703 | 0.464235 | 4.072726 | 4.65E-05 | 0.000298 | up |
| CNTF | 5.656126 | 1.063254 | 0.261285 | 4.069329 | 4.71E-05 | 0.000302 | up |
| TMEM256P2 | 13.33413 | -1.22353 | 0.300799 | -4.0676 | 4.75E-05 | 0.000304 | down |
| LOC285626 | 8.853044 | 1.300903 | 0.319902 | 4.066571 | 4.77E-05 | 0.000305 | up |
| SMIM10L2A | 11.59871 | -1.04097 | 0.256045 | -4.06559 | 4.79E-05 | 0.000306 | down |
| PRTFDC1 | 18.95519 | 1.406697 | 0.34629 | 4.062199 | 4.86E-05 | 0.00031 | up |
| FECH | 770.1757 | 1.066465 | 0.262572 | 4.061603 | 4.87E-05 | 0.00031 | up |
| DPYSL3 | 4.060999 | 1.713885 | 0.422231 | 4.059116 | 4.93E-05 | 0.000313 | up |
| TRAPPC3L | 12.17812 | 1.532575 | 0.377731 | 4.057315 | 4.96E-05 | 0.000315 | up |
| ACER2 | 35.34101 | 1.655683 | 0.408195 | 4.056108 | 4.99E-05 | 0.000317 | up |
| LINC02610 | 32.51276 | -1.08643 | 0.267882 | -4.05561 | 5.00E-05 | 0.000317 | down |
| CCL7 | 8.166711 | 3.176848 | 0.78445 | 4.049776 | 5.13E-05 | 0.000324 | up |
| SLIT2 | 3.520441 | 1.7798 | 0.439565 | 4.049003 | 5.14E-05 | 0.000325 | up |
| CAVIN3 | 49.61969 | 1.88473 | 0.46564 | 4.047611 | 5.17E-05 | 0.000327 | up |
| EMILIN1 | 72.55037 | 1.083303 | 0.267958 | 4.042816 | 5.28E-05 | 0.000333 | up |
| TRAJ38 | 5.493684 | -1.11231 | 0.275174 | -4.04221 | 5.29E-05 | 0.000333 | down |
| IGFBP2 | 63.58066 | 1.938908 | 0.479975 | 4.039601 | 5.35E-05 | 0.000337 | up |
| CD34 | 34.37179 | 1.753999 | 0.43419 | 4.039707 | 5.35E-05 | 0.000337 | up |
| BTG2 | 9592.76 | -1.04431 | 0.258522 | -4.03955 | 5.36E-05 | 0.000337 | down |
| LINC02055 | 6.420983 | 1.869066 | 0.463264 | 4.034557 | 5.47E-05 | 0.000343 | up |
| KLF14 | 5.050112 | 2.063349 | 0.512359 | 4.027156 | 5.65E-05 | 0.000352 | up |
| ERCC6L | 21.12985 | 1.055501 | 0.262528 | 4.020522 | 5.81E-05 | 0.000361 | up |
| RDM1 | 5.694973 | 1.247811 | 0.310464 | 4.019174 | 5.84E-05 | 0.000363 | up |
| C1orf198 | 338.982 | 1.02616 | 0.255811 | 4.011393 | 6.04E-05 | 0.000373 | up |
| NUPR1 | 3.501725 | 1.988533 | 0.49657 | 4.004535 | 6.21E-05 | 0.000383 | up |
| ST8SIA5 | 4.112311 | 1.950186 | 0.48725 | 4.002433 | 6.27E-05 | 0.000386 | up |
| KRT7 | 9.489657 | 1.623358 | 0.405683 | 4.001541 | 6.29E-05 | 0.000387 | up |
| SLC47A1 | 21.86144 | -1.28087 | 0.320155 | -4.00078 | 6.31E-05 | 0.000388 | down |
| ACVRL1 | 25.94532 | 1.34414 | 0.336004 | 4.000364 | 6.32E-05 | 0.000388 | up |
| IGFALS | 5.307866 | 1.420515 | 0.355142 | 3.999847 | 6.34E-05 | 0.000389 | up |
| PLBD1 | 6264.424 | 1.257738 | 0.314702 | 3.996601 | 6.43E-05 | 0.000394 | up |
| USH2A | 4.483327 | 2.427059 | 0.60877 | 3.986822 | 6.70E-05 | 0.000409 | up |
| H4C2 | 8.240494 | 1.501828 | 0.376867 | 3.985035 | 6.75E-05 | 0.000411 | up |
| ID1 | 409.32 | 1.130328 | 0.283656 | 3.984851 | 6.75E-05 | 0.000411 | up |
| LURAP1L | 3.733753 | 1.779042 | 0.446592 | 3.983596 | 6.79E-05 | 0.000413 | up |
| PKN2-AS1 | 6.983054 | 1.200314 | 0.30151 | 3.981004 | 6.86E-05 | 0.000416 | up |
| SEPTIN3 | 6.282738 | 1.351847 | 0.33988 | 3.977428 | 6.97E-05 | 0.000422 | up |
| PARS2 | 50.42713 | -1.00341 | 0.25242 | -3.97515 | 7.03E-05 | 0.000425 | down |
| TRAJ41 | 5.071034 | -1.33957 | 0.337795 | -3.96562 | 7.32E-05 | 0.000441 | down |
| RAB27B | 177.9044 | 1.098063 | 0.277231 | 3.960828 | 7.47E-05 | 0.000448 | up |
| SNRPA1-DT | 6.053555 | 1.526068 | 0.385387 | 3.959834 | 7.50E-05 | 0.00045 | up |
| LIPG | 3.610149 | 2.004281 | 0.506821 | 3.954612 | 7.67E-05 | 0.000457 | up |
| CNBD2 | 4.293348 | 1.259944 | 0.319146 | 3.947863 | 7.89E-05 | 0.00047 | up |
| TYRO3 | 8.593709 | 1.270128 | 0.321737 | 3.947717 | 7.89E-05 | 0.00047 | up |
| SPP1 | 7.07729 | 3.095829 | 0.784586 | 3.945811 | 7.95E-05 | 0.000473 | up |
| FCGR1CP | 26.63455 | 1.505104 | 0.381513 | 3.945097 | 7.98E-05 | 0.000474 | up |
| IGHJ4 | 8.849321 | 1.406156 | 0.356568 | 3.943587 | 8.03E-05 | 0.000477 | up |
| DPF1 | 5.709504 | 1.332195 | 0.337853 | 3.943116 | 8.04E-05 | 0.000477 | up |
| UICLM | 84.83253 | -1.79969 | 0.45652 | -3.94219 | 8.07E-05 | 0.000479 | down |
| ARHGAP42 | 31.50529 | 1.084173 | 0.275044 | 3.941809 | 8.09E-05 | 0.000479 | up |
| FAT3 | 4.209086 | 1.917102 | 0.486698 | 3.938993 | 8.18E-05 | 0.000484 | up |
| FASLG | 171.3864 | -1.09507 | 0.278044 | -3.9385 | 8.20E-05 | 0.000484 | down |
| SPAG6 | 8.805475 | 1.424334 | 0.361956 | 3.935105 | 8.32E-05 | 0.00049 | up |
| RAB3B | 3.73707 | 2.49787 | 0.635856 | 3.928359 | 8.55E-05 | 0.000502 | up |
| ITIH5 | 3.201326 | 2.369146 | 0.60331 | 3.926913 | 8.60E-05 | 0.000505 | up |
| ITIH3 | 4.403014 | 1.633342 | 0.416161 | 3.924784 | 8.68E-05 | 0.000509 | up |
| FAT1 | 3.512023 | 2.345752 | 0.598306 | 3.920655 | 8.83E-05 | 0.000516 | up |
| NAB2 | 170.1383 | 1.283808 | 0.327475 | 3.920329 | 8.84E-05 | 0.000516 | up |
| IL1B | 3131.907 | -1.72445 | 0.439875 | -3.92031 | 8.84E-05 | 0.000516 | down |
| IL1R2 | 157.0061 | 1.848111 | 0.472809 | 3.908791 | 9.28E-05 | 0.000539 | up |
| PILRB | 137.9805 | -1.00191 | 0.25665 | -3.90379 | 9.47E-05 | 0.000547 | down |
| ZNF572 | 10.65345 | -1.03763 | 0.265799 | -3.90381 | 9.47E-05 | 0.000547 | down |
| ASGR2 | 951.766 | 1.090887 | 0.279584 | 3.901816 | 9.55E-05 | 0.000552 | up |
| SRGAP1 | 57.72783 | 1.423825 | 0.365152 | 3.899264 | 9.65E-05 | 0.000557 | up |
| CCL4 | 2207.049 | -1.44022 | 0.369989 | -3.8926 | 9.92E-05 | 0.000571 | down |
| OSBPL6 | 12.06923 | 1.084871 | 0.279465 | 3.881957 | 0.000104 | 0.000594 | up |
| TNFSF4 | 138.0774 | 1.203624 | 0.310398 | 3.877682 | 0.000105 | 0.000602 | up |
| SORD2P | 8.866906 | 1.005109 | 0.259204 | 3.877672 | 0.000105 | 0.000602 | up |
| LBX2 | 5.764683 | 1.031153 | 0.265931 | 3.877521 | 0.000106 | 0.000602 | up |
| EPPK1 | 169.797 | -1.18967 | 0.307 | -3.87515 | 0.000107 | 0.000607 | down |
| ZNF835 | 23.75009 | -1.578 | 0.407233 | -3.87492 | 0.000107 | 0.000607 | down |
| ZNF14 | 288.2207 | -1.24359 | 0.321023 | -3.87384 | 0.000107 | 0.00061 | down |
| FOXB1 | 4.532382 | 1.439811 | 0.371765 | 3.872904 | 0.000108 | 0.000612 | up |
| PROSER2-AS1 | 3.571167 | 1.497509 | 0.387043 | 3.869105 | 0.000109 | 0.00062 | up |
| PTGER4 | 4906.034 | -1.10975 | 0.286869 | -3.8685 | 0.00011 | 0.000621 | down |
| CXCR4 | 9379.823 | -1.15722 | 0.299446 | -3.86455 | 0.000111 | 0.00063 | down |
| FAM151B | 38.74464 | 1.014843 | 0.262654 | 3.863801 | 0.000112 | 0.000632 | up |
| LY6E | 5581.058 | 1.114201 | 0.288399 | 3.863403 | 0.000112 | 0.000632 | up |
| MIR3976HG | 3.232109 | 2.167056 | 0.561475 | 3.859575 | 0.000114 | 0.000642 | up |
| PRKD1 | 6.040035 | 1.659908 | 0.430535 | 3.855455 | 0.000116 | 0.000652 | up |
| GIMAP8 | 1188.592 | -1.01493 | 0.263424 | -3.85283 | 0.000117 | 0.000658 | down |
| H4C6 | 5.764706 | 1.188642 | 0.308781 | 3.849466 | 0.000118 | 0.000666 | up |
| LAMA1 | 4.168775 | 1.959235 | 0.509006 | 3.84914 | 0.000119 | 0.000666 | up |
| PTGES3L | 6.161975 | 1.324653 | 0.344625 | 3.843756 | 0.000121 | 0.00068 | up |
| KY | 3.264869 | 2.198988 | 0.572469 | 3.841236 | 0.000122 | 0.000685 | up |
| LINC01484 | 4.435566 | 2.262796 | 0.589127 | 3.840931 | 0.000123 | 0.000686 | up |
| HORMAD1 | 14.9082 | 1.082985 | 0.282219 | 3.837387 | 0.000124 | 0.000694 | up |
| GCKR | 2.327581 | 3.120143 | 0.813108 | 3.837303 | 0.000124 | 0.000694 | up |
| LOC284930 | 7.280352 | 2.054052 | 0.535568 | 3.83528 | 0.000125 | 0.000699 | up |
| ZNF547 | 16.83146 | -1.26716 | 0.330585 | -3.83308 | 0.000127 | 0.000704 | down |
| NR2F1-AS1 | 4.995424 | 2.210911 | 0.576936 | 3.832159 | 0.000127 | 0.000706 | up |
| OASL | 1226.312 | 1.325992 | 0.346118 | 3.831042 | 0.000128 | 0.000709 | up |
| CCNA1 | 6.379389 | 2.187186 | 0.571026 | 3.830272 | 0.000128 | 0.000711 | up |
| DYNC1I1 | 6.662189 | 1.740046 | 0.45533 | 3.8215 | 0.000133 | 0.000735 | up |
| TMEM98 | 3.66403 | 2.412301 | 0.631695 | 3.818775 | 0.000134 | 0.000742 | up |
| NRXN1 | 4.030846 | 1.893597 | 0.495976 | 3.817918 | 0.000135 | 0.000744 | up |
| CT69 | 6.545613 | 1.359648 | 0.356666 | 3.812104 | 0.000138 | 0.000761 | up |
| LINC00402 | 185.2773 | -1.06145 | 0.278503 | -3.81125 | 0.000138 | 0.000763 | down |
| CDH13 | 3.981128 | 1.807652 | 0.474632 | 3.808531 | 0.00014 | 0.000771 | up |
| SLC6A19 | 2.146783 | 2.65029 | 0.697056 | 3.802118 | 0.000143 | 0.000789 | up |
| CABP5 | 30.6701 | 1.253919 | 0.329882 | 3.80111 | 0.000144 | 0.000791 | up |
| CHRNA5 | 3.856083 | 1.334179 | 0.351158 | 3.799365 | 0.000145 | 0.000796 | up |
| IGKV2D-26 | 7.248371 | 2.766231 | 0.72821 | 3.798674 | 0.000145 | 0.000798 | up |
| SCAMP5 | 32.17227 | 1.738054 | 0.457858 | 3.796057 | 0.000147 | 0.000805 | up |
| RNU2-6P | 4.355055 | -1.83414 | 0.483952 | -3.78991 | 0.000151 | 0.00082 | down |
| CDKN2B-AS1 | 11.98562 | 1.064349 | 0.281159 | 3.785575 | 0.000153 | 0.000834 | up |
| ANKRD33B | 48.93444 | 1.016266 | 0.268676 | 3.782491 | 0.000155 | 0.000842 | up |
| IFI44 | 695.2423 | 1.240487 | 0.328087 | 3.780968 | 0.000156 | 0.000847 | up |
| IGLV1-50 | 2.648557 | 2.607022 | 0.690229 | 3.77704 | 0.000159 | 0.00086 | up |
| PLIN4 | 13.77801 | 1.060906 | 0.281099 | 3.774135 | 0.000161 | 0.000867 | up |
| CDH5 | 11.32087 | 1.476054 | 0.391583 | 3.769454 | 0.000164 | 0.000882 | up |
| PTGFR | 6.985003 | 1.637022 | 0.434398 | 3.768487 | 0.000164 | 0.000884 | up |
| STAC | 10.18107 | 1.317039 | 0.349484 | 3.768524 | 0.000164 | 0.000884 | up |
| NRN1 | 6.770606 | 1.76706 | 0.469747 | 3.761731 | 0.000169 | 0.000904 | up |
| TEX9 | 13.87418 | 1.057665 | 0.281257 | 3.760493 | 0.00017 | 0.000907 | up |
| OLFM1 | 148.6107 | -1.85065 | 0.49236 | -3.75874 | 0.000171 | 0.000913 | down |
| A2ML1 | 4.350836 | 1.692655 | 0.450397 | 3.758136 | 0.000171 | 0.000915 | up |
| PKD1P5 | 18.62772 | -3.00765 | 0.800538 | -3.75704 | 0.000172 | 0.000918 | down |
| ADCY10 | 3.308975 | 1.667321 | 0.444279 | 3.752873 | 0.000175 | 0.000931 | up |
| TMEM17 | 10.20477 | 1.079149 | 0.287654 | 3.751554 | 0.000176 | 0.000934 | up |
| KCND1 | 18.04595 | 1.017194 | 0.271197 | 3.750757 | 0.000176 | 0.000937 | up |
| MUC16 | 14.06366 | 1.822947 | 0.486493 | 3.747114 | 0.000179 | 0.000948 | up |
| TLCD4 | 14.96075 | 1.886561 | 0.504022 | 3.743015 | 0.000182 | 0.000961 | up |
| ZFHX2 | 4.487407 | 1.350789 | 0.360982 | 3.741981 | 0.000183 | 0.000965 | up |
| ARG1 | 13.43128 | 1.263501 | 0.33779 | 3.740497 | 0.000184 | 0.00097 | up |
| MYCN | 4.280101 | 2.090372 | 0.559258 | 3.737761 | 0.000186 | 0.000978 | up |
| MYO5B | 13.43174 | 1.179727 | 0.316115 | 3.73195 | 0.00019 | 0.001 | up |
| WASH7P | 4.912882 | 2.343732 | 0.629079 | 3.725655 | 0.000195 | 0.001023 | up |
| PAX6 | 3.163718 | 1.920358 | 0.515788 | 3.723155 | 0.000197 | 0.001032 | up |
| STAB1 | 1763.597 | 1.029251 | 0.276596 | 3.721131 | 0.000198 | 0.001039 | up |
| IL22 | 2.966557 | 2.475862 | 0.66561 | 3.719689 | 0.000199 | 0.001044 | up |
| LGALSL | 161.2355 | 1.11778 | 0.300516 | 3.71954 | 0.0002 | 0.001044 | up |
| KCNG2 | 17.84865 | 1.530762 | 0.412196 | 3.713675 | 0.000204 | 0.001066 | up |
| FOSB | 7618.263 | -1.13687 | 0.306231 | -3.71246 | 0.000205 | 0.00107 | down |
| CCDC184 | 27.16363 | -1.50847 | 0.4064 | -3.7118 | 0.000206 | 0.001072 | down |
| PGLYRP1 | 1.518108 | 2.609308 | 0.703302 | 3.710081 | 0.000207 | 0.001078 | up |
| LINC01485 | 5.388007 | 1.563288 | 0.421506 | 3.708815 | 0.000208 | 0.001082 | up |
| SMIM10 | 5.618453 | 1.21619 | 0.328143 | 3.70628 | 0.00021 | 0.001091 | up |
| ABCC3 | 853.0487 | 1.01647 | 0.274528 | 3.702611 | 0.000213 | 0.001105 | up |
| LINC00472 | 3.360655 | 2.080648 | 0.563114 | 3.694895 | 0.00022 | 0.001133 | up |
| PLK2 | 539.9041 | -1.3788 | 0.373824 | -3.68838 | 0.000226 | 0.001158 | down |
| BEGAIN | 24.59584 | -1.14559 | 0.310961 | -3.68403 | 0.00023 | 0.001175 | down |
| NT5C3AP1 | 7.048674 | 1.194535 | 0.324336 | 3.683023 | 0.00023 | 0.001179 | up |
| SHH | 4.162497 | 1.303805 | 0.354063 | 3.682409 | 0.000231 | 0.00118 | up |
| ANXA8 | 3.349207 | 2.260076 | 0.615067 | 3.674523 | 0.000238 | 0.001212 | up |
| EXOC3L2 | 14.07416 | 1.563283 | 0.425614 | 3.673002 | 0.00024 | 0.001218 | up |
| SPDYA | 17.93509 | -1.13555 | 0.309648 | -3.66722 | 0.000245 | 0.001242 | down |
| RNF182 | 4.453876 | 3.347077 | 0.914686 | 3.659264 | 0.000253 | 0.001276 | up |
| RDH12 | 3.954526 | 1.384983 | 0.37864 | 3.657778 | 0.000254 | 0.001282 | up |
| MX1 | 4251.556 | 1.566854 | 0.428467 | 3.656883 | 0.000255 | 0.001286 | up |
| DIPK1B | 65.71643 | 1.368216 | 0.374207 | 3.656309 | 0.000256 | 0.001289 | up |
| KCNJ6 | 4.339864 | 2.140734 | 0.585932 | 3.653556 | 0.000259 | 0.001301 | up |
| LRRC2 | 7.053923 | 1.197419 | 0.328071 | 3.649874 | 0.000262 | 0.001317 | up |
| ENKUR | 69.73545 | 1.120639 | 0.307254 | 3.647267 | 0.000265 | 0.00133 | up |
| ZNF215 | 17.54215 | 1.739633 | 0.477218 | 3.645365 | 0.000267 | 0.001337 | up |
| ILDR2 | 8.151109 | 1.192199 | 0.32704 | 3.645419 | 0.000267 | 0.001337 | up |
| ALPK3 | 3.949832 | 1.407142 | 0.386124 | 3.644278 | 0.000268 | 0.001342 | up |
| CPNE4 | 3.637435 | 1.826899 | 0.501648 | 3.641798 | 0.000271 | 0.001354 | up |
| IGLC7 | 67.60505 | 2.061145 | 0.566256 | 3.639955 | 0.000273 | 0.001362 | up |
| SORCS3 | 18.63541 | -1.24009 | 0.34089 | -3.63781 | 0.000275 | 0.001371 | down |
| WT1 | 3.366983 | 2.153203 | 0.592042 | 3.636909 | 0.000276 | 0.001374 | up |
| LOC101928489 | 3.872948 | 1.530988 | 0.420943 | 3.637043 | 0.000276 | 0.001374 | up |
| GALNT18 | 6.394768 | 1.369255 | 0.376482 | 3.636973 | 0.000276 | 0.001374 | up |
| SMG1P1 | 33.79885 | -1.65092 | 0.453886 | -3.63729 | 0.000276 | 0.001374 | down |
| PHF24 | 5.183287 | 1.335241 | 0.367333 | 3.634958 | 0.000278 | 0.001383 | up |
| TRPM3 | 4.399441 | 2.114044 | 0.581606 | 3.634837 | 0.000278 | 0.001383 | up |
| CD300LD-AS1 | 3.764166 | 1.777434 | 0.489323 | 3.632435 | 0.000281 | 0.001394 | up |
| SIGLEC1 | 1459.244 | 2.08119 | 0.573012 | 3.632019 | 0.000281 | 0.001396 | up |
| MCEMP1 | 969.3112 | 1.268731 | 0.349553 | 3.629584 | 0.000284 | 0.001405 | up |
| MUC5B | 4.143201 | 1.844412 | 0.508385 | 3.627982 | 0.000286 | 0.001412 | up |
| WASF3 | 10.55328 | 1.552922 | 0.428046 | 3.627935 | 0.000286 | 0.001412 | up |
| SHE | 8.758799 | 1.523092 | 0.41984 | 3.627794 | 0.000286 | 0.001413 | up |
| VEGFC | 7.212802 | 2.02042 | 0.557379 | 3.624859 | 0.000289 | 0.001427 | up |
| SEMA3B | 4.898922 | 1.268713 | 0.350186 | 3.622969 | 0.000291 | 0.001436 | up |
| KLRC4 | 7.403615 | -1.17858 | 0.325311 | -3.62293 | 0.000291 | 0.001436 | down |
| RSPH1 | 3.897503 | 1.656239 | 0.457731 | 3.618367 | 0.000296 | 0.001459 | up |
| RAB13 | 7.152018 | 1.371163 | 0.379083 | 3.617052 | 0.000298 | 0.001464 | up |
| HOXB7 | 8.220622 | 1.243586 | 0.343988 | 3.615206 | 0.0003 | 0.001473 | up |
| CH25H | 15.72126 | 1.657778 | 0.458656 | 3.614427 | 0.000301 | 0.001476 | up |
| SPDYC | 13.09843 | 2.639811 | 0.730888 | 3.611788 | 0.000304 | 0.00149 | up |
| IGLL1 | 6.231446 | 1.887348 | 0.522913 | 3.609296 | 0.000307 | 0.001502 | up |
| LOC157273 | 8.327469 | 1.430448 | 0.396686 | 3.605998 | 0.000311 | 0.001518 | up |
| LOC101929128 | 8.327469 | 1.430448 | 0.396686 | 3.605998 | 0.000311 | 0.001518 | up |
| ASMT | 4.018241 | 1.503845 | 0.417468 | 3.602296 | 0.000315 | 0.001537 | up |
| ERICH3 | 6.248747 | 1.916469 | 0.532297 | 3.600376 | 0.000318 | 0.001547 | up |
| FAIM2 | 4.173516 | 1.41926 | 0.394254 | 3.599859 | 0.000318 | 0.00155 | up |
| CPXM1 | 33.27018 | 1.276552 | 0.354774 | 3.598208 | 0.00032 | 0.001558 | up |
| TEX41 | 8.639981 | 1.166513 | 0.324465 | 3.595183 | 0.000324 | 0.001575 | up |
| INKA1 | 71.8277 | 1.120952 | 0.311983 | 3.592991 | 0.000327 | 0.001584 | up |
| SCAT8 | 8.502217 | -1.09003 | 0.303498 | -3.59157 | 0.000329 | 0.001592 | down |
| ATP2B2 | 3.330105 | 1.733927 | 0.482869 | 3.590884 | 0.00033 | 0.001595 | up |
| MIR100HG | 3.947484 | 1.717531 | 0.478916 | 3.586291 | 0.000335 | 0.001621 | up |
| TIFAB | 15.7341 | -1.07723 | 0.300794 | -3.58129 | 0.000342 | 0.001646 | down |
| TRIM10 | 13.71357 | 1.944053 | 0.542872 | 3.581052 | 0.000342 | 0.001647 | up |
| EFCC1 | 9.861149 | 1.074036 | 0.299943 | 3.580795 | 0.000343 | 0.001647 | up |
| SMPDL3B | 9.168094 | 1.123633 | 0.313898 | 3.579616 | 0.000344 | 0.001654 | up |
| ABO | 50.31547 | 1.651774 | 0.461452 | 3.579511 | 0.000344 | 0.001655 | up |
| RBPMS2 | 54.55207 | 1.803325 | 0.504526 | 3.574295 | 0.000351 | 0.001685 | up |
| FCRL5 | 550.2316 | 1.102585 | 0.30858 | 3.573093 | 0.000353 | 0.00169 | up |
| LINC02323 | 5.345042 | 1.099329 | 0.307713 | 3.572581 | 0.000353 | 0.001693 | up |
| MIR3681HG | 8.362721 | 1.687576 | 0.472533 | 3.571339 | 0.000355 | 0.001699 | up |
| TDRP | 46.13252 | 1.158922 | 0.324935 | 3.566622 | 0.000362 | 0.001726 | up |
| LINC01010 | 6.031094 | 1.657397 | 0.465378 | 3.561397 | 0.000369 | 0.001756 | up |
| NTRK1 | 14.05423 | 1.00018 | 0.281039 | 3.558869 | 0.000372 | 0.001771 | up |
| RAPGEF5 | 14.60063 | 1.040539 | 0.292614 | 3.556008 | 0.000377 | 0.001788 | up |
| COLCA1 | 3.623415 | 1.941956 | 0.547166 | 3.549119 | 0.000387 | 0.00183 | up |
| TIMP4 | 2.196395 | 3.988253 | 1.125189 | 3.544519 | 0.000393 | 0.001861 | up |
| COLCA2 | 3.467666 | 2.398799 | 0.676941 | 3.543584 | 0.000395 | 0.001867 | up |
| MIR193BHG | 3.404295 | 1.860917 | 0.525223 | 3.543097 | 0.000395 | 0.001869 | up |
| LAMA3 | 5.463583 | 1.648366 | 0.465241 | 3.543039 | 0.000396 | 0.001869 | up |
| SCIN | 15.42869 | 1.142209 | 0.322511 | 3.541614 | 0.000398 | 0.001878 | up |
| LYVE1 | 7.443839 | 1.777658 | 0.502702 | 3.53621 | 0.000406 | 0.001914 | up |
| RNF208 | 4.578561 | 1.706237 | 0.483053 | 3.532194 | 0.000412 | 0.00194 | up |
| MYT1L | 7.546285 | 1.743005 | 0.493603 | 3.531184 | 0.000414 | 0.001946 | up |
| EHD4-AS1 | 7.510112 | 1.093156 | 0.309572 | 3.531189 | 0.000414 | 0.001946 | up |
| DNAH5 | 4.050315 | 1.982737 | 0.561525 | 3.530983 | 0.000414 | 0.001947 | up |
| TIMP3 | 17.66148 | 1.465289 | 0.415924 | 3.522974 | 0.000427 | 0.002002 | up |
| PDE3A | 21.14737 | 1.535334 | 0.4368 | 3.51496 | 0.00044 | 0.002057 | up |
| FCGR1B | 74.67512 | 1.057282 | 0.301197 | 3.510267 | 0.000448 | 0.002089 | up |
| SCG5 | 4.615264 | 1.207207 | 0.344269 | 3.506584 | 0.000454 | 0.002117 | up |
| HESX1 | 11.88286 | 1.61317 | 0.46007 | 3.50636 | 0.000454 | 0.002119 | up |
| CD83 | 5002.336 | -1.57733 | 0.450046 | -3.50482 | 0.000457 | 0.002127 | down |
| COL7A1 | 13.31694 | 1.022463 | 0.291755 | 3.504527 | 0.000457 | 0.002129 | up |
| CADM2 | 8.747986 | 1.436228 | 0.409973 | 3.503225 | 0.00046 | 0.002138 | up |
| ABCG8 | 4.593674 | 1.823842 | 0.521396 | 3.498001 | 0.000469 | 0.002176 | up |
| FREM1 | 7.848633 | 1.930522 | 0.552023 | 3.49718 | 0.00047 | 0.002181 | up |
| FEM1C | 496.851 | -1.1202 | 0.320538 | -3.49474 | 0.000475 | 0.002198 | down |
| HSPA4L | 4.727311 | 1.51435 | 0.43347 | 3.493549 | 0.000477 | 0.002206 | up |
| DRC7 | 9.773724 | 1.13146 | 0.323973 | 3.49245 | 0.000479 | 0.002215 | up |
| OXCT2 | 3.264867 | 1.565153 | 0.44893 | 3.486411 | 0.00049 | 0.002257 | up |
| NENFP1 | 3.563691 | 1.140335 | 0.327099 | 3.486203 | 0.00049 | 0.002258 | up |
| ESYT3 | 3.843338 | 1.358823 | 0.390193 | 3.482439 | 0.000497 | 0.002282 | up |
| ZNF252P-AS1 | 11.42709 | -1.01986 | 0.292858 | -3.48244 | 0.000497 | 0.002282 | down |
| OPRM1 | 4.524868 | 1.580456 | 0.453974 | 3.481381 | 0.000499 | 0.002291 | up |
| NFASC | 6.863027 | 1.326376 | 0.381283 | 3.478719 | 0.000504 | 0.00231 | up |
| RIMS2 | 3.440615 | 1.894996 | 0.546421 | 3.468014 | 0.000524 | 0.002391 | up |
| WSCD2 | 5.367256 | 1.765551 | 0.509167 | 3.467527 | 0.000525 | 0.002394 | up |
| NOTCH3 | 22.27488 | 1.712734 | 0.494055 | 3.46669 | 0.000527 | 0.0024 | up |
| IGKV6-21 | 40.67062 | 2.324173 | 0.670655 | 3.465528 | 0.000529 | 0.002409 | up |
| CA14 | 9.506779 | 1.045042 | 0.301612 | 3.464862 | 0.000531 | 0.002413 | up |
| GNG4 | 3.231956 | 1.583299 | 0.457081 | 3.463935 | 0.000532 | 0.00242 | up |
| GRIK5 | 3.770272 | 1.785831 | 0.515626 | 3.463423 | 0.000533 | 0.002424 | up |
| TEX29 | 5.091278 | 1.178831 | 0.34048 | 3.462263 | 0.000536 | 0.002431 | up |
| CCIN | 12.59906 | 1.105581 | 0.319332 | 3.462165 | 0.000536 | 0.002431 | up |
| SLC6A20 | 3.045144 | 1.501343 | 0.433667 | 3.461972 | 0.000536 | 0.002432 | up |
| PPM1K-DT | 3.511481 | 1.50934 | 0.436076 | 3.461185 | 0.000538 | 0.002437 | up |
| MYCT1 | 14.80413 | 1.377556 | 0.398001 | 3.461187 | 0.000538 | 0.002437 | up |
| QRSL1P3 | 30.30908 | -1.32016 | 0.381743 | -3.45825 | 0.000544 | 0.00246 | down |
| ZNF331 | 2675.727 | -1.67602 | 0.484815 | -3.45704 | 0.000546 | 0.002471 | down |
| SMIM43 | 8.775714 | 1.447613 | 0.418958 | 3.455268 | 0.00055 | 0.002485 | up |
| TLN2 | 15.30512 | 1.032049 | 0.299117 | 3.450323 | 0.00056 | 0.002526 | up |
| SAMMSON | 3.421825 | 2.208915 | 0.640724 | 3.447529 | 0.000566 | 0.00255 | up |
| MGAT3 | 12.14153 | 1.053772 | 0.305788 | 3.446087 | 0.000569 | 0.002562 | up |
| CCL20 | 87.43023 | -2.45935 | 0.714158 | -3.44371 | 0.000574 | 0.002581 | down |
| INTU | 7.173617 | 1.22822 | 0.356898 | 3.44138 | 0.000579 | 0.002599 | up |
| MGAM2 | 3.634031 | 1.458795 | 0.424334 | 3.437844 | 0.000586 | 0.002626 | up |
| PHYHD1 | 21.542 | 1.17787 | 0.342787 | 3.436159 | 0.00059 | 0.00264 | up |
| MUC17 | 4.989565 | 2.04792 | 0.596194 | 3.434991 | 0.000593 | 0.002651 | up |
| ACSL1 | 3203.647 | 1.057684 | 0.307928 | 3.434845 | 0.000593 | 0.002651 | up |
| ETV7 | 120.2749 | 1.446145 | 0.421115 | 3.434088 | 0.000595 | 0.002657 | up |
| TRAJ44 | 5.870431 | -1.05574 | 0.307471 | -3.43362 | 0.000596 | 0.002661 | down |
| CFAP58 | 3.694963 | 1.451644 | 0.422863 | 3.432896 | 0.000597 | 0.002666 | up |
| EEF1A1P10 | 16.80466 | -1.04907 | 0.305603 | -3.43278 | 0.000597 | 0.002667 | down |
| EPAS1 | 16.84952 | 1.0198 | 0.297208 | 3.431266 | 0.000601 | 0.00268 | up |
| MAFG-DT | 3.749784 | 1.386955 | 0.404681 | 3.427278 | 0.00061 | 0.002716 | up |
| ARMC3 | 11.24701 | 1.242106 | 0.362456 | 3.426911 | 0.00061 | 0.002718 | up |
| FGF13 | 3.440922 | 1.356597 | 0.396076 | 3.425091 | 0.000615 | 0.002733 | up |
| PHKA1 | 12.10358 | 1.492887 | 0.435995 | 3.424091 | 0.000617 | 0.00274 | up |
| DRICH1 | 3.686666 | 1.11049 | 0.324512 | 3.422032 | 0.000622 | 0.002758 | up |
| PNMT | 3.825878 | 1.443826 | 0.422723 | 3.415534 | 0.000637 | 0.002815 | up |
| ABCA13 | 7.585438 | 1.333629 | 0.39138 | 3.407505 | 0.000656 | 0.002887 | up |
| PLEK2 | 20.09216 | 1.181534 | 0.34785 | 3.396672 | 0.000682 | 0.002986 | up |
| SGIP1 | 4.372275 | 1.582746 | 0.466058 | 3.396029 | 0.000684 | 0.002989 | up |
| PRSS21 | 45.93844 | -1.74917 | 0.51507 | -3.39597 | 0.000684 | 0.002989 | down |
| SIM2 | 3.822143 | 1.13956 | 0.33569 | 3.394674 | 0.000687 | 0.003 | up |
| SVEP1 | 4.720128 | 1.821995 | 0.537563 | 3.389361 | 0.000701 | 0.003053 | up |
| TENM2 | 3.388989 | 1.774332 | 0.523846 | 3.387127 | 0.000706 | 0.003076 | up |
| CYB5R2 | 24.23857 | 1.069199 | 0.315779 | 3.385905 | 0.000709 | 0.003087 | up |
| EPSTI1 | 1007.933 | 1.096711 | 0.324299 | 3.381785 | 0.00072 | 0.003126 | up |
| KRT80 | 16.18186 | 1.517957 | 0.448887 | 3.381603 | 0.000721 | 0.003126 | up |
| MAGI1 | 4.925852 | 1.644033 | 0.48641 | 3.379932 | 0.000725 | 0.003141 | up |
| HSD3B7 | 20.89835 | 1.053731 | 0.312457 | 3.372398 | 0.000745 | 0.003215 | up |
| LINC02864 | 2.635492 | 2.72759 | 0.808901 | 3.371972 | 0.000746 | 0.003219 | up |
| DSC1 | 53.55681 | -1.40467 | 0.416582 | -3.3719 | 0.000747 | 0.003219 | down |
| COLGALT2 | 42.7314 | -1.11342 | 0.330381 | -3.37012 | 0.000751 | 0.003238 | down |
| PRRT1 | 8.791614 | -1.18194 | 0.350816 | -3.36912 | 0.000754 | 0.003248 | down |
| LOC100507547 | 8.791614 | -1.18194 | 0.350816 | -3.36912 | 0.000754 | 0.003248 | down |
| ERRFI1 | 35.56975 | -1.47892 | 0.439026 | -3.36865 | 0.000755 | 0.003252 | down |
| CREB3L3 | 4.438657 | 1.942336 | 0.576992 | 3.366311 | 0.000762 | 0.003272 | up |
| SNORA5A | 3.1291 | -1.25519 | 0.373939 | -3.35666 | 0.000789 | 0.003368 | down |
| LSMEM1 | 23.85386 | 1.167487 | 0.34794 | 3.355422 | 0.000792 | 0.003378 | up |
| GPR20 | 20.78023 | -1.20052 | 0.357967 | -3.35372 | 0.000797 | 0.003397 | down |
| ANKRD34B | 14.88192 | 1.861504 | 0.555315 | 3.352156 | 0.000802 | 0.003416 | up |
| MALRD1 | 5.918039 | 1.425974 | 0.426313 | 3.344895 | 0.000823 | 0.003496 | up |
| CD163 | 3833.555 | 1.380396 | 0.41286 | 3.343497 | 0.000827 | 0.00351 | up |
| OLFML2A | 7.823503 | 1.79184 | 0.536158 | 3.342001 | 0.000832 | 0.003524 | up |
| FLT3 | 487.5537 | 1.281729 | 0.3838 | 3.339571 | 0.000839 | 0.003549 | up |
| RHBDF1 | 4.595709 | 1.879438 | 0.563138 | 3.337439 | 0.000846 | 0.003573 | up |
| PLCH1 | 12.35479 | 1.348278 | 0.404684 | 3.331682 | 0.000863 | 0.003638 | up |
| MFAP3L | 224.0141 | 1.04024 | 0.312491 | 3.328861 | 0.000872 | 0.003671 | up |
| LOC101928198 | 224.0141 | 1.04024 | 0.312491 | 3.328861 | 0.000872 | 0.003671 | up |
| SEMA3F | 4.760503 | 2.063434 | 0.620272 | 3.326658 | 0.000879 | 0.003694 | up |
| LPIN3 | 4.870573 | 1.220952 | 0.367437 | 3.322884 | 0.000891 | 0.003732 | up |
| KLB | 6.613194 | 1.060121 | 0.319214 | 3.321034 | 0.000897 | 0.003752 | up |
| NDUFA4L2 | 5.037652 | 1.243102 | 0.374446 | 3.31984 | 0.000901 | 0.003764 | up |
| ISG15 | 2376.309 | 1.180458 | 0.355571 | 3.319891 | 0.000901 | 0.003764 | up |
| REEP1 | 5.570919 | 1.754319 | 0.52864 | 3.318553 | 0.000905 | 0.00378 | up |
| AQP9 | 1306.981 | 1.079946 | 0.325615 | 3.316639 | 0.000911 | 0.003803 | up |
| TRBV16 | 2.094006 | -1.44096 | 0.435009 | -3.31248 | 0.000925 | 0.003851 | down |
| FOLR3 | 670.661 | 1.505988 | 0.454662 | 3.312327 | 0.000925 | 0.003852 | up |
| MYZAP | 28.16908 | 1.490763 | 0.450612 | 3.308308 | 0.000939 | 0.003902 | up |
| CRHBP | 15.30076 | 1.469133 | 0.444388 | 3.30597 | 0.000946 | 0.003929 | up |
| KRT73-AS1 | 14.43103 | -1.13117 | 0.342248 | -3.3051 | 0.000949 | 0.003939 | down |
| COL1A2 | 9.741424 | 2.395364 | 0.724843 | 3.304666 | 0.000951 | 0.003944 | up |
| PLA2G7 | 216.0597 | -1.01993 | 0.308875 | -3.30207 | 0.00096 | 0.003973 | down |
| TRAJ2 | 4.545716 | -1.14242 | 0.345969 | -3.3021 | 0.00096 | 0.003973 | down |
| NEU4 | 19.60669 | 1.609218 | 0.487584 | 3.30039 | 0.000966 | 0.003993 | up |
| GREB1 | 5.16911 | 1.271171 | 0.385218 | 3.299872 | 0.000967 | 0.003998 | up |
| GPR141BP | 4.908746 | 1.15042 | 0.348762 | 3.298584 | 0.000972 | 0.004013 | up |
| PLCB4 | 5.347257 | 1.213715 | 0.3681 | 3.297239 | 0.000976 | 0.004029 | up |
| RPL7AP45 | 6.472861 | -1.00352 | 0.304536 | -3.29523 | 0.000983 | 0.004054 | down |
| BCAR1 | 3.438578 | 1.803278 | 0.547251 | 3.295159 | 0.000984 | 0.004054 | up |
| KCNA5 | 9.020947 | -1.1909 | 0.361593 | -3.29349 | 0.00099 | 0.004076 | down |
| VTRNA1-3 | 3.025808 | 1.424183 | 0.432543 | 3.29258 | 0.000993 | 0.004085 | up |
| FRAS1 | 5.914106 | 2.057543 | 0.62519 | 3.291069 | 0.000998 | 0.004104 | up |
| CNTN2 | 5.201318 | 1.562593 | 0.475728 | 3.284638 | 0.001021 | 0.004188 | up |
| HES1 | 51.71334 | -1.52943 | 0.46589 | -3.28282 | 0.001028 | 0.004207 | down |
| MT1L | 8.928929 | 1.832828 | 0.558589 | 3.281173 | 0.001034 | 0.004229 | up |
| SNORD17 | 4.856485 | 1.356326 | 0.413612 | 3.279223 | 0.001041 | 0.00425 | up |
| SLC22A16 | 112.8262 | 1.386467 | 0.4229 | 3.278474 | 0.001044 | 0.004259 | up |
| LGALS8-AS1 | 4.623824 | 1.030577 | 0.31487 | 3.273026 | 0.001064 | 0.004326 | up |
| LGALS3BP | 1782.833 | 1.030146 | 0.314771 | 3.272684 | 0.001065 | 0.00433 | up |
| ST7-AS1 | 4.059786 | 1.367524 | 0.418165 | 3.270295 | 0.001074 | 0.004362 | up |
| TNFAIP6 | 348.8176 | -2.05778 | 0.629522 | -3.26879 | 0.00108 | 0.004382 | down |
| CALN1 | 4.838533 | 1.638167 | 0.501898 | 3.263945 | 0.001099 | 0.004443 | up |
| MLF1 | 40.53533 | -1.06541 | 0.327087 | -3.25727 | 0.001125 | 0.004533 | down |
| NWD1 | 4.387198 | 1.509114 | 0.464095 | 3.251737 | 0.001147 | 0.004609 | up |
| MTCO2P12 | 6.136506 | 1.674075 | 0.515049 | 3.250321 | 0.001153 | 0.004623 | up |
| GLYATL1 | 5.020393 | 1.29327 | 0.398356 | 3.246519 | 0.001168 | 0.00468 | up |
| OLR1 | 285.7248 | -2.65688 | 0.818686 | -3.2453 | 0.001173 | 0.004696 | down |
| IL12A-AS1 | 3.612793 | 1.228826 | 0.378878 | 3.24333 | 0.001181 | 0.00472 | up |
| LINC00908 | 3.521039 | 1.430166 | 0.441843 | 3.236818 | 0.001209 | 0.004811 | up |
| LINC00683 | 3.521039 | 1.430166 | 0.441843 | 3.236818 | 0.001209 | 0.004811 | up |
| ATP2A1 | 8.168588 | 1.179366 | 0.364428 | 3.236208 | 0.001211 | 0.00482 | up |
| LOC646588 | 3.304176 | 1.536535 | 0.475325 | 3.232595 | 0.001227 | 0.004877 | up |
| IGLV1-41 | 30.7291 | 2.154576 | 0.667131 | 3.229613 | 0.00124 | 0.004921 | up |
| DNAH11 | 8.093486 | 1.029203 | 0.319344 | 3.222867 | 0.001269 | 0.005019 | up |
| CACHD1 | 23.5639 | -1.43677 | 0.446114 | -3.22064 | 0.001279 | 0.005051 | down |
| NAT8B | 23.85216 | 1.513434 | 0.47001 | 3.220002 | 0.001282 | 0.005061 | up |
| ATP9A | 113.21 | 1.026835 | 0.319208 | 3.216823 | 0.001296 | 0.005106 | up |
| LPO | 3.433399 | 1.475483 | 0.458947 | 3.214928 | 0.001305 | 0.005131 | up |
| ROBO4 | 4.695406 | 1.82274 | 0.56756 | 3.211538 | 0.00132 | 0.005181 | up |
| HGD | 31.95009 | 1.025768 | 0.319625 | 3.209281 | 0.001331 | 0.005215 | up |
| ID3 | 152.0303 | -1.01962 | 0.317843 | -3.20795 | 0.001337 | 0.005234 | down |
| IL1A | 29.74151 | -2.86206 | 0.892858 | -3.2055 | 0.001348 | 0.005269 | down |
| ERG | 13.77044 | 1.379562 | 0.430488 | 3.204648 | 0.001352 | 0.005281 | up |
| LOC101927787 | 3.46471 | 1.269719 | 0.396456 | 3.20267 | 0.001362 | 0.005315 | up |
| ATF3 | 999.9596 | -1.51817 | 0.474132 | -3.202 | 0.001365 | 0.005326 | down |
| TPRG1-AS1 | 23.31637 | 1.186183 | 0.371484 | 3.193091 | 0.001408 | 0.005477 | up |
| NRCAM | 32.51139 | -1.74238 | 0.546064 | -3.19079 | 0.001419 | 0.005511 | down |
| STXBP6 | 4.151467 | 1.132035 | 0.355234 | 3.18673 | 0.001439 | 0.005581 | up |
| ASS1P1 | 28.78937 | 1.001142 | 0.314336 | 3.18494 | 0.001448 | 0.005613 | up |
| ARMC9 | 19.77409 | 1.102446 | 0.346446 | 3.182162 | 0.001462 | 0.005654 | up |
| GPR137C | 4.372326 | 1.142927 | 0.359208 | 3.181798 | 0.001464 | 0.005658 | up |
| C3orf80 | 4.280183 | 1.827827 | 0.574481 | 3.1817 | 0.001464 | 0.005658 | up |
| RNU6-574P | 4.141326 | 1.383281 | 0.435051 | 3.179585 | 0.001475 | 0.005695 | up |
| ZNF702P | 35.34358 | -1.14884 | 0.361418 | -3.17871 | 0.001479 | 0.00571 | down |
| NFIB | 11.00197 | 1.382596 | 0.435236 | 3.176655 | 0.00149 | 0.005742 | up |
| CHRNA2 | 23.19338 | 1.710771 | 0.538744 | 3.175479 | 0.001496 | 0.005764 | up |
| LOC101927741 | 7.986788 | 1.293407 | 0.407383 | 3.174918 | 0.001499 | 0.005773 | up |
| ANK2 | 12.08957 | 1.425714 | 0.449388 | 3.172572 | 0.001511 | 0.005811 | up |
| ADCY6 | 16.33114 | 1.199834 | 0.378962 | 3.166104 | 0.001545 | 0.005918 | up |
| NR4A2 | 8605.351 | -1.48815 | 0.471241 | -3.15795 | 0.001589 | 0.00605 | down |
| TUBA8 | 7.141366 | 1.207262 | 0.382564 | 3.155713 | 0.001601 | 0.006093 | up |
| GLDN | 4.444541 | 1.402493 | 0.444585 | 3.154613 | 0.001607 | 0.006111 | up |
| CD248 | 99.22124 | -1.20909 | 0.383366 | -3.15388 | 0.001611 | 0.006123 | down |
| RSPH14 | 4.367515 | 1.120489 | 0.355303 | 3.153613 | 0.001613 | 0.006125 | up |
| LHFPL6 | 28.56168 | 1.249563 | 0.396859 | 3.14863 | 0.00164 | 0.006219 | up |
| KCNJ1 | 10.31406 | 1.059113 | 0.336452 | 3.147883 | 0.001645 | 0.006234 | up |
| SEC1P | 7.95687 | -1.47941 | 0.469998 | -3.14769 | 0.001646 | 0.006236 | down |
| KL | 13.13561 | -1.25703 | 0.399624 | -3.14553 | 0.001658 | 0.006274 | down |
| RNU6-645P | 3.427057 | 1.289518 | 0.410082 | 3.14454 | 0.001663 | 0.006294 | up |
| ZBTB21 | 501.9507 | -1.001 | 0.318407 | -3.14379 | 0.001668 | 0.006307 | down |
| MIR99AHG | 7.968875 | 1.306161 | 0.415509 | 3.143524 | 0.001669 | 0.006307 | up |
| TNR | 3.722195 | 1.15121 | 0.366409 | 3.141873 | 0.001679 | 0.006336 | up |
| COL5A3 | 19.16392 | -1.22094 | 0.388948 | -3.13907 | 0.001695 | 0.006391 | down |
| TRAJ42 | 3.598084 | -1.06717 | 0.340573 | -3.13346 | 0.001728 | 0.0065 | down |
| LINC00310 | 4.043881 | 1.108502 | 0.353877 | 3.132453 | 0.001734 | 0.006521 | up |
| SRSF12 | 10.90798 | -1.19557 | 0.382039 | -3.12946 | 0.001751 | 0.006574 | down |
| KIAA1549 | 6.241468 | 1.671086 | 0.534455 | 3.126707 | 0.001768 | 0.006627 | up |
| WNK2 | 4.600846 | 1.48758 | 0.476181 | 3.123983 | 0.001784 | 0.006683 | up |
| CTSG | 13.92123 | 2.595741 | 0.831354 | 3.122304 | 0.001794 | 0.006712 | up |
| TRAJ54 | 6.506107 | -1.00349 | 0.321809 | -3.11829 | 0.001819 | 0.006791 | down |
| PTPRH | 13.39907 | 1.538701 | 0.493825 | 3.115885 | 0.001834 | 0.006843 | up |
| GRM4 | 4.896367 | 1.467295 | 0.470936 | 3.115698 | 0.001835 | 0.006845 | up |
| GJB2 | 20.60253 | -2.95831 | 0.949797 | -3.11468 | 0.001841 | 0.006864 | down |
| CAPN11 | 9.231818 | 1.486418 | 0.477686 | 3.111707 | 0.00186 | 0.006916 | up |
| KANK2 | 35.82935 | 1.07701 | 0.346429 | 3.108893 | 0.001878 | 0.00697 | up |
| ADAMTSL3 | 3.286915 | 1.603652 | 0.515845 | 3.108789 | 0.001879 | 0.006971 | up |
| PHEX | 25.26496 | -1.30315 | 0.419267 | -3.10817 | 0.001883 | 0.006984 | down |
| PPFIA4 | 76.78121 | -1.21021 | 0.389639 | -3.10598 | 0.001896 | 0.007031 | down |
| LINC01750 | 5.352165 | 1.363074 | 0.438913 | 3.105568 | 0.001899 | 0.007037 | up |
| GCOM1 | 43.79146 | 1.437818 | 0.463107 | 3.104725 | 0.001905 | 0.007055 | up |
| CDH6 | 3.755082 | 1.833857 | 0.592112 | 3.097144 | 0.001954 | 0.007203 | up |
| TNF | 827.1577 | -1.23539 | 0.399779 | -3.09017 | 0.002 | 0.007352 | down |
| CDHR5 | 7.810578 | 1.121854 | 0.363225 | 3.088595 | 0.002011 | 0.007384 | up |
| MPO | 228.8482 | 1.194869 | 0.38739 | 3.084409 | 0.00204 | 0.007479 | up |
| PLA2G2C | 4.152228 | 1.348301 | 0.438165 | 3.077156 | 0.00209 | 0.007642 | up |
| LINC01206 | 4.420192 | 1.711289 | 0.55639 | 3.075699 | 0.0021 | 0.007671 | up |
| VNN1 | 914.887 | 1.169382 | 0.380208 | 3.075639 | 0.002101 | 0.007671 | up |
| PLAAT2 | 25.26713 | 1.326928 | 0.431678 | 3.073885 | 0.002113 | 0.007709 | up |
| GXYLT2 | 3.567493 | 1.152768 | 0.375054 | 3.073605 | 0.002115 | 0.007715 | up |
| SV2C | 3.82365 | 1.228223 | 0.399909 | 3.071258 | 0.002132 | 0.007771 | up |
| IGHV3-35 | 3.374484 | 1.496654 | 0.487722 | 3.068662 | 0.00215 | 0.007828 | up |
| ABI3BP | 3.796987 | 1.116756 | 0.364193 | 3.06639 | 0.002167 | 0.007881 | up |
| RORC | 71.07593 | -1.03544 | 0.337687 | -3.06629 | 0.002167 | 0.007882 | down |
| INKA2-AS1 | 5.885304 | 1.448306 | 0.473164 | 3.060899 | 0.002207 | 0.008003 | up |
| BEX1 | 7.238533 | 1.191343 | 0.389482 | 3.058788 | 0.002222 | 0.008051 | up |
| ARL4D | 137.6973 | -1.15887 | 0.37919 | -3.05616 | 0.002242 | 0.008108 | down |
| DUSP4 | 130.6456 | -1.12399 | 0.36811 | -3.05341 | 0.002263 | 0.008174 | down |
| PLA2G2D | 10.87757 | 1.072202 | 0.35171 | 3.048544 | 0.0023 | 0.008293 | up |
| KCNC1 | 5.464568 | 1.081776 | 0.355049 | 3.046835 | 0.002313 | 0.008333 | up |
| GRTP1 | 12.74044 | 1.252351 | 0.411256 | 3.045182 | 0.002325 | 0.008372 | up |
| WTIP | 4.186089 | 1.272781 | 0.418203 | 3.04345 | 0.002339 | 0.008411 | up |
| LOC339260 | 9.852188 | -1.42188 | 0.467188 | -3.04349 | 0.002339 | 0.008411 | down |
| CCDC144NL-AS1 | 9.852188 | -1.42188 | 0.467188 | -3.04349 | 0.002339 | 0.008411 | down |
| GOT2P6 | 4.236216 | 1.004661 | 0.330278 | 3.041865 | 0.002351 | 0.008452 | up |
| SHC3 | 4.016439 | 1.110313 | 0.365221 | 3.040114 | 0.002365 | 0.00849 | up |
| GPM6A | 26.34286 | -1.72532 | 0.567916 | -3.03799 | 0.002382 | 0.00854 | down |
| MIR6859-1 | 3.374374 | -1.09009 | 0.359632 | -3.03114 | 0.002436 | 0.008713 | down |
| COL6A3 | 43.90325 | 1.122858 | 0.371175 | 3.025144 | 0.002485 | 0.008854 | up |
| CPA3 | 9.080797 | 1.319914 | 0.436548 | 3.023528 | 0.002498 | 0.008892 | up |
| MAOA | 10.38144 | 1.628647 | 0.538814 | 3.022653 | 0.002506 | 0.008912 | up |
| ZNF704 | 4.041509 | 1.237312 | 0.409367 | 3.022503 | 0.002507 | 0.008914 | up |
| CCDC3 | 7.519485 | 2.007519 | 0.664808 | 3.019696 | 0.00253 | 0.008984 | up |
| DCSTAMP | 6.02095 | 1.320463 | 0.437671 | 3.017025 | 0.002553 | 0.009054 | up |
| XAF1 | 1318.187 | 1.014508 | 0.336754 | 3.012608 | 0.00259 | 0.009168 | up |
| NRARP | 89.6571 | -1.84909 | 0.61415 | -3.01081 | 0.002605 | 0.00921 | down |
| GLP1R | 3.88384 | 1.721452 | 0.572002 | 3.009522 | 0.002617 | 0.00924 | up |
| KNDC1 | 50.15048 | -1.19169 | 0.39625 | -3.00742 | 0.002635 | 0.009298 | down |
| C15orf65 | 5.036979 | 1.024251 | 0.340656 | 3.006701 | 0.002641 | 0.009316 | up |
| CGREF1 | 5.033879 | 1.661091 | 0.552964 | 3.003979 | 0.002665 | 0.009388 | up |
| PTPRVP | 4.775747 | 1.204895 | 0.401436 | 3.001462 | 0.002687 | 0.009447 | up |
| CMBL | 8.454393 | 1.176486 | 0.393847 | 2.987163 | 0.002816 | 0.009823 | up |
| KLF5 | 184.577 | 1.098296 | 0.3679 | 2.985311 | 0.002833 | 0.009872 | up |

Table S5. The result of DEGs in COVID-19 datasets.
