# Supplementary material for: Novel insight on marker genes and pathogenic peripheral neutrophil subtypes in acute pancreatitis
Source: Front Immunol. 2022 Aug 22;13:964622. doi: 10.3389/fimmu.2022.964622 (PMC9444397; doi:10.3389/fimmu.2022.964622)
Supplement: Supplementary file 8 [file Table_6.docx]

Table S6. Upregulated and downregulated metabolism pathways in group 0.

| Common upregulated pathway | Common downregulated pathway |
| --- | --- |
| Fatty acid degradation | N-Glycan biosynthesis |
| alpha-Linolenic acid metabolism | Fatty acid elongation |
| Sulfur metabolism | Citrate cycle (TCA cycle) |
| Fatty acid biosynthesis | Glycerolipid metabolism |
| Glycosaminoglycan biosynthesis - chondroitin sulfate / dermatan sulfate | |
| Ascorbate and aldarate metabolism | |
| Pantothenate and CoA biosynthesis | |
| Histidine metabolism | |
| Nicotinate and nicotinamide metabolism | |
| Glycosaminoglycan degradation | |
| Pentose phosphate pathway | |
